# Phase transition for random walks on graphs with added weighted random matching

**DOI:** 10.1007/s00440-024-01342-9

**Published:** 2024-11-28

**Authors:** Zsuzsanna Baran, Jonathan Hermon, Anđela Šarković, Perla Sousi

**Affiliations:** 1 University of Cambridge, Cambridge, UK; 2 University of British Columbia, Vancouver, Canada

**Keywords:** Random graph, Mixing time, Cutoff, Phase transition, Entropy, Quasi-trees

## Abstract

For a finite graph $G=(V,E)$ let $G^*$ be obtained by considering a random perfect matching of *V* and adding the corresponding edges to *G* with weight $\varepsilon$, while assigning weight 1 to the original edges of *G*. We consider whether for a sequence $(G_n)$ of graphs with bounded degrees and corresponding weights $(\varepsilon _n)$, the (weighted) random walk on $(G_n^*)$ has cutoff. For graphs with polynomial growth we show that $\log \left( \frac{1}{\varepsilon _n}\right) \ll \log |V_n|$ is a sufficient condition for cutoff. Under the additional assumption of vertex-transitivity we establish that this condition is also necessary. For graphs where the entropy of the simple random walk grows linearly up to some time of order $\log |V_n|$ we show that $\frac{1}{\varepsilon _n}\ll \log |V_n|$ is sufficient for cutoff. In the special case of expander graphs we also provide a complete picture for the complementary regime $\frac{1}{\varepsilon _n}\gtrsim \log |V_n|$.

**Supplementary Information:**

The online version contains supplementary material available at 10.1007/s00440-024-01342-9.

## Introduction

This paper is motivated by the question of what little random perturbation one can apply to a given sequence $(G_n)$ of graphs so that the random walk on the resulting graphs $(G_n^*)$ will exhibit cutoff whp.

### Definition 1.1

Let $(G_n=(V_n,E_n))$ be a sequence of connected graphs, with $|V_n|\rightarrow \infty$ as $n\rightarrow \infty$ and $|V_n|$ even for each *n*, and let $(\varepsilon _n)$ be a sequence taking values in (0, 1). We define the sequence $(G_n^*)$ of weighted random graphs as follows. We let $G_n^*$ have vertex set $V_n$ and edge set $E_n\sqcup E^*_n$ where $E^*_n$ is a uniformly random perfect matching of $V_n$, and let each edge in $E_n$ have weight 1 and each edge in $E^*_n$ have weight $\varepsilon _n$. (Note that $G_n^*$ might have multiple edges.) We refer to the edges in $E_n^*$ as *long-range edges*.

If $|V_n|$ is odd then we can define $G_n^*$ similarly, considering matchings of $V_n$ that leave one vertex unmatched. For simplicity we will assume in the proofs that $|V_n|$ is even.

An unweighted version of the above model (i.e. having $\varepsilon _n\equiv 1$) was considered in 2013 by Diaconis [13] who asked about the order of the mixing time of the random walks on $G_n^*$. In 2022 [18] proved that for a sequence of connected graphs $(G_n)$ with uniformly bounded degrees, the walks on $(G_n^*)$ exhibit cutoff whp at a time of order $\log |V_n|$.

If $\varepsilon _n$ is of constant order, it follows analogously to [18] that the walks on $(G_n^*)$ exhibit cutoff whp. On the other hand, it is easy to see intuitively that if $\varepsilon _n$ is sufficiently small so that by the mixing time of $G_n$ the walk is unlikely to cross any of the added edges $E^*_n$ of $G^*_n$, then the added edges do not affect the occurrence of cutoff, i.e. $(G_n^*)$ exhibits cutoff if and only if $(G_n)$ does. In this paper we seek to understand what happens between these two regimes.

Intuitively, one would expect that if the random walk on $(G_n)$ did not exhibit cutoff, and $\frac{1}{\varepsilon _n}\gg t_{\textrm{mix}}^{G^*_n}$ then the random walk on $G_n^*$ would not exhibit cutoff either. (This is because the walk would mix before it first crosses an added edge, and hence the mixing time would stay essentially the same.) So in order to introduce cutoff it is a necessary condition to have $t_{\textrm{mix}}^{G^*_n}\gtrsim \frac{1}{\varepsilon _n}$. A very closely related condition, that is often easily verifiable, would be that for any starting vertex the entropy of the first added edge crossed by the random walk is of strictly smaller order than $\log |V_n|$. (See Sect. 7 for more discussion on this condition.) In the case of two very large families of graphs (graphs with polynomial growth and graphs with linear growth of entropy) we prove that this latter assumption is sufficient for $(G_n^*)$ to have cutoff. In the case of two families (vertex-transitive graphs with polynomial growth and expander graphs) we also prove that if the entropy is of strictly larger order than $\log |V_n|$ then the added edges do not affect whether cutoff occurs, which can be viewed as a phase transition.

Before stating the main results, we recall the definition of total variation mixing time and cutoff.

Given a finite connected graph *G* we define the total variation mixing time of the discrete-time random walk *X* on *G* as follows. Let *P* be the transition matrix of *X* and let $\pi$ be the corresponding invariant distribution. Then for any $\theta \in (0,1)$ we define

$$\begin{aligned}t_{\textrm{mix}}^G(\theta ):=\min \left\{ t:d_{\textrm{TV}}\left( {P^t(x,\cdot )},{\pi (\cdot )}\right) \le \theta \text { for all }x\right\} ,\end{aligned}$$

where the total variation distance $d_{\textrm{TV}}\left( {\mu },{\nu }\right)$ of distributions $\mu$ and $\nu$ on state space *S* is defined as

$$\begin{aligned}d_{\textrm{TV}}\left( {\mu },{\nu }\right) := \frac{1}{2}\sum _{x\in S}\left| \mu (x)-\nu (x)\right| .\end{aligned}$$

We sometimes write $t_{\textrm{mix}}^G$ without specifying $\theta$ to mean $t_{\textrm{mix}}^G\left( \frac{1}{4}\right)$.

We say that a sequence $(G_n)$ of graphs exhibits cutoff at time $t_n$ with window of order $s_n$ if $\frac{s_n}{t_n}\rightarrow 0$ as $n\rightarrow \infty$, and for every $\theta \in (0,1)$ there exists a constant $c(\theta )$ such that for all *n* we have

1.1 $$\begin{aligned} t_n-c(\theta )s_n \le t_{\textrm{mix}}^{G_n}(\theta )\le t_n+c(\theta )s_n. \end{aligned}$$

For a random sequence $(G_n)$ of graphs we say that $(G_n)$ exhibits cutoff with high probability (whp) if (1.1) holds with probability tending to 1 as $n\rightarrow \infty$. The lazy version of a random walk with transition matrix *P* is defined to have transition matrix $P_{\textrm{lazy}}:=\frac{1}{2}(I+P)$. For lazy random walks we define the mixing time and cutoff analogously and write *lazy* in the superscript.

We also recall that the entropy of a random variable *W* taking values in a countable set $\mathcal {W}$ is defined as

$$\begin{aligned}H(W):=\sum _{w\in \mathcal {W}}\mathbb {P}\!\left( W=w\right) \left( -\log \mathbb {P}\!\left( W=w\right) \right) ,\end{aligned}$$

where $p(-\log p)$ is considered to be 0 when $p=0$.

Given two functions $f,g:\mathbb {Z}_{\ge 0}\rightarrow \mathbb {R}_{>0}$ or $f,g:\mathbb {R}_{>0}\rightarrow \mathbb {R}_{>0}$ we write $f(t)\lesssim g(t)$ if there exists a constant $c>0$ such that we have $f(t)\le cg(t)$ for all sufficiently large values of *t*. We write $f(t)\ll g(t)$ if we have $\frac{f(t)}{g(t)}\rightarrow 0$ as $t\rightarrow \infty$. We define $\gtrsim$ and $\gg$ analogously. We write $f(t)\asymp g(t)$ if we have $f(t)\lesssim g(t)$ and $f(t)\gtrsim g(t)$. In case *f*, *g* take negative values, we write $f(t)\lesssim g(t)$ if $(-f(t))\gtrsim (-g(t))$, and we define $\gtrsim$, $\ll$, $\gg$ and $\asymp$ analogously.

### Results

Now we state the main results of the paper.

#### Theorem 1

Let $\Delta$, *a*, *b* and *c* be given positive constants. Let $(\varepsilon _n)$ be a sequence of constants in (0, 1) and let $(G_n)$ be a sequence of connected graphs with the following properties: $|V_n|\rightarrow \infty$ as $n\rightarrow \infty$, all vertices have degree $\le \Delta$, and for any *n* and any $x_n\in V_n$ the entropy of the simple random walk on $G_n$ from $x_n$ at any time $t\le a\log |V_n|$ satisfies $H(X_t)\in [bt,ct]$. Let $G_n^*$ be as in Definition 1.1.

- If $\varepsilon _n\gg \frac{1}{\log |V_n|}$ then the random walk on $(G_n^*)$ exhibits cutoff whp, at a time of order $\log |V_n|$.
- If $\varepsilon _n\ll \frac{1}{t_{\textrm{mix}}^{G_n}(\theta )}$ for some $\theta \in (0,1)$, then the random walk on $(G_n^*)$ exhibits cutoff if and only if the random walk on $(G_n)$ does.
- If $\varepsilon _n\asymp \frac{1}{t_{\textrm{mix}}^{G_n,\textrm{lazy}}\left( \frac{1}{4}\right) }$ and the lazy random walk on $(G_n)$ does not exhibit cutoff, then neither does the random walk on $(G_n^*)$.

#### Theorem 2

Let $\Delta$ be a given positive constant and let *p* be a given polynomial. Let $(\varepsilon _n)$ be a sequence of constants in (0, 1) and let $(G_n)$ be a sequence of connected graphs with the following properties: $|V_n|\rightarrow \infty$ as $n\rightarrow \infty$, all vertices have degree $\le \Delta$, and for any *r* the volume of any ball of radius *r* in any $G_n$ is upper bounded by *p*(*r*). Let $G_n^*$ be as in Definition 1.1.

- If $\varepsilon _n\gtrsim |V_n|^{-o(1)}$ then the random walk on $(G_n^*)$ exhibits cutoff with high probability, at a time of order $\frac{1}{\varepsilon _n}\frac{\log |V_n|}{\log \left( \frac{1}{\varepsilon _n}\right) }$.
- If $\varepsilon _n\ll \frac{1}{t_{\textrm{mix}}^{G_n}(\theta )}$ for some $\theta \in (0,1)$, then the random walk on $(G_n^*)$ exhibits cutoff if and only if the random walk on $(G_n)$ does.
- If $\varepsilon _n\asymp \frac{1}{t_{\textrm{mix}}^{G_n,\textrm{lazy}}\left( \frac{1}{4}\right) }$ and the lazy random walk on $(G_n)$ does not exhibit cutoff, then neither does the random walk on $(G_n^*)$.

#### Remark 1.2

We will refer to graphs satisfying the assumptions of Theorem 1 (e.g. expanders) as graphs having linear growth of entropy, while we refer to graphs satisfying the assumptions of Theorem 2 (e.g. tori) as graphs having polynomial growth of balls.

We say that a sequence of graphs $(G_n)$ is an expander family if $|V_n|\rightarrow \infty$ as $n\rightarrow \infty$, all vertices have degree $\le \Delta$ for some constant $\Delta$, and there exists a constant $a>0$ such that for any set $A_n\subseteq V_n$ with $|A_n|\le \frac{1}{2}|V_n|$ we have $|\partial A_n|\ge a|A_n|$, where $\partial A_n$ is the set of edges between $A_n$ and $V_n{\setminus } A_n$ in $G_n$.

In Sect. 5 we present a proof of the standard fact that expander graphs have a linear growth of entropy. In this case we get a phase transition for the occurrence of cutoff around the value $\varepsilon _n=\frac{1}{\log |V_n|}$ as the following result shows.

#### Theorem 3

Let $(\varepsilon _n)$ be a sequence of constants in (0, 1) and let $(G_n)$ be a family of connected expander graphs. Let $G_n^*$ be as in Definition 1.1.

- If $\varepsilon _n\gg \frac{1}{\log |V_n|}$ then the random walk on $(G_n^*)$ exhibits cutoff with high probability, at a time of order $\log |V_n|$.
- If $\varepsilon _n\ll \frac{1}{\log |V_n|}$ then the random walk on $(G_n^*)$ exhibits cutoff if and only if the random walk on $(G_n)$ does.
- If $\varepsilon _n\asymp \frac{1}{\log |V_n|}$ and the lazy random walk on $(G_n)$ does not exhibit cutoff, then neither does the random walk on $(G_n^*)$.
- There exists a sequence of expanders $(G_n)$ such that the lazy random walk on $(G_n)$ exhibits cutoff, and for any sequence $(\varepsilon _n)$ with $\varepsilon _n\asymp \frac{1}{\log |V_n|}$, the random walk on $(G_n^*)$ also exhibits cutoff whp.
- There exists a sequence of expanders $(G_n)$ such that the lazy random walk on $(G_n)$ exhibits cutoff, but for any sequence $(\varepsilon _n)$ with $\varepsilon _n\asymp \frac{1}{\log |V_n|}$, the random walk on $(G_n^*)$ does not exhibit cutoff whp.

#### Theorem 4

Let $\Delta$ be a given positive integer and let *p* be a given polynomial. Let $(\varepsilon _n)$ be a sequence of constants in (0, 1) and let $(G_n)$ be a sequence of connected graphs with the following properties: each $G_n$ is vertex-transitive, $|V_n|\rightarrow \infty$ as $n\rightarrow \infty$, all vertices have degree $\Delta$, and for any *r* the volume of any ball of radius *r* in any $G_n$ is upper bounded by *p*(*r*). Let $G_n^*$ be as in Definition 1.1.

- If $\varepsilon _n\gtrsim |V_n|^{-o(1)}$ then the random walk on $(G_n^*)$ exhibits cutoff with high probability, at a time of order $\frac{1}{\varepsilon _n}\frac{\log |V_n|}{\log \left( \frac{1}{\varepsilon _n}\right) }$.
- If $\varepsilon _n\lesssim |V_n|^{-a_n}$ where $a_n\asymp 1$, then whp the random walk on $(G_n^*)$ does not exhibit cutoff. If in addition we have $\varepsilon _n\gg \frac{1}{\textrm{diam}\left( G_n\right) ^2}$ where $\textrm{diam}\left( G_n\right)$ is the diameter of $G_n$, then whp the mixing time of $G_n^*$ satisfies $t_{\textrm{mix}}^{G_n^*}\left( \frac{1}{4}\right) \asymp \frac{1}{\varepsilon _n}$.

Moreover, the random walk on $(G_n)$ does not exhibit cutoff.

We also obtain a result for general families of graphs with bounded degrees, as follows.

#### Theorem 5

Let $\Delta$ be a given positive constant. Let $(\varepsilon _n)$ be a sequence of constants in (0, 1) and let $(G_n)$ be a sequence of connected graphs such that $|V_n|\rightarrow \infty$ as $n\rightarrow \infty$ and all vertices have degree $\le \Delta$. Let $G_n^*$ be as in Definition 1.1.

- If $\varepsilon _n\gg \frac{\log \log |V_n|}{\log |V_n|}$ then the random walk on $(G_n^*)$ exhibits cutoff with high probability.
- If $\varepsilon _n\ll \frac{1}{t_{\textrm{mix}}^{G_n}(\theta )}$ for some $\theta \in (0,1)$, then the random walk on $(G_n^*)$ exhibits cutoff if and only if the random walk on $(G_n)$ does.
- If $\varepsilon _n\asymp \frac{1}{t_{\textrm{mix}}^{G_n,\textrm{lazy}}\left( \frac{1}{4}\right) }$ and the lazy random walk on $(G_n)$ does not exhibit cutoff, then neither does the random walk on $(G_n^*)$.

#### Remark 1.3

We conjecture that the above condition in (a) can be improved and in fact the random walk on $(G_n^*)$ exhibits cutoff for any sequence $\varepsilon _n\gg \frac{1}{\log |V_n|}$. See Sect. 7.1 for further discussion.

#### Remark 1.4

In the appropriate cases of Theorems 1, 2, 3 and 4 we indicated the order of the mixing time. In the proofs we obtain an expression for the multiplicative factor in terms of limiting quantities (speed and entropy) for a random walk on an auxiliary graph.

### Relation to other works

In this work we establish cutoff for random walks on randomly generated graphs at an entropic time. There have been multiple recent works proving cutoff at an entropic time, including [4, 6, 8, 9, 11, 12, 15, 17, 18]. For a more detailed overview please refer to [18]. For some exciting recent progress which extends the connection between cutoff and entropic concentration to non-random graphs, see [23, 25, 26].

Our model is a generalisation of the model in [18], where $\varepsilon _n\equiv 1$. In both cases the graph $G^*$ locally looks like a tree-like structure, as we explain in the overview below. In the case when $\varepsilon _n$ is of constant order or goes to 0 sufficiently slowly, the proofs in [18] become more technical, but can be adapted to prove cutoff. To establish cutoff for the full range of $\varepsilon _n$ as in Theorems 1 and 2 we need to use a different method. (We explain in more detail why this is necessary in Sect. 1.3.)

Our approach is inspired by [5, 20] that establish cutoff for non-backtracking random walks. To approximate the time 2*t* transition probability between two vertices *x* and *y* of the random graph they consider reversing the second half of a length 2*t* path and study the first *t* steps of two independent walks from *x* and *y*. These in turn can be approximated by the first *t* steps of two independent walks on two independent copies of the limiting tree. As far as we are aware this method has only been used for non-backtracking walks at fixed times, which makes the reversal of the second half of the path straightforward and allows to get a good control over the position of the walk on the tree.

In our work we use this idea for a simple random walk, and instead of looking at a fixed time we use it for a random time $\tau$. This means that we face additional challenges regarding both the reversal of paths and the study of a walk on the limiting tree-like structure. We prove that at the random time $\tau$ the position of the walk is close to the uniform distribution on the vertices, which has bounded $\ell ^{\infty }$ distance from the stationary distribution, and we also prove that $\tau$ is concentrated around some given time. To conclude cutoff our approach relies crucially on the connection between cutoff and concentration of hitting times of large sets established in [2]. To the best of our knowledge, this is the first time the results of [2] have been utilised in this fashion. We believe this method and variants of the arguments in this paper can also be used to analyse the random walk on more involved random graph models.

In case $\varepsilon _n\ll \frac{1}{t_{\textrm{mix}}^{G_n}}$ it is intuitively clear that the walk on $G^*_n$ has cutoff if and only if the walk on $(G_n)$ does, and we formalise this intuition in Proposition 6.5. In Theorem 4 (b) there is a regime with $\varepsilon _n\gtrsim \frac{1}{t_{\textrm{mix}}^{G_n}}$ but no cutoff and the proof of this is quite demanding. Here we need to use a different argument relying on reversing the second half of a path and considering walks on independent tree-like structures.

### Overview

Below we give an overview of the methods we use to establish an upper bound on the mixing time in the cutoff regime, which is the most difficult part of the proof.

In many commonly used random graph models (e.g. in Erdős-Rényi graphs $G_n\sim \mathcal {G}\left( n,\frac{d}{n}\right)$) the graph can be approximated locally with a Galton-Watson tree. In our case the situation is not this simple; the graph $G^*$ also retains some of the original structure of *G*, so it does not quite look like a tree, but it can still be approximated locally with a tree-like structure.

Similarly to [18] we define the random *quasi-tree* *T* corresponding to a graph *G*, radius *R* and weight $\varepsilon$ as follows. We consider a ball *B* of radius *R* around a uniformly chosen vertex $\rho$ of *G*. For each vertex *v* of the ball, except for $\rho$, we draw a new edge from *v* and attach an independently sampled copy of *B* to the other end. We repeat this for all vertices of the newly added balls, except their centres. Then proceed similarly, resulting in an infinite graph. We call the edges joining different balls of *T* *long-range edges* and we assign weight $\varepsilon$ to them. We call $\rho$ the root of *T*. We sometimes refer to the balls in *T* as *R*-balls. We will usually work with the ‘long-range distance’ on *T*. (For vertices *x* and *y* this is defined as the minimum number of long-range edges a path from *x* to *y* has to cross.)

**Fig. 1:**
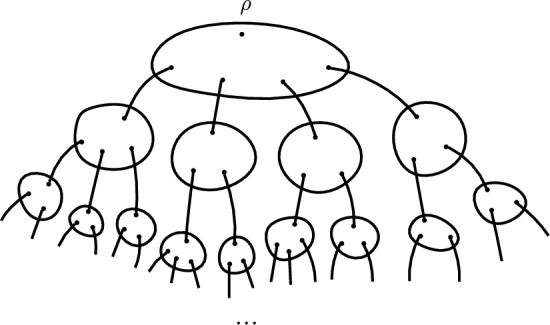
An illustration of a quasi-tree

Note that the offsprings of each ball in *T* are independent and identically distributed (iid), which will make it easier to study the behaviour of a random walk $\widetilde{X}$ on *T* than studying a random walk *X* on $G^*$ directly. We will show that locally $G^*$ and a random walk *X* on it can be well approximated by a quasi-tree *T* and a random walk $\widetilde{X}$ on it from its root.

In [18] the proof proceeds as follows. The first step is to obtain a concentration result on the speed and entropy of a random walk $\widetilde{X}$ on the quasi-tree *T*. After this a coupling is defined between the random graph $G^*$ and the walk *X* on it, and the random quasi-tree *T* and the walk $\widetilde{X}$. Using these it is then proved that at the entropic time, which is of order $\log |V_n|$, the $L_2$-distance between the distribution of the walk *X* conditioned on a certain typical event and the invariant distribution $\pi _{G^*}$ is bounded by $\exp \left( c\sqrt{\log |V_n|}\right)$ for a positive constant *c*. After that the proof of the upper bound on the mixing time can be concluded by showing that the absolute relaxation time is of constant order and using the Poincaré inequality.

In our model we are also able to establish concentration results for the speed and entropy of the walk on the quasi-tree (see Lemma 2.19 and Proposition 2.20). The bounds on the fluctuations of speed and entropy and the upper bound on the absolute relaxation time are all functions of $\varepsilon$, so the above approach would only work for weights $\varepsilon$ approaching 0 sufficiently slowly. To prove cutoff for $\varepsilon$ decaying faster, we need a different approach.

In particular, in order for the method of [18] to work one requires an entropic concentration estimate, which is analogous to the varentropy condition of [25, Theorem 5]. More precisely, we consider a random variable whose mean is the entropy of the law of the random walk on the quasi-tree at the entropic time. We need to show that the standard deviation of this random variable is $o\left( \frac{t_{\textrm{mix}}}{t_{\textrm{rel}}}\right)$. In case of some graphs covered by Theorems 1 and 2 the deviations of the entropy are too large. (For Theorem 1 the examples are graphs with Cheeger constant $O\left( \varepsilon \right)$, for example a lamplighter graph on a $d\ge 3$ dimensional torus or a locally expanding graph in the sense of Proposition 5.3 with a bottleneck. In this case one can show that $t_{\textrm{rel}}(G^*)\asymp \frac{1}{\varepsilon }$. For Theorem 2 one can consider two graphs of comparable sizes with polynomial growth of balls whose ratio of entropy is bounded away from 1 (e.g. two tori of different dimensions), connected by relatively few edges.) For the graphs from Theorem 4 one can prove stronger entropic concentration estimates which satisfy the above varentropy condition, however because in Theorem 4 we consider very small values of $\varepsilon$, an additional technical difficulty arises. The argument in [18] involves a certain exploration process in which a portion of the random graph is coupled with a quasi-tree. When $\varepsilon$ is sufficiently small, a substantial challenge that arises is that it becomes difficult to control the size of the revealed graph and ensure it is much smaller than *n*, which is required for the coupling to work.

We now describe a different approach, where we only have to reveal slightly more than $\sqrt{|V_n|}$ vertices, regardless of the value of $\varepsilon$.

In order to upper bound the mixing time, we define a random time $\tau$ and show that wherever the walk *X* starts from, (i) $\tau$ is likely to be $\le t$ for a given *t* that agrees with our lower bound on the $1-o(1)$ mixing time up to lower order terms, and (ii) the distribution of $X_{\tau }$ is close to the uniform distribution $\mathcal {U}$ on the set *V* of vertices. This implies that for any fixed small $\theta \in \left( 0,\frac{1}{4}\right)$, if *n* is sufficiently large, then any set $A\in V$ with $\pi (A)\ge 1-\theta$ is hit by time *t* with probability at least $1-\theta$. Using a result from [2] relating mixing and hitting times, together with an upper bound of order $\frac{1}{\varepsilon }$ on the absolute relaxation time of the walk on $G^*$, we get that $t_{\textrm{mix}}^{G^*}(2\theta )\le t+\frac{C(\theta )}{\varepsilon }$.

Assuming the walk *X* starts from a vertex *x* with a sufficiently nice neighbourhood in $G^*$, the random time we consider is $\tau _{2L}$, which is roughly speaking the first time when *X* has travelled long-range distance 2*L* from *x*, for an appropriate choice of *L*.^1^ To upper bound

$$\begin{aligned}d_{\textrm{TV}}\left( {\mathbb {P}_{x}\!\Big (X_{\tau _{2L}}=\cdot \;\Big \vert \;G^*\Big )},{\mathcal {U}(\cdot )}\right) =\sum _{y}\left( \frac{1}{n}-\mathbb {P}_{x}\!\Big (X_{\tau _{2L}}=y\;\Big \vert \;G^*\Big )\right) ^+,\end{aligned}$$

it is sufficient to lower bound $\mathbb {P}_{x}\!\Big (X_{\tau _{2L}}=y\;\Big \vert \;G^*\Big )$ for most values of *y*.

We would like to be able to express $\mathbb {P}_{x}\!\Big (X_{\tau _{2L}}=y\;\Big \vert \;G^*\Big )$ as

1.2 $$\begin{aligned} \mathbb {P}_{x}\!\Big (X_{\tau _{2L}}=y\;\Big \vert \;G^*\Big )\approx \sum _{w,z}\mathbb {P}_{x}\!\Big (X_{{\tau }^{(X)}_{L}}=z\;\Big \vert \;G^*\Big )\mathbb {P}_{\eta (y)}\!\Big (Y_{{\tau }^{(Y)}_{L}}=w\;\Big \vert \;G^*\Big ){{{\mathfrak {1}}}}_{w=\eta (z)}, \end{aligned}$$

where *X* and *Y* are independent random walks on $G^*$ from *x* and *y* respectively, ${\tau }^{(X)}_{L}$ and ${\tau }^{(Y)}_{L}$ are the first times when *X* and *Y* respectively, have travelled long-range distance *L*, and $\eta (v)$ denotes the long-range neighbour of vertex *v*. A decomposition similar to (1.2) was considered in [3, 5], but there it was applied for a non-backtracking random walk and deterministic times 2*t* and *t* instead of $\tau _{2L}$ and $\tau _L$, hence it could be written as an exact equality.

Now we explain how to obtain the approximate equality in (1.2) and how this decomposition can be used to lower bound $\mathbb {P}_{x}\!\Big (X_{\tau _{2L}}=y\;\Big \vert \;G^*\Big )$.

#### Approximate reversibility at a random time

**Fig. 2:**
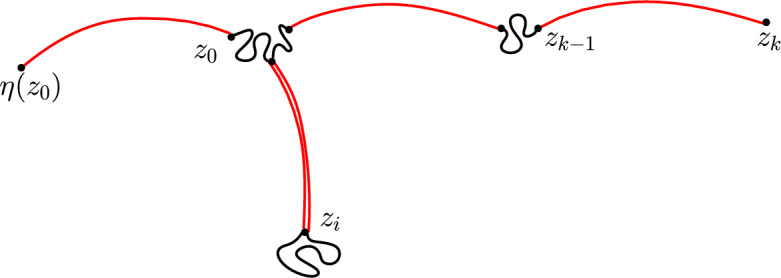
In this example the path $(z_0,...,z_k)$ starts by crossing some edges of *G* (pictured in black), then crosses a long-range edge (pictured in red), then crosses some more edges of *G* and backtracks the previous long-range edge, then crosses some edges of *G* and crosses a new long-range edge, then does this one more time. The path $(z_0,...,z_k)$ first reaches long-range distance 2 from $z_0$ at $z_k$, but the reverse path $(z_{k-1},...,z_0,\eta (z_0))$ already reaches long-range distance 2 from $z_{k-1}$ at $z_i$ (color figure online)

**Fig. 3:**
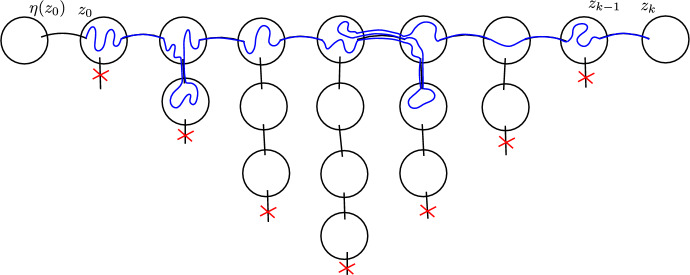
The blue path $z=(z_0,...,z_k)$ satisfies the condition $\Omega _1^z(7)$. If we instead considered a path that started like *z*, left the pictured region via a long-range edge marked with red, then returned and continued like *z*, it would fail the condition (color figure online)

The reason we do not have an exact equality in (1.2) is that for a path $(z_0,...,z_k)$ the event that *k* is the first time when the path reached long-range distance *L* is not equivalent to the event that *k* is the first time that the path $(z_{k-1},...,z_0,\eta (z_0))$ reached long-range distance *L*. See a counterexample in Fig. 2. For a path $z=(z_0,...,z_k)$ we define an event $\Omega ^{z}_1(L)$ which is equivalent for the path and its reversal and on this event *k* being the first time when *z* reaches long-range distance *L* is equivalent to *k* being the first time that $(z_{k-1},...,z_0,\eta (z_0))$ reaches long-range distance *L*. See an illustration in Fig. 3. Using that the walks only rarely cross the same long-range edge multiple times, we will show that for any $G^*$ the event $\Omega ^X_1(L)$ holds whp.

#### Lower bounding $\mathbb {P}_{x}\!\Big (X_{\tau _{2L}}=y\;\Big \vert \;G^*\Big )$ via (1.2)

We couple certain random neighbourhoods of *x* and *y* in $G^*$ and the walks *X* and *Y* on them by independent random quasi-trees $T_x$ and $T_y$ and independent random walks $\widetilde{X}$ and $\widetilde{Y}$ on them. We then define subsets $\partial T_x$ and $\partial T_y$ of the vertices of $T_x$ and $T_y$, respectively so that the long-range edges from these vertices are not yet revealed, for each pair $z\in \partial T_x$, $w\in \partial T_y$ the probability $\mathbb {P}_{x}\!\Big (X_{{\tau }^{(X)}_{L}}=\eta (z)\;\Big \vert \;G^*\Big )\mathbb {P}_{\eta (y)}\!\Big (Y_{{\tau }^{(Y)}_{L}}=\eta (w)\;\Big \vert \;G^*\Big )$ is sufficiently small, but the overall probability $\sum _{z\in \partial T_x,w\in \partial T_y}\mathbb {P}_{x}\!\Big (X_{{\tau }^{(X)}_{L}}=\eta (z)\;\Big \vert \;G^*\Big )\mathbb {P}_{\eta (y)}\!\Big (Y_{{\tau }^{(Y)}_{L}}=\eta (w)\;\Big \vert \;G^*\Big )$ is close to 1. (The random quasi-trees $T_x$ and $T_y$ are infinite, but we will only be concerned with the random finite regions where they agree with the corresponding neighbourhoods in $G^*$. The sets $\partial T_x$ and $\partial T_y$ will be the boundaries of these regions.)

Conditioning on the explorations of the neighbourhoods of *x* and *y*, we can complete the exploration of $G^*$ by choosing a uniform random matching of the yet unmatched long-range half-edges.^2^ We then use the following concentration result from [5] (based on [10]) to lower bound (1.2).

##### Lemma 1.5

(Lemma 5.1 in [5]) Let $\mathcal {I}$ be a set of even size. Let $(w_{i,j})_{(i,j)\in \mathcal {I}\times \mathcal {I}}$ be some non-negative weights and let $\eta$ be a uniform random pairing of $\mathcal {I}$. Then for all $a>0$ we have

$$\begin{aligned}\mathbb {P}\!\left( \sum _{i\in \mathcal {I}}w_{i,\eta (i)}<m-a\right) \le \exp \left( -\frac{a^2}{4bm}\right) \end{aligned}$$

where $$m=\frac{1}{|\mathcal {I}|-1}\sum _{i\in \mathcal {I}}\sum _{j\in \mathcal {I}\setminus \{i\}}w_{i,j}$$ and $$b=\max _{i\ne j}(w_{i,j}+w_{j,i})$$.

We let $w_{i,j}=\mathbb {P}_{x}\!\Big (X_{{\tau }^{(X)}_{L}}=\eta (i)\;\Big \vert \;G^*\Big )\mathbb {P}_{\eta (y)}\!\Big (Y_{{\tau }^{(Y)}_{L}}=\eta (j)\;\Big \vert \;G^*\Big ){{{\mathfrak {1}}}}_{i\in \partial T_x,j\in \partial T_y}$ and $a=\frac{1}{2}m$. Having defined $T_x$, $\partial T_x$,... appropriately, this will give us that with probability $1-o\left( \frac{1}{|V_n|^2}\right)$ the graph $G^*$ is such that for most pairs of vertices *x*, *y* (for all pairs satisfying a certain condition) the sum (1.2) is lower bounded by $\frac{1}{|V_n|}(1-\theta )$, where $\theta$ is an arbitrarily small constant.

Note that the probability $1-o\left( \frac{1}{|V_n|^2}\right)$ in the above result is high enough so that we can take a union bound over all pairs *x*, *y* satisfying the required condition and still get a high probability statement.

### Organisation

In Sect. 2 we define the notion of a random quasi-tree *T* and prove concentration results on the speed and entropy of a random walk on *T*. In this section we work under minimal assumptions on *G* and $\varepsilon$.

In Sect. 3 we introduce additional assumptions. We define an exploration process of $G^*$ and a coupling of *X* on $G^*$ to $\widetilde{X}$ on *T*. In this section we prove that the coupling succeeds with a large probability.

In Sect. 4 we still work under the assumptions of Sect. 3. Here we prove the upper and lower bounds on the mixing time.

In Sect. 5 we prove that sequences of graphs with polynomial growth of balls or linear growth of entropy satisfy the assumptions of Sect. 3, hence they have cutoff in the regimes $\varepsilon _n=|V_n|^{-o(1)}=o(1)$ and $\frac{1}{\log |V_n|}\ll \varepsilon _n\ll 1$ respectively. We also show that expanders and locally expanding graphs (to be defined later) have linear growth of entropy.

In Sect. 6 we discuss the other regimes for $\varepsilon _n$ and in case of expanders and vertex-transitive graphs with polynomial growth of balls we complete the picture for every possible sequence $\varepsilon _n$.

In Sect. 7 we discuss some conjectures regarding more general families of graphs and present a sketch of the proof of Theorem 5.

Appendix A lists a few standard results that are used in various proofs. Some of the proofs about speed and entropy that are analogous to the corresponding proofs in [18] and some further miscellaneous proofs are deferred to supplementary material [1].

## Limiting tree

In what follows we will work with $(G_n)$, $(\varepsilon _n)$ and $(G^*_n)$ as in Definition 1.1 and in addition we also assume that all vertices of all graphs have degree $\le \Delta$ (where $\Delta$ is a given constant) and $\varepsilon _n\rightarrow 0$ as $n\rightarrow \infty$. For ease of notation we will also assume that $|V_n|=n$.

In this section we start by defining a tree-like structure that will be used to approximate the graph $G_n^*$. It is the same as the definition of a *quasi-tree* in [18] except that the weights of the long-range edges in our setting are equal to $\varepsilon _n$. The goal is then to study the entropy of a random walk on these weighted *quasi-trees*.

### Definition 2.1

Given a graph *G* and constants $\varepsilon >0$ and $R\in \mathbb {Z}_{\ge 1}$ we define the associated *quasi-tree* $T=T_{G,R,\varepsilon }$, which is a random weighted infinite graph, as follows.

Let *B* be a random ball (in the graph distance of *G*) obtained by first sampling a uniform vertex and then considering its *R*-neighbourhood. We call such a ball a *T*-*R*-ball, or *R*-ball.

Let $\rho$ be the centre of *B* that we will call the root of *T*. Next join by an edge each other vertex of *B* (except for the root) to the centre of an i.i.d. copy of *B*. Repeat the same procedure for every vertex of the new balls except for their centres. We call edges joining different balls *long-range edges* and we assign weight $\varepsilon$ to them, while we assign weight 1 to all other edges.

Let $\mathcal {T}$ be the topological space consisting of all rooted finite, or infinite but locally finite unlabelled connected graphs with a collection of distinguished edges, called *long-range edges*, with the property that every simple path between a pair of vertices must cross the same collection of long-range edges. (In other words, the long-range edges give rise to a tree structure.) We call a member *T* of $\mathcal {T}$ a *quasi-tree* and we call the connected components of *T* without the long-range edges the *T*-balls of *T*. The graph $T_{G,R,\varepsilon }$ described above is a random variable taking values in this topological space $\mathcal {T}$ and its *T*-*R*-balls are exactly the *T*-balls in the above sense.

For a quasi-tree *T* and $x,y \in T$ we write $d_T(x,y)$ (or simply *d*(*x*, *y*) when *T* is clear from the context), for the number of long-range edges on the shortest path from *x* to *y*. Note that this is not the usual graph distance on *T*, but for us this will be a useful notion of distance. One can think of this distance as “the long-range distance”, but since we rarely consider the graph distance on *T*, we do not use this terminology. A level of *T* consists of all vertices at the same distance from $\rho$, i.e. when $d(\rho ,x)=r$, then *x* belongs to the *r*-th level. We denote the level of a vertex *x* by $\ell (x)$. We write $\mathcal {B}_r(x)=\mathcal {B}(x,r)=\{y:d_T(x,y) \le r \}$ for the ball of radius *r* centred at *x*.

### Definition 2.2

In the graph $G^*$ we define the (long-range) distance between *x* and *y* to be the minimal number of long-range edges needed to be crossed to go from *x* to *y*, when we only allow at most *R* consecutive edges of *G* in the path from *x* to *y* and we do not allow any long-range edge to be crossed more than once. (Like for the quasi-tree, we rarely use the regular graph distance on $G^*$, so unless specified otherwise the term “distance” will refer to the aforementioned distance.)

We write $\mathcal {B}_K^*(x)$ for the ball of radius *K* and centre *x* in this metric. We write $\mathcal {B}_{G}(x,r)$ for a ball centred at *x* of radius *r* in the graph metric of *G*. When $r=R$, we call it the $G^*$-*R*-ball centred at *x*.

Below we establish a few more notations and conventions.

Let *X* be a random walk on the weighted quasi-tree *T*. For a (directed) edge (*x*, *y*) we write

$$\begin{aligned} \tau ^+_{(x,y)} = \inf \{t\ge 1: (X_{t-1},X_t)= (x,y)\}, \end{aligned}$$

i.e. $\tau ^+_{(x,y)}$ is the first time that *X* crosses the edge (*x*, *y*).

Similarly we let $\tau _x$ be the first time when *X* hits vertex *x* and $\tau _k$ be the first time when *X* reaches (long-range) distance *k*.

For a long-range edge *e* we will often write $e=(e^-,e^+)$ where $e^-$ is the endpoint of *e* closer to the root (or other reference vertex made clear from the context) and $e^+$ is the other endpoint. We define $\ell (e):=\ell (e^+)$. For a vertex *x* we will denote its long-range neighbour by $x^+$ if it is further from the root (or reference vertex) than *x*, and denote by $x^-$ if it is closer.

In what follows most statements will concern sequences of graphs or sequences of quasi-trees. To simplify notation, we will often drop the super-/subscripts *n*. Some statements are about random quasi-trees associated to a sequence of graphs. In this case we assume that the graphs and the sequence of weights satisfy the assumptions in Definition 1.1, the graphs $G_n$ have bounded degrees, $\varepsilon _n\rightarrow 0$, and that $R_n\gtrsim \frac{1}{\varepsilon _n}$. Some statements are about sequences of non-random quasi-trees from the topological space $\mathcal {T}$. In this case (unless specified otherwise) we assume that the sequence is a possible realisation of random quasi-trees associated to a sequence of graphs as above.

Later we will talk about the boundary of an *R*-ball. By that (with some abuse of notation) we mean the vertices in the *R*-ball that are at graph distance *R* from its centre.

### Definition 2.3

Given a random walk *X* on a transient quasi-tree *T* let us define its loop-erasure $\xi$ as follows. Let $\xi _k$ be the long-range edge between levels $k-1$ and *k* that was last visited by *X*, which exists almost surely by transience. Note that this is equivalent to erasing the loops of *X* in the chronological order in which they were created and then only keeping the long-range edges. We say that a random sequence $\xi$ of long-range edges is a loop-erased random walk (LERW) on *T* if it has the same distribution as the loop-erasure of a random walk on *T*.

### Some preliminary bounds

In this section we bound the probability of a walk on a quasi-tree crossing any specific long-range edge, and then we use this to derive various bounds that will be used throughout the paper.

#### Lemma 2.4

Let $C>0$ and $\Delta \in \mathbb {Z}_{\ge 1}$. Let $(T^{(n)})$ be a sequence of weighted quasi-trees and suppose that the weights $\varepsilon _n$ converge to 0 as $n\rightarrow \infty$, $R_n\ge \frac{C}{\varepsilon _n}$ for all *n* and each vertex in each tree has degree $\le \Delta$ (not counting the long-range edges). Let $X^{(n)}$ be a random walk on $T^{(n)}$ starting from its root and let

$$\begin{aligned} \delta _n:=1-\inf _{(x_n,y_n)}\mathbb {P}_{y_n}\!\left( \tau _{(y_n,x_n)}^+=\infty \right) , \end{aligned}$$

where the infimum is taken over all long-range edges $(x_n,y_n)$ of $T^{(n)}$ (with $x_n$ being closer to the root than $y_n$).

Then there exist positive constants $c_1$, $c_2$ depending only on $\Delta$ such that

$$\begin{aligned} c_1\varepsilon _n\le \delta _n\le c_2\varepsilon _n^{1/3}. \end{aligned}$$

In particular, $\delta _n\rightarrow 0$ as $n\rightarrow \infty$.

The lemma above immediately implies the following.

#### Corollary 2.5

In the setup of Lemma 2.4 for any $z_n$ in the $R_n$-ball of $y_n$ we have

$$\begin{aligned} \mathbb {P}_{z_n}\!\left( \tau _{(y_n,x_n)}^+<\infty \right) \le \delta _n. \end{aligned}$$

#### Proof of Lemma 2.4

For ease of notation we drop the subscript *n*.

We fix a long-range edge (*x*, *y*). We immediately get that $\mathbb {P}_{y}\!\left( \tau _{(y,x)}^+<\infty \right) \ge \mathbb {P}_{y}\!\left( X_1=x\right) \ge \frac{\varepsilon }{\Delta +\varepsilon }$, showing the lower bound on $\delta$.

Let *N* be the first time when *X* leaves the *T*-*R*-ball of *y*. We will show that

2.1 $$\begin{aligned} \mathbb {P}_{y}\!\left( X_N=x\right) \le c\varepsilon ^{1/3} \end{aligned}$$

as $n\rightarrow \infty$ for some constant *c* depending only on $\Delta$. Once we have that we can establish the upper bound on $\delta$ as follows. Let *Y* be the random walk on the directed long-range edges induced by *X*. Then $d(\rho ,Y)$ stochastically dominates a biased random walk on $\mathbb {Z}_{\ge 0}$ that steps to the right with probability $1-c\varepsilon ^{1/3}$ and to the left with probability $c\varepsilon ^{1/3}$. The probability of this biased walk returning to the starting point is $\asymp \varepsilon ^{1/3}$ as $n\rightarrow \infty$.

We will now prove (2.1). Note that

$$\begin{aligned} \mathbb {P}_{y}\!\left( X_N=x\right) \le \mathbb {P}_{y}\!\left( N\le \frac{1}{\varepsilon ^{2/3}}\right) +\sum _{i\ge \frac{1}{\varepsilon ^{2/3}}}\mathbb {P}_{y}\!\left( N=i,X_i=x\right) \qquad \text {and}\\ \mathbb {P}_{y}\!\left( N=i,X_i=x\right) =\sum _{(x_0,...,x_{i-1})}\mathbb {P}_{y}\!\left( X_1=x_1,...,X_{i-1}=x_{i-1}\right) \mathbb {P}_{y}\!\left( X_1=x\right) \end{aligned}$$

where the sum is taken over all paths $(x_0,...,x_{i-1})$ inside the *T*-*R*-ball of *y* with $x_0=x_{i-1}=y$. This sum is equal to

$$\begin{aligned} \sum _{(x_0,...,x_{i-1})}\left( \prod _{j=0}^{i-2}\frac{1}{\deg (x_j)+\varepsilon }\right) \frac{\varepsilon }{\deg (y)+\varepsilon }, \end{aligned}$$

where the long-range edges are not counted towards $\deg$. Let *Z* be a simple random walk on the *T*-*R*-ball of *y*. The last expression above then equals

$$\begin{aligned}&\sum _{(x_0,...,x_{i-1})}\left( \prod _{j=0}^{i-2}\frac{1}{\deg (x_j)}\right) \left( \prod _{j=0}^{i-2}\frac{\deg (x_j)}{\deg (x_j)+\varepsilon }\right) \frac{\varepsilon }{\deg (y)+\varepsilon }\\&\quad =\sum _{(x_0,...,x_{i-1})}\mathbb {P}_{y}\!\left( Z_1=x_1,...,Z_{i-1}=x_{i-1}\right) \left( \prod _{j=0}^{i-2}\frac{\deg (x_j)}{\deg (x_j)+\varepsilon }\right) \frac{\varepsilon }{\deg (y)+\varepsilon }\\&\quad \le \mathbb {P}_{y}\!\left( Z_{i-1}=y\right) \left( \frac{\Delta }{\Delta +\varepsilon }\right) ^{i-1}\frac{\varepsilon }{1+\varepsilon }. \end{aligned}$$

Since *Z* is a simple random walk on a graph with $\ge R$ vertices and degrees bounded by $\Delta$, from Lemma A.2 we know that for every *j* we have $\mathbb {P}_{y}\!\left( Z_j=y\right) \lesssim \frac{1}{\sqrt{j}\wedge R}$.

Since at every step the probability of *X* leaving the *T*-*R*-ball of *y* is $\ge \frac{\varepsilon }{\Delta +\varepsilon }$, we also have by a union bound

$$\begin{aligned} \mathbb {P}_{y}\!\left( N\le \frac{1}{\varepsilon ^{2/3}}\right) \le \frac{1}{\varepsilon ^{2/3}}\frac{\varepsilon }{\Delta +\varepsilon }\le \varepsilon ^{1/3}. \end{aligned}$$

Together these give

$$\begin{aligned} \mathbb {P}_{y}\!\left( X_N=x\right)&\lesssim \varepsilon ^{1/3} + \sum _{i=\frac{1}{\varepsilon ^{2/3}}}^{R^2}\frac{1}{\sqrt{i-1}}\left( \frac{\Delta }{\Delta +\varepsilon }\right) ^{i-1}\frac{\varepsilon }{1+\varepsilon }\\&\quad + \sum _{i\ge R^2}\frac{1}{R}\left( \frac{\Delta }{\Delta +\varepsilon }\right) ^{i-1}\frac{\varepsilon }{1+\varepsilon }\\&\lesssim \varepsilon ^{1/3}+\varepsilon ^{1/3}\frac{\Delta +\varepsilon }{1+\varepsilon }+\frac{1}{R}\frac{\Delta +\varepsilon }{1+\varepsilon }\lesssim \varepsilon ^{1/3}+\frac{1}{R} \asymp \varepsilon ^{1/3}. \end{aligned}$$

(Note that $\frac{1}{R}\lesssim \varepsilon \ll \varepsilon ^{1/3}$, so the constant in the last $\asymp$ does not depend on $\inf _n\{R_n\varepsilon _n\}$.) $\square$

#### Definition 2.6

For a walk *X* on a quasi-tree *T* let us say that *t* is a regeneration time of *X* if $(X_{t-1},X_{t})$ is a long-range edge and is crossed by the walk exactly once. For a walk *X* on *T* and a given non-negative integer *K* let us define the sequence $(\sigma _i)_{i\ge 0}$ as follows. Let $\sigma _0$ be the first time when *X* reaches the *K*th level, and for each $i\ge 1$ let $\sigma _i$ be the *i*th regeneration time that happens at a level $\ge K+1$. If *K* is not specified, we take $K=0$. Let $\varphi _i=d_T(\rho ,X_{\sigma _i})$. (Recall that we are working with long-range distances.)

The following two statements will allow us to relate the walk at the regeneration times to the loop-erased random walk.

#### Lemma 2.7

Let $(T^{(n)})$ be a sequence of weighted quasi-trees as in Lemma 2.4, let $\rho _n$ be the root of $T^{(n)}$ and let $X^{(n)}$ be a random walk on $T^{(n)}$. Let $\sigma _1^{(n)}$ be the first regeneration time of $X^{(n)}$. Let $\xi ^{(n)}$ be a loop-erased random walk on $T^{(n)}$ and let $(x_n,y_n)$ be a long-range edge of $T^{(n)}$ in the first level. Then we have

$$\begin{aligned} \mathbb {P}_{\rho _n}\!\left( \left( X^{(n)}_{\sigma _1^{(n)}-1},X^{(n)}_{\sigma _1^{(n)}}\right) =(x_n,y_n)\right) = (1-o(1))\cdot \mathbb {P}_{\rho _n}\!\left( \xi ^{(n)}_1=(x_n,y_n)\right) , \end{aligned}$$

uniformly in $$(x_n,y_n)$$.

#### Proof

Let (*x*, *y*) be a long-range edge. We have

$$\begin{aligned}&\mathbb {P}_{\rho }\!\left( \xi _1=(x,y)\right) =\mathbb {P}_{\rho }\!\left( \tau _y<\infty \right) \sum _{k=0}^{\infty }\mathbb {P}_{y}\!\left( \tau _x<\infty \right) ^k\mathbb {P}_{x}\!\left( \tau _y<\infty \right) ^k\mathbb {P}_{y}\!\left( \tau _x=\infty \right) ,\\&\mathbb {P}_{\rho }\!\left( \left( X_{\sigma _1-1},X_{\sigma _1}\right) =(x,y)\right) =\mathbb {P}_{\rho }\!\left( \tau _y<\infty \right) \mathbb {P}_{y}\!\left( \tau _x=\infty \right) . \end{aligned}$$

Lemma 2.4 gives $\mathbb {P}_{y}\!\left( \tau _x<\infty \right) =o(1)$, hence $\sum _{k=0}^{\infty }\mathbb {P}_{y}\!\left( \tau _x<\infty \right) ^k\mathbb {P}_{x}\!\left( \tau _y<\infty \right) ^k=1+o(1)$. $\square$

#### Lemma 2.8

Let *T* be a quasi-tree as in Definition 2.1 with root $\rho$, let *X* be a random walk on *T* from $\rho$ and let $\xi$ be a loop-erased random walk on *T* from $\rho$. Let *K* be any non-negative integer and let $(\sigma _i)_{i\ge 0}$ be as in Definition 2.6. Then for any $r\ge 1$ and any vertex *y* that is the centre of an *R*-ball at (long-range) distance $K+r$ from $\rho$ we have

$$\begin{aligned} \mathbb {P}_{\rho }\!\left( X_{\sigma _1}=y\right) \le (2\delta ^2)^{r-1}\mathbb {P}_{\rho }\!\left( \tau _y<\infty \right) \le (2\delta ^2)^{r-1}\frac{1}{1-\delta }\mathbb {P}_{\rho }\!\left( \xi _{K+r}=(x,y)\right) , \end{aligned}$$

where *x* is the long-range neighbour of *y* and $\delta$ is defined as in Lemma 2.4.

#### Proof

Let *z* be the level *K* centre of the *R*-ball that is the ancestor of *x* and *y*. (Note that in case $K=0$ we have $z=\rho$.) Then

$$\begin{aligned} \mathbb {P}_{\rho }\!\left( X_{\sigma _1}=y\right)&=\mathbb {P}_{\rho }\!\left( \tau _z<\infty \right) \mathbb {P}_{\rho }\!\Big (X_{\sigma _1}=y\;\Big \vert \;\tau _z<\infty \Big )\qquad \text {and} \\ \mathbb {P}_{\rho }\!\left( \tau _y<\infty \right)&=\mathbb {P}_{\rho }\!\left( \tau _z<\infty \right) \mathbb {P}_{z}\!\left( \tau _y<\infty \right) . \end{aligned}$$

Let $x_0=\eta (z)$ be the long-range neighbour of *z*, let $y_0=z$, and let $(x_i,y_i)_{i=1}^r$ be the directed long-range edges between *z* and *y*. Let *Y* be the walk on the long-range edges induced by *X*. Then

$$\begin{aligned}&\mathbb {P}_{\rho }\!\Big (X_{\sigma _1}=y\;\Big \vert \;\tau _z<\infty \Big )\\&\quad =\mathbb {P}_{z}\!\left( Y\text { crosses each }(x_i,y_i)_{i=1}^{r-1}\text { at least 3 times, then crosses }(x_r,y_r) \text { for the first and last time}\right) \\&\quad \le \mathbb {P}_{z}\!\left( Y\text { crosses each }(x_i,y_i)_{i=1}^{r-1}\text { at least 3 times, then crosses }(x_r,y_r)\text { for the first time}\right) . \end{aligned}$$

For a path *p* of *Y* starting from $(x_0,y_0)$, ending at $(x_r,y_r)$ and crossing each of $(x_i,y_i)_{i=1}^{r-1}$ at least three times, let us associate a path $\widetilde{p}$ as follows. Let $(x_{j_1},y_{j_1})$ be the furthest edge *p* reaches before crossing $(y_1,x_1)$ again. Then let $\widetilde{p}$ start as $(x_1,y_1),...,(x_{j_1},y_{j_1}),(y_{j_1},x_{j_1}),...,(y_1,x_1),$ $(x_1,y_1),...,(x_{j_1},y_{j_1})$. Note that *p* must also cross these edges in this order before first crossing $(x_{j_1+1},y_{j_1+1})$. Then in each step let $(x_{j_{k+1}},y_{j_{k+1}})$ be the furthest edge *p* reaches before crossing $(y_{j_k+1},x_{j_k+1})$ again. Then append $(x_{j_k+1},y_{j_k+1}),...,(x_{j_{k+1}},y_{j_{k+1}}),$ $(y_{j_{k+1}},x_{j_{k+1}}),...,(y_{j_k+1},x_{j_k+1}),(x_{j_k+1},y_{j_k+1}),...,(x_{j_{k+1}},y_{j_{k+1}})$ to the end of $\widetilde{p}$. Continue this until $\tilde{p}$ reaches $(x_{r-1},y_{r-1})$. Then finally append $(x_r,y_r)$ to it. Note that $\widetilde{p}$ is a subsequence of *p*. Also note that $\widetilde{p}$ is always of length $(3r-2)$, can take $2^{r-1}$ different possible values (we can choose $(j_k)$ to be any subsequence of $(1,2,...,r-1)$) and that for each $\widetilde{p}$ the sum of the probabilities of the paths *p* associated to it is upper bounded by

$$\begin{aligned}  &   \prod _{i=1}^{3r-2}\mathbb {P}_{\widetilde{p}_{i-1}}\!\left( \tau _{\widetilde{p}_{i}}<\infty \right) \le \left( \prod _{i=1}^r\mathbb {P}_{(x_{i-1},y_{i-1})}\!\left( \tau _{(x_i,y_i)}<\infty \right) \right) \delta ^{2r-2}\\  &   \quad =\delta ^{2r-2}\mathbb {P}_{z}\!\left( \tau _{(x_r,y_r)}<\infty \right) , \end{aligned}$$

where $\widetilde{p}_i$ is the *i*th edge in $\widetilde{p}$. Summing over all $\widetilde{p}$ gives

$$\begin{aligned}\mathbb {P}_{\rho }\!\Big (X_{\sigma _1}=y\;\Big \vert \;\tau _z<\infty \Big )\le 2^{r-1}\delta ^{2r-2}\mathbb {P}_{z}\!\left( \tau _y<\infty \right) .\end{aligned}$$

We also know that

$$\begin{aligned} \mathbb {P}_{\rho }\!\left( \xi _{K+r}=(x,y)\right) \ge \mathbb {P}_{\rho }\!\left( \tau _y<\infty \right) \mathbb {P}_{y}\!\left( \tau _x=\infty \right) \ge (1-\delta )\mathbb {P}_{\rho }\!\left( \tau _y<\infty \right) . \end{aligned}$$

This finishes the proof. $\square$

Summing over all *y* at level $K+r$ in Lemma 2.8 we immediately get the following bounds.

#### Corollary 2.9

In the setup of Lemma 2.8 for any $r\ge 1$ we have

$$\begin{aligned}\mathbb {P}_{\rho }\!\left( \varphi _1=K+r\right)&\le \frac{1}{1-\delta }(2\delta ^2)^{r-1},\quad \text {and}\\ \mathbb {P}_{\rho }\!\left( \varphi _1\ge K+r\right)&\le \frac{1}{(1-\delta )(1-2\delta ^2)}(2\delta ^2)^{r-1}. \end{aligned}$$

#### Definition 2.10

Given a quasi-tree *T* with root $\rho$, let $T^a$ be obtained from *T* by adding an $\varepsilon$-weighted long-range edge from $\rho$ to a new vertex $\rho ^a$. (The root of $T^a$ is still at $\rho$.) Given a quasi-tree *T* and a vertex *x* that is the centre of an *R*-ball in *T*, let *T*(*x*) denote the quasi-tree that is formed by the *R*-ball of *x* and the *R*-balls that are its descendants in *T*. (The root of *T*(*x*) is at *x*.)

Given a random quasi-tree *T* corresponding to a graph *G*, let $(T',X')$ be distributed as $(T^a,X^a)$ conditioned on $X^a$ never hitting $\rho ^a$ (where $T^a$ is as above and $X^a$ is a random walk on it started from $\rho$). Note that we are conditioning the joint distribution of $(T^a,X^a)$, therefore in general $T'$ does not have the same distribution as *T*, and given $T'$, the process $X'$ is not a random walk on it. Let $\xi '$ be a loop-erased random walk on $T'$ from its root and let the times $(\sigma '_i)_{i\ge 0}$ and their levels $(\varphi '_i)_{i\ge 0}$ be defined as in Definition 2.6 for the tree $T'$ and the process $X'$ (instead of *T* and *X*).

Then $T'$, $X'$ and $\xi '$ also satisfy a version of Lemma 2.7 and Lemma 2.8 as follows.

#### Lemma 2.11

Let $K=0$ and let $T'$, $X'$, $\sigma '_1$ and $\xi '$ be as in Definition 2.10. Then for any realisation of $T'$ and for any $r\ge 1$ and any vertex *y* that is the centre of an *R*-ball at distance *r* from $\rho$ we almost surely have

$$\begin{aligned}\mathbb {P}_{\rho }\!\Big (X'_{\sigma '_1}=y\;\Big \vert \;T'\Big )\lesssim (2\delta ^2)^{r-1}\mathbb {P}_{\rho }\!\Big (\xi '_{r}=(x,y)\;\Big \vert \;T'\Big )\end{aligned}$$

where *x* is the long-range neighbour of *y*. If $r=1$ then we almost surely also have

$$\begin{aligned}\mathbb {P}_{\rho }\!\Big (X'_{\sigma '_1}=y\;\Big \vert \;T'\Big )\asymp \mathbb {P}_{\rho }\!\Big (\xi '_{1}=(x,y)\;\Big \vert \;T'\Big ).\end{aligned}$$

#### Proof

Using the definition of $X'$, and that by Lemma 2.4 we have $\mathbb {P}_{\rho }\!\Big (\tau _{\rho ^a}=\infty \;\Big \vert \;T^a\Big )=1-o(1)$ and writing $(\sigma _i^a)$ for the regeneration times of $X^a$ we get that

$$\begin{aligned}&\mathbb {P}_{\rho }\!\Big (X'_{\sigma '_1}=y\;\Big \vert \;T'\Big )=\mathbb {P}_{\rho }\!\Big (X^a_{\sigma ^a_1}=y\;\Big \vert \;T^a,\tau _{\rho ^a}=\infty \Big )\\&\quad =\frac{\mathbb {P}_{\rho }\!\Big (X^a_{\sigma ^a_1}=y,\tau _{\rho ^a}=\infty \;\Big \vert \;T^a\Big )}{\mathbb {P}_{\rho }\!\Big (\tau _{\rho ^a}=\infty \;\Big \vert \;T^a\Big )}\asymp \mathbb {P}_{\rho }\!\Big (X^a_{\sigma ^a_1}=y,\tau _{\rho ^a}=\infty \;\Big \vert \;T^a\Big )\\&\quad \le \mathbb {P}_{\rho }\!\Big (X^a_{\sigma ^a_1}=y\;\Big \vert \;T^a\Big ). \end{aligned}$$

Repeating the proof of Lemma 2.8 for $T^a$ instead of *T* we get that this is

$$\begin{aligned}\lesssim (2\delta ^2)^{r-1}\mathbb {P}_{\rho }\!\Big (\xi ^a_{r}=(x,y)\;\Big \vert \;T^a\Big ) = (2\delta ^2)^{r-1}\mathbb {P}_{\rho }\!\Big (\xi '_{r}=(x,y)\;\Big \vert \;T'\Big ),\end{aligned}$$

where the last equality follows by noting that $\xi ^a$ and $\xi '$ are loop-erased random walks on $T^a$ and $T'$ respectively. This finishes the proof of the first part of the result. For the second part of the result note that

$$\begin{aligned}&\mathbb {P}_{\rho }\!\Big (X'_{\sigma '_1}=y\;\Big \vert \;T'\Big )\asymp \mathbb {P}_{\rho }\!\Big (X^a_{\sigma ^a_1}=y,\tau _{\rho ^a}=\infty \;\Big \vert \;T^a\Big )\\&\quad \gtrsim \mathbb {P}_{\rho }\!\Big (\tau _{y}<\infty ,\tau _{y}<\tau _{\rho ^a}\;\Big \vert \;T^a\Big )\\&\quad \gtrsim \mathbb {P}_{\rho }\!\Big (\xi ^a_1=(x,y),\tau _{y}<\tau _{\rho ^a}\;\Big \vert \;T^a\Big )\asymp \mathbb {P}_{\rho }\!\Big (\xi '_{1}=(x,y)\;\Big \vert \;T'\Big )\end{aligned}$$

and this finishes the proof. $\square$

The proof of the following lemma is identical to the proof of the first and third points of [18, Lemma 3.6] and we omit the proof.

#### Lemma 2.12

Let *T* be a random quasi-tree associated to a graph *G*, with root $\rho$. Let $K\ge 0$ and let $T_0$ be a realisation of the first *K* levels of *T*. Let *X* be a simple random walk on *T* started from the root. Conditional on $\mathcal {B}(\rho ,K)=T_0$ we have that

(i) $\left( T(X_{\sigma _i})\setminus T(X_{\sigma _{i+1}}),(X_t)_{\sigma _i\le t\le \sigma _{i+1}}\right)$ are i.i.d. for $i\ge 1$ and are jointly independent from $\left( T\setminus T(X_{\sigma _{1}}),(X_t)_{0\le t\le \sigma _{1}}\right)$, and
(ii) for all $i\ge 1$, the pair $\left( T(X_{\sigma _i}),(X_t)_{t\ge \sigma _i}\right)$ has the same distribution as $(T',X')$.

The tail probability bounds for $(\sigma _i)$ and $(\varphi _i)$ from [18, Lemma 3.6] are no longer valid here and instead we get the following.

#### Lemma 2.13

There exist positive constants $c_1, c_2$ and *C* so that the following holds. Let *T* be a quasi-tree as in Definition 2.1, let *X* be a random walk on it from its root $\rho$ and let $(\sigma _i)$ and $(\varphi _i)$ be defined as in Definition 2.6 with $K=0$. Assume that $\varepsilon$ is small enough so that $\delta <\frac{1}{3}$. Then for any $r\ge 1$ we have

$$\begin{aligned} \mathbb {P}_{\rho }\!\left( \varphi _1\ge r\right)&\le C e^{-c_1(r-1)\log \left( \frac{1}{\varepsilon }\right) },\\ \mathbb {P}_{\rho }\!\left( \sigma _1\ge r\right)&\le Ce^{-c_2r\varepsilon }. \end{aligned}$$

#### Lemma 2.14

There exist positive constants $c_1, c_2$ and *C* so that the following holds. Let *T* be a quasi-tree with $\rho$ as in Definition 2.1. Let $(\sigma '_i)$ and $(\varphi '_i)$ be defined for $X'$ as before, with $K=0$. Assume that $\varepsilon <\frac{1}{27}$. Then

$$\begin{aligned} \mathbb {P}_{\rho }\!\left( \varphi '_1\ge r\right)&\le Ce^{-c_1(r-1)\log \left( \frac{1}{\varepsilon }\right) },\\ \mathbb {P}_{\rho }\!\left( \sigma '_1\ge r\right)&\le Ce^{-c_2r\varepsilon }. \end{aligned}$$

We also have $\mathbb {E}_{\rho }\!\left[ \sigma '_1\right] \ge \frac{1}{C\varepsilon }.$

Before proving these tail bounds, we will state and prove two immediate corollaries.

#### Corollary 2.15

There exist positive constants $c_1, c_2$ and *C* so that the following holds. Let *T* be a quasi-tree as in Definition 2.1, let *X* be a random walk from its root $\rho$ and let $(\sigma _i)$ and $(\varphi _i)$ be defined as in Definition 2.6 for some $K\ge 0$. Then for all $i\ge 1$ we have

$$\begin{aligned} \mathbb {P}_{\rho }\!\left( \varphi _{i+1}-\varphi _{i}\ge r\right) \le C e^{-c_1(r-1)\log \left( \frac{1}{\varepsilon }\right) },&\mathbb {P}_{\rho }\!\left( \sigma _{i+1}-\sigma _{i}\ge r\right) \le C e^{-c_2r\varepsilon } \quad \text { and }\\&\mathbb {E}_{\rho }\!\left[ \sigma _{i+1}-\sigma _{i}\right] \ge \frac{1}{C\varepsilon }. \end{aligned}$$

In case $K\ge 1$ we also have

$$\mathbb {P}_{\rho }\!\left( \varphi _{1}-\varphi _{0}\ge r\right) \le Ce^{-c_1(r-1)\log \left( \frac{1}{\varepsilon }\right) }.$$

(Note that in case $K=0$ we already have this bound.)

#### Proof

Applying Lemmas 2.12 and 2.14 gives the bounds for $(\sigma _{i+1}-\sigma _{i})$ and $(\varphi _{i+1}-\varphi _{i})$. In case $K\ge 1$, applying Lemma 2.14 to $T(X_L)$, where *L* is the last time when *X* enters level *K* gives the bound for $(\varphi _1-\varphi _0)$. $\square$

Corollary 2.9 and Lemma 2.13 immediately imply the following.

#### Corollary 2.16

In the setup of Corollary 2.15 for any $i\ge 1$ we have

$$\begin{aligned}\mathbb {E}\!\left[ \varphi _i-\varphi _{i-1}-1\right] \lesssim \delta ^2,\qquad \mathbb {E}\!\left[ (\varphi _i-\varphi _{i-1}-1)^2\right] \lesssim \delta ^2,\end{aligned}$$

and for any $i\ge 2$ we have

$$\begin{aligned}\mathbb {E}\!\left[ \sigma _i-\sigma _{i-1}\right] \asymp \frac{1}{\varepsilon },\qquad \mathbb {E}\!\left[ \left( \sigma _i-\sigma _{i-1}\right) ^2\right] \lesssim \frac{1}{\varepsilon ^2}.\end{aligned}$$

#### Proof of Lemma 2.13

By Corollary 2.9 and the bound on $\delta$ from Lemma 2.4 we have

$$\begin{aligned}\mathbb {P}_{\rho }\!\left( \varphi _1\ge r\right) \le \frac{1}{(1-\delta )(1-2\delta ^2)}(2\delta ^2)^{r-1}\lesssim (2\delta ^2)^{r-1}\lesssim e^{-c_1(r-1)\log \left( \frac{1}{\varepsilon }\right) }.\end{aligned}$$

Let *Y* be the random walk on the directed long-range edges induced by *X* and let $\sigma ^{Y}_1$ be the number of long-range edges crossed by *X* up to time $\sigma _1$. Then for any positive constants *a* and *b* we have

$$\begin{aligned}  &   \mathbb {P}_{\rho }\!\left( \sigma _1\ge r\right) \le \mathbb {P}_{\rho }\!\left( \varphi _1\ge a\varepsilon r\right) \,+\, \mathbb {P}_{\rho }\!\left( \varphi _1\le a\varepsilon r,\sigma ^{Y}_1\ge b\varepsilon r\right) \,\\  &   \quad + \mathbb {P}_{\rho }\!\left( \sigma ^{Y}_1\le b\varepsilon r,\sigma _1\ge r\right) . \end{aligned}$$

By the first part of the proof we have

$$\begin{aligned}\mathbb {P}_{\rho }\!\left( \varphi _1\ge a\varepsilon r\right) \le e^{-c_1\left( \lceil a\varepsilon r\rceil -1\right) \log \left( \frac{1}{\varepsilon }\right) }\lesssim 3\quad e^{-c_1'\varepsilon r}\end{aligned}$$

^3^

for some constant $c_1'>0$.

For $i=1,2,...$ let $\tau ^{(i)}$ be the number of steps between the *i*th and $(i+1)$th time that the walk crosses a long-range edge. Since at any vertex *v*, the walk has probability $\frac{\varepsilon }{\deg _G(v)+\varepsilon }\le \varepsilon$ of crossing a long-range edge, the sequence $\left( \tau ^{(i)}\right) _{i\ge 1}$ stochastically dominates a sequence of independent $\textrm{Geom}\!\left( \varepsilon \right)$ random variables. Also note that if $\sigma ^{Y}_1\le b\varepsilon r$ and $\sigma _1\ge r$ then $\tau ^{(1)}+...+\tau ^{({\lfloor b\varepsilon r\rfloor })}\ge r$. Hence (given that $b<1$) we have

$$\begin{aligned}&\mathbb {P}_{\rho }\!\left( \sigma ^{Y}_1\le b\varepsilon r,\sigma _1\ge r\right) \le \mathbb {P}\!\left( \textrm{NegBin}\!\left( \lfloor b\varepsilon r\rfloor ,\varepsilon \right) \ge r\right) =\mathbb {P}\!\left( \textrm{Bin}\!\left( r,\varepsilon \right) \le \lfloor b\varepsilon r\rfloor \right) \\&\quad \le 4\exp \left( -r\textrm{D}\left( b\varepsilon ||\varepsilon \right) \right) = \exp \left( -rb\varepsilon \log \left( \frac{b\varepsilon }{\varepsilon }\right) -r(1-b\varepsilon )\log \left( \frac{1-b\varepsilon }{1-\varepsilon }\right) \right) . \end{aligned}$$

^4^ Note that $\varepsilon <\frac{1}{2}$ and $b<1$, hence $\frac{(1-b)\varepsilon }{1-\varepsilon }<1$ and so

$$\begin{aligned}\log \left( \frac{1-b\varepsilon }{1-\varepsilon }\right) =\log \left( 1+\frac{(1-b)\varepsilon }{1-\varepsilon }\right) \ge \frac{1}{2}\frac{(1-b)\varepsilon }{1-\varepsilon }.\end{aligned}$$

Also for any sufficiently small $b\le \frac{1}{4}$ we have

$$\begin{aligned}\frac{1}{2}(1-b\varepsilon )\frac{(1-b)\varepsilon }{1-\varepsilon } > \frac{1}{2}(1-b)\varepsilon \ge b\log \left( \frac{1}{b}\right) \varepsilon .\end{aligned}$$

Together these show that for $b\le \frac{1}{4}$ and some positive constant $c'$ (depending on *b*) we have

$$\begin{aligned}\mathbb {P}_{\rho }\!\left( \sigma ^{Y}_1\le b\varepsilon r,\sigma _1\ge r\right) \le \exp \left( -c'r\varepsilon \right) .\end{aligned}$$

Note that if $\varphi _1\le a\varepsilon r$ and $\sigma ^{Y}_1\ge b\varepsilon r$, then $\inf _{j\ge b\varepsilon r}\ell (Y_{j})\le a\varepsilon r$. We know that $\ell (Y)$ stochastically dominates a biased random walk *S* on $\mathbb {Z}$ that steps right with probability $1-\delta$ and left with probability $\delta$. Hence (given that $\frac{2a+b}{2}\le (1-\delta )b$) we have

$$\begin{aligned}&\mathbb {P}_{\rho }\!\left( \varphi _1\le a\varepsilon r,\sigma ^{Y}_1\ge b\varepsilon r\right) \le \mathbb {P}_{\rho }\!\left( \inf _{j\ge b\varepsilon r}\ell (Y_{j})\le a\varepsilon r\right) \le \mathbb {P}_{0}\!\left( \inf _{j\ge b\varepsilon r}S_j\le a\varepsilon r\right) \\&\quad \le \mathbb {P}_{0}\!\left( S_{\lceil b\varepsilon r\rceil }\le 2a\varepsilon r\right) +\mathbb {P}_{\lceil 2a\varepsilon r\rceil }\!\left( \inf _{j}S_j\le a\varepsilon r\right) \\&\quad \le \mathbb {P}\!\left( \textrm{Bin}\!\left( \lceil b\varepsilon r\rceil ,1-\delta \right) \le \frac{2a\varepsilon r+\lceil b\varepsilon r\rceil }{2}\right) + \left( \frac{\delta }{1-\delta }\right) ^{a\varepsilon r}\\&\quad \le 5\,\exp \left( -2\lceil b\varepsilon r\rceil \left( 1-\delta -\frac{2a\varepsilon r+\lceil b\varepsilon r\rceil }{2\lceil b\varepsilon r\rceil }\right) ^2\right) \\&\qquad +\exp \left( -a\varepsilon \left( \log \left( \frac{1}{\delta }\right) -\log \left( \frac{1}{1-\delta }\right) \right) r\right) \\&\quad \le \exp \left( -2b\varepsilon r\left( 1-\delta -\frac{a\varepsilon r}{\lceil b\varepsilon r\rceil }-\frac{1}{2}\right) ^2\right) +\exp \left( -\frac{1}{2}a\varepsilon \log \left( \frac{1}{\delta }\right) r\right) . \end{aligned}$$

^5^ Choosing say $b=\frac{1}{4}$ and $a=\frac{1}{12}b$ we have $1-\delta -\frac{a\varepsilon r}{\lceil b\varepsilon r\rceil }-\frac{1}{2}\ge 1-\frac{1}{3}-\frac{a}{b}-\frac{1}{2}\ge \frac{1}{12}$.

This gives an overall bound of form

$$\mathbb {P}_{\rho }\!\left( \sigma _1\ge r\right) \lesssim e^{-c_2r\varepsilon }$$

and this finishes the proof. $\square$

#### Proof of Lemma 2.14

Repeating the above proof for $T^a$ and $X^a$ we get the desired tail bounds for $\varphi ^a_1$ and $\sigma ^a_1$.

Using that $\mathbb {P}\!\left( X^a\text { does not hit }\rho ^a\right) \ge 1-\delta \gtrsim 1$ we get that

$$\begin{aligned} \mathbb {P}_{\rho }\!\left( \varphi '_1\ge r\right)&= \mathbb {P}_{\rho }\!\Big (\varphi ^a_1\ge r\;\Big \vert \;\tau _{\rho ^a}=\infty \Big )=\frac{\mathbb {P}_{\rho }\!\left( \varphi ^a_1\ge r,\tau _{\rho ^a}=\infty \right) }{\mathbb {P}_{\rho }\!\left( \tau _{\rho ^a}=\infty \right) }\\&\lesssim \mathbb {P}_{\rho }\!\left( \varphi ^a_1\ge r\right) \lesssim e^{-c_1(r-1)\log \left( \frac{1}{\varepsilon }\right) }. \end{aligned}$$

Similarly, we get

$$\begin{aligned}\mathbb {P}_{\rho }\!\left( \sigma '_1\ge r\right) \lesssim e^{-c_2r\varepsilon }.\end{aligned}$$

Let $\tau ^a_1$ be the first time when $X^a$ crosses a long-range edge. Then $\sigma ^a_1\ge \tau ^a_1\ge _{\text {st}}\textrm{Geom}_{\ge 1}\!\left( \varepsilon \right)$ (where $A\ge _{\textrm{st}}B$ means that *A* stochastically dominates *B*), hence

$$\begin{aligned} \mathbb {E}_{\rho }\!\left[ \sigma '_1\right]&= \frac{\mathbb {E}_{\rho }\!\left[ \sigma ^a_1{{{\mathfrak {1}}}}_{\tau _{\rho ^a}=\infty }\right] }{\mathbb {P}_{\rho }\!\left( \tau _{\rho ^a}=\infty \right) }\ge \mathbb {E}_{\rho }\!\left[ \tau ^a_1{{{\mathfrak {1}}}}_{\tau _{\rho ^a}=\infty }\right] = \sum _{k\ge 1}\mathbb {P}_{\rho }\!\left( \tau ^a_1{{{\mathfrak {1}}}}_{\tau _{\rho ^a}=\infty }\ge k\right) \\&=\sum _{k\ge 1}\mathbb {P}_{\rho }\!\left( \tau ^a_1\ge k\right) \mathbb {P}_{\rho }\!\Big (\tau _{\rho ^a}=\infty \;\Big \vert \;\tau ^a_1\ge k\Big )\ge \sum _{k\ge 1}(1-\varepsilon )^k(1-\delta )\asymp \frac{1}{\varepsilon }. \end{aligned}$$

The last inequality holds because $\mathbb {P}_{\rho }\!\left( \tau ^a_1\ge k\right) \ge (1-\varepsilon )^k$ and

$$\begin{aligned} \mathbb {P}_{\rho }\!\Big (\tau _{\rho ^a}=\infty \;\Big \vert \;\tau ^a_1\ge k\Big )&= \sum _{u}\mathbb {P}_{\rho }\!\Big (X^a_{k-1}=u\;\Big \vert \;\tau ^a_1>k-1\Big )\mathbb {P}_{u}\!\left( \tau _{\rho ^a}=\infty \right) \\&\ge \sum _{u}\mathbb {P}_{\rho }\!\Big (X^a_{k-1}=u\;\Big \vert \;\tau ^a_1>k-1\Big )(1-\delta )= 1-\delta . \end{aligned}$$

This finishes the proof. $\square$

We conclude this section by proving two useful statements that we will use later.

#### Lemma 2.17

There exist positive constants $c_1, c_2$ and *C* so that the following holds. Let *T* be a quasi-tree with root $\rho$ and let *e* be a long-range edge from the *R*-ball of $\rho$. Let *X* be a random walk and $\xi$ a loop-erased random walk on *T*. Let $\tau _e$ be the first time when *X* crosses *e* and let $\tau _1$ be the first time when *X* crosses a long-range edge (i.e. it hits level 1). Then for any vertex *v* in the *R*-ball of $\rho$ and for any $\ell _0$ we have

$$\begin{aligned} \mathbb {P}_{v}\!\left( e\in \xi \right) = (1-o(1))\mathbb {P}_{v}\!\left( \tau _e<\infty \right) \le C (e^{c_1\ell _0\varepsilon \delta }\mathbb {P}_{v}\!\left( X_{\tau _1}=e^+\right) +e^{-c_2\ell _0\varepsilon }). \end{aligned}$$

#### Proof

Let $e=(e^-,e^+)$ where $e^-$ is the vertex in the *R*-ball of $\rho$, and for each vertex $x\ne \rho$ in this *R*-ball let $x^+$ denote its long-range neighbour.

For the equality simply note that

$$\begin{aligned}\mathbb {P}_{v}\!\left( \tau _e<\infty \right) \ge \mathbb {P}_{v}\!\left( e\in \xi \right) \ge (1-\delta )\mathbb {P}_{v}\!\left( \tau _e<\infty \right) .\end{aligned}$$

For the inequality, grouping the possible paths to *e* by their restriction $v=x_0,x_1,...,x_{\ell -1}=e^-$ to the *R*-ball of $\rho$, we get

$$\begin{aligned}&\mathbb {P}_{v}\!\left( \tau _e<\infty \right) \\&\quad = \sum _{\ell }\sum _{x_0,...,x_{\ell -1}}\left( \prod _{i=0}^{\ell -2}\left( P(x_i,x_{i+1})\sum _{j=0}^{\infty }\left( P(x_i,x_i^+)\mathbb {P}_{x_i^+}\!\left( \tau _{x_i}<\infty \right) \right) ^j\right) \right) P(e^-,e^+)\\&\quad \le \sum _{\ell }\sum _{x_0,...,x_{\ell -1}}\left( \frac{1}{1-\varepsilon \delta }\right) ^{\ell }\mathbb {P}_{v}\!\left( X_1=x_1,...,X_{\ell -1}=x_{\ell -1},X_\ell =e^+\right) \end{aligned}$$

where the sum is taken over all sequences such that $x_0=v$, $x_{\ell -1}=e^-$ and $x_0,...,x_{\ell -1}$ are in the *R*-ball of $\rho$, and *P* is the transition matrix of *X*. For the inequality we used that $P(x_i,x_i^+)\mathbb {P}_{x_i^+}\!\left( \tau _{x_i}<\infty \right) \le \varepsilon \delta$. The sum on the last line above is then equal to

$$\begin{aligned}&=\sum _{\ell }\left( \frac{1}{1-\varepsilon \delta }\right) ^\ell \mathbb {P}_{v}\!\left( \tau _1=\ell ,X_{\tau _1}=e^+\right) \\&\quad \le \left( \frac{1}{1-\varepsilon \delta }\right) ^{\ell _0}\mathbb {P}_{v}\!\left( X_{\tau _1}=e^+\right) + \sum _{\ell \ge \ell _0}\left( \frac{1}{1-\varepsilon \delta }\right) ^\ell \left( 1-\frac{\varepsilon }{\Delta +1}\right) ^{\frac{\ell }{2}-1}\varepsilon \\&\quad \lesssim \left( \frac{1}{1-\varepsilon \delta }\right) ^{\ell _0}\mathbb {P}_{v}\!\left( X_{\tau _1}=e^+\right) + \left( \frac{\sqrt{1-\frac{\varepsilon }{\Delta +1}}}{1-\varepsilon \delta }\right) ^{\ell _0}\frac{\varepsilon }{1-\left( \frac{\sqrt{1-\frac{\varepsilon }{\Delta +1}}}{1-\varepsilon \delta }\right) }\\&\quad \lesssim e^{c_1\ell _0\varepsilon \delta }\mathbb {P}_{v}\!\left( X_{\tau _1}=e^+\right) + e^{-c_2\ell _0\varepsilon }. \end{aligned}$$

For the second sum in the second line above we used that for any $x\ne \rho$ there is a long-range edge coming out of *x* and that the walk cannot be at $\rho$ at two consecutive steps. In the last inequality we used that $\log (1-\theta )\asymp -\theta$ around 0 and that $1-\left( \frac{\sqrt{1-\frac{\varepsilon }{\Delta +1}}}{1-\varepsilon \delta }\right) \asymp \varepsilon$. $\square$

#### Lemma 2.18

There exist positive constants *C* and $c'$ so that the following holds. Let *T* be a quasi-tree as in Definition 2.1. Let *v* be a vertex of *T* and let *e* be a long-range edge from the *R*-ball of *v*. Let *X* be a random walk on *T* from *v*. Then for any $\ell _0$ we have

$$\begin{aligned}  &   \mathbb {P}_{v}\!\left( X\text { hits level }\ell (v)+m\text { and then hits }e^+\right) \\  &   \quad \le C\left( \ell _0\delta ^m\mathbb {P}_{v}\!\left( X\text { hits }e^+\right) +\delta ^{m}e^{-c'\varepsilon \ell _0}\left( \ell _0+\frac{1}{\varepsilon }\right) \right) . \end{aligned}$$

#### Proof

By grouping the possible paths from *v* to $e^+$ that pass through level $\ell (v)+m$ by their restriction to the *R*-ball of *v* we get that

$$\begin{aligned}&\mathbb {P}_{v}\!\left( X\text { hits level }\ell (v)+m\text { and then hits }e^+\right) \\&\quad \le \sum _{\ell }\sum _{x_0,...,x_\ell }\sum _{r=0}^{\ell -1}\left( \prod _{s\ne r}\mathbb {P}_{x_s}\!\left( \text {hit }x_{s+1}\text { without visiting another vertex in the }R\text {-ball}\right) \right) \\&\qquad \cdot \mathbb {P}_{x_r}\!\left( \text {hit }x_{r+1}\text { after visiting level }\ell (v)+m, \text { without visiting another vertex in the }R\text {-ball}\right) \end{aligned}$$

where the second sum is taken over all paths $x_0,...,,x_\ell$ such that $x_0=v$, $x_{\ell }=e^{+}$ and $x_0,...x_{\ell -1}$ are in the *R*-ball of *v*. This is then

$$\begin{aligned}\le \sum _{\ell }\sum _{x_0,...,x_\ell }\ell \delta ^{m}\prod _{s}\mathbb {P}_{x_s}\!\left( \text {hit }x_{s+1}\text { without visiting another vertex in the }R\text {-ball}\right) .\end{aligned}$$

First let us bound the terms with $\ell \le \ell _0$.

$$\begin{aligned}&\sum _{\ell \le \ell _0}\sum _{x_0,...,x_\ell }\ell \delta ^{m}\prod _{s}\mathbb {P}_{x_s}\!\left( \text {hit }x_{s+1}\text { without visiting another vertex in the }R\text {-ball}\right) \\&\quad \le \sum _{\ell \le \ell _0}\ell \delta ^{m}\mathbb {P}_{v}\!\left( \text {hit }e^+\text { after visiting exactly }(\ell -1)\text { vertices in the }R\text {-ball}6\right) \\&\quad \le \ell _0\delta ^m\mathbb {P}_{v}\!\left( \text {hit }e^+\right) . \end{aligned}$$

^6^

Now let us bound the terms with $\ell >\ell _0$. Note that

$$\begin{aligned}&\mathbb {P}_{x_s}\!\left( \text {hit }x_{s+1}\text { without visiting another vertex in the }R\text {-ball}\right) \\&\quad =\sum _{j=0}^\infty \left( P(x_s,x_s^+)\mathbb {P}_{x_s^+}\!\left( \text {hit }x_s\right) \right) ^jP(x_s,x_{s+1})\\&\quad \le \sum _{j=0}^\infty \left( \varepsilon \delta \right) ^jP(x_s,x_{s+1}) =\frac{1}{1-\varepsilon \delta }P(x_s,x_{s+1}). \end{aligned}$$

Hence

$$\begin{aligned}&\sum _{\ell>\ell _0}\sum _{x_0,...,x_\ell }\ell \delta ^{m}\prod _{s}\mathbb {P}_{x_s}\!\left( \text {hit }x_{s+1}\text { without visiting another vertex in the }R\text {-ball}\right) \\&\quad \le \sum _{\ell>\ell _0}\sum _{x_0,...,x_\ell }\ell \delta ^{m}\left( \frac{1}{1-\varepsilon \delta }\right) ^\ell \prod _{s}P(x_s,x_{s+1})\\&\quad =\sum _{\ell >\ell _0}\ell \delta ^{m}\left( \frac{1}{1-\varepsilon \delta }\right) ^\ell \mathbb {P}_{v}\!\left( \text {hit }e^+\text { after exactly }\ell \text { steps, without leaving the }R\text {-ball}\right) . \end{aligned}$$

(More precisely, in the last probability above we mean that only the last step is allowed to be outside of the *R*-ball.) At each step when the walk is not in $\rho$, it has probability $\le 1-\frac{\varepsilon }{\Delta +1}$ of not leaving the *R*-ball. When the walk is in $e^-$ it has probability $\le \varepsilon$ of crossing to $e^+$. The walk cannot be at $\rho$ in two consecutive steps, hence

$$\begin{aligned}\mathbb {P}_{v}\!\left( \text {hit }e^+\text { after exactly }\ell \text { steps, without leaving the }R\text {-ball}\right) \le \left( 1-\frac{\varepsilon }{\Delta +1}\right) ^{\frac{\ell }{2}-1}\varepsilon .\end{aligned}$$

This then gives

$$\begin{aligned}&\sum _{\ell >\ell _0}\ell \delta ^{m}\left( \frac{1}{1-\varepsilon \delta }\right) ^\ell \mathbb {P}_{v}\!\left( \text {hit }e^+\text { after exactly }\ell \text { steps, without leaving the }R\text {-ball}\right) \\&\quad \le \delta ^{m}\sum _{\ell \ge \ell _0}\ell \left( \frac{1}{1-\varepsilon \delta }\right) ^\ell \left( 1-\frac{\varepsilon }{\Delta +1}\right) ^{\frac{\ell }{2}-1}\varepsilon = \delta ^{m}\frac{\varepsilon }{1-\frac{\varepsilon }{\Delta +1}} \sum _{\ell \ge \ell _0}\ell \left( \frac{\sqrt{1-\frac{\varepsilon }{\Delta +1}}}{1-\varepsilon \delta }\right) ^\ell \\&\quad \asymp \delta ^{m}\left( \frac{\sqrt{1-\frac{\varepsilon }{\Delta +1}}}{1-\varepsilon \delta }\right) ^{\ell _0}\left( \ell _0+\frac{1}{\varepsilon }\right) \lesssim \delta ^{m}e^{-c'\varepsilon \ell _0}\left( \ell _0+\frac{1}{\varepsilon }\right) . \end{aligned}$$

In the second last step we used that for any $\ell _0$ and any $p\in (0,1)$ we have

$$\begin{aligned}\sum _{\ell \ge \ell _0}\ell p^\ell = p^{\ell _0}\left( \frac{\ell _0}{1-p}+\frac{p}{(1-p)^2}\right) .\end{aligned}$$

Putting everything together gives the result. $\square$

### Speed and entropy

#### Lemma 2.19

Let *X* be a simple random walk on a random quasi-tree *T* induced by a graph *G* and let $\nu :=\frac{\mathbb {E}\!\left[ \varphi _2-\varphi _1\right] }{\mathbb {E}\!\left[ \sigma _2-\sigma _1\right] }$. Then $\nu \asymp \varepsilon$ and the (long-range) distance of $\rho$ and $X_t$ on *T* (as given in Definition 2.1) almost surely satisfies

$$\begin{aligned} \frac{ d_T(\rho , X_t)}{t}\rightarrow \nu \text { as } t\rightarrow \infty . \end{aligned}$$

Moreover, for all $\theta >0$, $J\ge 1$ there exists a positive constant *C* (depending on $\Delta$, $\theta$ and *J*) so that for all $t\ge \frac{J}{\varepsilon }$ we have

$$\begin{aligned} \mathbb {P}\!\left( |d_T(\rho ,X_t)-\nu t|>C\sqrt{\varepsilon t}\right) \le \theta \quad \text { and } \quad \mathbb {P}\!\left( \sup _{s:\,s\le t}d_T(\rho ,X_s)>\nu t+ 2C\sqrt{\varepsilon t}\right) \le \theta . \end{aligned}$$

The proof of this uses the iid structure between regenerations and the ergodic theorem. It is analogous to the proof of [18, Lemma 3.11] and so it is deferred to the supplementary material [1].

#### Proposition 2.20

Let *T* be a random quasi-tree corresponding to a graph *G* as in Definition 2.1, let $\xi$ and $\widetilde{\xi }$ be two independent loop-erased random walks on *T*, both started from the root $\rho$ and let $T'$, $X'$, $\sigma '_1$ and $\xi '$ be as in Definition 2.10, with $\xi '$ independent of $X'$ (conditional on $T'$). Let

$$\begin{aligned} \mathfrak {h}:=\frac{1}{\mathbb {E}\!\left[ \varphi _2-\varphi _1\right] }\cdot \mathbb {E}\!\left[ -\log \mathbb {P}_{\rho }\!\Big (X'_{\sigma '_1}\in \xi '\;\Big \vert \;X',T'\Big )\right] . \end{aligned}$$

Then almost surely

$$\begin{aligned} \frac{-\log \mathbb {P} \!\Big (\xi _k\in \widetilde{\xi }\;\Big \vert \;T,\xi \Big ) }{k}\rightarrow \mathfrak {h}\text { as } k\rightarrow \infty . \end{aligned}$$

Let

$$\begin{aligned} \mathfrak {V}:=\mathfrak {h}^2\vee \textrm{Var}\left( -\log \mathbb {P}_{\rho }\!\Big (X'_{\sigma '_1}\in \xi '\;\Big \vert \;T',X'\Big )\right) , \end{aligned}$$

and let $$Y'=-\log \mathbb {P}_{\rho }\!\Big (X'_{\sigma '_1}\in \xi '\;\Big \vert \;X',T'\Big )$$.

Assume that $\mathbb {E}\!\left[ (Y'-\mathbb {E}\!\left[ Y'\right] )^2\right] \lesssim \mathbb {E}\!\left[ Y'\right] ^2$ and $\mathbb {E}\!\left[ (Y'-\mathbb {E}\!\left[ Y'\right] )^4\right] \lesssim \mathbb {E}\!\left[ Y'\right] ^4$. (This will in particular imply that $\mathfrak {V}\asymp \mathfrak {h}^2$.)

Fix $K\ge 0$ and let $T_0$ be a realisation of the first *K* levels of *T*. Let *b*(*R*) be such that $b(R)\ge \frac{c}{\varepsilon }$ and $\log b(R)\ge c\mathfrak {h}$ for some constant *c* and the number of vertices in any *R*-ball of *T* is at most *b*(*R*). Then for all $\theta >0$, there exists a positive constant *C* (depending only on $\theta$ and $\Delta$) so that for all $k\ge \frac{(K\log b(R))^2}{\mathfrak {V}}\vee 1$ we have

2.2 $$\begin{aligned} \mathbb {P} \!\Big (\left| -\log \mathbb {P} \!\Big (\xi _k\in \widetilde{\xi }\;\Big \vert \;T,\xi \Big ) -\mathfrak {h}k\right| >C\sqrt{k\mathfrak {V}}\;\Big \vert \;\mathcal {B}_K(\rho )=T_0\Big )\le \theta . \end{aligned}$$

The convergence result again uses the iid structure between regenerations, and follows similarly to [18, Proposition 3.15]. In the proof of the concentration result we need to control the covariances of quantities similar to $Y'$ corresponding to the parts of the walk between two consecutive regeneration times. This becomes much more technical than [18, Proposition 3.15], and in the proof we use the assumptions on the moments of $Y'$. (We later verify these assumptions for the graphs we consider.) The bounds we use on the above covariances are summarised in the following lemma. Given the lemma, the remaining parts of the proof of Proposition 2.20 are analogous to the proof of [18, Proposition 3.15], and deferred to [1].

#### Lemma 2.21

Let us consider the setup of Proposition 2.20. Then we have

$$\begin{aligned}\textrm{Var}\left( -\log \mathbb {P}_{\rho }\!\Big (\xi _{\varphi _k}\in \widetilde{\xi }\;\Big \vert \;\xi ,T\Big )\;\Bigg \vert \;T_0\right) \lesssim k\mathfrak {V}+\left( K\log b(R)\right) ^2.\end{aligned}$$

#### Proof of Lemma 2.21

The proof idea is similar to the proof of [18, Lemma 3.14]. The full details are given in [1]. $\square$

The following lemma will help us to find the order of $\mathfrak {h}$ and $\mathfrak {V}$ (as functions of *n* and $\varepsilon$) in Proposition 2.20 for specific sequences of graphs and to check that the assumptions of (2.2) hold.

#### Lemma 2.22

Let *G* be as before. Let $\rho$ be any vertex of *G* and let *T* be any realisation of the random quasi-tree corresponding to *G*, rooted at $\rho$. Let *v* be any vertex of *G* such that the ball of radius $\frac{R}{2}$ around *v* is contained in the ball of radius *R* around $\rho$ (the balls are in graph distance). Let *Y* be a simple random walk on *G* from *v* and let *E* be a random variable taking values on $\mathbb {Z}_{\ge 0}$ such that $\mathbb {P}_{v}\!\Big (E=k\;\Big \vert \;E\ge k,Y_k=u\Big )=\frac{\varepsilon }{\deg (u)+\varepsilon }$ for all *k* and all *u*. Let $\left( \widetilde{Y},\widetilde{E}\right)$ be an independent copy of $\left( Y,E\right)$. Let $b\in \mathbb {Z}_{\ge 1}$. Let $\xi$ and $\widetilde{\xi }$ be independent loop-erased random walks on *T*. Also let $T'$, $X'$ and $\xi '$ be as in Definition 2.10, with $T'$ rooted at $\rho$. Let $\widetilde{\xi }'$ be an independent copy of $\xi '$ given $T'$.

Assume that there exist $h_b$ and *b*(*R*) (depending on *n*) such that the following properties hold.

(i) For any realisation of *T* and any choice of *v* we have
  $$\begin{aligned}\mathbb {E}\!\left[ \left( -\log \mathbb {P}_{v}\!\Big (Y_E=\widetilde{Y}_{\widetilde{E}}\;\Big \vert \;Y\Big )\right) ^b\right] \asymp h_b,\end{aligned}$$
  where the implicit constants in $\asymp$ do not depend on *T* and *v*.
(ii) The size of all *R*-balls in *G* is upper bounded by *b*(*R*), and *b*(*R*) satisfies
  $$\begin{aligned}\left( \log b(R)\right) ^b\left( 1-\frac{\varepsilon }{\Delta +\varepsilon }\right) ^{\frac{R}{2}}\ll h_b.\end{aligned}$$
(iii) $$R\gg \frac{1}{\varepsilon }$$.
(iv) The above assumptions also hold, with the same value of $h_b$, if we set the value of *R* to be *n*.

Then for any realisation of $T'$ we have

2.3 $$\begin{aligned} \mathbb {E}\!\left[ \left( -\log \mathbb {P}_{\rho }\!\Big (X'_{\sigma '_1}\in \widetilde{\xi }'\;\Big \vert \;X',T'\Big )\right) ^b\;\Bigg \vert \;T'\right] \asymp h_b, \end{aligned}$$

where the implicit constants in $\asymp$ do not depend on $T'$.

The proof is given in [1] and consists of the following steps. Firstly, we prove a statement similar to (2.3) with $X_{\tau _1}$ and $\widetilde{X}_{\widetilde{\tau }_1}$ on *T* ( [1, Lemma II.1]). Next, we show that the expectation stays the same order if we replace $X_{\tau _1}$ and $\widetilde{X}_{\widetilde{\tau }_1}$ with $\xi _1$ and $\widetilde{\xi }_1$ ( [1, Lemma II.2]). Then we replace these with $\xi '_1$ and $\widetilde{\xi }'_1$ on $T'$ ( [1, Lemma II.3]), and finally we replace $\xi '_1$ with $X'_{\sigma '_1}$ to get (2.3) ( [1, Lemma II.4]).

In what follows we will use the following notation for entropy and the analogous expectation with a higher power of the $\log$. We will also make use of some simple results listed in Appendix A.

#### Definition 2.23

For $b\in \mathbb {Z}_{\ge 1}$ and for a sequence $(p_i)$ of real numbers taking values in [0, 1] let

$$\begin{aligned}H_b(p_1,p_2,...):= \sum _{i}p_i(-\log p_i)^b,\end{aligned}$$

and for a random variable *W* taking values in a (countable) set $\mathcal {W}$ let

$$\begin{aligned}H_b(W):=\sum _{w\in \mathcal {W}}\mathbb {P}\!\left( W=w\right) \left( -\log \mathbb {P}\!\left( W=w\right) \right) ^b.\end{aligned}$$

In both cases $p(-\log p)^b$ is considered to be 0 when $p=0$.

#### Definition 2.24

For random variables *W*, *Z* taking values in (countable) sets $\mathcal {W}$ and $\mathcal {Z}$ respectively let

$$\begin{aligned} H_b(W|Z)&:=\sum _{z\in \mathcal {Z}}\mathbb {P}\!\left( Z=z\right) H_b(W|Z=z)\\&=\sum _{z\in \mathcal {Z}}\mathbb {P}\!\left( Z=z\right) \sum _{w\in \mathcal {W}}\mathbb {P} \!\Big (W=w\;\Big \vert \;Z=z\Big )\left( -\log \mathbb {P} \!\Big (W=w\;\Big \vert \;Z=z\Big )\right) ^b. \end{aligned}$$

Here $\mathbb {P}\!\left( Z=z\right) \mathbb {P} \!\Big (W=w\;\Big \vert \;Z=z\Big )\left( -\log \mathbb {P} \!\Big (W=w\;\Big \vert \;Z=z\Big )\right) ^b$ is considered to be 0 when $\mathbb {P}\!\left( Z=z\right) =0$ or $\mathbb {P} \!\Big (W=w\;\Big \vert \;Z=z\Big )=0$.

## Relating $G^*$ and *T*

As mentioned earlier, we will be mostly interested in two classes of graphs *G*, graphs with linear growth of entropy, and graphs with polynomial growth of balls. In the former case we will prove cutoff when $\varepsilon _n\gg \frac{1}{\log n}$, i.e. when $\varepsilon _n$ can be written as $\varepsilon _n=\frac{g(n)}{\log n}$ where $g(n)\le \log n$ is a function growing to infinity. In the latter case we will prove cutoff when $\varepsilon _n=n^{-o(1)}$, i.e. when $\varepsilon _n$ can be written as $\varepsilon _n=\exp \left( -\frac{\log n}{g(n)}\right)$ where *g*(*n*) is a function growing to infinity. In the two cases we will be able to write $\mathfrak {h}$ and $\mathfrak {V}$ in the same form using *g*(*n*) and $\log n$ and will also choose the parameters in the proofs to be of the same form in terms of $\varepsilon _n$, *g*(*n*) and $\log n$.

The reason that it is possible is that in both cases the entropy of the random walk on *G* and the growth of balls of *G* can be expressed using the same function *f* that relates $\varepsilon _n$ and $\frac{\log n}{g(n)}$. This motivates the following assumption.

### Assumption 3.1

(i) *f*(*t*) is a continuous increasing function $\mathbb {R}_{>0}\rightarrow \mathbb {R}_{>0}$ that satisfies $f(0^+)=0^+$, $f(\infty )=\infty$ and there exist positive constants *c* and *C* such that $c\log t\le f(t)\le C t$.
(ii) For any positive constant $\widehat{c},$ there exist positive constants *c* and *C* depending on $\widehat{c}$ such that for any $t\le \frac{\widehat{c}}{\varepsilon _n}$, $a\in \{1,2,4\}$ and for all $x_0$, a random walk *X* on *G* with $X_0=x_0$ satisfies that $c f(t)^a\le H_a(X_t)\le C f(t)^a$.
(iii) There exist a function *b* and positive constants *c* and *C* such that for any *t* the size of any ball of radius *t* in *G* is upper bounded by *b*(*t*), and we have $cf(t)\le \log b(t)\le C f(t)$ for all *t*.
(iv) There exist positive constants *C* and $\beta$ such that $\varepsilon _n\le C(\log n)^{-\beta }$.^7^
(v) There exists a function *g*(*n*) growing to infinity and there exist positive constants *c* and *C* such that $c\frac{\log n}{g(n)} \le f\left( \frac{1}{\varepsilon _n}\right) \le C\frac{\log n}{g(n)}$.
(vi) For any positive constant $\widehat{c}$ there exist positive constants *c* and *C* such that $c f\left( \frac{1}{\varepsilon _n}\right) \le f\left( \frac{\widehat{c}}{\varepsilon _n}\right) \le C f\left( \frac{1}{\varepsilon _n}\right)$ for all sufficiently large values of *n*.
(vii) There exists a positive constant *C* such that for all $x,y\ge C$ we have $f(xy)\le f(x)f(y)$.

Throughout this section and the next section, we will assume that Assumption 3.1 holds (in addition to the assumptions in Definition 1.1 and the assumption that the graphs $G_n$ have bounded degrees and $\varepsilon _n\rightarrow 0$).

In Sect. 5 we will show that graphs with linear growth of entropy satisfy it with $f(t)=t$, while graphs with polynomial growth of balls satisfy it with $f(t)=\log (1+t)$.

### Lemma 3.2

Suppose that Assumption 3.1 holds and assume that $R\gtrsim \frac{1}{\varepsilon }\log g(n)$. Let $K\asymp \frac{g(n)\log g(n)}{\log n}\asymp \frac{\log g(n)}{f\left( \frac{1}{\varepsilon }\right) }$ and let $T_0$ be a realisation of the first *K* levels of the random quasi-tree *T* corresponding to *G*. Then $\mathfrak {h}$ and $\mathfrak {V}$ defined as in Proposition 2.20 satisfy $\mathfrak {h}\asymp f\left( \frac{1}{\varepsilon }\right)$ and $\mathfrak {V}\asymp f\left( \frac{1}{\varepsilon }\right) ^2$, and for all $\theta >0$ there exists a positive constant *C* (depending only on $\theta$ and $\Delta$) so that for all $k\ge \frac{(K\log b(R))^2}{\mathfrak {V}}$ we have

3.1 $$\begin{aligned} \mathbb {P} \!\Big (\left| -\log \mathbb {P} \!\Big (\xi _k\in \widetilde{\xi }\;\Big \vert \;T,\xi \Big ) -\mathfrak {h}k\right| >C\sqrt{k\mathfrak {V}}\;\Big \vert \;\mathcal {B}_K(\rho )=T_0\Big )\le \theta . \end{aligned}$$

### Proof

We will show that for $b\in \{1,2,4\}$ the assumptions of Lemma 2.22 hold with $h_b\asymp f\left( \frac{1}{\varepsilon }\right) ^b$ and that Proposition 2.20 holds.

Let *Y* and *E* be defined as in Lemma 2.22. First we wish to show that $H_b(Y_E)\asymp f\left( \frac{1}{\varepsilon }\right) ^b$. By Lemma A.5 we know that

$$\begin{aligned} H_b\left( Y_E\Bigg |E\right) \le H_b(Y_E)\lesssim H_b\left( Y_E\Bigg |E\right) +H_b(E), \end{aligned}$$

so it is sufficient to show that $H_b\left( Y_E\big |E\right) \asymp f\left( \frac{1}{\varepsilon }\right) ^b$ and $H_b(E)\lesssim f\left( \frac{1}{\varepsilon }\right) ^b$.

First, note that for any $k\in \mathbb {Z}_{\ge 0}$ we have

$$\begin{aligned} \mathbb {P}\!\left( E=k\right)&=\left( \prod _{i=1}^k\mathbb {P} \!\Big (E\ge i\;\Big \vert \;E\ge i-1\Big )\right) \mathbb {P} \!\Big (E=k\;\Big \vert \;E\ge k\Big )\\&\le \left( \frac{\Delta }{\Delta +\varepsilon }\right) ^k\frac{\varepsilon }{1+\varepsilon }\asymp \left( \frac{\Delta }{\Delta +\varepsilon }\right) ^k\frac{\varepsilon }{\Delta +\varepsilon }, \end{aligned}$$

therefore $$H_b(E)\lesssim H_b\left( \textrm{Geom}_{\ge 0}\!\left( \frac{\varepsilon }{\Delta +\varepsilon }\right) \right) \asymp \log \left( \frac{1}{\varepsilon }\right) ^b\lesssim f\left( \frac{1}{\varepsilon }\right) ^b$$.

Note that for any *t* we have

$$\begin{aligned} H_b(Y_t|E=t) \lesssim \left( \log b(t)\right) ^b \lesssim f(t)^b. \end{aligned}$$

Using this and that *f* is increasing we get that for any fixed constant *C*

$$\begin{aligned} H_b\left( Y_E\Bigg |E\right)&=\sum _{t}\mathbb {P}\!\left( E=t\right) H_b(Y_t|E=t)\lesssim \sum _{t}\mathbb {P}\!\left( E=t\right) f(t)^b\\&\le \mathbb {P}\!\left( E\le \frac{C}{\varepsilon }\right) f\left( \frac{C}{\varepsilon }\right) ^b +\sum _{t\ge \frac{C}{\varepsilon }}\mathbb {P}\!\left( E=t\right) f(t)^b\lesssim f\left( \frac{1}{\varepsilon }\right) ^b\\&\quad + \sum _{t\ge \frac{C}{\varepsilon }}\mathbb {P}\!\left( E=t\right) f(t)^b. \end{aligned}$$

Let *C* be a sufficiently large constant as in (vii), so that $f(xy)\le f(x)f(y)$ for all $x,y\ge C$. Using this submultiplicativity and that $f(s)\lesssim s$, we get that

$$\begin{aligned}&\sum _{t\ge \frac{C}{\varepsilon }}\mathbb {P}\!\left( E=t\right) f(t)^b \lesssim \sum _{t\ge \frac{C}{\varepsilon }}\mathbb {P}\!\left( E=t\right) f\left( \frac{1}{\varepsilon }\right) ^b\left( \varepsilon t\right) ^b\\&\quad \le f\left( \frac{1}{\varepsilon }\right) ^b\mathbb {E}\!\left[ (\varepsilon E)^b\right] \asymp f\left( \frac{1}{\varepsilon }\right) ^b. \end{aligned}$$

This proves $$H_b\left( Y_E\big |E\right) \lesssim f\left( \frac{1}{\varepsilon }\right) ^b$$.

For the lower bound on $H_b\left( Y_E\big |E\right)$ we will show that for any positive constant *C* for all $t\le \frac{C}{\varepsilon }$ and all *u* we have

3.2 $$\begin{aligned} \mathbb {P}_{v}\!\Big (Y_t=u\;\Big \vert \;E=t\Big ) \asymp \mathbb {P}_{v}\!\left( Y_t=u\right) , \end{aligned}$$

hence

3.3 $$\begin{aligned} H_b(Y_t|E=t) \asymp H_b(Y_t) \asymp f(t)^b. \end{aligned}$$

For any $t\le \frac{C}{\varepsilon }$ and any *u* we have $\mathbb {P} \!\Big (Y_t=u\;\Big \vert \;E=t\Big )=\frac{\mathbb {P} \!\Big (E=t\;\Big \vert \;Y_t=u\Big )}{\mathbb {P}\!\left( E=t\right) }\mathbb {P} \!\left( Y_t=u\right)$. For any given path $u_1,...,u_t$ in *G* we have

$$\begin{aligned}\left( \frac{1}{1+\varepsilon }\right) ^{t}\frac{\varepsilon }{\Delta +\varepsilon } \le \mathbb {P} \!\Big (E=t\;\Big \vert \;Y_1=u_1,...,Y_t=u_t\Big ) \le \left( \frac{\Delta }{\Delta +\varepsilon }\right) ^{t}\frac{\varepsilon }{1+\varepsilon }.\end{aligned}$$

Here

$$\begin{aligned}0\ge \log \left( \left( \frac{1}{1+\varepsilon }\right) ^t\right) \asymp t\log \left( 1-\frac{\varepsilon }{1+\varepsilon }\right) \asymp -t\varepsilon \gtrsim -1,\end{aligned}$$

hence $\left( \frac{1}{1+\varepsilon }\right) ^{t}\frac{\varepsilon }{\Delta +\varepsilon }\asymp \varepsilon$. Similarly $\left( \frac{\Delta }{\Delta +\varepsilon }\right) ^{t}\frac{\varepsilon }{1+\varepsilon }\asymp \varepsilon$. This shows that for any *u* we have $\mathbb {P} \!\Big (E=t\;\Big \vert \;Y_t=u\Big )\asymp \varepsilon \asymp \mathbb {P}\!\left( E=t\right)$. This finishes the proof of (3.2).

Now using (3.3) we get that

$$\begin{aligned} H_b(Y_E|E)&= \sum _{t}\mathbb {P}\!\left( E=t\right) H_b(Y_t|E=t)\gtrsim \sum _{t=1}^{C/\varepsilon }\mathbb {P}\!\left( E=t\right) f(t)^b\\&\ge \mathbb {P}\!\left( \frac{c}{\varepsilon }\le E\le \frac{C}{\varepsilon }\right) f\left( \frac{c}{\varepsilon }\right) ^b \gtrsim \mathbb {P}\!\left( \frac{c}{\varepsilon }\le E\le \frac{C}{\varepsilon }\right) f\left( \frac{1}{\varepsilon }\right) ^b\\&\quad \asymp f\left( \frac{1}{\varepsilon }\right) ^b \end{aligned}$$

for sufficiently small values of the constant *c* and sufficiently large values of the constant *C*. This proves $H_b\left( Y_E\big |E\right) \gtrsim f\left( \frac{1}{\varepsilon }\right) ^b$, finishing the proof of $H_b(Y_E)\asymp f\left( \frac{1}{\varepsilon }\right) ^b$.

Now we show that all assumptions of Lemma 2.22 hold. We proved above that assumption (i) holds with $h_b\asymp f\left( \frac{1}{\varepsilon }\right) ^b$. It is immediate to see that assumption (iii) holds. Note that

$$\begin{aligned}\left( \log b(R)\right) ^b \asymp f(R)^b \lesssim f\left( \frac{1}{\varepsilon }\right) ^bf(\log g(n))^b \lesssim f\left( \frac{1}{\varepsilon }\right) ^b\left( \log g(n)\right) ^b\end{aligned}$$

and

$$\begin{aligned}\left( 1-\frac{\varepsilon }{\Delta +\varepsilon }\right) ^{\frac{R}{2}}\lesssim \exp \left( -c_1\varepsilon R\right) \lesssim \exp \left( -c_2\log g(n)\right) \ll \left( \log g(n)\right) ^{-b}\end{aligned}$$

for some positive constants $c_1$, $c_2$, hence

$$\begin{aligned}\left( \log b(R)\right) ^b\left( 1-\frac{\varepsilon }{\Delta +\varepsilon }\right) ^{\frac{R}{2}} \ll f\left( \frac{1}{\varepsilon }\right) ^b,\end{aligned}$$

so assumption (ii) also holds.

It is immediate to see that (ii) and (iii) also hold with *n* in place of *R*, hence (iv) is also satisfied. Then using Lemma 2.22, we get that in Proposition 2.20 we have $\mathfrak {h}\asymp \mathbb {E}\!\left[ Y'\right] \asymp f\left( \frac{1}{\varepsilon }\right)$, $\mathfrak {V}\asymp \mathbb {E}\!\left[ (Y')^2\right] \asymp f\left( \frac{1}{\varepsilon }\right) ^2$, $\mathbb {E}\!\left[ (Y'-\mathbb {E}\!\left[ Y'\right] )^2\right] \lesssim \mathbb {E}\!\left[ (Y')^2\right] \asymp f\left( \frac{1}{\varepsilon }\right) ^2$ and $\mathbb {E}\!\left[ (Y'-\mathbb {E}\!\left[ Y'\right] )^4\right] \lesssim \mathbb {E}\!\left[ (Y')^4\right] \asymp f\left( \frac{1}{\varepsilon }\right) ^4$. Also $b(R)\ge R\gtrsim \frac{1}{\varepsilon }$ and $\log b(R)\asymp f(R)\gtrsim f\left( \frac{1}{\varepsilon }\right) \asymp \mathfrak {h}$. Applying Proposition 2.20 gives the result. $\square$

### Values of some parameters

#### Definition 3.3

Let

$$\begin{aligned}t_0:=\frac{\log n}{\nu \mathfrak {h}}\asymp \frac{1}{\varepsilon }g(n),\qquad t_w:=\frac{1}{\nu \mathfrak {h}}\frac{\log n}{\sqrt{g(n)}}\asymp \frac{1}{\varepsilon }\sqrt{g(n)}\end{aligned}$$

where $\nu$ is the speed from Lemma 2.19 and $\mathfrak {h}$ is from Lemma 3.2 (defined for $G_n^*$, but as before, we do not include the dependence on *n* in the notation).

In what follows also let

$$\begin{aligned}  &   R:=C_R\frac{1}{\varepsilon }\log g(n),\qquad K:=C_K\log g(n),\qquad M:=C_M\log g(n),\qquad \\  &   \quad L:=\frac{1}{2}\nu (t_0+C_Lt_w) \end{aligned}$$

for some constants $C_R$, $C_K$, $C_M$ and $C_L$ to be chosen later. Let us also assume that $C_K\ge 2C_M$ as this will be needed later.

### *K*-roots and reversing paths

In this section we define *K*-roots the same way as in [18] and we define events $\Omega _0$ and $\Omega _1$ on which we will be able to consider reversals of paths. We prove some results that will mean that in the later proofs it is sufficient to only bound transition probabilities between *K*-roots, and only consider paths that can be reversed.

#### Definition 3.4

We call a vertex *x* of $G^*$ a *K*-root if $\mathcal {B}_K^*(x)$ (as in Definition 2.2) is a possible realisation of the first *K* levels of the random quasi-tree *T* corresponding to *G*. If *x* is a *K*-root and $i \le K$, we denote by $\partial \mathcal {B}^*_{i}(x)$ the collection of centres of *T*-*R*-balls at (long-range) distance *i* from *x*.

#### Lemma 3.5

There exists a positive constant *b* such that with high probability $G^*$ is such that starting a random walk from any vertex *x* with high probability we hit a *K*-root within $\frac{bK}{\varepsilon }$ steps.

#### Proof

Let *x* be any vertex of *G* and let us explore the ball $\mathcal {B}_{3K}^*(x)$ around it as follows. First let us consider the *R*-ball of *x* (i.e. the set of vertices that are within graph distance *R* from *x*). For each $v\ne x$ in this *R*-ball let us reveal the long-range edge starting from it and consider the *R*-ball around the newly revealed endpoint. If it contains any previously revealed vertex, let us say that an overlap occurred. Proceed similarly from each vertex that is in one of the new *R*-balls, but not a centre and was not already considered before. Continue the exploration for 3*K* levels.

Note that $|\mathcal {B}^*_{3K}(x)|\le b(R)^{3K+1}$, hence every time we reveal the long-range edge and the corresponding *R*-ball from a vertex *v* of $\mathcal {B}_{3K}^*(x)$, the probability that an overlap occurs is $\le \frac{b(R)^{3K+2}}{n}$. Therefore the total number *I* of overlaps is $\le _{\text {st}}\textrm{Bin}\!\left( b(R)^{3K+1},\frac{b(R)^{3K+2}}{n}\right)$. Then

$$\begin{aligned}\mathbb {P}\!\left( I\ge 2\right) \lesssim \frac{1}{n^2}b(R)^{12K+6} \ll \frac{1}{n}\end{aligned}$$

as $$\log \left( b(R)^{12K+6}\right) \asymp K\log b(R)\asymp Kf(R)\lesssim Kf\left( \frac{1}{\varepsilon }\right) f(\log g(n))\lesssim \log g(n)\frac{\log n}{g(n)}\log g(n)$$ $$\ll \log n$$.

Taking union bound over all vertices of *G* we get that whp for each *x* there is at most one overlap in the ball $\mathcal {B}^*_{3K}(x)$. If there is no overlap, then *x* is a *K*-root and we are done. Assume there is one overlap, say *y* and *z* are distinct vertices in $\mathcal {B}^*_{3K}(x)$ and $(y,y')$ and $(z,z')$ are long-range edges such that $\mathcal {B}_{G}(y',R)\cap \mathcal {B}_{G}(z',R)\ne \emptyset$. Let us consider the path from *x* to *y* that contains the fewest possible long-range edges, contains at most *R* edges of *G* in a row and does not cross the same long-range edge twice and does not pass through *z*. Let $y_0$ be the last vertex of this path that is in the *R*-ball of *x*. Let us define $z_0$ similarly. Repeating the proof of Lemma 2.4 we get that with high probability the walk from *x* will first leave the *R*-ball of *x* via a vertex other than $y_0$ and $z_0$ and will reach (long-range) distance 2*K* without returning to this *R*-ball. In this case the first vertex reached by the walk that is at distance 2*K* from *x* will be a *K*-root. Whp this vertex is reached in time $\le \frac{4K}{\nu }\asymp \frac{K}{\varepsilon }$. $\square$

#### Lemma 3.6

With high probability the number of vertices in $G^*$ that are not *K*-roots is *o*(*n*).

#### Proof

To determine whether a given vertex *x* of *G* is a *K*-root let us explore the ball $\mathcal {B}_K^*(x)$ around it as in the proof of Lemma 3.5.

Every time we reveal the long-range edge and the corresponding *R*-ball from a vertex *v* of $\mathcal {B}_K^*(x)$, the probability that an overlap occurs is $\le \frac{b(R)^{K+2}}{n}$. Therefore the total number *I* of overlaps is $\le _{\text {st}}\textrm{Bin}\!\left( b(R)^{K+1},\frac{b(R)^{K+2}}{n}\right)$. Then

$$\begin{aligned}\mathbb {P}\!\left( x\text { is not a }K\text {-root}\right) = \mathbb {P}\!\left( I\ge 1\right) \lesssim \frac{1}{n}b(R)^{2K+3} =:p.\end{aligned}$$

Similarly to the calculations in the proof of Lemma 3.5 we get that $p\ll 1$.

Let *J* be the total number of non-*K*-roots in $G^*$. Then for any constant $u>0$ we have

$$\begin{aligned}\mathbb {P}\!\left( J\ge n\sqrt{p}\right) \le \frac{\mathbb {E}\!\left[ J\right] }{n\sqrt{p}} \le \frac{np}{n\sqrt{p}} = \sqrt{p} \ll 1.\end{aligned}$$

This shows that whp the number of *K*-roots is $<n\sqrt{p}=o(n)$. $\square$

#### Lemma 3.7

For any realisation of $G^*$ and any vertex *x*, the number of vertices *y* with $\mathcal {B}_K^*(x)\cap \mathcal {B}_K^*(y)\ne \emptyset$ is *o*(*n*).

#### Proof

The number of such vertices *y* is $\le |\mathcal {B}_{2K}^*(x)|\le b(R)^{2K+1}$, which is *o*(*n*) since $\log \left( b(R)^{2K+1}\right) \lesssim Kf(R)\ll \log n$ as seen before. $\square$

Given a realisation of $G^*$, we now define the corresponding quasi-tree *T*, and given a walk *X* on $G^*$, we define the corresponding walk $\widetilde{X}$ on *T*.

#### Definition 3.8

For a vertex *v* of $G^*$ let $\eta (v)$ denote its long-range neighbour in $G^*$.

Given the graph $G^*$ and a vertex *v* in it, the associated full quasi-tree *T* and the map $\iota :T\rightarrow G^*$ are constructed as follows. Let *T* be rooted at a copy of *v* and let us add a copy of the *n*-ball of *v* in *G* around it. Let $\iota$ map each vertex in this ball to the corresponding vertex of $G^*$. For each vertex *u* in this ball (including the root) let us add a long-range edge of weight $\varepsilon$ from *u* and attach a copy of the *n*-ball of $\eta (\iota (u))$ in *G* to it. Let $\iota$ map each vertex of the new *n*-ball to the corresponding vertex in $G^*$. Repeat the same procedure from every vertex of the newly added *n*-balls except for their centres. Then proceed similarly, resulting in an infinite quasi-tree.

Given the graph $G^*$ and a vertex *v* the associated *R*-quasi-tree *T* and map $\iota _R$ are constructed analogously, but instead of *n*-balls we only consider *R*-balls and we do not add a long-range edge from the root. Note that this *R*-quasi-tree is a subgraph of the associated full quasi-tree and $\iota _R$ is a restriction of the map $\iota$ on the full quasi-tree.

Given a walk *X* on $G^*$ started from *v* we define the associated walk $\widetilde{X}$ on the associated full quasi-tree *T* as follows. Let $\widetilde{X}$ start from the root of *T*. Then $\iota (\widetilde{X}_0)=X_0$. Assume that $\iota (\widetilde{X}_k)=X_k$. If $X_{k+1}$ is a neighbour of $X_k$ in *G*, then let $\widetilde{X}_{k+1}$ be the unique vertex in the *n*-ball of $\widetilde{X}_k$ in *T* such that $\iota (\widetilde{X}_{k+1})=X_{k+1}$. Then $\widetilde{X}_{k+1}$ is also a neighbour of $\widetilde{X}_k$ in *T*. If $X_{k+1}$ is the long-range neighbour of $X_k$ in $G^*$ then let $\widetilde{X}_{k+1}$ be the long-range neighbour of $\widetilde{X}_{k}$ in *T*. Then we also have $\iota (\widetilde{X}_{k+1})=X_{k+1}$. (If $X_{k+1}$ is a neighbour of $X_k$ in *G* and is also the long-range neighbour of $X_k$, then we apply the first case with probability $\frac{1}{1+\varepsilon }$ and the second case otherwise.)

The corresponding walk on the corresponding *R*-quasi-tree is the restriction of $\widetilde{X}$ to the vertices of the *R*-quasi-tree, only defined up to the first time that $\widetilde{X}$ leaves the set of these vertices.

Given a walk *X* on $G^*$ from a vertex *v* and given a positive integer $\ell$ we define $\tau _{\ell }$ to be the first time when the corresponding walk $\widetilde{X}$ in the corresponding full quasi-tree *T* reaches level $\ell$.

Note that if *X* is a random walk on $G^*$ started from *v*, then $\widetilde{X}$ is a random walk on *T* started from the root, with $\iota (\widetilde{X}_k)=X_k$ for all *k*. Also if $\widetilde{X}$ is a random walk on *T* from the root, then $\iota (\widetilde{X}_k)$ is a random walk on $G^*$ from *v*.

We would also like to emphasise the differences of Definition 3.8 from previous similar definitions. While in Definition 2.1 we chose the new endpoint of a long-range edge uniformly at random, in Definition 3.8 we choose it as it appears in $G^*$. Also, in case of the full quasi-tree, we consider copies of the full graph *G* instead of *R*-balls. Also note that in Definition 3.8 we measure how much the walk *X* has travelled by considering the long-range distance travelled by $\widetilde{X}$. This does not coincide with the previous definition of long-range distance on $G^*$ (Definition 2.2), e.g. if *X* takes the path $(x_1,y_1,x_2,y_2,x_3,y_3,x_1)$ where $(x_i,y_i)$ are long-range edges and the sets $\{x_1,y_3\}$, $\{y_1, x_2\}$ and $\{y_2,x_3\}$ are at distance $>2R$ from each other in the graph distance of *G*, then the long-range distance in $G^*$ between the start point and endpoint is 0, while the long-range distance travelled by $\widetilde{X}$ is 3.

#### Definition 3.9

Let *X* be a walk on $G^*$ and let $\ell$ be a positive integer. Let *T* be the corresponding full quasi-tree and $\widetilde{X}$ be the corresponding walk on *T*. Let $\Omega ^{X}_0(\ell )$ be the event that up to time $\tau _{\ell }$ the walk $\widetilde{X}$ visits at most $\frac{R}{2}$ distinct vertices in each ball of *T* and that up to time $\tau _{\ell }$ the walk $\widetilde{X}$ does not cross the long-range edge from the root of *T*.

Note that if the event $\Omega ^{X}_0(\ell )$ holds then the walk corresponding to *X* on the corresponding *R*-quasi-tree is defined up to time at least $\tau _{\ell }$.

#### Lemma 3.10

Let *X* be a random walk on $G^*$ from vertex *x* and let $\ell \lesssim g(n)$ be a positive integer. Then for any sufficiently large values of $C_R$ in the definition of *R* we have

$$\begin{aligned} \mathbb {P}_{x}\!\Big (\Omega ^X_0(\ell )\;\Big \vert \;G^*\Big )= 1-o(1) \end{aligned}$$

uniformly over all possible realisations of $G^*$ and all choices of *x*.

#### Proof

Let *T* be the corresponding full quasi-tree and $\widetilde{X}$ the corresponding walk on *T*. Let *Z* be the induced walk on the directed long-range edges of *T*. We know that $\ell (Z)$ dominates a biased random walk on $\mathbb {Z}$ from 0 that steps right with probability $1-o(1)$, therefore $\mathbb {P}\!\left( \tau ^Z_\ell \ge 2\ell \right) =o(1)$. Also the probability that $\widetilde{X}$ ever crosses the long-range edge $(\rho ,\rho ^+)$ from the root is *o*(1).

At each step the probability that the walk $\widetilde{X}$ crosses a long-range edge and later never crosses that long-range edge back is $\ge \frac{\varepsilon }{\Delta +\varepsilon }(1-\delta )=:\varepsilon '\ge \frac{\varepsilon }{2\Delta }$, therefore

$$\begin{aligned}  &   1-\mathbb {P}_{x}\!\Big (\Omega ^X_0(\ell )\;\Big \vert \;G^*\Big )\le \mathbb {P} \!\Big (\tau ^{\widetilde{X}}_{\rho ^+}<\infty \;\Big \vert \;G^*\Big )+\mathbb {P} \!\Big (\tau ^Z_\ell \ge 2\ell \;\Big \vert \;G^*\Big )\\  &   \quad +2\ell \cdot \mathbb {P}\!\left( \textrm{Geom}_{\ge 0}\!\left( \varepsilon '\right) \ge \frac{1}{2}R\right) . \end{aligned}$$

We already know that $\mathbb {P} \!\Big (\tau ^{\widetilde{X}}_{\rho ^+}<\infty \;\Big \vert \;G^*\Big )=o(1)$ and $\mathbb {P} \!\Big (\tau ^Z_\ell \ge 2\ell \;\Big \vert \;G^*\Big )=o(1)$. We have

$$\begin{aligned} \log \mathbb {P}\!\left( \textrm{Geom}_{\ge 0}\!\left( \varepsilon '\right) \ge \frac{1}{2}R\right) = \frac{1}{2}R\log \left( 1-\varepsilon '\right) \le -\frac{1}{4\Delta }R\varepsilon \le -\frac{C_R}{4\Delta }\log g(n). \end{aligned}$$

This gives

$$\begin{aligned} 2\ell \cdot \mathbb {P}\!\left( \textrm{Geom}_{\ge 0}\!\left( \varepsilon '\right) \ge \frac{1}{2}R\right) \lesssim g(n)^{1-C_R/(4\Delta )} \end{aligned}$$

which is *o*(1) for sufficiently large values of $C_R$. $\square$

#### Definition 3.11

Let *X* be a walk on $G^*$ and let $\ell$ be a positive integer. Let *T* be the corresponding full quasi-tree and let $\widetilde{X}$ be the corresponding walk on *T*. Let $\Omega ^{X}_1(\ell )$ be the event that the following is satisfied. For $k=1,2,...,\lfloor \frac{\ell }{2}\rfloor$ up to time $\tau _\ell$ the walk $\widetilde{X}$ only visits level $2k-1$ of *T* at descendants of $\widetilde{\xi }^{(\ell )}_k$, where $\widetilde{\xi }^{(\ell )}$ denotes the loop-erasure of $(\widetilde{X}_i)_{i=0}^{\tau _\ell }$.

#### Lemma 3.12

Let *X* be a random walk on $G^*$ from vertex *x* and let $\ell \lesssim g(n)$ be a positive integer. Then we have

$$\begin{aligned} \mathbb {P}_{x}\!\Big (\Omega ^X_1(\ell )\;\Big \vert \;G^*\Big )= 1-o(1) \end{aligned}$$

uniformly over all possible realisations of $G^*$ and all choices of *x*.

#### Proof

Let *T* be the corresponding full quasi-tree and let $\widetilde{X}$ be the corresponding walk on it. Let $\Omega '_1$ be the event that for $k=1,2,...$ after hitting level $2k-1$ the walk $\widetilde{X}$ never returns to level $k-1$. Note that if $\Omega '_1$ holds, then $\Omega _1(\ell )$ holds for all $\ell$. Also note that by Lemma 2.4 the probability that $\widetilde{X}$ ever returns to a level *k* after hitting level $k+m$ is $\delta ^m$. Hence

$$\begin{aligned} 1-\mathbb {P}_{x}\!\Big (\Omega ^X_1(\ell )\;\Big \vert \;G^*\Big )\le 1-\mathbb {P}_{x}\!\Big (\Omega '_1\;\Big \vert \;G^*\Big )\le \sum _{k=1}^{\infty }\delta ^k \lesssim \delta = o(1). \end{aligned}$$

$$\square $$

For a finite path $\gamma$ in $G^*$ we define $\Omega _0^\gamma (\ell )$ and $\Omega _1^\gamma (\ell )$ analogously to Definitions 3.9 and 3.11. (If the path $\widetilde{\gamma }$ on the corresponding full quasi-tree *T* does not reach distance $\ell$, then these events are not defined.)

#### Lemma 3.13

Let $x=(x_0,\ldots , x_{k})$ be a path in $G^*$ such that $k=\tau ^x_{\ell }$ and $\Omega ^x_0(\ell )$ and $\Omega ^x_1(\ell )$ hold. Define a path *z* by setting $z_0=\eta (x_k)=x_{k-1}$, $z_1=x_{k-2}$, $z_2=x_{k-3}$,..., $z_{k-1}=x_0$, $z_k=\eta (x_0)$. Then we have $k=\tau ^z_{\ell }$, and the events $\Omega ^z_0(\ell )$ and $\Omega ^z_1(\ell )$ hold.

#### Proof

Let *T* be the full quasi-tree corresponding to $G^*$ and $x_0$, let $\iota$ be the corresponding map and let *y* be the path on *T* corresponding to *x*. Let $y_{-1}$ be the long-range neighbour of $y_0$. Note that rerooting *T* at $y_{k-1}$ it becomes a full quasi-tree corresponding to $G^*$ and $z_{0}$, and $(y_{k-1-i})$ is the path on it corresponding to *z*.

The condition that $k=\tau ^z_{\ell }$, $\Omega ^z_0(\ell )$ and $\Omega ^z_1(\ell )$ hold, and the condition that $k=\tau ^x_{\ell }$, $\Omega ^x_0(\ell )$ and $\Omega ^x_1(\ell )$ hold are both equivalent to the following points being satisfied.

- The vertex $y_k$ is at long-range distance $(\ell +1)$ from $\eta (y_0)$.
- The path $\eta (y_0),y_0,y_1,...,y_k$ has at most $\frac{R}{2}$ distinct vertices in each ball of *T*.
- Let $B_{-1}$, $B_0$, $B_1$,..., $B_\ell$ (in this order) be the balls in *T* visited by the loop-erasure of the path $\eta (y_0),y_0,y_1,...,y_k$. For a ball *B* in *T* let $i_B={{\,\textrm{argmin}\,}}_{i\in \{-1,0,...,\ell \}}d_T(B,B_i)$, i.e. the index of the ball $B_i$ at minimal long-range distance from *B*. (This is well-defined because of the tree structure of *T*.) The path $y_0,y_1,...,y_{k-1}$ never visits a ball *B* with $i_B+d_T(B,B_{i_B})>\ell$ or $(\ell -i_B)+d_T(B,B_{i_B})>\ell$. (Note that this is equivalent to saying that $y_0,y_1,...,y_{k-1}$ never visits a ball *B* that is at long-range distance $>\ell$ from $B_{-1}$ or at long-range distance $>\ell$ from $B_{\ell }$.) See Fig. 3 for an illustration.

**Fig. 4:**
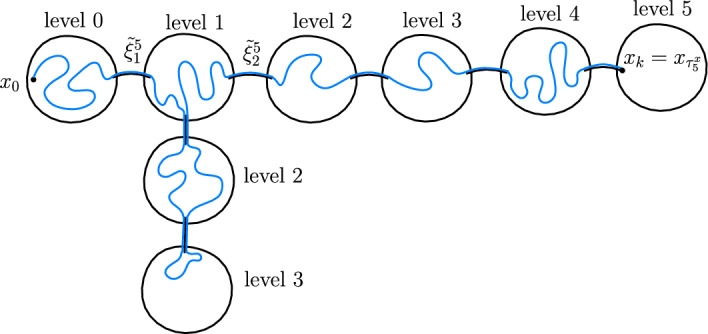
In this example $\Omega ^x_1(5)$ does not hold, since *x* reaches (long-range) distance 3 from $x_0$ at an *R*-ball that is not a descendant of $\widetilde{\xi }^{5}_2$

**Fig. 5:**
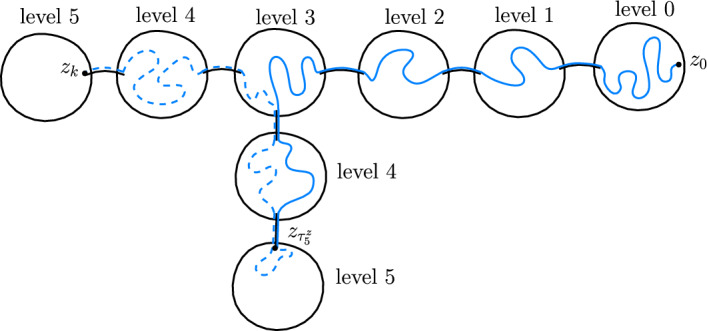
Here $\tau ^z_{\ell }\ne k$, since the reversed path *z* reaches distance 5 from $z_0$ before time *k*

Also see Figs. 4 and 5 for an example where $\Omega _1^x(\ell )$ does not hold and $\tau ^z_{\ell }\ne k$. $\square$

On the high probability event $\Omega _0\cap \Omega _1$, we can make an approximation similar to (1.2), and hence obtain an upper bound on the distance of $X_{\tau _{2\ell }}$ from the uniform distribution as follows.

#### Lemma 3.14

Let $\mu$ be a distribution on the vertices with $\mu (z)\asymp \frac{1}{n}$ $\forall z$ and let $\ell \lesssim g(n)$ be a positive integer. Then for any realisation of $G^*$ and any vertices *x* and *y* we have

$$\begin{aligned}&d_{\textrm{TV}}\left( {\mathbb {P}_{x}\!\Big (X_{\tau _{2\ell }}\in \cdot \;\Big \vert \;G^*\Big )},{\mu (\cdot )}\right) \\&\quad \le \sum _y\left( \mu (y)-\sum _{z}\mathbb {P}_{x}\!\Big (X_{\tau _{\ell }}=z,\Omega _0(\ell ),\Omega _1(\ell )\;\Big \vert \;G^*\Big )\right. \\&\qquad \left. \mathbb {P}_{\eta (y)}\!\Big (X_{\tau _{\ell }}=\eta (z),\Omega _0(\ell ),\Omega _1(\ell )\;\Big \vert \;G^*\Big )\right) ^++o(1). \end{aligned}$$

#### Proof

In what follows we consider $G^*$ fixed and omit it from the notation.

For any *x* and *y* we have

$$\begin{aligned}&\mathbb {P}_{x}\!\left( X_{\tau _{2\ell }}=y\right) \\&\quad \ge \quad \sum _{z}\mathbb {P}_{x}\!\left( X_{\tau _{\ell }}=z,\Omega _0(\ell ),\Omega _1(\ell )\right) \mathbb {P}_{z}\!\left( X_{\tau _{1}}\ne \eta (z),X_{\tau _{\ell }}=y,\Omega _0(\ell ),\Omega _1(\ell )\right) \\&\quad = \sum _{z}\mathbb {P}_{x}\!\left( X_{\tau _{\ell }}=z,\Omega _0(\ell ),\Omega _1(\ell )\right) \mathbb {P}_{z}\!\left( X_{\tau _{\ell }}=y,\Omega _0(\ell ),\Omega _1(\ell )\right) \\  &\qquad - \sum _z\mathbb {P}_{x}\!\left( X_{\tau _{\ell }}=z,\Omega _0(\ell ),\Omega _1(\ell )\right) \mathbb {P}_{z}\!\left( X_{\tau _{1}}=\eta (z),X_{\tau _{\ell }}=y,\Omega _0(\ell ),\Omega _1(\ell )\right) \end{aligned}$$

Note that by Lemma 3.13 we have that

$$\begin{aligned} \mathbb {P}_{z}\!\left( X_{\tau _{\ell }}=y,\Omega _0(\ell ),\Omega _1(\ell )\right) = \mathbb {P}_{\eta (y)}\!\left( X_{\tau _{\ell }}=\eta (z),\Omega _0(\ell ),\Omega _1(\ell )\right) . \end{aligned}$$

Using the definition of the events $\Omega _0$ and $\Omega _1$ we also see that

$$\begin{aligned}  &   \mathbb {P}_{z}\!\left( X_{\tau _{1}}=\eta (z),X_{\tau _{\ell }}=y,\Omega _0(\ell ),\Omega _1(\ell )\right) \\  &   \quad \le \mathbb {P}_{z}\!\left( X_{\tau _{1}}=\eta (z)\right) \mathbb {P}_{\eta (z)}\!\left( X_{\tau _{\ell -1}}=y,\Omega _0(\ell -1),\Omega _1(\ell -1)\right) . \end{aligned}$$

Using this we then obtain

$$\begin{aligned}&\sum _{y,z}\mathbb {P}_{x}\!\left( X_{\tau _{\ell }}=z,\Omega _0(\ell ),\Omega _1(\ell )\right) \mathbb {P}_{z}\!\left( X_{\tau _{1}}=\eta (z),X_{\tau _{\ell }}=y,\Omega _0(\ell ),\Omega _1(\ell )\right) \\&\quad \le \sum _{y,z}\mathbb {P}_{x}\!\left( X_{\tau _{\ell }}=z,\Omega _0(\ell ),\Omega _1(\ell )\right) \mathbb {P}_{z}\!\left( X_{\tau _{1}}=\eta (z)\right) \\&\qquad \mathbb {P}_{\eta (z)}\!\left( X_{\tau _{\ell -1}}=y,\Omega _0(\ell -1),\Omega _1(\ell -1)\right) \\&\quad \le \sum _{y,z}\mathbb {P}_{x}\!\left( X_{\tau _{\ell }}=z,\Omega _0(\ell ),\Omega _1(\ell )\right) \cdot \delta \cdot \mathbb {P}_{\eta (z)}\!\left( X_{\tau _{\ell -1}}=y,\Omega _0(\ell -1),\Omega _1(\ell -1)\right) \\&\quad \le \delta \sum _{z}\mathbb {P}_{x}\!\left( X_{\tau _{\ell }}=z,\Omega _0(\ell ),\Omega _1(\ell )\right) \le \delta = o(1). \end{aligned}$$

Altogether these give

$$\begin{aligned}&d_{\textrm{TV}}\left( {\mathbb {P}_{x}\!\left( X_{\tau _{2\ell }}\in \cdot \right) },{\mu (\cdot )}\right) = \sum _y\left( \mu (y)-\mathbb {P}_{x}\!\left( X_{\tau _{2\ell }}=y\right) \right) ^+\\&\quad \le \sum _y\left( \mu (y)-\sum _{z}\mathbb {P}_{x}\!\left( X_{\tau _{\ell }}=z,\Omega _0(\ell ),\Omega _1(\ell )\right) \mathbb {P}_{\eta (y)}\!\left( X_{\tau _{\ell }}=\eta (z),\Omega _0(\ell ),\Omega _1(\ell )\right) \right) ^++o(1). \end{aligned}$$

This now finishes the proof. $\square$

In what follows we will work towards finding a lower bound on

$$\sum _{z}\mathbb {P}_{x}\!\left( X_{\tau _{\ell }}=z,\Omega _0(\ell ),\Omega _1(\ell )\right) \mathbb {P}_{\eta (y)}\!\left( X_{\tau _{\ell }}=\eta (z),\Omega _0(\ell ),\Omega _1(\ell )\right) $$

for most values of *y* and showing that whp the contribution from the remaining vertices *y* is *o*(1). This will in turn give an upper bound on the total variation distance from Lemma 3.14 and then in Sect. 4 we will use a relationship between mixing times and hitting times to get an upper bound on the mixing time.

### Truncation

#### Definition 3.15

For a quasi-tree *T* and a long-range edge *e* let

$$\begin{aligned} \theta _T(e):=\mathbb {P}_{\rho }\!\Big (e\in \xi \;\Big \vert \;p(e)\in \xi \Big ) \end{aligned}$$

where $\xi$ is a loop-erased random walk and *p*(*e*) is the "parent" of *e*, i.e. the first long-range edge on the shortest path from *e* to $\rho$. (If *e* is between levels 0 and 1, then *p*(*e*) is not defined and $\theta _T(e)=\mathbb {P}_{\rho }\!\left( e\in \xi \right)$.) For a long-range edge *e* at level 1 we let

$$\begin{aligned} \widetilde{\theta }_{T,M}(e):=\mathbb {P}_{\rho }\!\left( X_{\tau _M}\in V_e\right) \end{aligned}$$

where *X* is a simple random walk on *T*, *M* is as in Definition 3.3, $\tau _M$ is the first time *X* hits level *M* and $V_e$ is the set of descendants of *e*.

Note that

$$\begin{aligned} W_T(e):=-\log \mathbb {P} \!\Big (e\in \xi \;\Big \vert \;T\Big )= -\sum _{i=1}^{\ell (e)}\log \theta _{T(y_{i-1})}(e_i) \end{aligned}$$

where we recall that $\ell (e)$ is the level of *e* and $e_1,...,e_{\ell (e)}$ are the long-range edges from $\rho$ to *e*, $e_i=(x_i,y_i)$, $y_0=\rho$, and $T(y_{i-1})$ is the quasi-tree formed by the ball of $y_{i-1}$ and the balls descending from it as in Definition 2.10.

Also, define

$$\begin{aligned} \widetilde{W}_T(e):=-\sum _{i=1}^{\ell (e)}\log \widetilde{\theta }_{T(y_{i-1}),M}(e_i).\end{aligned}$$

#### Definition 3.16

For a long-range edge *e*, constant $A>0$ and given positive integer *K* define the truncation event as

$$\begin{aligned} \textrm{Tr}(e,A): = \left\{ \widetilde{W}_T(e) > \frac{1}{2}\log n + A\sqrt{\frac{\mathfrak {V}\log n}{\mathfrak {h}}}\right\} \cap \{\ell (e)\ge K\}, \end{aligned}$$

where $\mathfrak {V}$ is from Lemma 3.2.

This is similar to the truncation criterion used in [6]. Roughly speaking it indicates that the long-range edge *e* is unlikely to be crossed by a random walk on *T*, but we use $\widetilde{W}$ so that the event only depends on the levels of *T* up to the level $\ell (e)+M$, not the whole of *T*. In the following lemma we show that $\textrm{Tr}(e,A)$ can be compared to a similar event with *W* instead.

Note that $\sqrt{\frac{\mathfrak {V}\log n}{\mathfrak {h}}}\asymp \frac{\log n}{\sqrt{g(n)}}$ and we know that $\frac{\log n}{g(n)}\asymp f\left( \frac{1}{\varepsilon }\right) \gtrsim \log \left( \frac{1}{\varepsilon }\right) \gtrsim \log \left( (\log n)^{\beta }\right) \asymp \log \log n$, hence $g(n)\lesssim \frac{\log n}{\log \log n}$, therefore $\sqrt{\frac{\mathfrak {V}\log n}{\mathfrak {h}}}\gtrsim \sqrt{(\log \log n)\log n}$. We will use these multiple times in the following proofs.

#### Lemma 3.17

Let *G* be as before, assume that Assumption 3.1 holds and let *R*, *K*, *M* and *L* be as in Definition 3.3. Let *T* be the random quasi-tree associated to *G*.

Then for any positive constants *A* and $A'<A$ the following holds for any sufficiently large *n*. For any realisation of *T* and for any long-range edge *e* of *T* at level *L*

3.4 $$\begin{aligned} \text {if}\qquad&\widetilde{W}_T(e)\ge \frac{1}{2}\log n+A\sqrt{\frac{\mathfrak {V}\log n}{\mathfrak {h}}}, \end{aligned}$$

3.5 $$\begin{aligned} \text {then}\qquad&{W}_T(e)\ge \frac{1}{2}\log n+A'\sqrt{\frac{\mathfrak {V}\log n}{\mathfrak {h}}}. \end{aligned}$$

#### Proof

The subscript *T* will be omitted. By replacing *A* with a constant in $(A',A)$, we see that it is sufficient to show the result with > instead of $\ge$ in (3.4). So we wish to show that for any *e* at level *L* we have

$$\begin{aligned} W(e)\ge \frac{1}{2}\log n+A'\sqrt{\frac{\mathfrak {V}\log n}{\mathfrak {h}}}\qquad \text {or} \qquad \widetilde{W}(e)\le \frac{1}{2}\log n+A\sqrt{\frac{\mathfrak {V}\log n}{\mathfrak {h}}}. \end{aligned}$$

Equivalently we wish to show that

$$\begin{aligned}  &   \prod _{i=1}^L\theta _{T(y_{i-1})}(e_i)\le \exp \left( -\frac{1}{2}\log n-A'\sqrt{\frac{\mathfrak {V}\log n}{\mathfrak {h}}}\right) \\  &   \quad \text {or}\quad \prod _{i=1}^L\widetilde{\theta }_{T(y_{i-1}),M}(e_i)\ge \exp \left( -\frac{1}{2}\log n-A\sqrt{\frac{\mathfrak {V}\log n}{\mathfrak {h}}}\right) . \end{aligned}$$

Since $\theta _{T(y_{i-1})}(e_i)\le 1$ for each *i*, it is sufficient to show that for any $A'<A$ we have

$$\begin{aligned}&\theta _{T(y_{i-1})}(e_i)\le \exp \left( -\frac{1}{2}\log n-A'\sqrt{\frac{\mathfrak {V}\log n}{\mathfrak {h}}}\right) \quad \text {for some }i\quad \text {or}\\&\quad \prod _{i=1}^L\widetilde{\theta }_{T(y_{i-1}),M}(e_i)\ge \prod _{i=1}^L\theta _{T(y_{i-1})}(e_i)\exp \left( -(A-A')\sqrt{\frac{\mathfrak {V}\log n}{\mathfrak {h}}}\right) . \end{aligned}$$

It is sufficient to show that for any edge *e* at level 1 and any $A'<A$ we have

$$\begin{aligned}  &   \theta _{T}(e)\le \exp \left( -\frac{1}{2}\log n-A'\sqrt{\frac{\mathfrak {V}\log n}{\mathfrak {h}}}\right) \quad \text {or} \quad \widetilde{\theta }_{T,M}(e)\ge \theta _{T}(e)\\  &   \quad \exp \left( -\frac{A-A'}{L}\sqrt{\frac{\mathfrak {V}\log n}{\mathfrak {h}}}\right) . \end{aligned}$$

Equivalently, we wish to show that for any long-range edge *e* at level 1 and any $A'<A$ we have the following.

3.6 $$\begin{aligned}&\text {If}\qquad \mathbb {P}_{\rho }\!\left( e\in \xi \right) \ge \exp \left( -\frac{1}{2}\log n-A'\sqrt{\frac{\mathfrak {V}\log n}{\mathfrak {h}}}\right) , \end{aligned}$$

3.7 $$\begin{aligned}&\text {then}\qquad \mathbb {P}_{\rho }\!\left( X_{\tau _M}\in V_e\right) \ge \mathbb {P}_{\rho }\!\left( e\in \xi \right) \exp \left( -\frac{A-A'}{L}\sqrt{\frac{\mathfrak {V}\log n}{\mathfrak {h}}}\right) . \end{aligned}$$

In the rest of the proof let us assume that (3.6) holds. We start by noting that

$$\begin{aligned} \mathbb {P}_{\rho }\!\left( e\in \xi \right)&= \mathbb {P}_{\rho }\!\left( e\in \xi ,X_{\tau _M}\in V_e\right) +\mathbb {P}_{\rho }\!\left( e\in \xi ,X_{\tau _M}\not \in V_e\right) \\&\le \mathbb {P}_{\rho }\!\left( X_{\tau _M}\in V_e\right) +\mathbb {P}_{\rho }\!\left( e\in \xi ,X_{\tau _M}\not \in V_e\right) . \end{aligned}$$

Now our goal is to show that

$$\begin{aligned} \mathbb {P}_{\rho }\!\left( e\in \xi ,X_{\tau _M}\not \in V_e\right) \le \left( 1-\exp \left( -\frac{A-A'}{L}\sqrt{\frac{\mathfrak {V}\log n}{\mathfrak {h}}}\right) \right) \mathbb {P}_{\rho }\!\left( e\in \xi \right) . \end{aligned}$$

Note that for any constants *A* and $A'<A$, using that $\mathfrak {h}\asymp f\left( \frac{1}{\varepsilon }\right)$ and $\mathfrak {V}\asymp f\left( \frac{1}{\varepsilon }\right) ^2$ from Lemma 3.2 and the value of *L* from Definition 3.3 we get $\frac{A-A'}{L}\sqrt{\frac{\mathfrak {V}\log n}{\mathfrak {h}}}\asymp \frac{\log n}{g(n)^{3/2}}$, and hence $1-\exp \left( -\frac{A-A'}{L}\sqrt{\frac{\mathfrak {V}\log n}{\mathfrak {h}}}\right) \ge \frac{1}{2}\wedge \frac{c_3\log n}{g(n)^{3/2}}$ for some constant $c_3$ and all sufficiently large *n*.

Note that

$$\begin{aligned} \mathbb {P}_{\rho }\!\left( e\in \xi ,X_{\tau _M}\not \in V_e\right) \le \mathbb {P}_{\rho }\!\left( \text {hit level }M\text { and then hit }e^+\right) . \end{aligned}$$

By Lemma 2.18 we know that for any $\ell _0$ this is

3.8 $$\begin{aligned} \lesssim \ell _0\delta ^M\mathbb {P}_{\rho }\!\left( \tau _{e^+}<\infty \right) +\delta ^{M}e^{-c'\varepsilon \ell _0}\left( \ell _0+\frac{1}{\varepsilon }\right) , \end{aligned}$$

where $\delta$ is as in Lemma 2.4.

We know that $\mathbb {P}_{\rho }\!\left( \tau _{e^+}<\infty \right) \asymp \mathbb {P}_{\rho }\!\left( e\in \xi \right) \ge \exp \left( -c_4\log n\right)$ for some positive constant $c_4$. Let us choose $\ell _0=\frac{c_4\log n}{c'\varepsilon }$. Then we get that (3.8) is

$$\begin{aligned} \lesssim \frac{\log n}{\varepsilon }\delta ^M\mathbb {P}_{\rho }\!\left( e\in \xi \right) . \end{aligned}$$

We have

$$\begin{aligned} \delta ^{\frac{M}{2}}= \exp \left( -\frac{C_M}{2}\left( \log g(n)\right) \log \left( \frac{1}{\delta }\right) \right) \lesssim \exp \left( -\frac{3}{2}\log g(n)\right) =\frac{1}{g(n)^{3/2}}, \end{aligned}$$

and hence using that $\delta \lesssim \varepsilon ^{1/3}$ from Lemma 2.4 we get that

$$\begin{aligned} \frac{\log n}{\varepsilon }\delta ^M \ll \frac{\log n}{g(n)^{3/2}}, \end{aligned}$$

and also

$$\begin{aligned} \frac{\log n}{\varepsilon }\delta ^M \lesssim \exp \left( \log \log n-\frac{C_M}{2}\left( \log g(n)\right) \log \left( \frac{1}{\delta }\right) \right) \ll 1. \end{aligned}$$

For the last step above we used that $\log (1/\delta )\asymp \log (1/\varepsilon )$ and the assumption that $\varepsilon \lesssim (\log n)^{-\beta }$.

Together these show that for all sufficiently large *n* we have

$$\begin{aligned} \mathbb {P}_{\rho }\!\left( e\in \xi ,X_{\tau _M}\not \in V_e\right) \le \left( 1-\exp \left( -\frac{A'-A}{L}\sqrt{\frac{\mathfrak {V}\log n}{\mathfrak {h}}}\right) \right) \mathbb {P}_{\rho }\!\left( e\in \xi \right) \end{aligned}$$

which finishes the proof. $\square$

#### Proposition 3.18

Let us consider the setup of Lemma 3.17. Let $T_0$ be any realisation of the first *K* levels of *T*. Let *X* be a simple random walk on *T* started from its root. Then for all $\theta \in (0,1)$ there exist $C_L$ (in the definition of *L* in Definition 3.3, depending on $\theta$) and *A* (depending on $\theta$ and $C_L$) sufficiently large such that

$$\begin{aligned} \mathbb {P} \!\Big (\bigcup _{k\le \tau _{L}}\textrm{Tr}((X_{k-1},X_k),A)\;\Big \vert \;B_K(\rho )=T_0\Big )<\theta . \end{aligned}$$

#### Proof

The proof is analogous to the proof of [18, Lemma 4.5] and we omit the details.

#### Definition 3.19

Given a quasi-tree *T* and *L* as in Definition 3.3, we define the level *L* truncation event for a long-range edge *e* as

$$\begin{aligned}\textrm{Tr}'(e):=\left\{ \ell (e)=L,\quad -\log \mathbb {P}_{\rho }\!\left( X_{\tau _L}=e^+\right) <\frac{1}{2}\log n+\log \log n\right\} .\end{aligned}$$

#### Proposition 3.20

Let us consider the setup of Lemma 3.17. Let $T_0$ be a realisation of the first *K* levels of *T*. Let *X* be a simple random walk on *T* started from its root. Then for all $\theta \in (0,1)$ there exist $C_L$ (as in Definition 3.3, depending on $\theta$) sufficiently large such that

$$\begin{aligned} \mathbb {P} \!\Big (\textrm{Tr}'(\left( X_{\tau _{L}-1},X_{\tau _{L}}\right) )\;\Big \vert \;B_K(\rho )=T_0\Big )<\theta . \end{aligned}$$

#### Proof

For any long-range edge *e* we have

$$\begin{aligned} \mathbb {P} \!\Big (e\in \xi \;\Big \vert \;T\Big )\ge (1-o(1))\mathbb {P} \!\Big (\left( X_{\tau _{\ell (e)}-1},X_{\tau _{\ell (e)}}\right) =e\;\Big \vert \;T\Big ), \end{aligned}$$

hence

$$\begin{aligned} \mathbb {P} \!\Big (\textrm{Tr}'(\xi _L)\;\Big \vert \;B_K(\rho )=T_0\Big ) \ge (1-o(1))\mathbb {P} \!\Big (\textrm{Tr}'(\left( X_{\tau _{L}-1},X_{\tau _{L}}\right) )\;\Big \vert \;B_K(\rho )=T_0\Big ) \end{aligned}$$

and $$\textrm{Tr}'(\xi _L)$$ implies $${W}_T(\xi _{L})<\frac{1}{2}\log n+\log \log n+o(1)$$.

From Lemma 3.2 we know that for a sufficiently large value of *C* we have

$$\begin{aligned} \mathbb {P} \!\Big (W_T(\xi _L)\le L\mathfrak {h}-C\sqrt{L\mathfrak {V}}\;\Big \vert \;T_0\Big )<\theta . \end{aligned}$$

Note that

$$\begin{aligned} L\mathfrak {h}-C\sqrt{L\mathfrak {V}}= \frac{1}{2}\log n+\frac{1}{2}C_L\frac{\log n}{\sqrt{g(n)}}-Ca\sqrt{\frac{(\log n)^2}{g(n)}+B\frac{(\log n)^2}{\sqrt{g(n)}}} \end{aligned}$$

where $a\asymp 1$. For sufficiently large values of $C_L$ (in terms of *C*) this is $\ge \frac{1}{2}\log n+\frac{1}{4}C_L\frac{\log n}{\sqrt{g(n)}}>\frac{1}{2}\log n+\log \log n+1$.

Putting these together gives the result. $\square$

### Coupling

#### Definition 3.21

Let *x* and *y* be two distinct vertices of the graph *G* and condition on both of them being *K*-roots in $G^*$ with disjoint *K*-neighbourhoods $T_{x,0}=\mathcal {B}_K^*(x)$ and $T_{y,0}=\mathcal {B}_K^*(y)$.

Let $z_1,...,z_{L_x}$ be the vertices of $\partial \mathcal {B}_{K/2}^*(x)$ and let $z_{L_x+1},...,z_{L_x+L_y}$ be the vertices of $\partial \mathcal {B}_{K/2}^*(y)$. For a $z_i$ in $\partial \mathcal {B}_{K/2}^*(x)$ let $V_{z_i}$ be the set of its descendants in $\partial \mathcal {B}_{K}^*(x)$.

For each $z_i$ we will define an exploration process of $G^*$ from $V_{z_i}$ by constructing a coupling between a subset of $G^*$ and two independent quasi-trees $T_x$, $T_y$ that are distributed like the random quasi-tree corresponding to the graph *G*, conditioned to be $T_{x,0}$ and $T_{y,0}$ respectively at the first *K* levels around their roots.

Let us assume that $i\le L_x$. (The exploration from $V_{z_i}$ with $L_x<i\le L_x+L_y$ is defined analogously.)

First let us reveal one by one all the long-range edges of $T_x$ which have one endpoint in the *R*-ball of some $w\in V_{z_i}$, but not in $V_{z_i}$ itself. Let us couple each of these long-range edges with the corresponding long-range edge of $G^*$ by using the optimal coupling between the two uniform distributions at every step. (At each step the endpoint in $T_x$ is chosen uniformly from all vertices, while the endpoint in $G^*$ is chosen uniformly from all vertices whose long-range edge has not been revealed yet.) If one of these couplings fails, let us truncate that edge and stop the exploration from this edge in $G^*$, but continue in $T_x$. If the coupling was successful for a given edge, let us also consider the *R*-ball around its newly revealed endpoint. Let us also truncate a long-range edge if this *R*-ball intersects the *R*-ball around any previously considered vertex. Once all long-range edges from the *R*-balls of $V_{z_i}$ are revealed, examine for each of them whether its level *M* ancestor satisfies the truncation criterion $\textrm{Tr}(e,A)$ (which is defined w.r.t. $T_x$, not $G^*$; note that since $M\le \frac{K}{2}$, it only depends on edges we have already revealed). If the level *M* ancestor satisfies the truncation criterion, then let us truncate that edge and stop the exploration from it in $G^*$.

Suppose we have already explored the long-range edges up to level *k* of $T_x$ and for each edge that is a descendant of ${z_i}$ and neither the edge nor any of its ancestors have been truncated, we have also explored the corresponding edge in $G^*$. Then let us reveal the edges between levels *k* and $k+1$ in $T_x$ and for each edge that is a descendant of $z_i$ and whose ancestors and itself are not truncated, let us couple the corresponding edge of $G^*$ to it using the optimal coupling. If the optimal coupling fails or the *R*-ball around the newly added endpoint intersects any previously revealed *R*-ball or if the level *M* ancestor of the edge satisfies the truncation criterion $\textrm{Tr}(e,A)$, then let us truncate it and stop the exploration from it in $G^*$. Let us continue this up to level $L-1$. Let us also consider the half-edges leading to level *L*, but do not reveal their level *L* endpoints yet. For each such half-edge let us truncate it if its level *M* ancestor satisfies the truncation criterion $\textrm{Tr}(e,A)$ or the half-edge itself satisfies $\textrm{Tr}'(e)$ (note that we do not need the other endpoint to determine whether this holds).

Let us run the exploration processes from $V_{z_i}$ for $i=1,2,...,L_x+L_y$ in this order. Let $\mathcal {F}_i$ be the $\sigma$-algebra generated by $T_{x,0}$, $T_{y,0}$ and the exploration processes started from $V_{z_1},...V_{z_i}$. Say that the vertex $z_i$ is *good* if no vertex from the *R*-balls of $V_{z_i}$ has been explored during the exploration processes corresponding to the sets $V_{z_1},...V_{z_{i-1}}$. Otherwise call $z_i$ *bad*. Note that the event $\{z_i\text { good}\}$ is $\mathcal {F}_{i-1}$-measurable.

Let $\partial T_x$ denote the set of vertices at level $L-1$ of $T_x$ that are the endpoints of non-truncated long-range half-edges leading to level *L*. Define $\partial T_y$ analogously.

Note that by construction $G^*$ agrees with $T_x$ and $T_y$ on the (random) regions around *x* and *y* enclosed by the truncated edges and the half-edges leading from levels $L-1$ to levels *L*.

After running the explorations from each $V_{z_i}$ let $\mathcal {I}$ be the set of yet unpaired long-range half-edges in $G^*$. Let us complete the exploration of $G^*$ by considering a uniform random matching of $\mathcal {I}$ and adding the corresponding long-range edges.

For $u\in V_{z_i}$ where $1\le i\le L_x$, given the explorations from all $V_{z_k}$ let us define the coupling of a random walk *X* on $G^*$ from *u* with a random walk $\widetilde{X}$ on $T_x$ from *u* as follows. Let us run $\widetilde{X}$ until it reaches level *L* in $T_x$ and move *X* together with it as long as the following hold.

(i) $\widetilde{X}$ does not cross a truncated edge;
(ii) $\widetilde{X}$ does not visit level $K-1$ of $T_x$;
(iii) $$\Omega ^X_0(L-K)$$ holds.

After the first time that (i), (ii) or (iii) fails to hold, let us move *X* independently from $\widetilde{X}$.

If (i), (ii) and (iii) hold, and also

(iv) $$\Omega ^X_1(L-K)$$ holds,

then we say that the coupling is successful. Otherwise we say that the coupling fails.

We can also define the coupling of a random walk *X* on $G^*$ from *x* and a random walk $\widetilde{X}$ on $T_x$ from *x* as follows. Let us run $\widetilde{X}$ until it hits level *K* and move *X* together with it. After that use the above coupling from the level *K* vertices. We say that the coupling is successful if $\Omega _0^X(K)$ and $\Omega _1^X(K)$ both hold (note that these only depend on $\widetilde{X}$ up to the first time it hits level *K*) and the coupling from the level *K* vertices is also successful. Otherwise let us say that the coupling fails. Let $\Omega _2^X$ denote the event that the coupling is successful. Note that on this event $\Omega _0^X(L)$ and $\Omega _1^X(L)$ also hold.

For $v\in V_{z_j}$ where $L_x<j\le L_x+L_y$ let us define the coupling of random walks on $G^*$ and $T_y$ from *v* analogously. Also define the coupling of random walks on $G^*$ and $T_y$ from *y* and the event $\Omega _2^Y$ analogously.

For ease of notation we will continue writing $\mathbb {P}\!\left( \cdot \right)$ for the measure of the coupling. We will also write $\mathbb {P}_{x}\!\left( \cdot \right)$ or $\mathbb {P}_{y}\!\left( \cdot \right)$ or $\mathbb {P}_{(x,y)}\!\left( \cdot \right)$ to emphasise the starting point of one or both of the walks *X*, *Y*.

#### Lemma 3.22

Let us consider the setup of Definition 3.21.

For each *i* let $D_i$ be the set of vertices explored during the exploration process from the set $V_{z_i}$. Then for all sufficiently large values of *n*, for any realisation of $(D_i)$ we have

$$\begin{aligned} \left| \bigcup D_i\right| \le N := \sqrt{n}\exp \left( 2A\sqrt{\frac{\mathfrak {V}\log n}{\mathfrak {h}}}\right) . \end{aligned}$$

Also there exists a positive constant *C* (not depending on $T_{x,0}$ and $T_{y,0}$) so that the number $\textrm{Bad}$ of bad vertices *z* satisfies

$$\begin{aligned} \mathbb {P} \!\Big (\textrm{Bad}\ge C\;\Big \vert \;\mathcal {B}_K^*(x)=T_{x,0},\mathcal {B}_K^*(y)=T_{y,0},\mathcal {B}_K^*(x)\cap \mathcal {B}_K^*(y)=\emptyset \Big ) = o\left( \frac{1}{n^2}\right) . \end{aligned}$$

#### Proof

The proof is similar to the derivation of [6, equation (3.11)].^8^

First we show that for each *k* the number of vertices $\widehat{S}_k$ at level *k* of $T_x$ whose level *M* ancestor (if exists) does not satisfy the truncation criterion is $\le \sqrt{n}\exp \left( \frac{3}{2}A\sqrt{\frac{\mathfrak {V}\log n}{\mathfrak {h}}}\right)$.

Let $\widetilde{S}_k$ be the set of level *k* vertices of $T_x$ not satisfying the truncation criterion. Note that by induction on *k* we have

$$\begin{aligned} \sum _{e\in \widetilde{S}_k}\exp \left( -\widetilde{W}_{T}(e)\right) \le 1 \end{aligned}$$

and by the definition of truncation criterion this gives that $|\widetilde{S}_k|\le \sqrt{n}\exp \left( A\sqrt{\frac{\mathfrak {V}\log n}{\mathfrak {h}}}\right)$.

Then using that

$$\begin{aligned} M\log b(R)\,\asymp \,\left( \log g(n)\right) f(R):\lesssim \,\left( \log g(n)\right) \frac{\log n}{g(n)}(\log g(n))\,\ll \,\frac{\log n}{\sqrt{g(n)}}\,\asymp \,\sqrt{\frac{\mathfrak {V}\log n}{\mathfrak {h}}} \end{aligned}$$

we get that

$$\begin{aligned} |\widehat{S}_k| \le b(R)^M|\widetilde{S}_{k-M}| \le \sqrt{n}\exp \left( \frac{3}{2}A\sqrt{\frac{\mathfrak {V}\log n}{\mathfrak {h}}}\right) . \end{aligned}$$

Analogously we get that the number of vertices at level *k* of $T_y$ whose level *M* ancestor does not satisfy the truncation criterion is also $\sqrt{n}\exp \left( \frac{3}{2}A\sqrt{\frac{\mathfrak {V}\log n}{\mathfrak {h}}}\right)$.

For each non-truncated long-range edge in $T_x$ and $T_y$ we reveal its *R*-ball, which has size $\le b(R)$. We continue the exploration for *L* levels in each tree, so overall the number of vertices we explore is

$$\begin{aligned} \le 2Lb(R)\sqrt{n}\exp \left( \frac{3}{2}A\sqrt{\frac{\mathfrak {V}\log n}{\mathfrak {h}}}\right) \le \sqrt{n}\exp \left( 2A\sqrt{\frac{\mathfrak {V}\log n}{\mathfrak {h}}}\right) . \end{aligned}$$

The last inequality holds for sufficiently large values of *n* and is because

$$\begin{aligned}&\log b(R) \asymp f(R) \lesssim \frac{\log n}{g(n)}\log g(n) \ll \frac{\log n}{\sqrt{g(n)}}\asymp \sqrt{\frac{\mathfrak {V}\log n}{\mathfrak {h}}}\qquad \text {and}\\&\log L \asymp \log g(n)\lesssim \log \left( \frac{\log n}{\log \log n}\right) \ll \sqrt{(\log \log n)\log n} \lesssim \sqrt{\frac{\mathfrak {V}\log n}{\mathfrak {h}}}. \end{aligned}$$

At each step of the exploration the probability of intersecting $\partial T_{x,0}$ or $\partial T_{y,0}$ is

$$\begin{aligned} \le \frac{|\partial T_{x,0}|+|\partial T_{y,0}|}{n}\le \frac{2b(R)^{K+1}}{n}, \end{aligned}$$

independently for different steps, so the probability of having $\ge C$ bad vertices is

$$\begin{aligned}&\mathbb {P} \!\Big (\mathrm{{Bad}}\ge C \;\Big \vert \;T_0\Big )\le \left( {\begin{array}{c}N\\ C\end{array}}\right) \left( \frac{2b(R)^{K+1}}{n}\right) ^{C}\\&\quad \le \exp \left( C\left( \frac{1}{2}\log n+2A\sqrt{\frac{\mathfrak {V}\log n}{\mathfrak {h}}}+2K\log b(R)-\log n\right) \right) \ll \frac{1}{n^2} \end{aligned}$$

if *C* is sufficiently large.

In the last step, we used that

$$\begin{aligned} K\log b(R)&\lesssim \log g(n)\frac{\log n}{g(n)}\log g(n) \ll \log n\qquad \text {and}\\ \sqrt{\frac{\mathfrak {V}\log n}{\mathfrak {h}}}&\asymp \frac{\log n}{\sqrt{g(n)}} \ll \log n. \end{aligned}$$

$$\square $$

#### Lemma 3.23

Let us consider the setup of Definition 3.21 and consider the coupling of *X* with $\widetilde{X}$ from $u\in V_{z_i}$ where $1\le i\le L_x+L_y$. Then for all $\theta >0$ there exist $C_L$ (in the definition of *L*) and *A* (in the truncation criterion $\textrm{Tr}(\cdot ,A)$) sufficiently large such that for all large enough *n* we have

$$\begin{aligned} \mathbb {P}_{u}\!\Big (\text {the coupling of } X \text { and } \widetilde{X} \text { succeeds}\;\Big \vert \;\mathcal {F}_{i-1}\Big ) \ge (1-\theta ){{{\mathfrak {1}}}}_{\{z_i\text { good}\}}. \end{aligned}$$

#### Proof

We follow the proof of [18, Lemma 5.6]. Using Propositions 3.18 and 3.20 with sufficiently small constants in place of $\theta$ it only remains to check the following.

- The probability that up to the time that $\widetilde{X}$ first reaches level *L* it ever returns to a vertex after visiting its depth *K* descendant should be *o*(1).
  The probability of ever returning to level *k* after visiting level $k+K$ is $\le \delta ^{K}$. We have
  $$\begin{aligned}  &   \log \left( L\delta ^K\right) =\log L-K\log \left( \frac{1}{\delta }\right) \asymp \log g(n)-\left( \log g(n)\right) \log \left( \frac{1}{\varepsilon }\right) \rightarrow \\  &   \quad -\infty \quad \text {as}\quad n\rightarrow \infty \end{aligned}$$
  since $\log \left( \frac{1}{\varepsilon }\right) \gg 1$. This proves the required bound.
- The probability that the first time when the walk visits a given level (which is at most *L*) there is an overlap at one of the vertices within distance 2*K* at the same level should be *o*(1).
  We can upper bound this probability by $L b(R)^{2K+1}\frac{b(R)N}{n-N}$. Note that
  $$\begin{aligned} \log L&\asymp \log g(n) \ll \log n,\\ K\log b(R)&\lesssim \frac{\log n}{g(n)}(\log g(n))^2 \ll \log n,\\ N&\lesssim n^{2/3}, \end{aligned}$$
  therefore $L b(R)^{(2K+1)}\frac{b(R)N}{n-N}=o(1)$ as required.
- The probability that the first time when the walk visits a given level (which is at most *L*) the optimal coupling fails in one of the vertices within distance 2*K* should be *o*(1).
  As in the previous point we get $L b(R)^{2K+1}\frac{N}{n}=o(1)$ as required.
- The probability of hitting level $K-1$ should be *o*(1). This is true by Lemma 2.4.
- The probability of $\Omega _0^X(L)$ or $\Omega _1^X(L)$ failing to hold should be *o*(1). This is also true by Lemmas 3.10 and 3.12.

$$\square $$

#### Lemma 3.24

Let us consider the setup of Definition 3.21 and consider the coupling of *X* with $\widetilde{X}$ from *x*. Then for all $\theta >0$ there exist $C_L$ and *A* sufficiently large such that we have

$$\begin{aligned}  &   \mathbb {P} \bigg (\mathbb {P}_{x}\!\Big (\text {the coupling of } X \text { and } \widetilde{X} \text { succeeds}\;\Big \vert \;\mathcal {F}_{L_x+L_y}\Big )>1-\theta \bigg | \\  &   \quad \bigg |\mathcal {B}_K^*(x)=T_{x,0},\mathcal {B}_K^*(y)=T_{y,0},\mathcal {B}_K^*(x)\cap \mathcal {B}_K^*(y)=\emptyset \bigg ) \ge 1-o\left( \frac{1}{n^2}\right) . \end{aligned}$$

An analogous result holds with analogous proof for *Y* and $\widetilde{Y}$.

#### Proof

We use a martingale argument similar to the one used in the proof of [18, Theorem 1.1]. To simplify notation we will write $\{T_{x,0},T_{y,0}\}$ for the event

$\left\{ B_K^*(x)=T_{x,0},\mathcal {B}_K^*(y)=T_{y,0},\mathcal {B}_K^*(x)\cap \mathcal {B}_K^*(y)=\emptyset \right\}$. Throughout the proof we will work conditional on this event.

Set

$$\begin{aligned} V:=\left\{ z_i\in \partial \mathcal {B}^*_{K/2}(x):\,\mathbb {P}_{x}\!\Big (X_{\tau _K}\in V_{z_i},\text { coupling of }X,\widetilde{X}\text { from }X_{\tau _K}\text { fails}\;\Big \vert \;\mathcal {F}_{L_x+L_y}\Big )\ge \theta \right\} , \end{aligned}$$

$$\begin{aligned} \widehat{V}:=\bigcup _{z\in V}V_{z}, \end{aligned}$$

and for $w\in \partial \mathcal {B}^*_K(x)$ set $h(w):=\mathbb {P}_{x}\!\Big (X_{\tau _K}=w\;\Big \vert \;\mathcal {F}_{L_x+L_y}\Big )$.

We will show that

3.9 $$\begin{aligned} \mathbb {P} \!\Big (h(\widehat{V})>3\theta \;\Big \vert \;T_{x,0},T_{y,0}\Big ) \ll \frac{1}{n^2}. \end{aligned}$$

Once we have this, we can finish the proof as follows. Note that by Lemmas 3.10 and 3.12

$$\begin{aligned}&\mathbb {P}_{x}\!\Big (\text {the coupling of } X \text { and } \widetilde{X} \text { fails}\;\Big \vert \;\mathcal {F}_{L_x+L_y}\Big )\\&\quad \le \mathbb {P}_{x}\!\Big (\Omega _0(K)^c\;\Big \vert \;\mathcal {F}_{L_x+L_y}\Big )+\mathbb {P}_{x}\!\Big (\Omega _1(K)^c\;\Big \vert \;\mathcal {F}_{L_x+L_y}\Big )\\&\qquad + \sum _{i}\mathbb {P}_{x}\!\Big (X_{\tau _K}\in V_{z_i},\text { coupling of }X,\widetilde{X}\text { from }X_{\tau _K}\text { fails}\;\Big \vert \;\mathcal {F}_{L_x+L_y}\Big )\\&\quad \le o(1)+ o(1)+\theta +\mathbb {P}_{x}\!\Big (X_{\tau _K}\in \widehat{V}\;\Big \vert \;\mathcal {F}_{L_x+L_y}\Big ). \end{aligned}$$

By (3.9) we know that with probability $1-o\left( \frac{1}{n^2}\right)$ this last expression is $<5\theta$. Considering $\frac{1}{5}\theta$ instead of $\theta$ gives the result.

Now we proceed to prove (3.9). Note that

3.10 $$\begin{aligned} h(\widehat{V})\le \sum _{i}h(V_{z_i}){{{\mathfrak {1}}}}_{\{z_i\in V\}}{{{\mathfrak {1}}}}_{\{z_i\text { good}\}}+\sum _{i}h(V_{z_i}){{{\mathfrak {1}}}}_{\{z_i\text { bad}\}}. \end{aligned}$$

We know that for any *i* using Corollary 2.5 we have

$$\begin{aligned} h(V_{z_i})\le \mathbb {P}_{x}\!\left( \tau _{z_i}<\infty \right) \le \delta ^{\frac{K}{2}} \le \frac{1}{(\log n)^2} \end{aligned}$$

since $\varepsilon \le (\log n)^{-\beta }$ for some $\beta >0$, $\delta \lesssim \varepsilon ^{\frac{1}{3}}$, and $K\gg 1$.

Then by Lemma 3.22 with probability $1-o\left( \frac{1}{n^2}\right)$ we can bound the second sum in (3.10) as

$$\begin{aligned} \sum _{i}h(V_{z_i}){{{\mathfrak {1}}}}_{\{z_i\text { bad}\}}\le \frac{C}{(\log n)^2} \ll 1. \end{aligned}$$

We know that

$$\begin{aligned}&\mathbb {E}\!\left[ h(V_{z_i}){{{\mathfrak {1}}}}_{\{z_i\in V\}}{{{\mathfrak {1}}}}_{\{z_i\text { good}\}}\;\Bigg \vert \;\mathcal {F}_{i-1}\right] \\&\quad = h(V_{z_i}){{{\mathfrak {1}}}}_{\{z_i\text { good}\}}\\&\qquad \mathbb {P} \!\Big (\mathbb {P}_{x}\!\Big (X_{\tau _K}\in V_{z_i},\text { coupling of }X,\widetilde{X}\text { from }X_{\tau _K}\text { fails}\;\Big \vert \;\mathcal {F}_{L_x+L_y}\Big )\ge \theta \;\Big \vert \;\mathcal {F}_{i-1}\Big ). \end{aligned}$$

By Markov’s inequality we have

$$\begin{aligned}&\mathbb {P} \!\Big (\mathbb {P}_{x}\!\Big (X_{\tau _K}\in V_{z_i},\text { coupling of }X,\widetilde{X}\text { from }X_{\tau _K}\text { fails}\;\Big \vert \;\mathcal {F}_{L_x+L_y}\Big )\ge \theta \;\Big \vert \;\mathcal {F}_{i-1}\Big )\\&\quad \le \frac{\mathbb {E}\!\left[ \mathbb {P}_{x}\!\Big (X_{\tau _K}\in V_{z_i},\text { coupling of }X,\widetilde{X}\text { from }X_{\tau _K}\text { fails}\;\Big \vert \;\mathcal {F}_{L_x+L_y}\Big )\;\Bigg \vert \;\mathcal {F}_{i-1}\right] }{\theta }\\&\quad \le \max _{u\in V_{z_i}}\frac{\mathbb {P}_{u}\!\Big (\text {coupling of }X,\widetilde{X}\text { fails}\;\Big \vert \;\mathcal {F}_{i-1}\Big )}{\theta }\le \frac{\theta ^2{{{\mathfrak {1}}}}_{\{z_i\text { good}\}}+{{{\mathfrak {1}}}}_{\{z_i\text { bad}\}}}{\theta } \end{aligned}$$

for sufficiently large values of $C_L$ and *A*, by using Lemma 3.23.

This then gives

$$\begin{aligned} \mathbb {E}\!\left[ h(V_{z_i}){{{\mathfrak {1}}}}_{\{z_i\in V\}}{{{\mathfrak {1}}}}_{\{z_i\text { good}\}}\;\Bigg \vert \;\mathcal {F}_{i-1}\right] \le \theta h(V_{z_i}). \end{aligned}$$

Let

$$\begin{aligned} R_i:= h(V_{z_i}){{{\mathfrak {1}}}}_{\{z_i\in V\}}{{{\mathfrak {1}}}}_{\{z_i\text { good}\}},\qquad M_k:= \sum _{i=1}^{k}(R_i-\mathbb {E}\!\left[ R_i\;\Bigg \vert \;\mathcal {F}_{i-1}\right] ). \end{aligned}$$

Then $(M_k)$ is a martingale with respect to the filtration $\left( \mathcal {F}_k\right)$, and $|M_k-M_{k-1}|\le h(V_{z_k})$. Also

$$\begin{aligned} M_{L_x+L_y}\ge \sum _{i}R_i-\theta \sum _ih(V_{z_i})= \sum _{i}R_i-\theta . \end{aligned}$$

Then by the Azuma-Hoeffding inequality we get that

$$\begin{aligned}\mathbb {P} \!\Big (\sum _{i}h(V_{z_i}){{{\mathfrak {1}}}}_{\{z_i\in V\}}{{{\mathfrak {1}}}}_{\{z_i\text { good}\}}>2\theta \;\Big \vert \;T_{x,0},T_{y,0}\Big )&\le \mathbb {P} \!\Big (M_{L_x+L_y}>\theta \;\Big \vert \;T_{x,0},T_{y,0}\Big )\\&\le \exp \left( -\frac{c\theta ^2}{\sum _{i}h(V_{z_i})^2}\right) \\&\le \exp \left( -c\theta ^2(\log n)^2\right) \ll \frac{1}{n^2}. \end{aligned}$$

In the second line we used that $\sum _{i}h(V_{z_i})^2\le \left( \max _i h(V_{z_i})\right) \sum _{i}h(V_{z_i})\le \frac{1}{(\log n)^2}$.

This finishes the proof. $\square$

## Proof of cutoff

In this section we prove that for *G* and $\varepsilon$ satisfying Assumption 3.1, $t_0$ and $t_w$ as in Definition 3.3, with high probability the simple random walk on $G^*$ has cutoff around $t_0$ with a window of order $t_w$. In the next section we explain how to use this to deduce part (a) in Theorems 1, 2, 3 and 4 in the case when $\varepsilon _n\lesssim (\log n)^{-\beta }$ for some $\beta >0$. In Sect. 6 we prove the rest of the statement of these theorems.

### Lower bounding $\sum _{z}\mathbb {P}_{x}\!\left( X_{\tau _{L}}=z,\Omega _0(L),\Omega _1(L)\right) \mathbb {P}_{\eta (y)}\!\left( X_{\tau _{L}}=\eta (z),\Omega _0(L),\Omega _1(L)\right)$

To prove concentration of the probability that a walk from *x* and a walk from *y* hits level *L* of the corresponding trees at *z* and *w* respectively that are long-range neighbours, we will use Lemma 1.5. This lemma is based on a result of Chatterjee in [10], and was stated in this form and used for non-backtracking walks on the configuration model in [5].

#### Proposition 4.1

Let *x*, *y* be two vertices of the graph *G* at graph distance $>2K$ and let $T_{x,0}$ and $T_{0,y}$ be two possible realisations of the first *K* levels of the quasi-tree of corresponding to $G^*$ centred at *x* and *y*, respectively. Then for any $\theta >0$ for sufficiently large values of $C_L$ and *A* we have

$$\begin{aligned}  &   \mathbb {P} \bigg (\sum _{z}\mathbb {P}_{x}\!\Big (X_{\tau _{L}}=z,\Omega _0(L),\Omega _1(L)\;\Big \vert \;G^*\Big )\mathbb {P}_{y}\!\Big (X_{\tau _{L}}=\eta (z),\Omega _0(L),\Omega _1(L)\;\Big \vert \;G^*\Big )\\  &   \quad \ge \frac{1}{n}(1-\theta )\bigg | \bigg |\mathcal {B}_K^*(x)=T_{x,0},\mathcal {B}_K^*(y)=T_{y,0},\mathcal {B}_K^*(x)\cap \mathcal {B}_K^*(y)=\emptyset \bigg )\\  &   \quad \ge 1-o\left( \frac{1}{n^2}\right) . \end{aligned}$$

#### Proof

For $$u\in \partial T_x$$, $$v\in \partial T_y$$ let

$$\begin{aligned}w_x(u):=\mathbb {P}_{x}\!\Big (X_{\tau _{L}}=u,\Omega ^X_2\;\Big \vert \;G^*\Big ),\quad w_y(v):=\mathbb {P}_{y}\!\Big (Y_{\tau _{L}}=w,\Omega ^Y_2\;\Big \vert \;G^*\Big ).\end{aligned}$$

where we recall that $\Omega _2^X$ is the event that the coupling of *X* and $\widetilde{X}$ is successful and $\Omega _2^Y$ is the analogous event for *Y*.

Then we have

$$\begin{aligned}&\sum _{z}\mathbb {P}_{x}\!\Big (X_{\tau _{L}}=z,\Omega _0(L),\Omega _1(L)\;\Big \vert \;G^*\Big )\mathbb {P}_{y}\!\Big (X_{\tau _{L}}=\eta (z),\Omega _0(L),\Omega _1(L)\;\Big \vert \;G^*\Big )\\&\quad \ge \sum _{u\in \partial T_x}\sum _{v\in \partial T_y}w_x(u)w_y(v){{{\mathfrak {1}}}}_{v=\eta (u)}. \end{aligned}$$

Let $w_{u,v}=w_x(u)w_y(v)$ for $u\in \partial T_x$, $v\in \partial T_y$ and 0 otherwise. By the definition of $\textrm{Tr}'(\cdot )$ we have $w_x(u),w_y(v)\le \frac{1}{\sqrt{n}\log n}$ for $u\in \partial T_x$, $v\in \partial T_y$, hence $w_{u,v}\le \frac{1}{n(\log n)^2}$ for all *u*, *v*.

Using Lemma 1.5 for these weights and $a=\frac{c}{|\mathcal {I}|-1}$ where *c* is some sufficiently small constant and noting that $\max _{i\ne j}(w_{i,j}+w_{j,i})\le \frac{2}{n(\log n)^2}$, $m\le \frac{1}{|\mathcal {I}|-1}$ and $|\mathcal {I}|\le n$ we get that

$$\begin{aligned}&\mathbb {P} \!\Big (\sum _{u\in \partial T_x}\sum _{v\in \partial T_y}w_x(u)w_y(v){{{\mathfrak {1}}}}_{v=\eta (u)}>\frac{1}{|\mathcal {I}|-1}\left( m-a\right) \;\Big \vert \;\mathcal {F}_{L_x+L_y}\Big )\\&\quad \le \exp \left( -\frac{c^2}{8}(\log n)^2\right) \ll \frac{1}{n^2} \end{aligned}$$

where

$$\begin{aligned}  &   m=\frac{1}{|\mathcal {I}|-1}\left( \sum _{u\in \partial T_x}w_x(u)\right) \left( \sum _{v\in \partial T_y}w_y(v)\right) =\frac{1}{|\mathcal {I}|-1}\mathbb {P}_{x}\!\Big (\Omega _2^X\;\Big \vert \;\mathcal {F}_{L_x+L_y}\Big )\\  &   \quad \mathbb {P}_{y}\!\Big (\Omega _2^Y\;\Big \vert \;\mathcal {F}_{L_x+L_y}\Big ). \end{aligned}$$

By Lemma 3.24 we know that for sufficiently large values of $C_L$ and *A*, conditional on the event $\{\mathcal {B}_K^*(x)=T_{x,0},\mathcal {B}_K^*(y)=T_{y,0},\mathcal {B}_K^*(x)\cap \mathcal {B}_K^*(y)=\emptyset \}$, with probability $1-o\left( \frac{1}{n^2}\right)$ we have

$$\begin{aligned} \mathbb {P}_{x}\!\Big (\Omega _2^X\;\Big \vert \;\mathcal {F}_{L_x+L_y}\Big )\mathbb {P}_{y}\!\Big (\Omega _2^Y\;\Big \vert \;\mathcal {F}_{L_x+L_y}\Big ) \ge (1-\theta )^2. \end{aligned}$$

We also know that $\frac{1}{|\mathcal {I}|-1}\ge \frac{1}{n}$.

Putting these together gives the result. $\square$

### Upper bounding the mixing time

For a random walk *X* on $G^*$ we define a stopping time $\tau$ as follows. Let $\tau _{K\text {-root}}$ be the first time the walk hits a *K*-root and let $\tau _{2L}$ be defined as before for the walk $(X_s)_{s\ge \tau _{K\text {-root}}}$. Let

4.1 $$\begin{aligned} \tau =\tau _{K\text {-root}}+\tau _{2L}, \end{aligned}$$

where *K* and *L* are as in Definition 3.3, with values of the constants to be specified later.

To upper bound the mixing time we show that with probability close to 1 the value of $\tau$ is upper bounded by $t_0+O(t_w)$ (where $t_0$ and $t_w$ are as in Definition 3.3) and show that for any starting point, the distribution of $X_{\tau }$ is close to uniform. This gives an upper bound on the hitting time of large sets, then we use a result comparing mixing and hitting times.

We denote the uniform distribution on the vertices of *G* by $\mathcal {U}$.

#### Lemma 4.2

For any $\theta >0$ for sufficiently large values of $C_L$ we have

$$\mathbb {P}\!\left( \max _{x}d_{\textrm{TV}}\left( {\mathbb {P}_{x}\!\Big (X_{\tau }\in \cdot \;\Big \vert \;G^*\Big )},{\mathcal {U}(\cdot )}\right) \le \theta \right) = 1-o(1).$$

#### Proof

We are conditioning on $G^*$ throughout the proof, but it will be omitted from the notation.

It is sufficient to show that

$$\mathbb {P}\!\left( \max _{x:K\text {-root}}d_{\textrm{TV}}\left( {\mathbb {P}_{x}\!\left( X_{\tau _{2L}}\in \cdot \right) },{\mathcal {U}(\cdot )}\right) \le \theta \right) = 1-o(1).$$

By Lemma 3.14 for any *K*-root *x* we have

$$\begin{aligned}&d_{\textrm{TV}}\left( {\mathbb {P}_{x}\!\left( X_{\tau _{2L}}\in \cdot \right) },{\mathcal {U}(\cdot )}\right) \\&\quad \le \sum _y\left( \mathcal {U}(y)-\sum _{z}\mathbb {P}_{x}\!\left( X_{\tau _{L}}=z,\Omega _0(L),\Omega _1(L)\right) \mathbb {P}_{\eta (y)}\!\left( X_{\tau _{L}}=\eta (z),\Omega _0(L),\Omega _1(L)\right) \right) ^++o(1)\\&\quad \le 9\sum _{y:\text {non-}K\text {-root}}\mathcal {U}(y)+\sum _{y:\mathcal {B}_K^*(x)\cap \mathcal {B}_K^*(y)\ne \emptyset }\mathcal {U}(y)\\&\qquad +\sum _{\begin{array}{c} y:K\text {-root},\\ \mathcal {B}_K^*(x)\cap \mathcal {B}_K^*(y) =\emptyset \end{array}}\\&\quad \left( \mathcal {U}(y)-\sum _{z}\mathbb {P}_{x}\!\left( X_{\tau _{L}}=z,\Omega _0(L),\Omega _1(L)\right) \mathbb {P}_{y}\!\left( X_{\tau _{L}}=\eta (z),\Omega _0(L),\Omega _1(L)\right) \right) ^++o(1). \end{aligned}$$

^9^

By Lemma 3.6 we know that whp for all *x* we have $\sum _{y:\text {non-}K\text {-root}}\mathcal {U}(y)=o(1)$ and by Lemma 3.7 we know that for all *x* and all realisations of $G^*$ we have $\sum _{y:\mathcal {B}_K^*(x)\cap \mathcal {B}_K^*(y)\ne \emptyset }\mathcal {U}(y)=o(1)$. Also

$$\begin{aligned}  &   \mathbb {P}\!\left( \exists  K\text {-root }x:\right) \\  &   \quad \mathbb {P}\!\left( \sum _{\begin{array}{c} y:K\text {-root},\\ \mathcal {B}_K^*(x)\cap B_K^*(y)=\emptyset \end{array}}\left( \mathcal {U}(y)-\sum _{z}\mathbb {P}_{x}\!\left( X_{\tau _{L}}=z,\Omega _0(L),\Omega _1(L)\right) \mathbb {P}_{y}\!\left( X_{\tau _{L}}=\eta (z),\Omega _0(L),\Omega _1(L)\right) \right) ^+>\theta \right) \\  &   \quad \le \mathbb {P}\bigg (\exists  K\text {-roots }x,y\text { with }\mathcal {B}_K^*(x)\cap \mathcal {B}_K^*(y)=\emptyset :\hspace{8cm}\\  &   \qquad \sum _{z}\mathbb {P}_{x}\!\left( X_{\tau _{L}}=z,\Omega _0(L),\Omega _1(L)\right) \mathbb {P}_{y}\!\left( X_{\tau _{L}}=\eta (z),\Omega _0(L),\Omega _1(L)\right)<\frac{1}{n}(1-\theta )\bigg )\\  &   \quad \le \sum _{x,y}\mathbb {P}\bigg (\sum _{z}\mathbb {P}_{x}\!\left( X_{\tau _{L}}=z,\Omega _0(L),\Omega _1(L)\right) \mathbb {P}_{y}\!\left( X_{\tau _{L}}=\eta (z),\Omega _0(L),\Omega _1(L)\right)<\frac{1}{n}(1-\theta ),\hspace{3cm}\\  &   \qquad {x,y K\text {-roots},\, \mathcal {B}_K^*(x)\cap \mathcal {B}_K^*(y)=\emptyset }\bigg )\\  &   \quad \le 10\sum _{\begin{array}{c} x,y:\\ d_G(x,y)>2K \end{array}}\mathbb {P}\bigg (\sum _{z}\mathbb {P}_{x}\!\left( X_{\tau _{L}}=z,\Omega _0(L),\Omega _1(L)\right) \mathbb {P}_{y}\!\left( X_{\tau _{L}}=\eta (z),\Omega _0(L),\Omega _1(L)\right) \\  &   \quad <\frac{1}{n}(1-\theta )\bigg |\bigg |{x,y K\text {-roots},\,\mathcal {B}_K^*(x)\cap \mathcal {B}_K^*(y)=\emptyset }\bigg ) . \end{aligned}$$

^10^

From Proposition 4.1 we know that each of the probabilities appearing in the last sum is $o\left( \frac{1}{n^2}\right)$, hence the whole sum is *o*(1). $\square$

#### Lemma 4.3

Let $\tau$ be defined as above. Then for any $\theta$ and $C_L$, there exists a constant *C* such that the following holds. With high probability $G^*$ is such that for any vertex *x* we have

$$\begin{aligned} \mathbb {P}_{x}\!\Big (\tau >t_0+Ct_w\;\Big \vert \;G^*\Big ) \le \theta . \end{aligned}$$

In the proof we will use the following result.

#### Lemma 4.4

Let $T_0$ be any possible realisation of the first *K* levels of a random quasi-tree, and let *T* be a random quasi-tree conditioned on its first *K* levels being equal to $T_0$. Then for any $\theta \in (0,1)$ there exists a positive constant *C* (not depending on $T_0$, but depending on $C_L$) with the following property. With probability $1-o\left( \frac{1}{n}\right)$ the quasi-tree *T* is such that a random walk on *T* starting from its root $\rho$ satisfies

4.2 $$\begin{aligned} \mathbb {P}_{\rho }\!\Big (\tau _L>\frac{1}{2}t_0+Ct_w\;\Big \vert \;T\Big )\le \theta . \end{aligned}$$

#### Proof of Lemma 4.4

For each $z\in \partial \mathcal {B}_K(\rho )$ let us consider the quasi-tree *T*(*z*) descending from *z* (as in Definition 2.10). These *T*(*z*) are distributed as random quasi-trees, independently of each other.

Let $\theta _1\in (0,1)$ and $c_1$ be constants to be specified later and let

4.3 $$\begin{aligned} h(z):=&\mathbb {P}_{\rho }\!\Big (X_{\tau _K}=z\;\Big \vert \;T\Big ), \end{aligned}$$

4.4 $$\begin{aligned} \widehat{V}:=&\left\{ z\in \partial \mathcal {B}_K(\rho ):\,\mathbb {P}_{z}\!\Big (d_{T(z)}\left( z,X^{(z)}_{\frac{1}{2}t_0+c_1t_w}\right) <L\;\Big \vert \;T(z)\Big )\le \theta _1\right\} , \end{aligned}$$

where $X^{(z)}$ is a random walk on *T*(*z*), started from *z*.

We will show that for any $\theta _2\in (0,1)$, for sufficiently large values of $c_1$, with probability $1-o\left( \frac{1}{n}\right)$ we have

4.5 $$\begin{aligned} \mathbb {P}_{\rho }\!\Big (X_{\tau _K}\in \widehat{V}\;\Big \vert \;T\Big ) = \sum _{z\in \partial \mathcal {B}_K(\rho )}h(z){{{\mathfrak {1}}}}_{z\in \widehat{V}} \ge 1-\theta _2. \end{aligned}$$

Then we will show that on the event (4.5) the bound (4.2) holds for sufficiently large values of *C*.

Using Lemma 2.19, Markov’s inequality, and the definition of the parameters $t_0$, $t_w$ and *L*, we get that for any $\theta _3\in (0,1)$, for sufficiently large values of $c_1$, all $z\in \partial \mathcal {B}_K(\rho )$ satisfy $\mathbb {P}\!\left( z\in \widehat{V}\right) \ge 1-\theta _3$, and these events are independent for different *z*.

Also, by Lemma 2.4 we know that for any *z* we have $h(z)\le \delta ^K$. This is $\le (\log n)^{-2}$ for sufficiently large *n*.

Then using [16, Theorem 21.6] for the random variables $\delta ^{-K}h(z){{{\mathfrak {1}}}}_{z\in \widehat{V}}$ we get that for any $\theta _3<\theta _2$ we have

$$\begin{aligned} \mathbb {P}\!\left( \sum _{z\in \partial \mathcal {B}_K(\rho )}h(z){{{\mathfrak {1}}}}_{z\in \widehat{V}}\le 1-\theta _2\right) \le \exp \left( -\frac{(\theta _2-\theta _3)^2}{2\left( 1-\frac{1}{3}\theta _2-\frac{2}{3}\theta _3\right) }\delta ^{-K}\right) \ll \frac{1}{n}. \end{aligned}$$

Now let us work on the event (4.5) to show that the bound (4.2) holds.

Let *X* be a random walk on *T*, started from $\rho$. Note that for (4.2) it is sufficient that the following events hold for some $c_1$ and $c_2$.

(i) $$\tau _K\le c_2t_w$$,
(ii) $$X_{\tau _K}\in \widehat{V}$$,
(iii) *X* does not backtrack the long-range edge $\left( X_{\tau _K-1},X_{\tau _K}\right)$, and
(iv) the walk $(X_s)_{s\ge \tau _K}$ hits level $L-K$ of *T*(*z*) in $\le \frac{1}{2}t_0+c_1t_w$ steps,

For any fixed *T* satisfying (4.5), we can bound the probability of each of (i)-(iv) as follows.

It is easy to see that (i) and (iii) are both high probability events. We are working on (4.5), so we know that (ii) has probability $\ge 1-\theta _2$. If $X_{\tau _K}=z$, then the walk $(X_s)_{s\ge \tau _K}$ can be coupled with a walk $X^{(z)}$ on *T*(*z*) such that on the event that *X* does not backtrack the long-range edge $\left( X_{\tau _K-1},X_{\tau _K}\right)$, the two walks agree. Using this and the definition of $\widehat{V}$ we see that the probability of (ii) and (iii) holding, but (iv) failing is $\le \theta _1$.

Choosing $\theta _1$ and $\theta _2$ sufficiently small, we get the result. $\square$

#### Proof of Lemma 4.3

Firstly, we explain how we can use Lemma 4.4 to bound $\tau _L$ for a walk on $G^*$ starting from a *K*-root. Then we explain how we can break down $\tau$ into smaller parts that each concern the time it takes to hit a *K*-root, or $\tau _L$ from a *K*-root on $G^*$.

Let *x* be any vertex of *G* and let us condition on the event that it is a *K*-root with the first *K* levels being equal to $T_{x,0}$. Let us explore a random neighbourhood of *x* and couple it to a random quasi-tree $T_x$ conditioned to be equal to $T_{x,0}$ in the first *K* levels, using an exploration and coupling identical to the ones in Definition 3.21 (but only for one vertex, not two). Let us also couple a walk *X* on $G^*$ from *x* to a walk $\widetilde{X}$ on *T* from *x*, as in Definition 3.21. Then from Lemma 3.24 we see that for any $\theta \in (0,1)$, with probability $1-o\left( \frac{1}{n}\right)$ the explored neighbourhood of *x* in $G^*$ is such that the coupling of *X* and $\widetilde{X}$ succeeds up to level *L* with probability $\ge 1-\theta$. Combining this with Lemma 4.4 we also get that for any $\theta \in (0,1)$, with probability $1-o\left( \frac{1}{n}\right)$ the explored neighbourhood of *x* in $G^*$ is such that *X* satisfies $\mathbb {P}_{x}\!\Big (\tau _L<\frac{1}{2}t_0+Ct_w\;\Big \vert \;G^*\Big )\ge 1-\theta$. Taking a union bound we get that for any $\theta \in (0,1)$, whp $G^*$ is such that for any *K*-root *x*, a walk on $G^*$ starting from *x* satisfies $\mathbb {P}_{x}\!\Big (\tau _L<\frac{1}{2}t_0+Ct_w\;\Big \vert \;G^*\Big )\ge 1-\theta$.

In what follows, let us work on the event *A* that is the intersection of the above high probability event and the high probability event in Lemma 3.5.

Then for any *x*, a walk on $G^*$ from *x* with high probability satisfies

$$\begin{aligned} \tau _{K\text {-root}}\le \frac{bK}{\varepsilon } \asymp \frac{1}{\varepsilon }\log g(n) \ll \frac{1}{\varepsilon }\sqrt{g(n)} \asymp t_w. \end{aligned}$$

Let $\tau _L^{(1)}$ be defined as $\tau _L$ for the walk $(X_s)_{s\ge \tau _{K\text {-root}}}$, let $\tau _{K\text {-root}}^{(2)}$ be the first time when the walk ${(X_k)_{k\ge \tau _{K\text {-root}}+\tau _L^{(1)}}}$ hits a *K*-root and let $\tau ^{(2)}_L$ be defined as $\tau _{L}$ for the walk ${(X_k)_{k\ge \tau _{K\text {-root}}+\tau _L+\tau _{K\text {-root}}^{(2)}}}$. Let $\widetilde{X}$ be the walk corresponding to *X* on the quasi-tree *T* corresponding to $G^*$ (as in Definition 3.8).

Note that on the event that

(i) $$\tau _{K\text {-root}}<Ct_w$$,
(ii) $$\tau ^{(1)}_L<\frac{1}{2}t_0+Ct_w$$,
(iii) $\widetilde{X}$ does not backtrack the long-range edge $\left( \widetilde{X}_{\tau _{K\text {-root}}+\tau ^{(1)}_L-1},\widetilde{X}_{\tau _{K\text {-root}}+\tau ^{(1)}_L}\right)$,
(iv) $$\tau _{K\text {-root}}^{(2)}<Ct_w$$,
(v) $\widetilde{X}$ does not backtrack the long-range edge $\left( \widetilde{X}_{\tau _{K\text {-root}}+\tau ^{(1)}_L+\tau _{K\text {-root}}^{(2)}-1},\widetilde{X}_{\tau _{K\text {-root}}+\tau ^{(1)}_L+\tau _{K\text {-root}}^{(2)}}\right)$, and
(vi) $$\tau ^{(2)}_L<\frac{1}{2}t_0+Ct_w$$,

we have $$\tau _{2L}<t_0+4Ct_w$$.

Using the definition of *A* and that the probability of $\widetilde{X}$ backtracking a given long-range edge is $\le \delta \ll 1$, we get that with probability $\ge 1-3\theta$ the events (i)-(vi) all hold. This finishes the proof. $\square$

We recall that the relaxation time and the absolute relaxation time of a Markov chain with transition matrix *P* are defined as

$$\begin{aligned} t_{\textrm{rel}}:=&\left( 1-\sup \{\lambda :\,\lambda \text { is an eigenvalue of }P,\,\lambda \ne 1\}\right) ^{-1},\\ t_{\textrm{rel}}^{\textrm{abs}}:=&\left( 1-\sup \{|\lambda |:\,\lambda \text { is an eigenvalue of }P,\,\lambda \ne 1\}\right) ^{-1}. \end{aligned}$$

#### Lemma 4.5

With high probability $G^*$ is such that the absolute relaxation time of the random walk on $G^*$ satisfies $t_{\textrm{rel}}^{\textrm{abs}}\lesssim \frac{1}{\varepsilon }$.

#### Proof

Let *K* be the graph with the same vertices and edges as $G^*$, but all edges having weight 1. From [18, Theorem 6.1] we know that whp $t_{\textrm{rel}}^{\text {abs},K}\lesssim 1$. It is easy to check that $\varepsilon P_{K}(x,y)\le P_{G^*}(x,y)\lesssim P_{K}(x,y)$ and $\pi _{G^*}(x)\asymp \pi _{K}(x)$ for all *x* and *y*. This implies a comparison of the Dirichlet forms [19, (13.2)] for the two chains. Then using [19, Lemma 13.18] we get the required bound on $t_{\textrm{rel}}^{\text {abs},G^*}$. $\square$

#### Lemma 4.6

(Proposition 1.8 and Remark 1.9 in [2]) For any reversible irreducible finite chain and any $\theta \in \left( 0,\frac{1}{4}\right]$ we have

$$\textrm{hit}_{1-\theta /4}\left( \frac{5}{4}\theta \right) \le t_{\textrm{mix}}\left( \theta \right) \le \textrm{hit}_{1-\theta /4}\left( \frac{3}{4}\theta \right) +\Bigg \lceil \frac{3}{2}t_{\textrm{rel}}^{\textrm{abs}}\left| \log \left( \frac{\theta }{4}\right) \right| \Bigg \rceil $$

where $$\textrm{hit}_{\alpha }\left( \theta \right) =\min \left\{ s:\forall x,\forall A\text { with }\pi (A)\ge \alpha \text { we have }\mathbb {P}_{x}\!\left( \tau _A>s\right) \le \theta \right\} $$.

#### Lemma 4.7

(Corollary 3.4 in [2]) For any reversible irreducible finite chain and any $0<\theta _1<\theta _2<0$ and $0<\alpha _1\le \alpha _2<1$ we have

$$\begin{aligned} \textrm{hit}_{\alpha _2}\left( \theta _2\right) \le \textrm{hit}_{\alpha _1}\left( \theta _2\right) \le \textrm{hit}_{\alpha _2}\left( \theta _2-\theta _1\right) +\Bigg \lceil \frac{1}{\alpha _1}t_{\textrm{rel}}^{\textrm{abs}}\log \left( \frac{1-\alpha _1}{(1-\alpha _2)\theta _1}\right) \Bigg \rceil . \end{aligned}$$

#### Proposition 4.8

For any $\theta \in (0,1)$, there exists $C>0$ such that with high probability the mixing time of the random walk on $G^*$ satisfies

$$\begin{aligned} t_{\textrm{mix}}\left( \theta \right) \le t_0+Ct_w \end{aligned}$$

for all sufficiently large *n*.

#### Proof

From Lemma 4.2 we know that for sufficiently large values of $C_L$ with high probability we have

$$\begin{aligned} \max _{x}d_{\textrm{TV}}\left( {\mathbb {P}_{x}\!\Big (X_{\tau }\in \cdot \;\Big \vert \;G^*\Big )},{\mathcal {U}(\cdot )}\right) \le \frac{1}{8}\theta . \end{aligned}$$

From Lemma 4.3 we know that for the above $C_L$ and for a sufficiently large $C'$, with high probability we have

$$\begin{aligned} \max _x\mathbb {P}_{x}\!\Big (\tau >t_0+C't_w\;\Big \vert \;G^*\Big )\le \frac{1}{8}\theta . \end{aligned}$$

Let *x* be any vertex and let *A* be any set with $\pi (A)\ge 1-\frac{1}{4(\Delta +1)}\theta$. Then $\pi (A^c)\ge \frac{|A^c|}{(\Delta +1)n}=\frac{1}{\Delta +1}\mathcal {U}(A^c)$, hence $\mathcal {U}(A)\ge 1-\frac{1}{4}\theta$. Then on the above high probability event we have

$$\begin{aligned}&\mathbb {P}_{x}\!\left( \tau _A>t_0+C't_w\right) \le \mathbb {P}_{x}\!\left( X_{\tau }\not \in A\right) +\mathbb {P}_{x}\!\left( \tau>t_0+C't_w\right) \\&\quad \le d_{\textrm{TV}}\left( {\mathbb {P}_{x}\!\left( X_{\tau }\in \cdot \right) },{\mathcal {U}(\cdot )}\right) +\mathcal {U}(A^c)+\mathbb {P}_{x}\!\left( \tau >t_0+C't_w\right) \le \frac{1}{8}\theta +\frac{1}{4}\theta +\frac{1}{8}\theta . \end{aligned}$$

Therefore $\textrm{hit}_{1-\frac{1}{4(\Delta +1)}\theta }\left( \frac{1}{2}\theta \right) \le t_0+C't_w$. From Lemma 4.5 we know that $t_{\textrm{rel}}\le t_{\textrm{rel}}^{\textrm{abs}}\lesssim \frac{1}{\varepsilon }\ll t_w$. These together with Lemmas 4.7 and 4.6 give the result. $\square$

### Lower bounding the mixing time

The lower bound on the mixing time can be proved analogously to the proof in [18]. The exploration and coupling used here will be very similar to the ones used for the upper bound, but we will only explore around one vertex *x*, and the threshold for the truncation criterion and the number of explored levels will be different.

Given a quasi-tree *T* let us define a truncation event for a long-range edge *e* and a constant $A>0$ as

$$\begin{aligned} \widetilde{\textrm{Tr}}(e,A):=\left\{ \widetilde{W}_T(e)>\log n-A\sqrt{\frac{\mathfrak {V}\log n}{\mathfrak {h}}}\right\} . \end{aligned}$$

Let

$$\begin{aligned} t:=t_0-Bt_w,\qquad \widetilde{L}:=\nu t+2D\sqrt{\varepsilon t} \end{aligned}$$

for some constants *B* and *D* to be chosen later.

#### Proposition 4.9

Let *G* be as before, assume that Assumption 3.1 holds and let *R*, *K* and *M* be as in Definition 3.3. Let *T* be the random quasi-tree associated to *G*. Let $T_0$ be any realisation of the first *K* levels of *T*. Let *X* be a simple random walk on *T* started from its root. Then for all $\theta \in (0,1)$ there exist *B* (depending on $\theta$) and *A* (depending on $\theta$ and *B*) sufficiently large such that

$$\begin{aligned} \mathbb {P} \!\Big (\bigcup _{k\le \tau _{\widetilde{L}}}\widetilde{\textrm{Tr}}((X_{k-1},X_k),A)\;\Big \vert \;B_K(\rho )=T_0\Big )<\theta . \end{aligned}$$

#### Proof

Similarly to the proof of Proposition 3.18 it is sufficient to show that

$$\begin{aligned} \mathbb {P} \!\Big (\widetilde{W}_T(\xi _{\widetilde{L}})\ge \log n-A\sqrt{\frac{\mathfrak {V}\log n}{\mathfrak {h}}}\;\Big \vert \;T_0\Big ) \le \theta . \end{aligned}$$

Similarly to the proof of Lemma 3.17 we can show that $\widetilde{W}_T(e)\ge \log n-A\sqrt{\frac{\mathfrak {V}\log n}{\mathfrak {h}}}$ implies

${W}_T(e)\ge \log n-A'\sqrt{\frac{\mathfrak {V}\log n}{\mathfrak {h}}}$ for all $A'>A$ for sufficiently large *n*.

From Proposition 2.20 we know that for a sufficiently large value of $C'$ we have

$$\begin{aligned} \mathbb {P} \!\Big (W_T(\xi _{\widetilde{L}})\ge \widetilde{L}\mathfrak {h}+C'\sqrt{\widetilde{L}\mathfrak {V}}\;\Big \vert \;T_0\Big ) \le \theta . \end{aligned}$$

For any value of $A'$ for sufficiently large values of *B* and *n* we have

$$\begin{aligned} \widetilde{L}\mathfrak {h}+C'\sqrt{\widetilde{L}\mathfrak {V}}\le \log n-A'\sqrt{\frac{\mathfrak {V}\log n}{\mathfrak {h}}}, \end{aligned}$$

hence

$$\begin{aligned} \mathbb {P}\!\left( {W}_T(\xi _{\widetilde{L}})\ge \log n-A'\sqrt{\frac{\mathfrak {V}\log n}{\mathfrak {h}}}\right) \le \theta . \end{aligned}$$

This finishes the proof. $\square$

#### Definition 4.10

Let *x* be a vertex of *G* and let us condition on it being a *K*-root with *K*-neighbourhood $T_0=\mathcal {B}^*_{K}(x)$.

Let us define $z_1,...,z_{L_x}$ and $V_{z_i}$ as in Definition 3.21. For each $z_i$ we will define an exploration process of $G^*$ corresponding to the set $V_{z_i}$ by constructing a coupling between a subset of $G^*$ and a quasi-tree *T* that is distributed like the random quasi-tree corresponding to the graph *G*, conditioned to be $T_{0}$ at the first *K* levels around its root.

Let us define the exploration as in Definition 3.21 with the following modifications. We use the truncation criterion $\widetilde{\textrm{Tr}}(e,A)$ instead of $\textrm{Tr}(e,A)$. We explore up to level $\widetilde{L}$. We do not consider the half-edges from the last explored level of *T*.

Let us define $\mathcal {F}_i$ and good and bad vertices as in Definition 3.21.

Define the coupling of a random walk *X* on $G^*$ from $u\in V_{z_i}$ with a random walk $\widetilde{X}$ on *T* from *u* by moving them together until $\widetilde{X}$ reaches level $\widetilde{L}$ as long as none of the following happen.

(i) $\widetilde{X}$ crosses a truncated edge;
(ii) $\widetilde{X}$ visits level $K-1$ of *T*;
(iii) $\Omega _0(\widetilde{L}-K)$ fails to hold, i.e. $\widetilde{X}$ crosses more than $\frac{R}{2}$ short edges in a row.

If none of these events happen, we say that the coupling is successful. Otherwise we say that the coupling fails.

Let us also define the coupling of a random walk *X* on $G^*$ from *x* and a random walk $\widetilde{X}$ on *T* from *x* by moving them together until $\widetilde{X}$ hits level *K* and then using the above coupling from the level *K* vertices. We say that the coupling is successful if $\Omega _0^X(K)$ holds (note that this only depends on *X* up to the first time it hits level *K*) and the coupling from the level *K* vertices is also successful. Otherwise let us say that the coupling fails. Let $\Omega _2^X$ denote the event that the coupling is successful.

#### Lemma 4.11

Let $T_0$ be a possible realisation of the first *K* levels of *T*. For each *i* let $D_i$ be the set of vertices explored during the exploration process from the set $V_{z_i}$. Then for all sufficiently large values of *n*, for any realisation of $(D_i)$ we have

$$\left| \bigcup D_i\right| \le N:= n\exp \left( -\frac{1}{3}A\sqrt{\frac{\mathfrak {V}\log n}{\mathfrak {h}}}\right) .$$

Also there exists a positive constant *C* (not depending on $T_0$) so that the number $\textrm{Bad}$ of bad vertices *z* satisfies

$$\begin{aligned} \mathbb {P} \!\Big (\mathrm{{Bad}}\ge C\sqrt{\frac{\log n}{\mathfrak {h}}} \;\Big \vert \;\mathcal {B}_K^*(x_0)=T_0\Big )\le o\left( \frac{1}{n^2}\right) . \end{aligned}$$

#### Proof

The proof of the bound on $\left| \bigcup D_i\right|$ is analogous to the proof in Lemma 3.22. For the bound on the number of bad vertices we get that

$$\begin{aligned}&\mathbb {P} \!\Big (\mathrm{{Bad}}\ge C\sqrt{\frac{\log n}{\mathfrak {h}}} \;\Big \vert \;T_0\Big )\le \left( {\begin{array}{c}N\\ C\sqrt{\frac{\log n}{\mathfrak {h}}}\end{array}}\right) \left( \frac{b(R)^{K+1}}{n}\right) ^{C\sqrt{\frac{\log n}{\mathfrak {h}}}}\\&\le \exp \left( C\sqrt{\frac{\log n}{\mathfrak {h}}}\left( \log n-\frac{1}{3}A\sqrt{\frac{\mathfrak {V}\log n}{\mathfrak {h}}}+2K\log b(R)-\log n\right) \right) \ll \frac{1}{n^2} \end{aligned}$$

if *C* is sufficiently large, since

$$\begin{aligned}K\log b(R) \lesssim \log g(n)\frac{\log n}{g(n)}\log g(n)&\ll \frac{\log n}{\sqrt{g(n)}} \asymp \sqrt{\frac{\mathfrak {V}\log n}{\mathfrak {h}}},\qquad \text {and}\\ \sqrt{\frac{\log n}{\mathfrak {h}}}\sqrt{\frac{\mathfrak {V}\log n}{\mathfrak {h}}}&\asymp \log n. \end{aligned}$$

$$\square $$

#### Lemma 4.12

Let us consider the setup of Definition 4.10 and consider the coupling of *X* with $\widetilde{X}$ from $u\in V_{z_i}$. Then for all $\theta >0$ there exist *B* and *A* sufficiently large such that for all large enough *n* we have

$$\begin{aligned} \mathbb {P}_{u}\!\Big (\text {the coupling of } X \text { and } \widetilde{X} \text { succeeds}\;\Big \vert \;\mathcal {F}_{i-1}\Big ) \ge (1-\theta ){{{\mathfrak {1}}}}_{\{z_i\text { good}\}}. \end{aligned}$$

#### Proof

Analogous to the proof of Lemma 3.23. $\square$

#### Proposition 4.13

For any $\theta \in (0,1)$, for a sufficiently large constant $C>0$, with high probability the mixing time of the random walk on $G^*$ satisfies

$$\begin{aligned} t_{\textrm{mix}}\left( \theta \right) \ge t_0-Ct_w \end{aligned}$$

for all sufficiently large *n*.

#### Proof

Analogously to the proof of the lower bound in [18, Theorem 1.1], we use Lemmas 4.11 and 4.12 to show that up to time $t_0-Ct_w$ the walk is likely to stay in the explored region, and this region only contains a small proportion of the vertices of $G^*$. $\square$

## Specific graphs

In this section we prove that for graphs as in Theorems 1, 2, 3, and 4, and $\varepsilon _n$ satisfying the lower bound in part (a) and $\varepsilon _n\ll (\log n)^{-\beta }$ for some $\beta >0$, Assumption 3.1 is satisfied with specific functions *f* and *g*. Then we use the results from Sect. 4 to deduce cutoff in this regime.

### Graphs with polynomial growth of balls

#### Proposition 5.1

Let us assume that *G* and $\varepsilon$ satisfy the following. For any *t* the size of all balls of radius *t* are upper bounded by $ct^d$ where $c,d>0$ are constants; $\varepsilon _n\lesssim (\log n)^{-\beta }$ for some constant $\beta >0$; and $\log (\varepsilon )\gg -\log n$ (i.e. $\varepsilon =n^{-o(1)}$). Then for any $\theta \in \left( 0,1\right)$, for a sufficiently large constant $C>0$, whp the mixing time of the random walk on $G^*$ satisfies

$$\begin{aligned} \left| t_{\textrm{mix}}^{G^*}(\theta )-t_0\right| \le Ct_w \end{aligned}$$

for all sufficiently large *n*, where $t_0\asymp \frac{1}{\varepsilon }\frac{\log n}{\log \left( \frac{1}{\varepsilon }\right) }$ and $t_w\asymp \frac{1}{\varepsilon }\sqrt{\frac{\log n}{\log \left( \frac{1}{\varepsilon }\right) }}$. This means that the chain exhibits cutoff around $t_0$ with window $t_w$.

#### Proof

Let $g(n)=\frac{\log n}{\log \left( \frac{1}{\varepsilon }\right) }$ and let $f(t)=\log (1+t)$. Note that $f(t)\asymp \log t$ for $t\ge 2$. Then by assumption $g(n)\rightarrow \infty$ as $n\rightarrow \infty$ and $f\left( \frac{1}{\varepsilon }\right) \asymp \frac{\log n}{g(n)}$.

Also for any constant $\widehat{c}$ we have $\log \left( 1+\frac{\widehat{c}}{\varepsilon }\right) \asymp \log \left( \frac{1}{\varepsilon }\right) +\log {\widehat{c}}\asymp \log \left( \frac{1}{\varepsilon }\right)$, since $\frac{1}{\varepsilon }\gg 1$.

For any $x,y>e^2-1$ we have $\log \left( 1+xy\right) \le \log (1+x)+\log (1+y)\le$

$$2\max \{\log (1+x),\log (1+y)\}\le \log (1+x)\log (1+y)$$.

We have already assumed that the sizes of balls of radius *t* are upper bounded by *b*(*t*) where $b(t)\asymp t^d$, hence $\log b(t)\asymp \log t\asymp f(t)$.

Let *X* be a random walk on *G*. We will show that for all $t\lesssim \frac{1}{\varepsilon }$ and all $b\in \mathbb {Z}_{\ge 1}$ we have $H_b(X_t)\asymp (\log t)^b\asymp f(t)^b$. From Lemma A.4 (iv) we immediately get that

$$\begin{aligned} H_b(X_t) \le \left( \log b(t)\right) ^b \lesssim \left( \log t\right) ^b \asymp f(t)^b. \end{aligned}$$

From Lemma A.2 we know that for all $t\lesssim n^2$ and any *u* we have $\mathbb {P}_{v}\!\left( X_t=u\right) \lesssim \frac{1}{\sqrt{t}}$. Note that $\frac{1}{\varepsilon }=n^{o(1)}\ll n^2$ for all sufficiently large *n*. This gives that for any $t\le \frac{C}{\varepsilon }$ we have

$$\begin{aligned}  &   H_b(X_t)=\sum _{u}\mathbb {P}_{v}\!\left( X_t=u\right) \left( -\log \mathbb {P}_{v}\!\left( X_t=u\right) \right) ^b\\  &   \quad \ge \sum _{u}\mathbb {P}_{v}\!\left( X_t=u\right) \left( -\log \left( \frac{c'}{\sqrt{t}}\right) \right) ^b\asymp \left( \log t\right) ^b. \end{aligned}$$

This means that Assumption 3.1 holds with $f(t)=\log (1+t)$ and $g(n)=\frac{\log n}{\log \left( \frac{1}{\varepsilon }\right) }$. Propositions 4.8 and 4.13 give the result. $\square$

### Graphs with linear growth of entropy

#### Proposition 5.2

Let us assume that *G* and $\varepsilon$ satisfy the following. For any vertex *x* and any $t\le c\log n$ (where *c* is some positive constant), the entropy of a simple random walk *X* on *G* starting from *x* satisfies $H_1(X_t)\gtrsim t$; and $(\log n)^{-1}\ll \varepsilon _n\lesssim (\log n)^{-\beta }$ for some constant $\beta >0$. Then for any $\theta \in \left( 0,1\right)$, for a sufficiently large constant $C>0$, whp the mixing time of the random walk on $G^*$ satisfies

$$\begin{aligned} \left| t_{\textrm{mix}}^{G^*}(\theta )-t_0\right| \le Ct_w \end{aligned}$$

for all sufficiently large *n*, where $t_0\asymp \log n$ and $t_w\asymp \sqrt{\frac{\log n}{\varepsilon }}$.

This means that the chain exhibits cutoff around $t_0$ with window $t_w$.

#### Proof

Let $g(n)=\varepsilon \log n$ and let $f(t)=t$. Then by assumption $g(n)\rightarrow \infty$ as $n\rightarrow \infty$ and $f\left( \frac{1}{\varepsilon }\right) =\frac{\log n}{g(n)}$.

Also $f\left( \frac{\widehat{c}}{\varepsilon }\right) \asymp f\left( \frac{1}{\varepsilon }\right)$ for any constant $\widehat{c}$, and $f(xy)=f(x)f(y)$ for any $x,y>0$.

Since each vertex has degree $\le \Delta$, the sizes of balls of radius *t* are upper bounded by $b(t):=\Delta ^{t+1}$, which gives $\log b(t)\asymp t\asymp f(t)$.

Let *X* be a random walk on *G*. We will show that for all $t\lesssim \frac{1}{\varepsilon }\ll \log n$ and all $b\in \{1,2,4\}$ we have $H_b(X_t)\asymp (\log t)^b\asymp f(t)^b$. From Lemma A.4 (iv) we immediately get that for any $b\in \mathbb {Z}_{\ge 1}$ we have

$$\begin{aligned} H_b(X_t)\le \left( \log b(t)\right) ^b \lesssim t^b \asymp f(t)^b. \end{aligned}$$

By assumption we also have $H_1(X_t)\gtrsim t$, which by Cauchy-Schwarz also gives $H_2(X_t)\gtrsim t^2$ and $H_4(X_t)\gtrsim t^4$.

This means that Assumption 3.1 holds with $f(t)=t$ and $g(n)=\varepsilon \log n$. Propositions 4.8 and 4.13 give the result. $\square$

Now we give some examples of notable families of graphs covered by Proposition 5.2.

#### Proposition 5.3

For any positive constants *a* and *b* there exist positive constants *C* and *c* with the following property. If the graph *G* is such that for any set $A\subseteq V$ of size $\le n^a$ we have $|\partial A|\ge b|A|$ (where $\partial A$ denotes the set of edges between *A* and $V{\setminus } A$) then for any vertex *x* of *G* and any $t\le C\log n$, the entropy of a simple random walk *X* on *G* starting from *x* satisfies $H_1(X_t)\ge ct$.

#### Remark 5.4

This means that families of locally expanding graphs (in the above sense) satisfy the condition of Proposition 5.2. These include in particular families of expanders.

#### Proof

Using [22, Theorem 2] for the lazy random walk on *G*, the assumption on the size of boundaries of sets, and that $\pi (x)\asymp \frac{1}{n}$ for all *n*, we get that there exist positive constants $c_1$, $c_2$ and $c_3$ such that

$$\begin{aligned} P^t_{G,\textrm{lazy}}(x,y)\le c_2\exp \left( -c_3t\right) \qquad \forall t\le c_1\log n,\quad \forall x,y\in V. \end{aligned}$$

Then using that $P_{G}^k(x,y)\le P_{G}^{k-2}(x,y)$ and that $\max _{x,y}P_{G}^{k}(x,y)\asymp \max _{x,y}P_{G}^{k-1}(x,y)$ we get that

$$\begin{aligned} \max _{x,y}P^t_{G,\textrm{lazy}}(x,y) = \max _{x,y}\sum _{k=0}^{t}\mathbb {P}\!\left( \textrm{Bin}\!\left( t,\frac{1}{2}\right) =k\right) P_{G}^k(x,y) \gtrsim \max _{x,y}P_{G}^{t/2}(x,y). \end{aligned}$$

Since for $t\le \frac{1}{2}c_1\log n$ the transition probabilities of $P_{G}$ are exponentially small in *t*, we also get that the entropy $H_1(X_t)$ is growing linearly in *t*. (We use that the entropy is lower bounded by the log of the inverse of the maximal transition probability, and upper bounded by log of the number of the achievable states.) $\square$

#### Definition 5.5

For a finite connected simple graph $K=(V(K),E(K))$, the corresponding lamplighter graph $G=(V(G),E(G))$ is defined as follows. Let $V(G)=\{0,1\}^{V(K)}\times V(K)$ and for $(\sigma ,u),(\rho ,v)\in V(G)$ let $\left\{ (\sigma ,u),(\rho ,v)\right\} \in E(G)$ if and only if $\{u,v\}\in E(K)$ and $\sigma (w)=\rho (w)$ for all $w\in V(K){\setminus }\{u,v\}$.^11^

Note that the maximal degree of *G* is 4 times the maximal degree of *K*.

For a path *y* on *V*(*K*) and a given time *t*, let $R_t(y):=\{y_0,...,y_t\}$ denote the range of *y* up to time *t*.

#### Proposition 5.6

For any positive constants $c_1$ and $c_2$ there exist positive constants $c_3$ and $c_4$ with the following property. If a graph *K* is such that for any $t\le c_1|V(K)|$ and any vertex *v* in *K*, the expected size of the range of a random walk *Y* from *v* up to time *t* satisfies $\mathbb {E}_{v}\left| R_t(Y)\right| \ge c_2t$ then the corresponding lamplighter graph *G* is such that for any $t\le c_3\log |V(G)|$ and any $x\in V(G)$ the entropy of a simple random walk *X* on *G* starting from *x* satisfies $H_1(X_t)\ge c_4t$.

#### Proof

Let *X* be a simple random walk on *G* and let *Y* be the corresponding simple random walk on *K*.

Note that for any starting state $X_0=(\sigma ,v)$, conditional on $(Y_0,...,Y_t)=(y_0,...,y_t)$ the state $X_t$ is uniform among all states $(\rho ,u)$ where $u=y_t$ and $\rho (w)=\sigma (w)$ for all $w\in V(K)\setminus R_t(y)$. There are $2^{|R_t(y)|}$ such states in total, so we have

$$\begin{aligned} H_1(X_t|Y_0=y_0,...,Y_t=y_t) = \log \left( 2^{|R_t(y)|}\right) \asymp |R_t(y)|. \end{aligned}$$

Therefore

$$\begin{aligned} H_1(X_t)\ge H_1(X_t|Y_0,...,Y_t) \asymp \mathbb {E}_{v}\left| R_t(Y)\right| . \end{aligned}$$

For $t\le c_1|V(K)|\asymp \log |V(G)|$ this is $\gtrsim t$ by assumption. This finishes the proof. $\square$

#### Remark 5.7

This means that if $(K_n)$ is a sequence of graphs with bounded degrees and linearly growing range (in the above sense), then the sequence $(G_n)$ of the corresponding lamplighter graphs satisfies the condition of Proposition 5.2.

#### Remark 5.8

One example of graphs $(K_n)$ satisfying the condition of Proposition 5.6 is $K_n=\mathbb {Z}_n^d$ where $d\ge 3$ is fixed. Note that the corresponding family $(G_n)$ is not locally expanding, so it is not covered by Proposition 5.3.

### Remark on other functions *f*

Note that for any graph *G* with *n* vertices and degrees bounded by $\Delta$ and any $a\in \{1,2,4\}$, a random walk *X* on *G* and $b(t):=\sup _{x}\left| \mathcal {B}_{G}(x,t)\right|$ satisfies

$$\begin{aligned} (\log t)^a \lesssim H_a(X_t) \lesssim \left( \log b(t)\right) ^a \lesssim t^a. \end{aligned}$$

It means that if we want to ensure

5.1 $$\begin{aligned} H_a(X_t) \asymp \left( \log b(t)\right) ^a \asymp f(t)^a \end{aligned}$$

with $f(t)=\log t$, it is sufficient to assume $\log b(t)\lesssim \log t$, while to ensure (5.1) with $f(t)=t$, it is sufficient to assume $H_1(X_t)\gtrsim t$.

In case we want (5.1) to hold with a function $\log t\ll f(t)\ll t$, we need to assume both a lower bound $H_1(X_t)\gtrsim f(t)$ and an upper bound $\log b(t)\lesssim f(t)$. It is less straightforward to see that graphs (and especially graphs of interest) satisfying these assumptions exist.

## Other values of $\varepsilon$

### Larger values

In Assumption 3.1 we only considered weights $(\varepsilon _n)$ satisfying $\varepsilon _n\lesssim (\log n)^{-\beta }$ for some $\beta >0$. Now we cover the case $(\log n)^{-\frac{1}{3}}\ll \varepsilon _n\ll 1$, and then the case $\varepsilon _n\asymp 1$.

#### Assumption 6.1

This is the same as Assumption 3.1, except that (iv) changes to

(iv’) $$(\log n)^{-\frac{1}{3}}\ll \varepsilon _n\ll 1$$.

#### Proposition 6.2

Let us assume that Assumption 6.1 holds. Then for any $\theta \in \left( 0,1\right)$ whp the mixing time of the random walk on $G^*$ satisfies

$$\begin{aligned} \left| t_{\textrm{mix}}^{G^*}(\theta )-t_0\right| \ll t_0 \end{aligned}$$

where $t_0\asymp \frac{1}{\varepsilon }g(n)$ as in Definition 3.3. This means that the chain exhibits cutoff around $t_0$.

#### Proof

Let us recall $t_w$, *R*, *K* and *M* from Definition 3.3. Let $t=t_0-Bt_w$ and $\widetilde{L}=\nu t+2D\sqrt{\varepsilon t}$ as in Sect. 4.3.

Let us use the same truncation criterion and exploration process as in Sect. 4.3. It can be checked that in this case the results in Sect. 4.3 still hold. This immediately gives the lower bound.

Analogously to [18, Proposition 5.7] we also get the following.

#### Proposition 6.3

For all $\theta >0$, there exist *B* (in the definition of *t*), *A* (in the definition of the truncation criterion) depending on $\theta$ and *B* and a positive constant $\Gamma$ sufficiently large such that for all *n* sufficiently large, on the event $\{\mathcal {B}^*_K(x_0)=T_0\}$, for all *i* and all $x\in \partial \mathcal {B}_K^*(x_0)$ descendants of $z_i\in \partial \mathcal {B}_{K/2}^*(x_0)$, on the event $\{z_i \text { is good}\}$ we have for all $s \ge 0$ that

$$\begin{aligned} \mathbb {P} \!\Big ({d}_x(t+s)<e^{-\tfrac{s}{t_{\textrm{rel}}(G^*)}}\cdot \frac{1}{1-\theta } \exp \left( \Gamma \sqrt{\frac{\mathfrak {V}\log n}{\mathfrak {h}}}\right) + \theta \;\Big \vert \;\mathcal {F}_{i-1}\Big )\ge 1-2\theta , \end{aligned}$$

where ${d}_x(r)=d_{\textrm{TV}}\left( {\mathbb {P}_{x}\!\Big ({X}_r\in \cdot \;\Big \vert \;G^*\Big )},{\pi }\right)$ for every $r\in \mathbb {Z}_{\ge 1}$.

Since $t_{\textrm{rel}}(G^*)\lesssim \frac{1}{\varepsilon }$ and $g(n)\gg (\log n)^{\frac{2}{3}}$, it is possible to choose *s* with $t_{\textrm{rel}}(G^*)\frac{\log n}{\sqrt{g(n)}}\ll s\ll \frac{1}{\varepsilon }g(n)$. This then gives $e^{-\tfrac{s}{t_{\textrm{rel}}(G^*)}}\cdot \frac{1}{1-\theta } \exp \left( \Gamma \sqrt{\frac{\mathfrak {V}\log n}{\mathfrak {h}}}\right) \ll 1$. We can finish the proof of the upper bound on the mixing time following the same steps as the proof of [18, Theorem 1.1], and we get an upper bound of the form $t_0+s$ where $s\ll t_0$. $\square$

#### Proposition 6.4

Let us assume that $\varepsilon _n\asymp 1$ and the graphs $G_n$ are connected, have bounded degree and $G_n$ has *n* vertices. Then for any $\theta \in (0,1)$ whp the mixing time of the random walk on $G^*$ satisfies

$$\begin{aligned} \left| t_{\textrm{mix}}^{G^*}(\theta )-t_0\right| \ll t_0 \end{aligned}$$

where $t_0\asymp \log n$. This means that the chain exhibits cutoff around $t_0$.

#### Proof

The proof is exactly the same as for the $\varepsilon _n\equiv 1$ case in [18], except for the proof of [18, Lemma 3.3]. This result can be proved by using the result for the $\varepsilon _n\equiv 1$ case and noting that in the case $\varepsilon _n\asymp 1$ the effective resistances change by a constant factor. $\square$

### Smaller values

#### Proposition 6.5

Let us assume that $\varepsilon \ll \frac{1}{t_{\textrm{mix}}^G(\theta )}$ for some $\theta \in (0,1)$. Then the random walk on $G^*$ exhibits cutoff if and only if the random walk on *G* does.

The proof of this is not difficult and is deferred to [1].

The following proposition will make use of a comparison between hitting and mixing times. We recall that

$$\begin{aligned}\textrm{hit}_{\alpha }\left( \theta \right) =\min \left\{ s:\forall x,\forall A\text { with }\pi (A)\ge \alpha \text { we have }\mathbb {P}_{x}\!\left( \tau _A>s\right) \le \theta \right\} \end{aligned}$$

and we let $\textrm{hit}_{\alpha }=\textrm{hit}_{\alpha }\left( \frac{1}{4}\right)$. We say that $\textrm{hit}_{\alpha }$-cutoff occurs if we have $\left| \textrm{hit}_{\alpha }\left( \theta \right) -\textrm{hit}_{\alpha }\right| \ll \textrm{hit}_{\alpha }$ for all $\theta \in (0,1)$.

#### Proposition 6.6

Let us assume that the lazy random walk on the original graph *G* does not exhibit cutoff and that $\varepsilon \asymp \frac{1}{t_{\textrm{mix}}^{G,\textrm{lazy}}}$. Then the random walk on $G^*$ does not exhibit cutoff either.

#### Proof

Assume that the lazy random walk on *G* does not exhibit cutoff, but the random walk on $G^*$ exhibits cutoff. Then by [2, Remark 1.9] we have $t_{\textrm{rel}}^{\text {abs},G^*}\ll t_{\textrm{mix}}^{G^*}$, and by this and [2, equation (1.4)] (which holds with $t_{\textrm{rel}}^{\text {abs},G^*}$ by Remark 1.9) the random walk on $G^*$ exhibits $\textrm{hit}_{\frac{1}{2}}$-cutoff. Then by [2, Corollary 3.4] it also exhibits $\textrm{hit}_{\alpha }$-cutoff for all $\alpha \in \left( 0,\frac{1}{2}\right)$ and $\textrm{hit}^{G^*}_{\alpha _1}(u_1)=(1\pm o(1))\textrm{hit}^{G^*}_{\alpha _2}(u_2)$ for any $\alpha _1,\alpha _2\in \left( 0,\frac{1}{2}\right)$, $u_1,u_2\in (0,1)$. By Lemma A.3 we have $\textrm{hit}_{\alpha }^{\textrm{lazy},G^*}(u)=(1\pm o(1))\frac{1}{2}\textrm{hit}_{\alpha }^{G^*}((1\pm o(1))u)$ for all $\alpha$ and all *u*, hence

the lazy walk on $G^*$ also exhibits $\textrm{hit}_{\alpha }$-cutoff for all $\alpha \in \left( 0,\frac{1}{2}\right)$ and satisfies

6.1 $$\begin{aligned} \textrm{hit}^{\textrm{lazy},G^*}_{\alpha _1}(u_1)=(1\pm o(1))\textrm{hit}^{\textrm{lazy},G^*}_{\alpha _2}(u_2)\qquad \forall \alpha _1,\alpha _2\in \left( 0,\frac{1}{2}\right) ,u_1,u_2\in (0,1). \end{aligned}$$

Let *X* be a lazy random walk on *G* and *Y* be a lazy random walk on $G^*$. Let $\tau _{\textrm{LR}}$ be the first time that *Y* crosses a long-range edge. Since *X* does not exhibit cutoff, by [2, Theorem 3] it does not exhibit $\text {hit}_{\alpha }$-cutoff for any $\alpha \in \left( 0,\frac{1}{2}\right)$ either. Therefore for any $\alpha \in \left( 0,\frac{1}{2}\right)$ there exists $\theta \in (0,1)$ such that along a subsequence (of the graphs $G_n$) we have

$$\begin{aligned} \text {hit}^X_\alpha (\theta )-\text {hit}^X_\alpha (1-\theta )\asymp \text {hit}^X_\alpha (\theta ). \end{aligned}$$

If $t_{\textrm{rel}}^{X}\ll t_{\textrm{mix}}^{X}$ then by [2, Proposition 1.8] and [2, Corollary 3.4] we have $\text {hit}^X_\alpha (\theta )\asymp t_{\textrm{mix}}^{X}$. If $t_{\textrm{rel}}^{X}\asymp t_{\textrm{mix}}^{X}$ then by [2, Corollary 3.1] and the last display equation in the proof of [2, Proposition 3.7] we still have $\text {hit}^X_\alpha (\theta )\asymp t_{\textrm{mix}}^{X}$.

Let $t=\text {hit}^X_\alpha (\theta )-1$. Then there exist a vertex *x* and a set *A* such that $\pi ^X(A)\ge \alpha$ and $\mathbb {P}_{x}\!\left( \tau ^X_A>t\right) >\theta$. For any vertex *z* we have $\pi ^Y(z)=\frac{\deg (z)}{2E}=\frac{\deg (z)+\varepsilon }{2E+n\varepsilon }(1\pm o(1))=\pi ^X(z)(1\pm o(1))$, therefore $\pi ^Y(A)\ge \frac{1}{2}\alpha$ for all sufficiently large values of *n*.

Note that $t\asymp t_{\textrm{mix}}^X\asymp \frac{1}{\varepsilon }$. For any vertices *y* and *z* that are adjacent in *G* we have

$$\begin{aligned}\mathbb {P}_{y}\!\left( Y_1=z,\tau _{\textrm{LR}}>1\right) =\frac{1}{\deg (y)+\varepsilon }=(1-O(\varepsilon ))\frac{1}{\deg (y)}=(1-O(\varepsilon ))\mathbb {P}_{y}\!\left( X_1=z\right) .\end{aligned}$$

Summing this over all paths *p* of length *t* in *G* that start in *x* and do not visit *A*, we get that

$$\begin{aligned}  &   \mathbb {P}_{x}\!\left( \tau ^Y_A>t\right) \ge \mathbb {P}_{x}\!\left( \tau ^Y_A>t,\tau _{\textrm{LR}}>t\right) =(1-O(\varepsilon ))^t\\  &   \quad \mathbb {P}_{x}\!\left( \tau ^X_A>t\right) \asymp \mathbb {P}_{x}\!\left( \tau ^X_A>t\right) >\theta , \end{aligned}$$

therefore there exists $c_1>0$ such that

$$\begin{aligned} \text {hit}^Y_{\frac{1}{2}\alpha }(c_1\theta )\ge \text {hit}^X_{\alpha }(\theta ). \end{aligned}$$

Now let $s=\text {hit}^X_\alpha (1-\theta )$ and let *x* be any vertex and *A* be any set with $\pi ^Y(A)\ge \frac{1}{2}\alpha +\frac{1}{4}$. Then we also have $\pi ^X(A)\ge \alpha$, hence

$$\begin{aligned} \mathbb {P}_{x}\!\left( \tau ^Y_A\le s\right) \ge \mathbb {P}_{x}\!\left( \tau ^Y_A\le s,\tau _{\textrm{LR}}>s\right) \asymp \mathbb {P}_{x}\!\left( \tau ^X_A\le s\right) \ge \theta . \end{aligned}$$

This shows that there exists $c_2>0$ such that

$$\begin{aligned} \text {hit}^Y_{\frac{1}{2}\alpha +\frac{1}{4}}(1-c_2\theta )\le \text {hit}^X_{\alpha }(1-\theta ). \end{aligned}$$

Together these give

$$\begin{aligned} \text {hit}^Y_{\frac{1}{2}\alpha }(c_1\theta )-\text {hit}^Y_{\frac{1}{2}\alpha +\frac{1}{4}}(1-c_2\theta )\asymp \text {hit}^Y_{\frac{1}{2}\alpha }(c_1\theta ), \end{aligned}$$

contradicting (6.1). $$\square $$

### Completing the picture for expanders

#### Proposition 6.7

There exists a sequence of expander graphs *G* such that the simple random walk on *G* exhibits cutoff, but for any $\varepsilon \asymp \frac{1}{\log n}$ whp the simple random walk on $G^*$ does not exhibit cutoff.

#### Proof

Let *G* be the 5-regular expander constructed in the proof of [21, Theorem 1], with a sufficiently large value of the parameter *L* in the construction. Then the simple random walk on *G* has cutoff.

Let *X* and *Y* be simple random walks on *G* and $G^*$, respectively. Let $\tau _{\textrm{LR}}$ and $\tau _{\textrm{LR}}^{(2)}$ be the first and second time, respectively when *Y* crosses a long-range edge. Also let $\tau ^X_{\ell }$ be the first time that *X* reaches level $3h+2$ and $\tau ^Y_{\ell }$ be the first time that *Y* reaches level $3h+2$.

From [21] we know that the mixing time of *X* is $t_{\textrm{mix}}^X(\theta )=(1\pm o(1))t_0$ for all $\theta \in (0,1)$, where $t_0=\frac{5}{3}\left( 5L^2-3L+1\right) h$. Let *L* be sufficiently large so that $t_0\ge 8L^2h$. We also know that for any vertex *v* at level $>h$, the mixing time of *X* started from *v* satisfies $t_{\textrm{mix}}^X(x;\theta )<6L^2h$ for all $\theta \in (0,1)$.

Since $\varepsilon \asymp \frac{1}{\log n}\asymp h$, we have $\mathbb {P}_{\rho }\!\Big (\tau _{\textrm{LR}}>2t_0\;\Big \vert \;G^*\Big )\ge \theta _1$ for some $\theta _1\in (0,1)$. By the regularity of *G* we also have $(Y_k)_{k\le 2t_0}\big |\left\{ Y_0=\rho ,\tau _{\textrm{LR}}>2t_0\right\} {\mathop {=}\limits ^{\textrm{d}}}(X_k)_{k\le 2t_0}\big |\left\{ X_0=\rho \right\}$. From [21, Claim 2.3] we know that starting *X* from the root $\rho$ we have $\tau ^X_\ell =(1+o(1))t_0$ whp. Together these show that for any realisation of $G^*$ and for any constant $a\in (0,1)$ we have

$$\begin{aligned}&\mathbb {P}_{\rho }\!\Big (\tau ^Y_{\ell }>(1-a)t_0\;\Big \vert \;G^*\Big ) \ge \mathbb {P}_{\rho }\!\Big (\tau ^Y_{\ell }>(1-a)t_0,\tau _{\textrm{LR}}>2t_0\;\Big \vert \;G^*\Big )\\&\quad =\mathbb {P}_{\rho }\!\Big (\tau _{\textrm{LR}}>2t_0\;\Big \vert \;G^*\Big )\mathbb {P}_{\rho }\!\left( \tau ^X_{\ell }>(1-a)t_0\right) \ge \,\theta _1(1-o(1)). \end{aligned}$$

Level $3h+2$ contains at least a constant $b>0$ proportion of all vertices, so this implies

6.2 $$\begin{aligned} \textrm{hit}^Y_{b}\left( \frac{1}{2}\theta _1\right) \ge (1-o(1))t_0. \end{aligned}$$

For any vertex *x* at level $>h$ and any $t<2t_0$ we have

$$\begin{aligned}&d_{\textrm{TV}}\left( {\mathbb {P}_{x}\!\Big (Y_t=\cdot \;\Big \vert \;G^*\Big )},{\pi (\cdot )}\right) \\&\quad \le \mathbb {P}_{x}\!\Big (\tau _{\textrm{LR}}\le 2t_0\;\Big \vert \;G^*\Big )d_{\textrm{TV}}\left( {\mathbb {P}_{x}\!\Big (Y_t=\cdot \;\Big \vert \;G^*;\tau _{\textrm{LR}}\le 2t_0\Big )},{\pi (\cdot )}\right) \\&\qquad +\mathbb {P}_{x}\!\Big (\tau _{\textrm{LR}}>2t_0\;\Big \vert \;G^*\Big )d_{\textrm{TV}}\left( {\mathbb {P}_{x}\!\Big (Y_t=\cdot \;\Big \vert \;G^*;\tau _{\textrm{LR}}>2t_0\Big )},{\pi (\cdot )}\right) \\&\quad \le (1-\theta _1)+\theta _1d_{\textrm{TV}}\left( {\mathbb {P}_{x}\!\left( X_t=\cdot \right) },{\pi (\cdot )}\right) . \end{aligned}$$

In particular this shows that

6.3 $$\begin{aligned} t_{\textrm{mix}}^Y\left( x;1-\frac{1}{2}\theta _1\right) \le t_{\textrm{mix}}^X\left( x;\frac{1}{2}\right) < 6L^2h\qquad \text {for all }x\text { at level }>h. \end{aligned}$$

Now we will also upper bound the mixing time from vertices at level $\le h$.

Note that levels 0 to $\frac{5}{4}h$ in *G* contain $\asymp 4^{\frac{5}{4}h}$ vertices and the total number of vertices is $\asymp 4^{3h}$. Therefore the probability that $G^*$ has any long-range edges between two vertices at level $\le \frac{5}{4}h$ is $\lesssim 4^{-\frac{1}{2}h}=o(1)$. So whp $G^*$ is such that all long-range edges starting from a level $\le \frac{5}{4}h$ lead to a level $>\frac{5}{4}h$.

Note that we have $\mathbb {P} \!\Big (\tau _{\textrm{LR}}<\frac{1}{4}h,\tau _{\textrm{LR}}^{(2)}>7L^2h\;\Big \vert \;G^*\Big )\ge \theta _2$ for some $\theta _2\in (0,1)$. By the strong Markov property and by the regularity of *G*, for any vertex *v* we have

$$\begin{aligned} (Y_k)_{k=\tau _{\textrm{LR}}}^{\tau _{\textrm{LR}}+6L^2h}\bigg |\left\{ \tau _{\textrm{LR}}<\frac{1}{4}h,\tau _{\textrm{LR}}^{(2)}>7L^2h,Y_{\tau _{\textrm{LR}}}=v\right\} {\mathop {=}\limits ^{\textrm{d}}}(X_k)_{k=0}^{6L^2h}\big |\left\{ X_0=v\right\} . \end{aligned}$$

Let $t=6\,L^2\,h+\frac{1}{4}h$ and let *U* be the set of vertices at levels $>\frac{5}{4}h$. Then on the above high probability event for any vertex *x* at level $\le h$ we have

$$\begin{aligned}&d_{\textrm{TV}}\left( {\mathbb {P}_{x}\!\Big (Y_t=\cdot \;\Big \vert \;G^*\Big )},{\pi (\cdot )}\right) \\&\quad \le \left( 1-\mathbb {P}_{x}\!\Big (\tau _{\textrm{LR}}<\frac{1}{4}h,\tau _{\textrm{LR}}^{(2)}>7L^2h\;\Big \vert \;G^*\Big )\right) \\&\qquad +\sum _{u\in U,s\le \frac{1}{4}h}\mathbb {P}_{x}\!\Big (\tau _{\textrm{LR}}=s,\tau _{\textrm{LR}}^{(2)}>7L^2h,Y_{\tau _{\textrm{LR}}}=u\;\Big \vert \;G^*\Big )d_{\textrm{TV}}\left( {\mathbb {P}_{u}\!\left( X_{t-s}=\cdot \right) },{\pi (\cdot )}\right) \\&\quad \le (1-\theta _2)+\theta _2\cdot \frac{1}{2}. \end{aligned}$$

In particular this shows that with high probability $G^*$ is such that

6.4 $$\begin{aligned} t_{\textrm{mix}}^Y\left( x;1-\frac{1}{2}\theta _2\right) < 6L^2h+\frac{1}{4}h\qquad \text {for all }x\text { at level }\le h. \end{aligned}$$

Assume that *Y* exhibits cutoff. Then we have $t_{\textrm{rel}}^{\text {abs},Y}\ll t_{\textrm{mix}}^Y$ and we have $t_{\textrm{mix}}^Y(\theta )=(1\pm o(1))t_{\textrm{mix}}^Y$ for any $\theta \in (0,1)$. By [2, Remark 1.9] and [2, Corollary 3.4] we also have $\textrm{hit}^Y_{\alpha }(\theta )=(1\pm o(1))t_{\textrm{mix}}^Y$ for any $\alpha ,\theta \in (0,1)$. Then (6.2), (6.3) and (6.4) would give a contradiction. So *Y* does not exhibit cutoff. $\square$

#### Proposition 6.8

There exists a sequence of expander graphs *G* such that the simple random walk on *G* exhibits cutoff and for any $\varepsilon \asymp \frac{1}{\log n}$ whp the simple random walk on $G^*$ also exhibits cutoff.

#### Proof

First let us consider a random sequence *G*, the sequence of random 3-regular graphs. From [20, Theorem 1] we know that whp the simple random walk on *G* exhibits cutoff.

It can be proved using the methods of [6, 18] that the simple random walk on $G^*$ also exhibits cutoff whp. (In this case *T* will be a weighted tree where each vertex has three edges of weight 1 and one edge of weight $\varepsilon$.) This shows that whp *G* is such that the random walk on $G^*$ exhibits cutoff whp.

Also, *G* is an expander whp (see e.g. [14]).

Combining these we get that whp *G* has the required properties, in particular we can choose a non-random sequence *G* that also has the required properties. $\square$

#### Lemma 6.9

Assume that the simple random walk on a sequence of graphs *G* exhibits cutoff. Then the lazy random walk on *G* also exhibits cutoff.

Propositions 6.7 and 6.8 and Lemma 6.9 immediately imply the following.

#### Corollary 6.10

There exists a sequence of expander graphs *G* such that the lazy random walk on *G* exhibits cutoff and for any $\varepsilon \asymp \frac{1}{\log n}$ the simple random walk on $G^*$ whp does not exhibit cutoff.

Also there exists a sequence of expander graphs *G* such that the lazy random walk on *G* exhibits cutoff and for any $\varepsilon \asymp \frac{1}{\log n}$ the simple random walk on $G^*$ whp also exhibits cutoff.

#### Proof of Lemma 6.9

Let *X* be a simple random walk and let *Y* be a lazy random walk on *G*.

By [2, Remark 1.9] we know that $t_{\textrm{rel}}^{\textrm{abs}}\ll t_{\textrm{mix}}^X$ and that *X* exhibits $\textrm{hit}_{\alpha }$-cutoff for some $\alpha \in (0,1)$.

Then by Lemma A.3^12^ the walk *Y* also exhibits $\textrm{hit}_{\alpha }$-cutoff and $t_{\textrm{rel}}\lesssim t_{\textrm{rel}}^{\textrm{abs}}\ll \textrm{hit}^{Y}_{\alpha }(\theta )$ for all $\theta \in (0,1)$.

Then by [2, Corollary 3.1] we have

$$\begin{aligned} \textrm{hit}^Y_{\beta }(\theta )= (1\pm o(1))\textrm{hit}^Y_{\alpha }(\theta \pm o(1)) \end{aligned}$$

for all $\beta \in (0,1)$. In particular *Y* exhibits $\textrm{hit}_{\beta }$-cutoff for some $\beta \in \left( 0,\frac{1}{2}\right)$. Then by [2, Theorem 3] the chain *Y* also exhibits cutoff. $\square$

#### Lemma 6.11

Let *G* be connected and have degrees bounded by $\Delta$. Then there exists a $\theta \in (0,1)$ (depending on $\Delta$) such that

$$\begin{aligned} t_{\textrm{mix}}^G(1-\theta )\lesssim t_{\textrm{mix}}^{G,\textrm{lazy}}. \end{aligned}$$

#### Proof

Let

$$t_{\textrm{ave}}(\theta ):=\min \left\{ t:\,\max _{x}d_{\textrm{TV}}\left( {\frac{P^t(x,\cdot )+P^{t+1}(x,\cdot )}{2}},{\pi (\cdot )}\right) \le \theta \right\} .$$

Using that by [24, Theorem 1.4] we have $t_{\textrm{ave}}\left( \frac{1}{4}\right) \asymp t_{\textrm{mix}}^{\textrm{lazy}}\left( \frac{1}{4}\right)$, the definition of $t_{\textrm{ave}}\left( \frac{1}{4}\right)$, that $\pi (y)\asymp \pi (z)$ for all *y*, *z* and that the degrees are bounded, we get the result. See [1] for more details. $\square$

### Completing the picture for vertex-transitive graphs with polynomial growth of balls

In this section we will consider vertex-transitive graphs *G*, and denote the volume of a ball of radius *r* in *G* by *V*(*r*).

#### Assumption 6.12

(i) *G* is vertex-transitive.
(ii) There exist constants $C_1,C_2,a_1,a_2>0$ such that $C_1n^{-a_2}\le \varepsilon \le C_2n^{-a_1}$. Let $a:=\frac{\log \left( \frac{1}{\varepsilon }\right) }{\log n}\asymp 1$.
(iii) $\varepsilon \gg \frac{1}{t_{\textrm{mix}}^G(\theta )}$ for all $\theta \in (0,1)$.
(iv) There exist constants $c_1,c_2,c_3,c_4>0$ with the following property. For any vertex *x*, for any positive integers *t* and *r* with $t\le \frac{c_1}{\varepsilon }$, $c_2\sqrt{t}\le r\le c_3\sqrt{t}$, and for any $y\in B_{G}(x,r)$, we have $P^t_{G,\textrm{lazy}}(x,y)\ge \frac{c_4}{V(r)}$.
(v) For any $c'>0$ there exists $c>0$ such that $V\left( \frac{c}{\sqrt{\varepsilon }}\right) \le c'n$.

First we show the following.

#### Proposition 6.13

Assume that *G* and $\varepsilon$ satisfy Assumption 6.12. Then whp the random walk on $G^*$ does not exhibit cutoff, and $t_{\textrm{mix}}^{G^*}\left( \frac{1}{4}\right) \asymp \frac{1}{\varepsilon }$.

After that we show that vertex-transitive graphs with polynomial growth of balls with $\varepsilon \asymp n^{-a}\gg \frac{1}{t_{\textrm{mix}}^G(\theta )}$ satisfy Assumption 6.12, hence the corresponding $G^*$ do not exhibit cutoff.

#### Proposition 6.14

Assume that there exist positive constants $\Delta$, *c* and *d* such that each *G* is a vertex-transitive graph of degree $\Delta$, satisfying $V(r)\le c r^d$ for all *r*. Assume further that $\varepsilon \asymp n^{-a}\gg \frac{1}{t_{\textrm{mix}}^G(\theta )}$ for all $\theta \in (0,1)$ where $a\asymp 1$. Then *G* and $\varepsilon$ satisfy Assumption 6.12.

#### Corollary 6.15

Let *G* and $\varepsilon$ be as in Proposition 6.14. Then whp the random walk on $G^*$ does not exhibit cutoff, and $t_{\textrm{mix}}^{G^*}\left( \frac{1}{4}\right) \asymp \frac{1}{\varepsilon }$.

Finally we show that the simple random walk and lazy random walk on vertex-transitive graphs *G* with polynomial growth of balls do not have cutoff and conclude that for any $\varepsilon \lesssim n^{-\Theta (1)}$ whp $G^*$ does not exhibit cutoff.

#### Proposition 6.16

Let *G* be as in Proposition 6.14 and let $\varepsilon \lesssim n^{-a}$ where $a\asymp 1$. Then whp the random walk on $G^*$ does not exhibit cutoff, and when $\varepsilon \gg \frac{1}{\textrm{diam}\left( G\right) ^2}$, we have $t_{\textrm{mix}}^{G^*}\left( \frac{1}{4}\right) \asymp \frac{1}{\varepsilon }$.

In what follows we will be working under Assumption 6.12, towards the proof of Proposition 6.13.

#### Lemma 6.17

For any constant $C>0$ there exists $\theta \in (0,1)$ such that $t_{\textrm{mix}}^{G^*}(\theta )\ge \frac{C}{\varepsilon }$.

#### Proof

Let $t=\frac{C}{\varepsilon }$. Note that we have

$$\begin{aligned}&1-d_{\textrm{TV}}\left( {P^t_{G^*}(x,\cdot )},{\pi _{G^*}(\cdot )}\right) =\sum _{y}P^t_{G^*}(x,y)\wedge \pi _{G^*}(y)\\&\quad \le \mathbb {P}\!\left( \tau _{\textrm{LR}}>t\right) \sum _{y}\mathbb {P}_{x}\!\Big (X_t=y\;\Big \vert \;\tau _{\textrm{LR}}>t\Big )\wedge \pi _{G^*}(y)+\mathbb {P}\!\left( \tau _{\textrm{LR}}\le t\right) \\&\quad =1-\mathbb {P}\!\left( \tau _{\textrm{LR}}>t\right) \left( 1-\sum _{y}\mathbb {P}_{x}\!\Big (X_t=y\;\Big \vert \;\tau _{\textrm{LR}}>t\Big )\wedge \pi _{G^*}(y)\right) . \end{aligned}$$

Since each vertex in *G* has the same degree, we have $\pi _{G^*}(y)=\frac{1}{n}=\pi _{G}(y)$ and

$$\mathbb {P}_{x}\!\Big (X_t=y\;\Big \vert \;\tau _{\textrm{LR}}>t\Big )= P^t_{G}(x,y)$$, hence

$$\begin{aligned}\sum _{y}\mathbb {P}_{x}\!\Big (X_t=y\;\Big \vert \;\tau _{\textrm{LR}}>t\Big )\wedge \pi _{G^*}(y)&= \sum _{y}P^t_{G}(x,y)\wedge \pi _{G}(y)\\&=1-d_{\textrm{TV}}\left( {P^t_{G}(x,\cdot )},{\pi _{G}(\cdot )}\right) \ll 1, \end{aligned}$$

where for the last bound we used that by Assumption 6.12 we have $t\asymp \frac{1}{\varepsilon }\ll t_{\textrm{mix}}^G(\theta )$ for all $\theta \in (0,1)$.

Since $t\asymp \frac{1}{\varepsilon }$, we also know that $\mathbb {P}\!\left( \tau _{\textrm{LR}}>t\right) \gtrsim 1$. Together these show that

$$\begin{aligned}  &   d_{\textrm{TV}}\left( {P^t_{G^*}(x,\cdot )},{\pi _{G^*}(\cdot )}\right) \, \\  &   \ge \,\mathbb {P}\!\left( \tau _{\textrm{LR}}>t\right) \left( 1-\sum _{y}\mathbb {P}_{x}\!\Big (X_t=y\;\Big \vert \;\tau _{\textrm{LR}}>t\Big )\wedge \pi _{G^*}(y)\right) \,\gtrsim \,1, \end{aligned}$$

hence $t\le t_{\textrm{mix}}^{G^*}(\theta )$ for some $\theta \in (0,1)$. $\square$

#### Definition 6.18

For a vertex *z* of $G^*$ and positive integers $r_{0}$,..., $r_{k}$ we define the $(r_{0},...,r_{k})$-neighbourhood around *z* as follows. Let us consider a ball of radius $r_{0}$ in *G* centred at *z*. This will be level 0. Given a level *i* consisting of balls of radius $r_{i}$ in *G*, we consider the long-range edge of $G^*$ from each vertex of these balls, except for the centres, and attach a copy of the ball of radius $r_{i+1}$ in *G* around the other endpoint. These new balls will form level $(i+1)$. We continue this up to level *k*. Note that the neighbourhood might contain multiple copies of a given vertex of *G*. (In particular copies of the same vertex might appear in multiple levels of the neighbourhood.) Let $\iota$ be the function mapping each vertex of the neighbourhood to the corresponding vertex of *G*. We say that *z* is an $(r_{0},...,r_{k})$-root if the image of the level *k* vertices of the neighbourhood under $\iota$ has size $\asymp \prod _{i=0}^{k}V(r_i)$.^13^

#### Lemma 6.19

For given $(r_{0},...,r_{k})$ and given vertex *z* we have

$$\begin{aligned} \mathbb {P}\!\left( z\text { is an }(r_{0},...,r_{k})\text {-root}\right) = 1-O\left( \frac{1}{n}\prod _{i=0}^{k}V(r_i)\right) . \end{aligned}$$

#### Proof

Let us consider the vertices $v_1$, $v_2$,..., $v_N$ at level *k* of the neighbourhood of *z* (where $N=\prod _{i=0}^{k}(V(r_i)-1)\asymp \prod _{i=0}^{k}V(r_i)$).

For each *i* let $A_{i}$ be the event that $\iota (v_i)$ agrees with one of $\iota (v_1)$,..., $\iota (v_{i-1})$. Note that for each $i\ne j$ we have $\mathbb {P}\!\left( \iota (v_i)=\iota (v_j)\right) \lesssim \frac{1}{n}$, hence for each *i* we have $\mathbb {P}\!\left( A_i\right) \lesssim \frac{i-1}{n}\lesssim \frac{N}{n}$. Then

$$\begin{aligned}&\mathbb {P}\!\left( z\text { is not an }(r_{0},...,r_{k})\text {-root}\right) \\&\quad = \mathbb {P}\!\left( \left| \left\{ \iota (v_1),...,\iota (v_N)\right\} \right|<c'N\right) \\&\quad =\mathbb {P}\!\left( \sum _{i}{{{\mathfrak {1}}}}_{A_{i}^c}<c'N\right) = \mathbb {P}\!\left( \sum _{i}{{{\mathfrak {1}}}}_{A_i}>(1-c')N\right) \\&\quad \le \frac{\mathbb {E}\!\left[ \sum _{i}{{{\mathfrak {1}}}}_{A_i}\right] }{(1-c')N} \lesssim \frac{N\cdot \frac{N}{n}}{N}\asymp \frac{N}{n}. \end{aligned}$$

This finishes the proof.

#### Corollary 6.20

If $\prod _{i=0}^{k}V(r_i)\ll n$ then for any $c\in (0,1)$ with high probability $G^*$ is such that at least *cn* of its vertices are $(r_{0},...,r_{k})$-roots.

#### Proof

Let $$N=\prod _{i=0}^{k}V(r_i)$$. Then

$$\begin{aligned}  &   \mathbb {P}\!\left( \sum _{z}{{{\mathfrak {1}}}}_{\left\{ z\text { an }(r_{0},...,r_{k})\text {-root}\right\} }<cn\right) \le \frac{n\mathbb {P}\!\left( z\text { not an }(r_{0},...,r_{k})\text {-root}\right) }{(1-c)n} \\  &   \quad \lesssim \frac{N}{n}\ll 1. \end{aligned}$$

$$\square $$

#### Remark 6.21

Note that for any $r_0$ and any realisation of $G^*$, any vertex is an $(r_0)$-root.

#### Definition 6.22

Let $c_0>0$ be a sufficiently small constant, to be chosen later. Let *A* be such that $V\left( \frac{c_0}{\sqrt{\varepsilon }}\right) = n^{A}$. Note that $n\ge V\left( \frac{c_0}{\sqrt{\varepsilon }}\right) \gtrsim \varepsilon ^{-\frac{1}{2}}\asymp n^{\frac{a}{2}},$ hence $1\ge A\gtrsim 1$. Let $M\in \mathbb {Z}_{\ge 1}$ and let $B\in [0,A)$ be such that

$$\begin{aligned} AM+B\ge 1+\frac{2\log \log n}{\log n} \qquad \text {and}\qquad (M-1)A+B\le 1. \end{aligned}$$

For sufficiently large values of *n*, such *M* and *B* exist. Since *A* is bounded away from 0, the value of *M* is bounded. Let *r* be such that $V(r)\asymp n^B$. Note that for any *j* we have $V(j)\le V(j+1)\le \Delta V(j)$, hence we can choose *r* to make the multiplicative implicit constants in $V(r)\asymp n^B$ arbitrarily small.

For two given vertices *x* and *y* of $G^*$ let $\Omega _{x,y}$ be the event that *y* is an $(r_{y,0},...,r_{y,M-1})$-root and the $(r_{y,0},...,r_{y,M-1})$-neighbourhood of *y* is disjoint from the $(r_x)$-neighbourhood of *x*, where $r_x=r_{y,1}=...=r_{y,M-1}=\frac{c_0}{\sqrt{\varepsilon }}$ and $r_{y,0}=r$.

#### Lemma 6.23

There exists a constant $c>0$ such that for sufficiently small values of $c_0$ and *r*, for any vertices *x* and *y* having graph distance at least $r_x+r_{y,0}$ in *G*, we have

$$\begin{aligned} \mathbb {P} \!\Big (\mathbb {P}_{x}\!\Big (X_{\tau }=y\;\Big \vert \;G^*\Big )\ge \frac{c}{n}\;\Big \vert \;\Omega _{x,y}\Big )= 1-o\left( \frac{1}{n^2}\right) . \end{aligned}$$

#### Proof

Let us work conditional on the event $\Omega _{x,y}$. Let $\mathcal {X}$ be the set of vertices at level 0 of the $(r_x)$-neighbourhood of *x* and let $\mathcal {Y}$ be the set of vertices at level $M-1$ of the $(r_{y,0},...,r_{y,M-1})$-neighbourhood of *y*. Then we have $|\mathcal {X}|=V(r_x)\asymp n^A$ and $|\mathcal {Y}|\asymp \prod _{i=0}^{M-1}V(r_{y,i})\asymp n^{B+(M-1)A}$ (since *y* is a root).

Let *X* be a lazy random walk on $G^*$ and let us consider a stopping time defined as $\tau =\tau _{\textrm{LR}}^{(M)}+r^2$, where $\tau _{\textrm{LR}}^{(k)}$ is the *k*th time that *X* crosses a long-range edge.

Let *Y* and $\widehat{Y}$ be two processes on the vertices of $G^*$ defined as follows. In the first $r^2$ steps *Y* moves as a lazy simple random walk on *G*, while $\widehat{Y}$ moves as a lazy random walk on $G^*$. Then they cross a long-range edge. Afterwards they both move as a lazy random walk on $G^*$. Let $\tau _{\textrm{LR}}^{(k,Y)}$ be the *k*th time that $(Y_{r^2+1+i})_{i\ge 0}$ crosses a long-range edge and let $\tau _{\textrm{LR}}^{(k,\widehat{Y})}$ be the *k*th time that $(\widehat{Y}_{r^2+1+i})_{i\ge 0}$ crosses a long-range edge.

Note that for any $m\ge 0$ and any path $(z_0,z_1,..z_m)$ we have

$$\begin{aligned}  &   \mathbb {P}_{z_m}\!\left( \left( \widehat{Y}_{i}\right) _{i=0}^{r^2+1+\tau _{\textrm{LR}}^{(k,\widehat{Y})}}=(z_m,z_{m-1},...,z_0)\right) \\  &   \quad =\mathbb {P}_{z_0}\!\left( \left( \eta \left( X_0\right) ,\left( {X}_{i}\right) _{i=0}^{\tau _{\textrm{LR}}^{(k,X)}+r^2}\right) =(z_0,z_1,...,z_m)\right) . \end{aligned}$$

Also note that $r^2\lesssim \frac{1}{\varepsilon }$, hence for any path $y_0,...,y_{r^2}$ in *G* and for any realisation of $G^*$ we have

$$\begin{aligned}\mathbb {P}_{y_0}\!\Big (\widehat{Y}_1=y_1,...,\widehat{Y}_{r^2}=y_{r^2}\;\Big \vert \;G^*\Big )&= (1-O(\varepsilon ))^{r^2}\mathbb {P}_{y_0}\!\Big (Y_1=y_1,...,Y_{r^2}=y_{r^2}\;\Big \vert \;G^*\Big )\\&\asymp \mathbb {P}_{y_0}\!\Big (Y_1=y_1,...,Y_{r^2}=y_{r^2}\;\Big \vert \;G^*\Big ). \end{aligned}$$

Hence for any vertices *x* and *y* we have

$$\begin{aligned}  &   \mathbb {P}_{x}\!\Big (X_{\tau _{\textrm{LR}}^{(k,X)}+r^2}=y\;\Big \vert \;G^*\Big )=\mathbb {P}_{y}\!\Big (\widehat{Y}_{r^2+1+\tau _{\textrm{LR}}^{(k,\widehat{Y})}}=\eta (x)\;\Big \vert \;G^*\Big )\\  &   \quad \asymp \mathbb {P}_{y}\!\Big (Y_{r^2+1+\tau _{\textrm{LR}}^{(k,Y)}}=\eta (x)\;\Big \vert \;G^*\Big ). \end{aligned}$$

Therefore

6.5 $$\begin{aligned}  &   \mathbb {P}_{x}\!\Big (X_{\tau }=y\;\Big \vert \;G^*\Big )\asymp \sum _{z,w}\mathbb {P}_{x}\!\Big (X_{\tau _{\textrm{LR}}^{(1,X)}}=z\;\Big \vert \;G^*\Big )\mathbb {P}_{y}\!\Big (Y_{r^2+1+\tau _{\textrm{LR}}^{(M-1,Y)}}=w\;\Big \vert \;G^*\Big )\nonumber \\  &   \quad {{{\mathfrak {1}}}}_{\eta (z)=w}. \end{aligned}$$

To simplify notation let $\tau ^Y=r^2+1+\tau _{\textrm{LR}}^{(M-1,Y)}$.

We will now bound the terms in the sum in (6.5). For any $z\in \mathcal {X}$ we have

$$\begin{aligned}w^\mathcal {X}_{z}&:=\mathbb {P}_{x}\!\Big (X_{\tau _{\textrm{LR}}-1}=z\;\Big \vert \;G^*\Big )= \sum _{j\ge 0}\mathbb {P}_{x}\!\Big (\tau _{\textrm{LR}}=j+1,X_{j}=z\;\Big \vert \;G^*\Big )\\&\gtrsim \sum _{j=C_0^2/\varepsilon }^{C_1^2/\varepsilon }\varepsilon P^j_{G,\textrm{lazy}}(x,z)\gtrsim \frac{1}{V(r_x)}\asymp n^{-A}. \end{aligned}$$

The first $\gtrsim$ is because $\tau _{\textrm{LR}}\sim \textrm{Geom}_{\ge 1}\!\left( \frac{1}{2}\frac{\varepsilon }{\Delta +\varepsilon }\right)$, so $\mathbb {P}_{x}\!\Big (\tau _{\textrm{LR}}=j+1\;\Big \vert \;G^*\Big )\asymp \varepsilon$ for $j\in \left[ C_0^2/\varepsilon ,C_1^2/\varepsilon \right]$ for any constants $C_0<C_1$, and $\mathbb {P}_{x}\!\Big (X_j=z\;\Big \vert \;G^*,\tau _{\textrm{LR}}=j+1\Big )\asymp P^j_{G,\textrm{lazy}}(x,z)$. The last $\gtrsim$ is true for appropriate choices of $C_0$ and $C_1$ and sufficiently small values of $c_0$ by Assumption 6.12.

Similarly, for all *z* in the $(r_{y,0})$-neighbourhood of *y* we have

$$\begin{aligned} \mathbb {P}_{y}\!\Big (Y_{r^2}=z\;\Big \vert \;G^*\Big )\gtrsim \frac{1}{V(r_{y,0})}\asymp n^{-B}. \end{aligned}$$

Repeatedly using the above results we also get that for any $z\in \mathcal {Y}$ we have

$$\begin{aligned} w^\mathcal {Y}_{z}:=\mathbb {P}_{y}\!\Big (Y_{\tau ^Y}=z\;\Big \vert \;G^*\Big )\gtrsim n^{-B-(M-1)A}. \end{aligned}$$

Let us condition on the $(r_x)$-neighbourhood of *x* and the $(r_{y,0},...,r_{y,M-1})$-neighbourhood of *y* and let $\mathcal {I}$ be the set of yet unpaired long-range half edges. Consider a uniform random matching of $\mathcal {I}$ to complete the graph $G^*$. Let

$$\begin{aligned} w_{i,j}:={\left\{ \begin{array}{ll} w^\mathcal {X}_{i}w^\mathcal {Y}_{j}\wedge n^{-B-MA} &  \text {if }i\in \mathcal {X},j\in \mathcal {Y},\\ 0 &  \text {otherwise}. \end{array}\right. } \end{aligned}$$

Then the right-hand side of (6.5) can be written as $\sum _{i\in \mathcal {I}}w_{i,\eta (i)}$.

By Lemma 1.5 we get that

$$\mathbb {P}\!\left( \sum _{i\in \mathcal {I}}w_{i,\eta (i)}<\frac{1}{2}m\right) \le \exp \left( -\frac{m}{16n^{-B-MA}}\right) $$

where $$m=\frac{1}{|\mathcal {I}|-1}\sum _{i\in \mathcal {I}}\sum _{j\in \mathcal {I}\setminus \{i\}}w_{i,j}$$.

Note that

$$\begin{aligned} n-|\mathcal {I}|\lesssim n^A+n^{B+(M-1)A}\lesssim n. \end{aligned}$$

We used here that $A\le 1$ and $(M-1)A+B\le 1$. We can make the constant in the $\lesssim$ arbitrarily small by choosing the constant $c_0$ in $\frac{c_0}{\sqrt{\varepsilon }}=r_{x}=r_{y,1}=...=r_{y,M-1}$ small and choosing $r=r_{y,0}$ to be small.^14^ Hence $|\mathcal {I}|\asymp n$. This gives

$$\begin{aligned} m\asymp \frac{1}{n}|\mathcal {X}||\mathcal {Y}|n^{-B-MA}\asymp \frac{1}{n}. \end{aligned}$$

Then

$$\begin{aligned} \frac{m}{16n^{-B-MA}}\gtrsim n^{B+MA-1} \gg 15 \log n, \end{aligned}$$

^15^ hence

$$\begin{aligned} \exp \left( -\frac{m}{16n^{-B-MA}}\right) \ll \frac{1}{n^2}. \end{aligned}$$

This finishes the proof. $\square$

#### Lemma 6.24

For any vertex *x* and any realisation of $G^*$ at least a constant proportion of vertices *y* are such that the image under $\iota$ of the $(r_x)$-neighbourhood of *x* is disjoint from the image under $\iota$ of the $(r_{y,0},...,r_{y,M-1})$-neighbourhood of *y*.

#### Proof

We will omit writing that we are considering the images under $\iota$.

The $(r_x)$-neighbourhood of *x* intersects level *j* of the $(r_{y,0},...,r_{y,M-1})$-neighbourhood of *y* if and only if *x* is in the $(r_{y,0},...,r_{y,j-1},r_{y,j}+r_x)$-neighbourhood of *y*. This is equivalent to *y* being in the $(r_{y,j}+r_x,r_{y,j-1},...,r_{y,0})$-neighbourhood of *x*.

For $j\in \{1,2,...,M-1\}$ the $(r_{y,j}+r_x,r_{y,i-j},...,r_{y,0})$-neighbourhood of *x* contains $\asymp n^{jA+B}$ vertices. The $(r_{y,0}+r_x)$-neighbourhood of *x* contains $\asymp n^{A}$ vertices. Taking union over $j=0,1,...,M-1$ we get that there are

$$\begin{aligned} \lesssim n^{A}+\sum _{j=1}^{M-1}n^{jA+B}\lesssim n^A+n^{(M-1)A+B} \end{aligned}$$

vertices *y* with the neighbourhoods intersecting.

This is $\lesssim n$ and we can make the constant in the $\lesssim$ arbitrarily small by choosing $c_0$ and *r* sufficiently small. (We are using the last point of Assumption 6.12 here.) $\square$

#### Lemma 6.25

For any $c\in (0,1)$ there exists a $C>0$ such that for any vertex *x* and any realisation of $G^*$ we have

$$\begin{aligned} \mathbb {P}_{x}\!\Big (\tau >\frac{C}{\varepsilon }\;\Big \vert \;G^*\Big )\le c. \end{aligned}$$

#### Proof

Use that $\tau _{\textrm{LR}}^{M}$ is dominated by the sum of *M* iid $\textrm{Geom}_{\ge 1}\!\left( \frac{1}{2}\frac{\varepsilon }{1+\varepsilon }\right)$ random variables.

#### Lemma 6.26

There exist constants $C>0$ and $\alpha ,\theta \in (0,1)$ such that whp $\textrm{hit}^{G^*}_{\alpha }(\theta )\le \frac{C}{\varepsilon }$.

#### Proof

For each vertex *x* let $V_x=\{y:\,\Omega _{x,y}\text { holds}\}$. Let

$$\begin{aligned} \Omega _0:=\left\{ |V_x|\ge C_1n\quad \forall x;\quad \mathbb {P}_{x}\!\Big (X_{\tau }=y\;\Big \vert \;G^*\Big )\ge \frac{C_2}{n}\quad \forall x,\forall y\in V_x\right\} . \end{aligned}$$

First we show that for sufficiently small values of $C_1$ and $C_2$, the event $\Omega _0$ holds whp, and then we show that on this event we have $\textrm{hit}^{G^*}_{\alpha }(\theta )\le \frac{C}{\varepsilon }$ for some *C*, $\alpha$ and $\theta$.

Let *c* be the constant in Lemma 6.24 and let $\Omega _{\text {root}}$ be the event that at least $\left( 1-\frac{c}{2}\right) n$ vertices *y* are $(r_{y,0},...,r_{y,M-1})$-roots. By Corollary 6.20 this is a high probability event. Also on this event for any given vertex *x*, the event $\Omega _{x,y}$ holds for at least a constant proportion of vertices *y*. This shows that for $C_1$ sufficiently small we have

$$\begin{aligned} \mathbb {P}\!\left( |V_x|\ge C_1n\quad \forall x\right) = 1-o(1). \end{aligned}$$

Also by Lemma 6.23 we have

$$\begin{aligned}&\mathbb {P}\!\left( \exists \, x,y\in V_x:\mathbb {P}_{x}\!\Big (X_{\tau }=y\;\Big \vert \;G^*\Big )<\frac{C_2}{n}\right) \\&\quad \le \sum _{x,y}\mathbb {P}\!\left( \Omega _{x,y},\,\mathbb {P}_{x}\!\Big (X_{\tau }=y\;\Big \vert \;G^*\Big )<\frac{C_2}{n}\right) \\&\quad \le \sum _{\begin{array}{c} x,y:\\ d_G(x,y)\ge r_{x}+r_{y,0} \end{array}}\mathbb {P} \!\Big (\mathbb {P}_{x}\!\Big (X_{\tau }=y\;\Big \vert \;G^*\Big )<\frac{C_2}{n}\;\Big \vert \;\Omega _{x,y}\Big )\le n^2\cdot o\left( \frac{1}{n^2}\right) = o(1). \end{aligned}$$

Together these show that $\mathbb {P}\!\left( \Omega _0\right) =1-o(1)$.

In what follows we work on the event $\Omega _0$. Let *x* be any given vertex of $G^*$.

Note that

$$\begin{aligned} 1-d_{\textrm{TV}}\left( {\mathbb {P}_{x}\!\Big (X_{\tau }=\cdot \;\Big \vert \;G^*\Big )},{\pi _{G^*}(\cdot )}\right) =\sum _{y}\mathbb {P}_{x}\!\Big (X_{\tau }=\cdot \;\Big \vert \;G^*\Big )\wedge \pi _{G^*}(y)\gtrsim 1, \end{aligned}$$

since $\pi _{G^*}(y)\asymp \frac{1}{n}$ for all *y*, $\mathbb {P}_{x}\!\Big (X_{\tau }=\cdot \;\Big \vert \;G^*\Big )\gtrsim \frac{1}{n}$ for all $y\in V_x$ and we have $|V_x|\gtrsim n$. Let $\theta '\in (0,1)$ be a constant such that $d_{\textrm{TV}}\left( {\mathbb {P}_{x}\!\Big (X_{\tau }=\cdot \;\Big \vert \;G^*\Big )},{\pi _{G^*}(\cdot )}\right) \le 1-\theta '$ (for all *x* and all $G^*$ where $\Omega _0$ holds).

Let *A* be any subset of the vertices with $\pi _{G^*}(A)\ge 1-\alpha$ where $\alpha \in \left( 0,\frac{\theta '}{3}\right)$. Let *C* be such that Lemma 6.25 holds with $c=\frac{\theta '}{3}$. Then

$$\begin{aligned}&\mathbb {P}_{x}\!\Big (\tau _A>\frac{C}{\varepsilon }\;\Big \vert \;G^*\Big )\\&\quad \le d_{\textrm{TV}}\left( {\mathbb {P}_{x}\!\Big (X_{\tau }=\cdot \;\Big \vert \;G^*\Big )},{\pi _{G^*}(\cdot )}\right) +\pi _{G^*}(A^c)+\mathbb {P}_{x}\!\Big (\tau >\frac{C}{\varepsilon }\;\Big \vert \;G^*\Big )\\&\quad \le (1-\theta ')+\frac{\theta '}{3}+\frac{\theta '}{3}= 1-\frac{\theta '}{3}. \end{aligned}$$

This shows that on the high probability event $\Omega _0$ we have $\textrm{hit}^{G^*,\textrm{lazy}}_{\alpha }({\theta })\le \frac{{C}}{\varepsilon }$ for $\alpha$ and *C* as above and ${\theta }=1-\frac{\theta '}{3}$.

As in the proof of Lemma 6.9 we get that $\textrm{hit}^{G^*,\textrm{lazy}}_{\alpha }({\theta })=2(1\pm o(1))\textrm{hit}^{G^*}_{\alpha }({\theta }\pm o(1))$. Hence we also have $\textrm{hit}^{G^*}_{\alpha }(\widetilde{\theta })\le \frac{\widetilde{C}}{\varepsilon }$ for some $\alpha$, $\widetilde{C}$ and $\widetilde{\theta }$. $\square$

Now we are ready to conclude that whp $G^*$ does not exhibit cutoff.

#### Proof

Let *C*, $\theta$ and $\alpha$ be as in Lemma 6.26 and work on the high probability event that $\textrm{hit}^{G^*}_{\alpha }(\theta )\le \frac{C}{\varepsilon }$.

By Lemma 4.5 we have that $t_{\textrm{rel}}^{\textrm{abs}}(G^*)\lesssim \frac{1}{\varepsilon }$, and hence using [2, Proposition 3.3 and Corollary 3.4] together with the above bound on $\textrm{hit}^{G^*}_{\alpha }(\theta )$ we get that $\textrm{hit}^{G^*}_{1/2}(\theta ')\lesssim \frac{1}{\varepsilon }$ for any $\theta '\in (0,1)$. Applying [2, Proposition 1.8 and Remark 1.9] we conclude that $t_{\textrm{mix}}^{G^*}(\theta ')\lesssim \frac{1}{\varepsilon }$ for any $\theta '\in (0,1)$.

Let *c* be such that $t_{\textrm{mix}}^{G^*}(1-\theta ')\le \frac{c}{\varepsilon }$. By Lemma 6.17 we have that $t_{\textrm{mix}}^{G^*}(\theta '')\ge \frac{2c}{\varepsilon }$ for some $\theta ''$. Together these show that the walk on $G^*$ does not exhibit cutoff.

If $t_{\textrm{rel}}^{\textrm{abs}}(G^*)\gtrsim \frac{1}{\varepsilon }$ then we immediately get $t_{\textrm{mix}}^{G^*}\left( \frac{1}{4}\right) \gtrsim t_{\textrm{rel}}^{\textrm{abs}}(G^*)\gtrsim \frac{1}{\varepsilon }$. If $t_{\textrm{rel}}^{\textrm{abs}}(G^*)\ll \frac{1}{\varepsilon }$ then using Lemma 6.17, the comparison between the mixing and hitting times from [2, Proposition 1.8 and Remark 1.9], and [2, Proposition 3.3], we again get $t_{\textrm{mix}}^{G^*}\left( \frac{1}{4}\right) \gtrsim \frac{1}{\varepsilon }$. $\square$

Now we proceed to prove Proposition 6.14. The hardest part is establishing the lower bound on the transition probabilities. We prove this via a series of lemmas. First we show the lower bound and a matching upper bound for the diagonal entries $P_{G,\textrm{lazy}}^{t}(o,o)$. Then we upper bound the difference $\left| P_{G,\textrm{lazy}}^{t}(o,o)-P_{G,\textrm{lazy}}^{t}(o,x)\right|$ in terms of the diagonal transition probabilities.

#### Lemma 6.27

In the setup of Proposition 6.14 there exist constants *C* and *L* such that for any $r,k>0$ we have $V(rk)\le Ck^LV(r)$.

#### Proof

This is a consequence of [27, Corollary 1.5]. $\square$

#### Lemma 6.28

In the setup of Proposition 6.14 for any constant $c>0$, any $t>0$ and any vertex *o* we have

$$\begin{aligned} P_{G,\textrm{lazy}}^{t}(o,o)\gtrsim \frac{1}{V(c\sqrt{t})}. \end{aligned}$$

The proof of this is similar to the proof of [7, Lemma 6.12] and is deferred to [1].

#### Lemma 6.29

Let *G* be as in Proposition 6.14. There exists a constant *C* such that for any vertex *o*, any $t\le C\textrm{diam}\left( G\right) ^2$ and any constant $c>0$ we have

$$\begin{aligned} P_{G,\textrm{lazy}}^t(o,o)\lesssim \frac{1}{V(c\sqrt{t})}. \end{aligned}$$

The proof of this relies on a bound of mixing times in terms of spectral profile and is quite technical. Full details are provided in [1].

#### Lemma 6.30

(Gradient inequality) Let *G* be a vertex-transitive graph on *n* vertices. Then for any vertices *o*, *x*, *y* and any $t,s>0$ we have

$$\begin{aligned} \left| P_{G,\textrm{lazy}}^{t+s}(o,x)-P_{G,\textrm{lazy}}^{t+s}(o,y)\right| \lesssim d(x,y)\frac{1}{\sqrt{s}}P_{G,\textrm{lazy}}^{t}(o,o). \end{aligned}$$

#### Proof

The proof is analogous to the proof [7, Proposition 6.1], with the modification that instead of a continuous-time chain we directly consider a lazy chain, and instead of $q(u)=\sqrt{u}e^{-2su}$ we use $q(u)=\sqrt{u}(1-u)^s$. $\square$

#### Proof of Proposition 6.14

Let $P=P_{G,\textrm{lazy}}$. From Lemma 6.30 we get that

$$\begin{aligned} P^{2t}(o,x)\ge P^{2t}(o,o)-C'\frac{d(o,x)}{\sqrt{t}}P^{t}(o,o) \end{aligned}$$

where $C'$ is some positive constant.

From Lemmas 6.28 and 6.29 we know that for any *t* we have $P^{2t}(o,o)\ge \frac{a_1}{V(\sqrt{t})}$, and for any $t\le C\textrm{diam}\left( G\right) ^2$ we have $P^{t}(o,o)\le \frac{a_2}{V(\sqrt{t})}$ where $a_1,a_2>0$ are constants. Let $a_4>0$ be small enough so that $a_1-C'a_2a_4>0$ and let $0<a_3<a_4$.

Then for any *r* with $a_3\sqrt{t}\le r\le a_4\sqrt{t}$ and $x\in B(o,r)$ we have

$$\begin{aligned} P^{2t}(o,x)\ge \left( a_1-C'a_2a_4\right) \frac{1}{V(\sqrt{t})}\asymp \frac{1}{V(\sqrt{t})}\asymp \frac{1}{V(r)}, \end{aligned}$$

where the last $\asymp$ follows from Lemma 6.27. We can get a bound on $P^{2t+1}(o,x)$ with $t\le C\textrm{diam}\left( G\right) ^2$ analogously. To show that the bound holds for any $t\le \frac{c_1}{\varepsilon }$ it remains to check that $\frac{1}{\varepsilon }\lesssim \textrm{diam}\left( G\right) ^2$. For this it is sufficient to prove that $t_{\textrm{mix}}^{G}(\theta )\lesssim \textrm{diam}\left( G\right) ^2$ for some $\theta \in (0,1)$.

Let $t=\textrm{diam}\left( G\right) ^2$. We know that for sufficiently small *c* we have $P^t(o,x)\gtrsim \frac{1}{V(\sqrt{t})}=\frac{1}{n}$ for any $x\in B(o,c\sqrt{t})$. Then

$$\begin{aligned} 1-d_{\textrm{TV}}\left( {P^t(o,\cdot )},{\pi (\cdot )}\right) = \sum _{x}P^t(o,x)\wedge \pi (x) \gtrsim 1, \end{aligned}$$

i.e. $\exists \,\theta '$ with $t_{\textrm{mix}}^{G,\textrm{lazy}}\left( \theta '\right) \le \textrm{diam}\left( G\right) ^2$. Using this, that $t_{\textrm{rel}}^{\textrm{abs}}\lesssim \frac{1}{\varepsilon }$ by Lemma 4.5, that $t_{\textrm{mix}}^G(\theta )\gg \frac{1}{\varepsilon }$ for all $\theta$ by assumption, Lemmas 4.6 and 4.7, and equation (A.1), we get that $t_{\textrm{mix}}^{G}\left( \theta '\right) \le \textrm{diam}\left( G\right) ^2$ for some $\theta '$.

For the last point of Assumption 6.12 it is sufficient to prove that for any $c'$ we have $V(c\textrm{diam}\left( G\right) )\le c'n$ for sufficiently small values of *c*.

Let *x* and *y* be two vertices of *G* with $d(x,y)=\textrm{diam}\left( G\right)$ and let $x_0,...,x_{\textrm{diam}\left( G\right) }$ be a geodesic between *x* and *y*. Then for any $k\in \mathbb {Z}_{\ge 1}$ the balls $B(x_{(3j-1)r},r)$ where $r=\big \lfloor \frac{\textrm{diam}\left( G\right) }{3k}\big \rfloor \asymp \textrm{diam}\left( G\right)$, $j=1,2,...,k$ are disjoint, hence $V(r)\le \frac{n}{k}$.

This finishes the proof. $\square$

Now we proceed to prove Proposition 6.16

#### Lemma 6.31

Let *G* be as in Proposition 6.14. Then the lazy random walk on *G* does not exhibit cutoff.

#### Proof

Let *X* be a lazy random walk on *G*, and let *P* denote its transition matrix.

First we show that $\exists \theta \in (0,1)$ such that we have $t_{\textrm{mix}}^{G,\textrm{lazy}}(\theta )\gtrsim \textrm{diam}\left( G\right) ^2$.

Consider any sequence $t\ll \textrm{diam}\left( G\right) ^2$. Then for a sufficiently small constant *c* we have $P^t(o,x)\gtrsim \frac{1}{V(\sqrt{t})}$ for all $x\in B(o,c\sqrt{t})$. Therefore $\mathbb {P}_{o}\!\left( X_t\in B(o,c\sqrt{t})\right) \gtrsim 1$, while $\pi \left( B(o,c\sqrt{t})\right) =\frac{V(\sqrt{t})}{n}\ll 1$, hence $d_{\textrm{TV}}\left( {P^t(o,\cdot )},{\pi (\cdot )}\right) \gtrsim 1$, where the constant in $\gtrsim 1$ only depends on the choice of *c*.

This shows that there exists $\theta \in (0,1)$ such that for any $t\ll \textrm{diam}\left( G\right) ^2$ for sufficiently large *n* we have $t_{\textrm{mix}}^G(\theta )>t$. This shows $t_{\textrm{mix}}^G(\theta )\gtrsim \textrm{diam}\left( G\right) ^2$.

Now we show that for any $t\asymp \textrm{diam}\left( G\right) ^2$ there exists $\theta \in (0,1)$ with $t_{\textrm{mix}}^{G,\textrm{lazy}}(1-\theta )\le t$.

It is sufficient to consider $t=c\cdot \textrm{diam}\left( G\right) ^2$ where *c* is a sufficiently small constant. Then by Lemma 6.27 we have $V(\sqrt{t})\asymp n$, and we know that $P^t(o,x)\gtrsim \frac{1}{V(\sqrt{t})}$ for all $x\in B(o,c\sqrt{t})$. Therefore

$$1-d_{\textrm{TV}}\left( {P^t(o,\cdot )},{\pi (\cdot )}\right) \ge \sum _{x\in B(o,c\sqrt{t})}P^t(o,x)\wedge \pi (x)\gtrsim n\cdot \frac{1}{n} \asymp 1.$$

This shows that $t_{\textrm{mix}}^{G,\textrm{lazy}}(1-\theta )\le t$ for some $\theta \in (0,1)$. This finishes the proof. $\square$

Lemma 6.31 and Lemma 6.9 immediately gives following.

#### Corollary 6.32

Assume that there exist positive constants $\Delta$, *c* and *d* such that each *G* is a vertex-transitive graph of degree $\Delta$, satisfying $V(r)\le c r^d$ for all *r* (i.e. let *G* be as in Proposition 6.14). Then the simple random walk on *G* does not exhibit cutoff.

#### Lemma 6.33

Let *G* be as in Proposition 6.14. Then $t_{\textrm{mix}}^{G,\textrm{lazy}}\left( \frac{1}{4}\right) \asymp \textrm{diam}\left( G\right) ^2$, $t_{\textrm{mix}}^{G}(\theta )\gtrsim \textrm{diam}\left( G\right) ^2$ for all $\theta \in (0,1)$, and $t_{\textrm{mix}}^{G}(1-\theta )\lesssim \textrm{diam}\left( G\right) ^2$ for some $\theta \in (0,1)$.

The proof of this lemma is deferred to the supplementary material [1].

#### Proof of Proposition 6.16

If $\varepsilon \ll \frac{1}{\textrm{diam}\left( G\right) ^2}$ then by Proposition 6.5, Corollary 6.32 and Lemma 6.33 the random walk on $G^*$ does not exhibit cutoff.

If $\varepsilon \asymp \frac{1}{\textrm{diam}\left( G\right) ^2}$ then by Proposition 6.6, Corollary 6.32 and Lemma 6.33 the random walk on $G^*$ does not exhibit cutoff.

If $\varepsilon \asymp n^{-\Theta (1)}\gg \frac{1}{\textrm{diam}\left( G\right) ^2}$ then by Corollary 6.15 and Lemma 6.33 the random walk on $G^*$ does not exhibit cutoff.

Any sequence $\varepsilon \lesssim n^{-\Theta (1)}$ has a subsequence which falls into one of the above categories. $\square$

### Proof of Theorems 1, 2, 3 and 4

#### Proof of Theorem 1

This follows from Proposition 5.2 (case $\frac{1}{n}\ll \varepsilon \lesssim (\log n)^{-1/3}$), Proposition 6.2 (case $(\log n)^{-1/3}\ll \varepsilon \ll 1$), Proposition 6.4 (case $\varepsilon \asymp 1$), Proposition 6.5 and Proposition 6.6. $\square$

#### Proof of Theorem 2

As above, with Proposition 5.1 instead of Proposition 5.2.

#### Proof of Theorem 3

From Remark 5.4 we know that expanders have linear growth of entropy. It is immediate to see that $t_{\textrm{mix}}^{\textrm{lazy}}\left( \frac{1}{4}\right) \asymp \log n$ and $t_{\textrm{mix}}(\theta )\gtrsim \log n$ for all $\theta \in (0,1)$. Then from Lemma 6.11 we get that $t_{\textrm{mix}}(\theta )\asymp \log n$ for some $\theta \in (0,1)$, so the first three points of Theorem 3 follow from Theorem 1.

The last two points follow from Corollary 6.10$\square$

#### Proof of Theorem 4

This follows from Theorem 2, Proposition 6.16 and Corollary 6.32. $\square$

## General sequences of graphs

In this section we discuss some conjectures and questions regarding more general families of graphs and present a sketch of the proof of Theorem 5.

### Conjectures and open problems

In the two main regimes we considered in the above proofs (graphs with polynomial growth of balls, graphs with linear growth of entropy) we expressed $\mathfrak {h}$, which captures the average entropy of a walk on the quasi-tree, in terms of $\varepsilon$, and proved that cutoff holds in case $\mathfrak {h}\ll \log n$, with mixing time of order $\frac{\log n}{\varepsilon \mathfrak {h}}$. In both cases the order of the entropy of the first long-range edge crossed by the walk did not depend on the starting point. Using this and the assumption on the growth of balls allowed us to prove entropic concentration (2.2). We believe that this phenomenon holds more generally, in the following form.

For a given graph *G*, weight $\varepsilon$ and vertex $\rho$ let

$$\begin{aligned} h(\varepsilon ,\rho ):= H\left( X_{\tau _{\textrm{LR}}}^{(\rho )}\right) \end{aligned}$$

be the entropy of the first long-range edge crossed by a walk on $G^*$, when started from $\rho$.

Also let

$$\begin{aligned} h(\varepsilon ):= \frac{1}{n}\sum _{\rho }h(\varepsilon ,\rho ). \end{aligned}$$

#### Conjecture 7.1

Let $(G_n)$ be a sequence of graphs with (uniformly) bounded degrees and diverging sizes, and let $(\varepsilon _n)$ be a sequence of constants in (0, 1). Let $(G_n^*)$ be as in Definition 1.1. If $h_n(\varepsilon _n,\rho _n)\asymp h_n(\varepsilon _n)$ for all *n* and all $\rho _n$ and $h_n(\varepsilon _n)\ll \log |V_n|$, then the random walk on $(G_n^*)$ exhibits cutoff with high probability, with a mixing time of order $\frac{\log |V_n|}{\varepsilon _n h_n(\varepsilon _n)}$.

Note in particular, that for vertex-transitive graphs the assumption $h(\varepsilon ,\rho )\asymp h(\varepsilon )$ holds for any $\varepsilon$, so Conjecture 7.1 gives a sufficient condition on $\varepsilon$ to ensure cutoff.

We established the result under Assumption 3.1, which in addition to $h(\varepsilon ,\rho )\asymp h(\varepsilon )$ also assumes a matching upper bound on the volume of balls and makes some technical assumptions on $H_2$ and $H_4$. One way we could relax the assumption on the growth of balls is by using better upper bounds on the maximum distance a walk on *G* reaches up to a given time. (This would allow us to choose a smaller radius *R* in the quasi-tree, while still ensuring that the walk will likely not hit the boundary of *R*-balls.)

A-priori it seems plausible that for vertex-transitive graphs the entropy of the walk at a given time *t* is always of the same order as the log of the size of a ball with radius the expected distance travelled by time *t*. The former is always bounded by the latter up to a multiplicative constant, but there is a counterexample for the other direction. For example consider a version of the lamplighter graph where the base graph is a copy of $\mathbb {Z}_k$ and each lamp takes values in $\mathbb {Z}_k$ (and takes steps as a simple random walk on $\mathbb {Z}_k$). We would like to thank Gady Kozma for this example.

The condition $h(\varepsilon ,\rho )\asymp h(\varepsilon )$ is included in Conjecture 7.1 as it should make it easier to control the fluctuations of the entropy, but one might also ask the following question.

#### Open Problem 7.2

Is Conjecture 7.1 true if we replace the condition $h_n(\varepsilon _n,\rho _n)\asymp h_n(\varepsilon _n)\ll \log |V_n|$ with $\max _{\rho _n}h_n(\varepsilon _n,\rho _n)\ll \log |V_n|$?

Since we have $h(\rho ,\varepsilon )\lesssim \frac{1}{\varepsilon }$ for any $\rho$ and any $\varepsilon$ with $\frac{1}{\varepsilon }\le \textrm{diam}\left( G\right)$, a special case of the above would be the following.

#### Conjecture 7.3

Let $(G_n)$ be a sequence of graphs with (uniformly) bounded degrees and diverging sizes, and let $(\varepsilon _n)$ be a sequence of constants in (0, 1) satisfying $\varepsilon _n\gg \frac{1}{\log |V_n|}$. Let $(G_n^*)$ be as in Definition 1.1. Then the random walk on $(G_n^*)$ exhibits cutoff with high probability, with a mixing time of order $\frac{\log |V_n|}{\varepsilon _n h_n(\varepsilon _n)}$.

With a minor modification of the proofs presented above we are able to prove a slightly weaker version of the above statement, namely Theorem 5. We present the required modifications below.

### Sketch proof of Theorem 5

Parts (b) and (c) follow from Propositions 6.5 and 6.6, respectively.

In case $\varepsilon \asymp 1$ the proof is essentially the same as the proof of the $\varepsilon \equiv 1$ case in [18]. In what follows we assume $1\gg \varepsilon \gg \frac{\log \log n}{\log n}$. In this case the proof is similar to the proof of cutoff presented in Sects. 2, 3 and 4, and we explain the required modifications below.

We choose the following values of the parameters.

$$\begin{aligned} R:=C_R\frac{1}{\varepsilon }\log \log n,\qquad K:=C_K\frac{\log \log n}{\log \left( \frac{1}{\varepsilon }\right) },\qquad M:=\frac{1}{2}K, \end{aligned}$$

where $C_R$ and $C_K$ are sufficiently large constants.

We prove concentration of entropy on the event that the graph distance in *T* between two consecutive regeneration edges is at most *R* and then use that with high probability this event will hold up to the level that the walk on the quasi-tree reaches by the mixing time. More precisely, we define

$$\begin{aligned} A_k:=\left\{ d_{T,\text {gr}}\left( X_{\sigma _{k-1}},X_{\sigma _k}\right) \le C_R\frac{1}{\varepsilon }\log \log n\right\} , \end{aligned}$$

where $d_{T,\text {gr}}$ denotes the graph distance on *T*, and

$$\begin{aligned}\mathfrak {h}:=\frac{1}{\mathbb {E}\!\left[ \varphi _2-\varphi _1\right] }\cdot \mathbb {E}\!\left[ \left( -\log \mathbb {P}_{\rho }\!\Big (X'_{\sigma '_1}\in \widetilde{\xi }'\;\Big \vert \;X',T'\Big )\right) {{{\mathfrak {1}}}}_{A_1}\right] .\end{aligned}$$

We prove that for any $b\ge 2$ we have

$$\begin{aligned}  &   \mathbb {E}\!\left[ \left( -\log \mathbb {P}_{\rho }\!\Big (X'_{\sigma '_1}\in \widetilde{\xi }'\;\Big \vert \;X',T'\Big )\right) ^b{{{\mathfrak {1}}}}_{A_1}\right] \lesssim \mathfrak {h}\left( \frac{\log \log n}{\varepsilon }\right) ^{b-1}\\  &   \quad +(\log \log n)^b\ll \mathfrak {h}(\log n)^{b-1}, \end{aligned}$$

and define

$$\begin{aligned} \mathfrak {V}:=\mathfrak {h}\left( \frac{\log \log n}{\varepsilon }\right) +(\log \log n)^2. \end{aligned}$$

Then we can prove that for all $\theta >0$ and $\widehat{C}>0$, there exists a positive constant *C* so that for all $\frac{(K\log b(R))^2}{\mathfrak {V}}\vee 1\le k\le \frac{\widehat{C}\log n}{\mathfrak {h}}=:\ell$ we have

7.1 $$\begin{aligned} \mathbb {P} \!\Big (\left| -\log \mathbb {P} \!\Big (\xi _{\varphi _k}\in \widetilde{\xi }\;\Big \vert \;T,\xi \Big ) -\mathfrak {h}\varphi _k\right| >C\sqrt{\varphi _k\mathfrak {V}},\,\bigcap _{i=1}^{\ell }A_{i}\;\Big \vert \;\mathcal {B}_K(\rho )=T_0\Big ) \le \quad \theta . \end{aligned}$$

For upper bounding the mixing time we let

$$\begin{aligned} t_0:=\frac{\log n}{\nu \mathfrak {h}},\qquad t_w:=\frac{1}{\nu \mathfrak {h}}\sqrt{\frac{\mathfrak {V}\log n}{\mathfrak {h}}},\qquad L:=\frac{1}{2}\nu (t_0+C_Lt_w), \end{aligned}$$

we use the truncation criteria from Definitions 3.16 and 3.19, and we bound the probability of crossing a truncated edge by using (7.1) with $\ell \ge L$, and that $\mathbb {P}\!\left( \left( \bigcap _{i=1}^{\ell }A_{i}\right) ^c\right) \ll 1$.

The lower bound on the mixing time follows analogously, with the above values of the parameters. $\square$

#### Remark 7.4

We worked on event $A_1$ to be able to bound the variance of $-\log \mathbb {P}_{\rho }\!\Big (X'_{\sigma '_1}\in \widetilde{\xi }'\;\Big \vert \;X',T'\Big )$ in terms of the expectation $\mathfrak {h}$. It is easy to check that the value of $\mathfrak {h}$ would be asymptotically the same without the indicator ${{{\mathfrak {1}}}}_{A_1}$.

## Supplementary Information

Below is the link to the electronic supplementary material.

Supplementary file 1 (pdf 540 KB)

## Data Availability

No datasets were generated or analysed during the current study.

## Some auxiliary results

The proofs of the following three lemmas can be found in [1].

### Lemma A.1

Let *G* be a connected graph on *n* vertices with all vertices having degree $\le \Delta$. Let *x* be a vertex of *G* and let *X* be a lazy simple random walk on *G*, starting from *x*. Let *A* be a positive constant. Then there exists a positive constant *c* depending only on $\Delta$ and *A* such that for any vertex *y* and any $t<An^2$ we have

$$\begin{aligned} \mathbb {P}_{x}\!\left( X_t=y\right) \le \frac{c}{\sqrt{t}}. \end{aligned}$$

### Lemma A.2

Let *G* be a connected graph on *n* vertices with all vertices having degree $\le \Delta$. Let *x* be a vertex of *G* and let *X* be a simple random walk on *G*, starting from *x*.Then there exists a positive constant *c* depending only on $\Delta$ such that for any vertex *y* and any time *t* we have

$$\begin{aligned} \mathbb {P}_{x}\!\left( X_t=y\right) \le \frac{c}{\sqrt{t}\wedge n}. \end{aligned}$$

### Lemma A.3

Let $\alpha \in (0,1)$ be given, let $\left( X^{(n)}\right)$ be a sequence of Markov chains satisfying $\textrm{hit}^{X^{(n)}}_{\alpha }(\theta )\gg 1$ for all $\theta \in (0,1)$, and let $Y^{(n)}$ be the lazy version of $X^{(n)}$. Then for any $\theta \in (0,1)$ the hitting times of the chains satisfy

A.1 $$\begin{aligned} \textrm{hit}^{Y^{(n)}}_{\alpha }(\theta )=(1\pm o(1))\cdot 2\textrm{hit}^{X^{(n)}}_{\alpha }(\theta \pm o(1)). \end{aligned}$$

The following properties of $H_b(\cdot )$ and $H_b(\cdot |\cdot )$ (as defined in Definitions 2.23 and 2.24) are easy to check.

### Lemma A.4

Let $b\in \mathbb {Z}_{\ge 1}$ and let $(p_i)$, $(q_i)$ and $(r_i)$ be sequences of real numbers taking values in $[0,\theta ]$ where $\theta \in (0,1)$ is a sufficiently small constant (depending on *b*). Let *W* be a random variable taking values in a countable set $\mathcal {W}$. Then the following properties are satisfied.

(i) If $p_i\ge q_i$ for all *i*, then
  $$\begin{aligned} H_b(p_1,p_2,...)\ge H_b(q_1,q_2,...). \end{aligned}$$
(ii) If $p_i=q_i+r_i$ for all *i* then
  $$\begin{aligned} H_b(p_1,p_2,...)\le H_b(q_1,q_2,...)+H_b(r_1,r_2,...). \end{aligned}$$
(iii) If $p_i=q_ir_i$ for all *i* then
  $$\begin{aligned} H_b(p_1,p_2,...)\lesssim H_b(q_1,q_2,...)+H_b(r_1,r_2,...). \end{aligned}$$
(iv) $$\begin{aligned} H_b(W)\le \left( \log |\mathcal {W}|\right) ^b. \end{aligned}$$
(v) If $p_1+...+p_N=p<1$ and $p_i=0$ for $i>N$ then
  $$\begin{aligned} H_b(p_1,p_2,...)\lesssim p\left( \log N\right) ^b+p(-\log p)^b. \end{aligned}$$

### Lemma A.5

Let $b\in \mathbb {Z}_{\ge 1}$ and let *W* and *Z* be random variables taking values in countable sets. Then

$$\begin{aligned} H_b(W|Z)\le H_b(W)\lesssim H_b(W|Z)+H_b(Z). \end{aligned}$$

## Footnotes

1 The actual definition of $\tau _{2L}$ is more involved and uses the fact that the long-range distance is well-defined on the quasi-tree.

2 Similarly to the configuration model, we think of $G^*$ as adding a long-range half-edge to each vertex of *G* and connecting these according to a uniformly chosen perfect matching.

3 If $a\varepsilon r\le 1$, then LHS=1 and $\varepsilon r\lesssim 1$, so $e^{-c_1'\varepsilon r}\asymp 1$. If $a\varepsilon r>1$, then $\left( \lceil a\varepsilon r\rceil -1\right) \log \left( \frac{1}{\varepsilon }\right) \ge \frac{1}{2}a\varepsilon r\log \left( \frac{1}{\varepsilon }\right) \gtrsim \varepsilon r$.

4 Here we use the following tail bound for binomial random variables. If $0<q<p<1$ then $\mathbb {P}\!\left( \textrm{Bin}\!\left( n,p\right) \le qn\right) \le \exp \left( -n\textrm{D}\left( q||p\right) \right)$ where $\textrm{D}\left( q||p\right) =q\log \left( \frac{q}{p}\right) +(1-q)\log \left( \frac{1-q}{1-p}\right)$. This can be proved using the Chernoff bound.

5 Here we used the following tail bound. If $0<q<p<1$ then $\mathbb {P}\!\left( \textrm{Bin}\!\left( n,p\right) \le qn\right) \le \exp \left( -2n\left( p-q\right) ^2\right)$. This can be proved using Hoeffding’s inequality.

6 counting with multiplicity.

7 We will consider the regime $\varepsilon _n\gg (\log n)^{-\frac{1}{3}}$ in Sect. 6. This means that it would be sufficient to consider Assumption 3.1 with $\beta =\frac{1}{3}$, but the following results hold in case of any $\beta >0$, so we chose to present their proof under this weeker assumption.

8 The improvement to constant *C* compared to [6] is due to the fact that we reveal much fewer vertices for each $z_i$.

9 Here we change the indexing from *y* to $\eta (y)$ and use that $\mathcal {U}(y)=\mathcal {U}(\eta (y))$.

10 We use here that $2K<R$, so for *x*, *y* with $d_G(x,y)\le 2K$ we have $\mathbb {P}\!\left( \mathcal {B}_K^*(x)\cap \mathcal {B}_K^*(y)=\emptyset \right) =0$.

11 Imagine that a lamp is placed at each vertex of *K* and a lamplighter performs a simple random walk on *V*(*K*), at each step switching the lamps at the two corresponding vertices on or off randomly, independently of each other and everything else. The vertices of *G* represent the possible configurations of the system and a random walk on *G* corresponds to the dynamics of the system.

12 *G* has bounded degree, therefore $t_{\textrm{mix}}^X(\theta )\gg 1$ for all $\theta \in (0,1)$ and so $\textrm{hit}_{\alpha }^X(\theta )\gg 1$ for all $\theta \in (0,1)$.

13 More precisely it has size $\ge c'\prod _{i=0}^{k}V(r_i)$ where $c'>0$ is a sufficiently small fixed constant. Note that level *k* contains $\prod _{i=0}^{k}(V(r_i)-1)\asymp \prod _{i=0}^{k}V(r_i)$ vertices; being a root means that the number of different vertices of *G* they correspond to is also of the same order.

14 We used the last point of Assumption 6.12 here.

15 We used here that $B+MA=1+\frac{\omega (1)+\log \log n}{\log n}$.
