# Supplementary material for: Phase transition for random walks on graphs with added weighted random matching
Source: Probab Theory Relat Fields. 2024 Nov 28;193(3-4):989–1074. doi: 10.1007/s00440-024-01342-9 (PMC12680896; doi:10.1007/s00440-024-01342-9)
Supplement: Supplementary file 1 — (pdf 540 KB) [file 440_2024_1342_MOESM1_ESM.pdf]

Supplementary material for the article 'Phase transition for random walks on graphs with added weighted random matching'

Zsuzsanna Baran[1,*], Jonathan Hermon[2], Anđela Šarković[1], and Perla Sousi[1]

[1]University of Cambridge, Cambridge, UK.
[2]University of British Columbia, Vancouver, CA.

[*]Corresponding author, email: zb251@cam.ac.uk
Further emails: jhermon@math.ubc.ca, as2572@cam.ac.uk, p.sousi@statslab.cam.ac.uk

This document contains details of some of the more technical proofs from the above article.

I Concentration of speed and entropy

Proof of Lemma 2.19. We will mimic the proof of [6, Lemma 3.11].

From Corollary 2.16 we know that $\mathbb{E}[\varphi_2 - \varphi_1] \asymp 1$ and $\mathbb{E}[\sigma_2 - \sigma_1] \asymp \frac{1}{\varepsilon}$, hence $\nu \asymp \varepsilon$.

A.s. convergence:

By the ergodic theorem we have $\frac{\varphi_k}{k} \to \mathbb{E}[\varphi_2 - \varphi_1]$ and $\frac{\sigma_k}{k} \to \mathbb{E}[\sigma_2 - \sigma_1]$ almost surely. These give

$$\frac{d_T(\rho, X_{\sigma_k})}{\sigma_k} = \frac{\varphi_k}{\sigma_k} \to \frac{\mathbb{E}[\varphi_2 - \varphi_1]}{\mathbb{E}[\sigma_2 - \sigma_1]} = \nu \quad \text{a.s..}$$

Let $M_k = \max\{i : \sigma_i \le k\}$ and let $b_j = \sup_{i: i \le \sigma_{j+1}} d_T(\rho, X_i) - \varphi_{j+1}$. The variables $(b_j)_{j \ge 1}$ are identically distributed and have finite mean and variance, hence $\frac{b_j}{j} \to 0$ a.s. as $j \to \infty$.

Then

$$\frac{\varphi_{M_k}}{\sigma_{M_k}} \frac{\sigma_{M_k}}{k} \quad \le \quad \frac{d_T(\rho, X_k)}{k} \quad \le \quad \frac{\varphi_{M_k+1} + b_{M_k}}{\sigma_{M_k+1}} \frac{\sigma_{M_k+1}}{k}$$

and both sides converge to ν almost surely. [1]

Bound on the deviations for X_{σ_k}:

[1] $\frac{\varphi_{M_k}}{\sigma_{M_k}} \to \nu$ a.s. by above. $\frac{b_{M_k}}{\sigma_{M_k+1}} \le \frac{b_{M_k}}{M_k} \to 0$ a.s. . $\frac{\sigma_{M_k}}{k} \le 1 \le \frac{\sigma_{M_k+1}}{k}$ and $\frac{\sigma_{M_k+1} - \sigma_{M_k}}{k} \le \frac{\sigma_{M_k+1} - \sigma_{M_k}}{\sigma_{M_k}} = \frac{\sigma_{M_k+1} - \sigma_{M_k}}{M_k} \frac{M_k}{\sigma_{M_k}} \to 0$ a.s., hence $\frac{\sigma_{M_k}}{k}, \frac{\sigma_{M_k+1}}{k} \to 1$ a.s. .

For any $\alpha > 0$ we have

$$\begin{aligned}
&\mathbb{P}\big(|d_T(\rho, X_{\sigma_k}) - \nu\sigma_k| > C'\sqrt{\varepsilon\sigma_k}\big) \qquad (1)\\
&\quad \le \quad \mathbb{P}\bigg(|\varphi_k - k\mathbb{E}[\varphi_2 - \varphi_1]| > \frac{1}{2}C'\sqrt{\alpha k}\bigg)\\
&\qquad + \quad \mathbb{P}\bigg(|\nu\sigma_k - \nu k\mathbb{E}[\sigma_2 - \sigma_1]| > \frac{1}{2}C'\sqrt{\alpha k}\bigg) \quad + \quad \mathbb{P}\bigg(\sigma_k \le \alpha\frac{k}{\varepsilon}\bigg).
\end{aligned}$$

We know that $\mathbb{P}\Big(|\varphi_k - \varphi_1 - (k-1)\mathbb{E}[\varphi_2 - \varphi_1]| > C''\sqrt{k\mathrm{Var}\,(\varphi_2 - \varphi_1)}\Big) \to 0$ as $C'' \to \infty$, that $\mathbb{P}(\varphi_1 \ge C'') \to 0$ as $C'' \to \infty$ and that $\mathrm{Var}(\varphi_2 - \varphi_1) \lesssim 1$ and $\mathbb{E}[\varphi_2 - \varphi_1] \asymp 1$ [2]. This allows us to control the first term in (1).

We know that $\mathbb{P}\Big(|\nu(\sigma_k - \sigma_1) - \nu(k-1)\mathbb{E}[\sigma_2 - \sigma_1]| > C''\nu\sqrt{k\mathrm{Var}(\sigma_2 - \sigma_1)}\Big) \to 0$ as $C'' \to \infty$, that $\mathbb{P}(\nu\sigma_1 > C'') \to 0$ as $C'' \to \infty$ and that $\mathbb{E}[\sigma_1] \lesssim \frac{1}{\varepsilon}$, $\mathrm{Var}(\sigma_2 - \sigma_1) \lesssim \frac{1}{\varepsilon^2}$ and $\nu \asymp \varepsilon$. [3] This lets us control the second term in (1).

The last term in (1) goes $\to 0$ as $\alpha \to 0$.

Hence for any $\theta > 0$ there exists C' such that for all k we have

$$\mathbb{P}\big(|d_T(\rho, X_{\sigma_k}) - \nu\sigma_k| > C'\sqrt{\varepsilon\sigma_k}\big) \le \theta.$$

Bound on the deviations for the whole sequence:

Fix any $k \ge \frac{J}{\varepsilon}$ and let $s^- = \left(\frac{\sqrt{\ell^2 + \frac{4k}{\mathbb{E}[\sigma_2 - \sigma_1]}} - \ell}{2}\right)^2$ and $s^+ = \left(\frac{\sqrt{\ell^2 + \frac{4k}{\mathbb{E}[\sigma_2 - \sigma_1]}} + \ell}{2}\right)^2$ where ℓ is a large integer to be chosen later.

Note that

$$\begin{aligned}
\mathbb{P}\Big(|d_T(\rho, X_k) - \nu k| \ge C\sqrt{\varepsilon k}\Big) \quad &\le \quad \mathbb{P}(k \notin [\sigma_{s^-}, \sigma_{s^+}])\\
&\quad + \quad \mathbb{P}\Big(d_T(\rho, X_k) \ge \nu k + C\sqrt{\varepsilon k},\ k \in [\sigma_{s^-}, \sigma_{s^+}]\Big)\\
&\quad + \quad \mathbb{P}\Big(d_T(\rho, X_k) \le \nu k - C\sqrt{\varepsilon k},\ k \in [\sigma_{s^-}, \sigma_{s^+}]\Big).
\end{aligned}$$

We will show that the three terms on the right $\to 0$ as $\ell \to \infty$ and $C \to \infty$ (in terms of ℓ).

For any s we have

$$\begin{aligned}
&\mathbb{P}\big(|\sigma_s - s\mathbb{E}[\sigma_2 - \sigma_1]| \ge \ell\sqrt{s}\mathbb{E}[\sigma_2 - \sigma_1]\big)\\
&\le \mathbb{P}\bigg(|(\sigma_s - \sigma_1) - (s-1)\mathbb{E}[\sigma_2 - \sigma_1]| \ge \frac{1}{2}\ell\sqrt{s}\mathbb{E}[\sigma_2 - \sigma_1]\bigg) + \mathbb{P}\bigg(|\sigma_1 - \mathbb{E}[\sigma_2 - \sigma_1]| \ge \frac{1}{2}\ell\sqrt{s}\mathbb{E}[\sigma_2 - \sigma_1]\bigg).
\end{aligned}$$

We know that $(\sigma_s - \sigma_1)$ is the sum of $(s-1)$ iid random variables that have mean $\mathbb{E}[\sigma_2 - \sigma_1] \asymp \frac{1}{\varepsilon}$ and variance $\lesssim \frac{1}{\varepsilon^2} \asymp \mathbb{E}[\sigma_2 - \sigma_1]^2$. We also know that σ_1 has mean $\asymp \frac{1}{\varepsilon} \asymp \mathbb{E}[\sigma_2 - \sigma_1]$ and variance $\lesssim \frac{1}{\varepsilon^2} \lesssim \mathbb{E}[\sigma_2 - \sigma_1]^2$. Hence both probabilities on the RHS $\to 0$ as $\ell \to \infty$, uniformly in s.

Note that

$$s^-\mathbb{E}[\sigma_2 - \sigma_1] + \ell\sqrt{s^-}\mathbb{E}[\sigma_2 - \sigma_1] \quad = \quad s^+\mathbb{E}[\sigma_2 - \sigma_1] - \ell\sqrt{s^+}\mathbb{E}[\sigma_2 - \sigma_1] \quad = \quad k,$$

[2] By Corollary 2.16.
[3] By Corollary 2.16.

hence we get that $\mathbb{P}(k \notin [\sigma_{s^-}, \sigma_{s^+}]) \to 0$ as $\ell \to \infty$.

We know that $\varepsilon k \geq J$ and that $\frac{4k}{\mathbb{E}[\sigma_2 - \sigma_1]} \asymp \varepsilon k \gtrsim 1$, hence for any fixed ℓ we have $s^+ - s^- = \ell\sqrt{\ell + \frac{4k}{\mathbb{E}[\sigma_2-\sigma_1]}} \asymp \sqrt{\varepsilon k}$ and $\sqrt{s^-} \asymp \sqrt{\ell^2 + \frac{4k}{\mathbb{E}[\sigma_2-\sigma_1]}} - \ell \asymp \sqrt{\varepsilon k}$.

Note that

$$\begin{aligned}
&\mathbb{P}\Big(d_T(\rho, X_k) \geq \nu k + C\sqrt{\varepsilon k},\ k \in [\sigma_{s^-}, \sigma_{s^+}]\Big) \\
&\leq \quad \mathbb{P}\Big(\varphi_{s^-} \geq \nu\sigma_{s^-} + \frac{1}{3}C\sqrt{\varepsilon\sigma_{s^-}}\Big) \quad + \quad \mathbb{P}\Big(\varphi_{s^+} - \varphi_{s^-} \geq \frac{1}{3}C\sqrt{\varepsilon k}\Big) \\
&\qquad + \quad \mathbb{P}\Bigg(\sup_{i \in [\sigma_{s^-}, \sigma_{s^+}]} d_T(\rho, X_i) \geq \varphi_{s^-} + \frac{2}{3}C\sqrt{\varepsilon k};\ \varphi_{s^+} \leq \varphi_{s^-} + \frac{1}{3}C\sqrt{\varepsilon k}\Bigg).
\end{aligned}$$

By the statement for the subsequence (X_{σ_i}) we know that $\mathbb{P}\big(\varphi_{s^-} \geq \nu\sigma_{s^-} + \frac{1}{3}C\sqrt{\varepsilon\sigma_{s^-}}\big) \to 0$ as $C \to \infty$.

We know that $\varphi_{s^+} - \varphi_{s^-}$ has mean $(s^+ - s^-)\mathbb{E}[\varphi_2 - \varphi_1] \asymp (s^+ - s^-) \asymp \sqrt{\varepsilon k}$ and variance $(s^+ - s^-)\mathrm{Var}\,(\varphi_2 - \varphi_1) \lesssim (s^+ - s^-) \asymp \sqrt{\varepsilon k}$, hence $\mathbb{P}\Big(\varphi_{s^+} - \varphi_{s^-} \geq \frac{1}{3}C\sqrt{\varepsilon k}\Big) \to 0$ as $C \to \infty$.

By noting that the walk has to backtrack at least $\frac{1}{3}C\sqrt{\varepsilon k}$ levels after first hitting level $\varphi_{s^-} + \frac{2}{3}C\sqrt{\varepsilon k}$ we get that

$$\mathbb{P}\Bigg(\sup_{i \in [\sigma_{s^-}, \sigma_{s^+}]} d_T(\rho, X_i) \geq \varphi_{s^-} + \frac{2}{3}C\sqrt{\varepsilon k};\ \varphi_{s^+} \leq \varphi_{s^-} + \frac{1}{3}C\sqrt{\varepsilon k}\Bigg) \quad \lesssim \quad \delta^{\frac{1}{3}C\sqrt{\varepsilon k}} \quad \lesssim \quad \Big(\delta^{\frac{1}{3}\sqrt{\varepsilon}}\Big)^C.$$

This $\to 0$ as $C \to \infty$.

Note that

$$\begin{aligned}
&\mathbb{P}\Big(d_T(\rho, X_k) \leq \nu k - C\sqrt{\varepsilon k},\ k \in [\sigma_{s^-}, \sigma_{s^+}]\Big) \quad \leq \quad \mathbb{P}(\varphi_{s^-} \leq \nu\sigma_{s^+} - C\sqrt{\varepsilon\sigma_{s^-}}) \\
&\leq \quad \mathbb{P}\Big(\varphi_{s^-} \leq \nu\sigma_{s^-} - \frac{1}{2}C\sqrt{\varepsilon\sigma_{s^-}}\Big) \quad + \quad \mathbb{P}\Big(\nu\sigma_{s^-} - \frac{1}{2}C\sqrt{\varepsilon\sigma_{s^-}} < \nu\sigma_{s^+} - C\sqrt{\varepsilon\sigma_{s^-}}\Big).
\end{aligned}$$

By the result for the subsequence X_{σ_i} we know that $\mathbb{P}\big(\varphi_{s^-} \leq \nu\sigma_{s^-} - \frac{1}{2}C\sqrt{\varepsilon\sigma_{s^-}}\big) \to 0$ as $C \to \infty$.

Since $\nu\,(\sigma_{s^+} - \sigma_{s^-})$ has mean and variance $\asymp s^+ - s^- \gtrsim 1$, therefore $\mathbb{P}(\nu\,(\sigma_{s^+} - \sigma_{s^-}) \geq u(s^+ - s^-)) \to 0$ as $u \to \infty$.

Since $\varepsilon\sigma_{s^-}$ has mean and variance $\asymp s^- \gtrsim 1$, therefore $\mathbb{P}\Big(\sqrt{\varepsilon\sigma_{s^-}} \leq \beta\sqrt{s^-}\Big) \to 0$ as $\beta \to 0$.

Also, $s^+ - s^- \asymp \sqrt{s^-}$. Together these give that $\mathbb{P}\big(\nu\,(\sigma_{s^+} - \sigma_{s^-}) > \frac{1}{2}C\sqrt{\varepsilon\sigma_{s^-}}\big) \to 0$ as $C \to \infty$.

Bound on the sup*:*

Note that

$$\begin{aligned}
&\mathbb{P}\Big(\sup_{s:\, s \leq t} d_T(\rho, X_s) > \nu t + 2C\sqrt{\varepsilon t}\Big) \\
&\leq \mathbb{P}\Big(d_T(\rho, X_t) > \nu t + C\sqrt{\varepsilon t}\Big) + \sum_{s<t} \mathbb{P}\Big(d_T(\rho, X_s) > \nu t + 2C\sqrt{\varepsilon t},\ d_T(\rho, X_t) < \nu t + C\sqrt{\varepsilon t}\Big).
\end{aligned}$$

We already know that the first term $\to 0$ as $C \to \infty$. In the sum each term is $\leq \delta^{C\sqrt{\varepsilon t}}$, so the whole sum is $\leq t\delta^{C\sqrt{\varepsilon t}}$. This $\to 0$ as $C \to \infty$. □

Proof of Prop 2.20. This is similar to the proof of [6, Proposition 3.15].

A.s. convergence:

Let $Y_i = -\log \frac{\mathbb{P}_\rho\left(\xi_{\varphi_i} \in \widetilde{\xi} \mid \xi, T\right)}{\mathbb{P}_\rho\left(\xi_{\varphi_{i-1}} \in \widetilde{\xi} \mid \xi, T\right)}$ for $i \geq 1$ and let $Y_0 = -\log \mathbb{P}_\rho\left(\xi_{\varphi_0} \in \widetilde{\xi} \,\middle|\, \xi, T\right)$. We know that variables $(Y_i)_{i \geq 2}$ are distributed as $-\log \mathbb{P}_\rho\left(X'_{\sigma'_1} \in \xi' \,\middle|\, T', X'\right)$ (whether or not we are conditioning on T_0). Hence by the ergodic theorem

$$\frac{-\log \mathbb{P}_\rho\left(\xi_{\varphi_k} \in \widetilde{\xi} \,\middle|\, T, \xi\right)}{k} = \frac{\sum_{i=0}^k Y_i}{k} \to \mathbb{E}\left[-\log \mathbb{P}_\rho\left(X'_{\sigma'_1} \in \xi' \,\middle|\, T', X'\right)\right] =: \widetilde{h} \quad \text{a.s.} \,.$$

We know that $\frac{\varphi_k}{k} \to \mathbb{E}[\varphi_2 - \varphi_1]$ a.s., hence we get the a.s. convergence to $\mathfrak{h}$ along the subsequence (ξ_{φ_k}). Using that $\{\xi_k \in \widetilde{\xi}\}$ is non-increasing in k, we also get a.s. convergence to $\mathfrak{h}$ for the full sequence (ξ_k).

Bound on the deviations for ξ_{φ_k}:

By Lemma 2.21 and by the assumption $k \geq \frac{(K \log b(R))^2}{\mathfrak{V}}$ we have

$$\operatorname{Var}\left(-\log \mathbb{P}_\rho\left(\xi_{\varphi_k} \in \widetilde{\xi} \,\middle|\, \xi, T\right) \,\middle|\, T_0\right) \quad \lesssim \quad k\mathfrak{V} + (K \log b(R))^2 \quad \lesssim \quad k\mathfrak{V}.$$

Applying Chebyshev we get

$$\begin{aligned} &\mathbb{P}\left(\left|-\log \mathbb{P}\left(\xi_{\varphi_k} \in \widetilde{\xi} \,\middle|\, T, \xi\right) - \mathfrak{h}\varphi_k\right| > C\sqrt{k\mathfrak{V}} \,\middle|\, T_0\right) \\ \leq \quad & \frac{2\operatorname{Var}\left(-\log \mathbb{P}\left(\xi_{\varphi_k} \in \widetilde{\xi} \,\middle|\, T, \xi\right) - \mathfrak{h}\varphi_k \,\middle|\, T_0\right)}{\left(C\sqrt{k\mathfrak{V}} - \left|\mathbb{E}\left[-\log \mathbb{P}\left(\xi_{\varphi_k} \in \widetilde{\xi} \,\middle|\, T, \xi\right) \,\middle|\, T_0\right] - \mathbb{E}[\mathfrak{h}\varphi_k \mid T_0]\right|\right)^2} \end{aligned}$$

We know from above that $\operatorname{Var}\left(-\log \mathbb{P}\left(\xi_{\varphi_k} \in \widetilde{\xi} \,\middle|\, T, \xi\right) \,\middle|\, T_0\right) \lesssim k\mathfrak{V}$.
Also $\operatorname{Var}(\mathfrak{h}\varphi_k \mid T_0) = \operatorname{Var}(\varphi_k \mid T_0)\, \mathfrak{h}^2 \lesssim k\mathfrak{V}$. [4] Together these give

$$\operatorname{Var}\left(-\log \mathbb{P}\left(\xi_{\varphi_k} \in \widetilde{\xi} \,\middle|\, T, \xi\right) - \mathfrak{h}\varphi_k \,\middle|\, T_0\right) \quad \lesssim \quad k\mathfrak{V}.$$

To bound the denominator note that

$$\begin{aligned} \mathbb{E}\left[-\log \mathbb{P}\left(\xi_{\varphi_k} \in \widetilde{\xi} \,\middle|\, T, \xi\right) \,\middle|\, T_0\right] \quad &= \quad (k-1)\mathfrak{h}\mathbb{E}[\varphi_2 - \varphi_1] + \mathbb{E}[Y_0 + Y_1 \mid T_0] \quad \text{and} \\ \mathbb{E}[\mathfrak{h}\varphi_k \mid T_0] \quad &= \quad \mathfrak{h}\left((k-1)\mathbb{E}[\varphi_2 - \varphi_1] + \mathbb{E}[\varphi_1 \mid T_0]\right). \end{aligned}$$

The same way as we proved the bound on $\mathbb{E}\left[(Y_0 + Y_1)^2 \,\middle|\, T_0\right]$ for Lemma 2.21 we get

$$\mathbb{E}[Y_0 + Y_1 \mid T_0] \quad \lesssim \quad K \log b(R) \quad \lesssim \quad \sqrt{k\mathfrak{V}}.$$

By Corollary 2.16 we know that

$$\mathbb{E}[\varphi_1 \mid T_0] \lesssim K + 1 \lesssim \sqrt{k\mathfrak{V}},$$

[4] By Corollary 2.16 we know that $\operatorname{Var}(\varphi_i - \varphi_{i-1} \mid T_0) \leq \mathbb{E}\left[(\varphi_i - \varphi_{i-1})^2 \,\middle|\, T_0\right] \lesssim 1$ for $i = 2, ..., k$ and that $\operatorname{Var}(\varphi_1 \mid T_0) = \operatorname{Var}(\varphi_1 - \varphi_0 \mid T_0) \leq \mathbb{E}\left[(\varphi_1 - \varphi_0)^2 \,\middle|\, T_0\right] \lesssim 1$. By Lemma 2.12 we know that $(\varphi_i - \varphi_{i-1})_{i=2}^k$ and φ_1 are all independent, hence $\operatorname{Var}(\varphi_k \mid T_0) = \operatorname{Var}(\varphi_1 \mid T_0) + \sum_{i=2}^k \operatorname{Var}(\varphi_i - \varphi_{i-1} \mid T_0) \lesssim k$. By definition $\mathfrak{h}^2 \lesssim \mathfrak{V}$.

hence for any sufficiently large C we have

$$\left(C\sqrt{k\mathfrak{V}} - \left|\mathbb{E}\left[-\log\mathbb{P}\left(\xi_{\varphi_k}\in\widetilde{\xi}\;\middle|\;T,\xi\right)\;\middle|\;T_0\right] - \mathbb{E}[\mathfrak{h}\varphi_k \mid T_0]\right|\right)^2 \quad\geq\quad \frac{C^2}{2}k\mathfrak{V}.$$

This then gives

$$\mathbb{P}\left(\left|-\log\mathbb{P}\left(\xi_{\varphi_k}\in\widetilde{\xi}\;\middle|\;T,\xi\right) - \mathfrak{h}\varphi_k\right| > C\sqrt{k\mathfrak{V}}\;\middle|\;T_0\right) \quad\lesssim\quad \frac{2}{C^2}.$$

This $\to 0$ as $C\to\infty$. We established the deviation bound for the sequence $(\xi_{\varphi_k})_{k\geq 1}$.

Bound on the deviations for the whole sequence:

Fix any k and let $s^- = \left(\frac{\sqrt{\ell^2+\frac{4(k-K)}{\mathbb{E}[\varphi_2-\varphi_1]}}-\ell}{2}\right)^2$ and $s^+ = \left(\frac{\sqrt{\ell^2+\frac{4(k-K)}{\mathbb{E}[\varphi_2-\varphi_1]}}+\ell}{2}\right)^2$ where ℓ is a large integer to be chosen later. Then we have

$$\begin{aligned}
&\mathbb{P}\left(\left|-\log\mathbb{P}\left(\xi_k\in\widetilde{\xi}\;\middle|\;T,\xi\right) - \mathfrak{h}k\right| > C\sqrt{k\mathfrak{V}}\;\middle|\;T_0\right)\\
\leq\quad &\mathbb{P}\left(-\log\mathbb{P}\left(\xi_k\in\widetilde{\xi}\;\middle|\;T,\xi\right) > \mathfrak{h}k + C\sqrt{k\mathfrak{V}};\; k\in[\varphi_{s^-},\varphi_{s^+}]\;\middle|\;T_0\right)\\
+\quad &\mathbb{P}\left(-\log\mathbb{P}\left(\xi_k\in\widetilde{\xi}\;\middle|\;T,\xi\right) < \mathfrak{h}k - C\sqrt{k\mathfrak{V}};\; k\in[\varphi_{s^-},\varphi_{s^+}]\;\middle|\;T_0\right)\\
+\quad &\mathbb{P}(k\notin[\varphi_{s^-},\varphi_{s^+}] \mid T_0).
\end{aligned}$$

We wish to show that each term on the RHS $\to 0$ as $\ell\to\infty$ and $C\to\infty$ (in terms of ℓ).

For any $s\geq 2$ we have

$$\begin{aligned}
&\mathbb{P}\big(|\varphi_s - K - s\mathbb{E}[\varphi_2-\varphi_1]| \geq \ell\sqrt{s}\mathbb{E}[\varphi_2-\varphi_1]\big)\\
\leq\quad &\mathbb{P}\left(|(\varphi_s-\varphi_1) - (s-1)\mathbb{E}[\varphi_2-\varphi_1]| \geq \frac{1}{2}\ell\sqrt{s}\mathbb{E}[\varphi_2-\varphi_1]\right)\\
+\quad &\mathbb{P}\left(|\varphi_1 - K - \mathbb{E}[\varphi_2-\varphi_1]| \geq \frac{1}{2}\ell\sqrt{s}\mathbb{E}[\varphi_2-\varphi_1]\right).
\end{aligned}$$

We know that $(\varphi_s-\varphi_1)$ has mean $(s-1)\mathbb{E}[\varphi_2-\varphi_1]$ and variance $\lesssim (s-1)\asymp(s-1)\mathbb{E}[\varphi_2-\varphi_1]^2$, hence $\mathbb{P}\big(|(\varphi_s-\varphi_1)-(s-1)\mathbb{E}[\varphi_2-\varphi_1]| \geq \frac{1}{2}\ell\sqrt{s}\mathbb{E}[\varphi_2-\varphi_1]\big)\to 0$ as $\ell\to\infty$ (uniformly).

We also know that $\varphi_1 - K = \varphi_1-\varphi_0$ has mean $\asymp 1\asymp\mathbb{E}[\varphi_2-\varphi_1]$ and variance $\lesssim 1$, hence $\mathbb{P}\big(|\varphi_1 - K - \mathbb{E}[\varphi_2-\varphi_1]| \geq \frac{1}{2}\ell\sqrt{s}\mathbb{E}[\varphi_2-\varphi_1]\big)\to 0$ as $\ell\to\infty$ (uniformly).

$$s^-\mathbb{E}[\varphi_2-\varphi_1] + \ell\sqrt{s^-}\mathbb{E}[\varphi_2-\varphi_1] \quad=\quad s^+\mathbb{E}[\varphi_2-\varphi_1] - \ell\sqrt{s^+}\mathbb{E}[\varphi_2-\varphi_1] \quad=\quad k-K,$$

hence we get that $\mathbb{P}(k\notin[\varphi_{s^-},\varphi_{s^+}])\to 0$ as $\ell\to\infty$.

By the monotonicity of events $\{\xi_i\in\widetilde{\xi}\}$ we get that

$$\begin{aligned}
&\mathbb{P}\left(-\log\mathbb{P}\left(\xi_k\in\widetilde{\xi}\;\middle|\;T,\xi\right) > \mathfrak{h}k + C\sqrt{k\mathfrak{V}};\; k\in[\varphi_{s^-},\varphi_{s^+}]\;\middle|\;T_0\right)\\
\leq\quad &\mathbb{P}\left(-\log\mathbb{P}\left(\xi_{\varphi_{s^+}}\in\widetilde{\xi}\;\middle|\;T,\xi\right) > \mathfrak{h}\varphi_{s^-} + C\sqrt{\varphi_{s^-}\mathfrak{V}}\;\middle|\;T_0\right) \quad\text{and}\\
&\mathbb{P}\left(-\log\mathbb{P}\left(\xi_k\in\widetilde{\xi}\;\middle|\;T,\xi\right) < \mathfrak{h}k - C\sqrt{k\mathfrak{V}};\; k\in[\varphi_{s^-},\varphi_{s^+}]\;\middle|\;T_0\right)\\
\leq\quad &\mathbb{P}\left(-\log\mathbb{P}\left(\xi_{\varphi_{s^-}}\in\widetilde{\xi}\;\middle|\;T,\xi\right) < \mathfrak{h}\varphi_{s^+} - C\sqrt{\varphi_{s^-}\mathfrak{V}}\;\middle|\;T_0\right).
\end{aligned}$$

For any $\alpha \in (0,1)$ we have

$$\begin{aligned}
&\mathbb{P}\Big(-\log\mathbb{P}\Big(\xi_{\varphi_{s^+}} \in \widetilde{\xi} \,\Big|\, T,\xi\Big) > \mathfrak{h}\varphi_{s^-} + C\sqrt{\varphi_{s^-}\mathfrak{V}} \,\Big|\, T_0\Big) \\
\leq\ & \mathbb{P}\Big(-\log\mathbb{P}\Big(\xi_{\varphi_{s^+}} \in \widetilde{\xi} \,\Big|\, T,\xi\Big) > \mathfrak{h}\varphi_{s^+} + \alpha C\sqrt{\varphi_{s^+}\mathfrak{V}} \,\Big|\, T_0\Big) \\
+\ & \mathbb{P}\Big(\mathfrak{h}\left(\varphi_{s^+} - \varphi_{s^-}\right) \geq C\left(\sqrt{\mathfrak{V}\varphi_{s^-}} - \alpha\sqrt{\mathfrak{V}\varphi_{s^+}}\right) \,\Big|\, T_0\Big).
\end{aligned}$$

We already know that the first term on RHS $\to 0$ as $\alpha C \to \infty$.

We also know that $\mathfrak{h} \asymp \sqrt{\mathfrak{V}}$, that $\mathbb{P}(\varphi_{s^+} - \varphi_{s^-} \geq u\,(s^+ - s^-)) \to 0$ as $u \to \infty$, and that $\mathbb{P}\big(\varphi_{s^-} \leq \frac{1}{2}s^-\big) = 0$ and $\mathbb{P}\big(\varphi_{s^+} \geq \frac{1}{2\alpha^2}s^+\big) \to 0$ as $\frac{1}{2\alpha^2} \to \infty$ [5]. Putting these together and using that for any fixed ℓ we have $s^+ - s^- \asymp \sqrt{s^+} \asymp \sqrt{s^-}$ we get that the second term on the RHS $\to 0$ as $C \to \infty$, $\alpha \to 0$.

Similarly,

$$\begin{aligned}
&\mathbb{P}\Big(-\log\mathbb{P}\Big(\xi_{\varphi_{s^-}} \in \widetilde{\xi} \,\Big|\, T,\xi\Big) < \mathfrak{h}\varphi_{s^+} - C\sqrt{\varphi_{s^-}\mathfrak{V}} \,\Big|\, T_0\Big) \\
\leq\ & \mathbb{P}\left(-\log\mathbb{P}\Big(\xi_{\varphi_{s^-}} \in \widetilde{\xi} \,\Big|\, T,\xi\Big) < \mathfrak{h}\varphi_{s^-} - \frac{1}{2}C\sqrt{\varphi_{s^-}\mathfrak{V}} \,\middle|\, T_0\right) \\
+\ & \mathbb{P}\left(\mathfrak{h}\left(\varphi_{s^+} - \varphi_{s^-}\right) \geq \frac{1}{2}C\sqrt{\mathfrak{V}\varphi_{s^-}} \,\middle|\, T_0\right).
\end{aligned}$$

Again both terms on RHS $\to 0$ as $C \to \infty$. □

Proof of Lemma 2.21. The conditioning on T_0 will be assumed throughout, but often dropped from notation. As in the proof of Proposition 2.20 let $Y_i = -\log\frac{\mathbb{P}_\rho(\xi_{\varphi_i}\in\widetilde{\xi}\,|\,\xi,T)}{\mathbb{P}_\rho(\xi_{\varphi_{i-1}}\in\widetilde{\xi}\,|\,\xi,T)}$ for $i \geq 1$ and let $Y_0 = -\log\mathbb{P}_\rho\Big(\xi_{\varphi_0} \in \widetilde{\xi} \,\Big|\, \xi, T\Big)$. Note that

$$\frac{\mathbb{P}_\rho\Big(\xi_{\varphi_i} \in \widetilde{\xi} \,\Big|\, \xi,T\Big)}{\mathbb{P}_\rho\Big(\xi_{\varphi_{i-1}} \in \widetilde{\xi} \,\Big|\, \xi,T\Big)} = \mathbb{P}_\rho\Big(\xi_{\varphi_i} \in \widetilde{\xi} \,\Big|\, \xi, T, \xi_{\varphi_{i-1}} \in \widetilde{\xi}\Big) = \mathbb{P}\big(X_{\sigma_i} \in \xi(i) \,\big|\, (X_t)_{t\geq\sigma_{i-1}}, T(X_{\sigma_{i-1}})\big).$$

where $\xi(i)$ is the loop-erasure of a random walk X^i on $T(X_{\sigma_{i-1}})$ started from $X_{\sigma_{i-1}}$.

Then

$$\begin{aligned}
\mathrm{Var}\left(-\log\mathbb{P}_\rho\Big(\xi_{\varphi_k} \in \widetilde{\xi} \,\Big|\, \xi,T\Big) \,\Big|\, T_0\right) \quad &= \quad \mathrm{Var}\left(\sum_{i=0}^{k} Y_i \,\middle|\, T_0\right) \qquad (2) \\
= \quad \sum_{i=2}^{k}\mathrm{Var}\left(Y_i \mid T_0\right) \quad &+ \quad \mathrm{Var}\left(Y_0 + Y_1 \mid T_0\right) \\
+ \quad 2\sum_{i=2}^{k}\sum_{j=i+1}^{k}\mathrm{Cov}\left(Y_i, Y_j \mid T_0\right) \quad &+ \quad 2\sum_{i=2}^{k}\mathrm{Cov}\left(Y_i, Y_0 + Y_1 \mid T_0\right).
\end{aligned}$$

We will now bound each of the terms on the right-hand side.

---

[5] $\mathfrak{h} \asymp \sqrt{\mathfrak{V}}$ holds by assumption. $\mathbb{P}\big(\varphi_{s^+} - \varphi_{s^-} \geq u\,(s^+ - s^-)\big) \leq \frac{\mathbb{E}[\varphi_{s^+} - \varphi_{s^-}\,|\,T_0]}{u(s^+ - s^-)} = \frac{\mathbb{E}[\varphi_2 - \varphi_1]}{u} \to 0$ as $u \to \infty$. $\mathbb{P}\big(\varphi_{s^+} \geq vs^+\big) \leq \frac{E[\varphi_{s^+}]}{vs^+} \leq \frac{K + \mathbb{E}[\varphi_1 - \varphi_0] + (s^+ - 1)\mathbb{E}[\varphi_2 - \varphi_1]}{vs^+} \lesssim \frac{K + s^+}{vs^+} \lesssim \frac{1}{v} \to 0$ as $v \to \infty$. Here we used that $s^+ \asymp (k - K) \gtrsim K$, which is true by the assumption that $\log b(R) \gtrsim \mathfrak{h} \asymp \sqrt{\mathfrak{V}}$.

Bounding $\sum_{i=2}^{k} \mathrm{Var}\left(Y_i \mid T_0\right)$*:*

Note that for each $i \geq 2$ we have $(T(X_{\sigma_{i-1}}), X_{\sigma_i}, \xi(i))\big|T_0 \stackrel{\mathrm{d}}{=} (T', X'_{\sigma'_1}, \xi')$ where T', X', σ'_1 are defined as in Definition 2.10 and ξ' is a loop-erased random walk on T' independently of X'.

Therefore the random variables $(Y_i|T_0)_{i\geq 2}$ have the same distribution as $-\log \mathbb{P}_\rho\Big(X'_{\sigma'_1} \in \xi' \;\Big|\; T', X'\Big)$. Hence

$$\sum_{i=2}^{k} \mathrm{Var}\left(Y_i \mid T_0\right) \quad \leq \quad k\mathfrak{V}.$$

Bounding $\mathrm{Var}\left(Y_0 + Y_1 \mid T_0\right)$*:*

Let A_{K+r} denote the set of long-range edges between levels $K+r-1$ and $K+r$ of T. From Lemma 2.8 we know that for any $r \geq 1$, $e \in A_{K+r}$ we have $\mathbb{P}((X_{\sigma_1 - 1}, X_{\sigma_1}) = e) \lesssim (2\delta^2)^{r-1}\mathbb{P}(\xi_{K+r} = e)$. We also know that for any $e \in A_K$ we have $\mathbb{P}((X_{\sigma_1 - 1}, X_{\sigma_1}) = e) \leq \mathbb{P}(\xi_K = e)$. Hence

$$\begin{aligned}
\mathrm{Var}\left(Y_0 + Y_1 \mid T_0\right) \quad &\leq \quad \mathbb{E}\big[(Y_0+Y_1)^2 \mid T_0\big] \quad = \quad \mathbb{E}\left[\Big(-\log \mathbb{P}_\rho\Big(X_{\sigma_1} \in \widetilde{\xi} \;\Big|\; X, T\Big)\Big)^2 \;\middle|\; T_0\right] \\
&\lesssim \quad \mathbb{E}\left[\mathbb{E}\left[\sum_{r\geq 0}\sum_{e \in A_{K+r}} \mathbb{P}_\rho\big(X_{\sigma_1} = e^+\big)\left(-\log \mathbb{P}_\rho(\xi_{K+r} = e)\right)^2 \;\middle|\; T\right] \;\middle|\; T_0\right] \\
\lesssim \quad &\mathbb{E}\left[\mathbb{E}\left[\sum_{r\geq 1}(2\delta^2)^{r-1} \sum_{e \in A_{K+r}} \mathbb{P}_\rho(\xi_{K+r} = e)\left(-\log \mathbb{P}_\rho(\xi_{K+r} = e)\right)^2 \;\middle|\; T\right] \;\middle|\; T_0\right] \\
&+ \quad \mathbb{E}\left[\mathbb{E}\left[\sum_{e \in A_K} \mathbb{P}_\rho(\xi_K = e)\left(-\log \mathbb{P}_\rho(\xi_K = e)\right)^2 \;\middle|\; T\right] \;\middle|\; T_0\right].
\end{aligned}$$

We also know that for any $r \geq 0$ we have $\sum_{e\in A_{K+r}} \mathbb{P}_\rho(\xi_{K+r} = e) = 1$ and $|A_{K+r}| \leq b(R)^{K+r+1}$, hence for any realisation of T we have

$$\begin{aligned}
\sum_{e \in A_{K+r}} \mathbb{P}_\rho(\xi_{K+r} = e)\left(-\log \mathbb{P}_\rho(\xi_{K+r} = e)\right)^2 \quad &\leq \quad \log\left(b(R)^{K+r+1}\right)^2 \\
&= \quad \left((K+r+1)\log b(R)\right)^2.
\end{aligned}$$

This then gives

$$\mathrm{Var}\left(Y_0 + Y_1 \mid T_0\right) \lesssim \left(K \log b(R)\right)^2 + \sum_{r\geq 1}(2\delta^2)^{r-1}\left((K+r)\log b(R)\right)^2 \lesssim \left(K \log b(R)\right)^2.$$

Bounding $\sum_{i=2}^{k}\sum_{j=i+1}^{k} \mathrm{Cov}\left(Y_i, Y_j \mid T_0\right)$*:*

We will show that there exist positive constants a and u such that for any $i \geq 2$, $j \geq i+u$ we have

$$\mathrm{Cov}\left(Y_i, Y_j \mid T_0\right) \quad \lesssim \quad \mathfrak{V}e^{-a(j-i)}. \tag{3}$$

Once we established that, we immediately get that $\sum_{i=2}^{k}\sum_{j=i+1}^{k} \mathrm{Cov}\left(Y_i, Y_j \mid T_0\right) \lesssim k\mathfrak{V}$.

In order to prove (3) we will show that for some positive constants a, b and u, for all $i \geq 2$, $j \geq i+u$ there exists a random variable $Y_{i,j}$ and an event $B_{i,j}$ satisfying the following properties.

(i) $B_{i,j}$ and $Y_{i,j}\mathbf{1}_{B_{i,j}}$ are independent of Y_j,

(ii) $\mathbb{P}_{T_0}\left(B_{i,j}^c\right) \lesssim e^{-2a(j-i)}$,

(iii) $|Y_i - Y_{i,j}|\mathbf{1}_{B_{i,j}} \le e^{-b(j-i)}$.

Once we have these variables, we can prove (3) as follows.

$$\begin{aligned}\mathrm{Cov}_{T_0}(Y_i, Y_j) &= \mathbb{E}_{T_0}\left[(Y_i - \mathbb{E}_{T_0}[Y_i])(Y_j - \mathbb{E}_{T_0}[Y_j])\mathbf{1}_{B_{i,j}^c}\right] \\ &+ \mathbb{E}_{T_0}\left[(Y_i - \mathbb{E}_{T_0}[Y_i])(Y_j - \mathbb{E}_{T_0}[Y_j])\mathbf{1}_{B_{i,j}}\right]\end{aligned}$$

Using Cauchy-Schwarz, (ii) and that $\mathbb{E}_{T_0}\left[(Y_j - \mathbb{E}_{T_0}[Y_j])^4\right] \lesssim \mathfrak{V}^2$ for all j [6] we get that

$$\begin{aligned}&\mathbb{E}_{T_0}\left[(Y_i - \mathbb{E}_{T_0}[Y_i])(Y_j - \mathbb{E}_{T_0}[Y_j])\mathbf{1}_{B_{i,j}^c}\right] \\ &\le \sqrt[4]{\mathbb{E}_{T_0}[(Y_i - \mathbb{E}_{T_0}[Y_i])^4]\,\mathbb{E}_{T_0}[(Y_j - \mathbb{E}_{T_0}[Y_j])^4]\,\mathbb{P}_{T_0}\left(B_{i,j}^c\right)^2} \quad \lesssim \quad \mathfrak{V}e^{-a(j-i)}.\end{aligned}$$

Using (i), Cauchy-Schwarz, (iii) and that $\mathbb{E}_{T_0}\left[(Y_j - \mathbb{E}_{T_0}[Y_j])^2\right] \lesssim \mathfrak{V}$ for all j we get that

$$\begin{aligned}\mathbb{E}_{T_0}\left[(Y_i - \mathbb{E}_{T_0}[Y_i])(Y_j - \mathbb{E}_{T_0}[Y_j])\mathbf{1}_{B_{i,j}}\right] &= \mathbb{E}_{T_0}\left[(Y_i - Y_{i,j})(Y_j - \mathbb{E}_{T_0}[Y_j])\mathbf{1}_{B_{i,j}}\right] \\ \le \sqrt{\mathbb{E}_{T_0}\left[(Y_i - Y_{i,j})^2\mathbf{1}_{B_{i,j}}\right]\mathbb{E}_{T_0}[(Y_j - \mathbb{E}_{T_0}[Y_j])^2]} &\lesssim \sqrt{\mathfrak{V}}e^{-b(j-i)}.\end{aligned}$$

This finishes the proof of (3).

Now let us define $Y_{i,j}$ and $B_{i,j}$ and prove that they have the required properties. As before, let X^i be a random walk on $T(X_{\sigma_{i-1}})$ from $X_{\sigma_{i-1}}$ and let $\xi(i)$ be its loop-erasure. Let $\xi(i,j)$ be the loop-erasure of X^i up to the first time that it hits the level φ_{j-1} of $X_{\sigma_{j-1}}$. Let

$$\begin{aligned}&Z_i := \mathbb{P}_{T_0}\left(X_{\sigma_i} \in \xi(i) \mid X, T(X_{\sigma_{i-1}})\right), \quad Z_{i,j} := \mathbb{P}_{T_0}\left(X_{\sigma_i} \in \xi(i,j) \mid X, T(X_{\sigma_{i-1}})\right), \\ &Y_{i,j} := -\log Z_{i,j} \quad \text{and} \quad B_{i,j} := \left\{\varphi_i - \varphi_{i-1} \le j - i,\ d_g(X_{\sigma_{i-1}}, X_{\sigma_i}) \le \frac{j-i}{\varepsilon^2}\right\}.\end{aligned}$$

Also note that $Y_i = -\log Z_i$.

Then by Lemma 2.13 (used for tree $T(X_{\sigma_{i-1}})$), and using that $\sigma_i - \sigma_{i-1} \ge d_g(X_{\sigma_{i-1}}, X_{\sigma_i})$ we have

$$\begin{aligned}\mathbb{P}_{T_0}\left(B_{i,j}^c\right) &\le \mathbb{P}_{T_0}(\varphi_i - \varphi_{i-1} \ge j - i) + \mathbb{P}_{T_0}\left(d_g(X_{\sigma_{i-1}}, X_{\sigma_i}) \ge \frac{j-i}{\varepsilon^2}\right) \\ &\lesssim e^{-c_1(j-i)} + e^{-c_2\frac{j-i}{\varepsilon}}.\end{aligned}$$

This shows (ii).

Note that $B_{i,j}$ and $Y_{i,j}$ only depend on $(X_s)_{s\le\sigma_{j-1}}$ and $T \setminus T(X_{\sigma_{j-1}})$, whereas Y_j depends only on $T(X_{\sigma_{j-1}})$ and the walk $(X_s)_{s\ge\sigma_{j-1}}$ on this tree. From Lemma 2.12 we know that these are independent of each other. This proves (i).

Now we turn to proving (iii). Note that $|Y_i - Y_{i,j}| = |-\log Z_i + \log Z_{i,j}| \le \frac{|Z_i - Z_{i,j}|}{Z_i \wedge Z_{i,j}}$.

[6] This holds because $Y_j|T_0 \overset{\mathrm{d}}{=} Y'$ and we assumed that $\mathbb{E}\left[(Y' - \mathbb{E}[Y'])^4\right] \lesssim \mathbb{E}[Y']^4$.

Let $A_{i,j} = \{X^i \text{ revisits level } \varphi_i \text{ after hitting level } \varphi_{j-1}\}$. Note that

$$\begin{aligned} |Z_i - Z_{i,j}| &\leq \mathbb{P}_{T_0}\big(X_{\sigma_i} \in \xi(i), X_{\sigma_i} \notin \xi(i,j) \bigm| X, T(X_{\sigma_{i-1}})\big) \\ &\quad + \mathbb{P}_{T_0}\big(X_{\sigma_i} \notin \xi(i), X_{\sigma_i} \in \xi(i,j) \bigm| X, T(X_{\sigma_{i-1}})\big) \\ &\leq \mathbb{P}_{T_0}\big(X_{\sigma_i} \in \xi(i), A_{i,j} \bigm| X, T(X_{\sigma_{i-1}})\big) \\ &\quad + \mathbb{P}_{T_0}\big(X_{\sigma_i} \in \xi(i,j), A_{i,j} \bigm| X, T(X_{\sigma_{i-1}})\big). \end{aligned}$$

We will show that on event $B_{i,j}$ the first and second terms on the right-hand side of the line above are $\lesssim \delta^{\frac{j-i}{3}} Z_i$ and $\lesssim \delta^{\frac{j-i}{3}} Z_{i,j}$ respectively, and that $Z_{i,j} \asymp Z_i$. These together immediately imply (iii).

First note that

$$\begin{aligned} \mathbb{P}_{T_0}\big(X_{\sigma_i} \in \xi(i,j), A_{i,j} \bigm| X, T(X_{\sigma_{i-1}})\big) &\leq \mathbb{P}_{T_0}\big(X_{\sigma_i} \in \xi(i,j) \bigm| X, T(X_{\sigma_{i-1}})\big)\, \delta^{\varphi_{j-1}-\varphi_i} \\ &\leq \mathbb{P}_{T_0}\big(X_{\sigma_i} \in \xi(i,j) \bigm| X, T(X_{\sigma_{i-1}})\big)\, \delta^{j-i-1} \quad \lesssim \quad \delta^{\frac{j-i}{3}} Z_{i,j}. \end{aligned}$$

Let $e_1, ..., e_{\varphi_i - \varphi_{i-1}}$ be the long-range edges leading from $X_{\sigma_{i-1}}$ to X_{σ_i} and let $e_0^+ = X_{\sigma_{i-1}}$. Then

$$\mathbb{P}_{T_0}\big(X_{\sigma_i} \in \xi(i), A_{i,j} \bigm| X, T(X_{\sigma_{i-1}})\big) \leq \mathbb{P}\big(X^i \text{ hits level } \varphi_{j-1} \text{ and then hits } X_{\sigma_i}\big)$$

$$\leq \sum_{m=0}^{\varphi_i - \varphi_{i-1} - 1} \left(\prod_{\ell \neq m} \mathbb{P}_{e_\ell^+}\big(X^i \text{ hits } e_{\ell+1}^+\big) \right) \mathbb{P}_{e_m^+}\big(X^i \text{ hits level } \varphi_{j-1} \text{ and then hits } e_{m+1}^+\big). \qquad (4)$$

By Lemma 2.18 and using that $\varphi_{j-1} - \ell(e_m^+) \geq j - i - 1$ we get that

$$\begin{aligned} &\mathbb{P}_{e_m^+}\big(X^i \text{ hits level } \varphi_{j-1} \text{ and then hits } e_{m+1}^+\big) \\ &\lesssim \ell_0 \delta^{j-i-1} \mathbb{P}_{e_m^+}\big(\text{hit } e_{m+1}^+\big) + \delta^{j-i-1} e^{-c_3 \varepsilon \ell_0} \left(\ell_0 + \frac{1}{\varepsilon} \right) \end{aligned}$$

for some positive constant c_3. On event $B_{i,j}$ this is then

$$\lesssim \ell_0 \delta^{j-i-1} \mathbb{P}_{e_m^+}\big(\text{hit } e_{m+1}^+\big) + \delta^{j-i-1} e^{-c_3 \varepsilon \ell_0} \left(\ell_0 + \frac{1}{\varepsilon} \right) \varepsilon^{-1} \Delta^{\frac{j-i}{\varepsilon^2}} \mathbb{P}_{e_m^+}\big(\text{hit } e_{m+1}^+\big).$$

Choosing $\ell_0 = \frac{j-i}{c_3 \varepsilon^3} \log \Delta$ we get that this is

$$\lesssim (j-i) \frac{1}{\varepsilon^3} \delta^{j-i-1} \mathbb{P}_{e_m^+}\big(\text{hit } e_{m+1}^+\big) \lesssim \delta^{\frac{j-i}{2}} \mathbb{P}_{e_m^+}\big(\text{hit } e_{m+1}^+\big).\ [7]$$

Plugging this back to (4) we get that on event $B_{i,j}$

$$\begin{aligned} \mathbb{P}_{T_0}\big(X_{\sigma_i} \in \xi(i), A_{i,j} \bigm| X, T(X_{\sigma_{i-1}})\big) &\lesssim (\varphi_i - \varphi_{i-1}) \delta^{\frac{j-i}{2}} \prod_{\ell} \mathbb{P}_{e_\ell^+}\big(X^i \text{ hits } e_{\ell+1}^+\big) \\ \lesssim (j-i) \delta^{\frac{j-i}{2}} \mathbb{P}\big(X^i \text{ hits } X_{\sigma_i}\big) &\lesssim (j-i) \delta^{\frac{j-i}{2}} \frac{1}{1-\delta} \mathbb{P}(X_{\sigma_i} \in \xi(i)) \\ \lesssim \delta^{\frac{j-i}{3}} \mathbb{P}_{T_0}\big(X_{\sigma_i} \in \xi(i) \bigm| X, T(X_{\sigma_{i-1}})\big) &\asymp \delta^{\frac{j-i}{3}} Z_i. \end{aligned}$$

[7] We used here that $j - i$ is at least some sufficiently large constant.

We also have (on event $B_{i,j}$) that

$$\begin{aligned}
\mathbb{P}_{T_0}\big(X_{\sigma_i} \in \xi(i) \bigm| X, T(X_{\sigma_{i-1}})\big) &\geq (1-\delta)\mathbb{P}_{T_0}\big(X_{\sigma_i} \in \xi(i,j) \bigm| X, T(X_{\sigma_{i-1}})\big)\,, \\
\mathbb{P}_{T_0}\big(X_{\sigma_i} \in \xi(i,j) \bigm| X, T(X_{\sigma_{i-1}})\big) &\geq \mathbb{P}_{T_0}\big(X_{\sigma_i} \in \xi(i), X_{\sigma_i} \in \xi(i,j) \bigm| X, T(X_{\sigma_{i-1}})\big) \\
&\geq \left(1 - c_4 \delta^{\frac{j-i}{3}}\right) \mathbb{P}_{T_0}\big(X_{\sigma_i} \in \xi(i) \bigm| X, T(X_{\sigma_{i-1}})\big)\,,
\end{aligned}$$

hence

$$Z_i = \mathbb{P}_{T_0}\big(X_{\sigma_i} \in \xi(i) \bigm| X, T(X_{\sigma_{i-1}})\big) \asymp \mathbb{P}_{T_0}\big(X_{\sigma_i} \in \xi(i,j) \bigm| X, T(X_{\sigma_{i-1}})\big) = Z_{i,j}.$$

This finishes the proof of (iii).

Bounding $\sum_{i=2}^{k} \operatorname{Cov}(Y_i, Y_0 + Y_1 \mid T_0)$*:*

By Cauchy-Schwarz and the previous bounds we have

$$\begin{aligned}
\sum_{i=2}^{k} \operatorname{Cov}(Y_i, Y_0 + Y_1 \mid T_0) &\leq \sqrt{\operatorname{Var}_{T_0}\left(\sum_{i=2}^{k} Y_i\right) \operatorname{Var}_{T_0}(Y_0 + Y_1)} \\
&\lesssim \sqrt{k\mathfrak{V} \cdot (K \log b(R))^2} \leq k\mathfrak{V} + (K \log b(R))^2 . \square
\end{aligned}$$

II Estimating the entropy (Lemma 2.22)

The proof of Lemma 2.22 follows immediately from the following four results.

Lemma II.1. *Let G be as before. Let ρ be any vertex of G and let T be any realisation of the random quasi-tree corresponding to G, rooted at ρ. Let v be any vertex of G such that the ball of radius $\frac{R}{2}$ around v is contained in the ball of radius R around ρ (the balls are in graph distance). Let X and $\widetilde{X}$ be independent random walks on T from v and let τ_1 be the first time when X hits level 1 and $\tilde{\tau}_1$ be the first time when $\widetilde{X}$ hits level 1. Let Y be a simple random walk on G from v and let E be a random variable taking values on $\mathbb{Z}_{\geq 0}$ such that $\mathbb{P}_v(E = k \mid E \geq k, Y_k = u) = \frac{\varepsilon}{\deg(u)+\varepsilon}$ for all k and all u. Let $\left(\widetilde{Y}, \widetilde{E}\right)$ be an independent copy of (Y, E). Let $b \in \mathbb{Z}_{\geq 1}$.*

Assume the following three properties hold.

(i) There exists h_b such that for every choice of v we have

$$\mathbb{E}\left[\left(-\log \mathbb{P}_v\left(Y_E = \widetilde{Y}_{\widetilde{E}} \,\middle|\, Y\right)\right)^b \,\middle|\, v\right] \asymp h_b.$$

(ii) The size of all R-balls in G is upper bounded by a $b(R)$ satisfying

$$(\log b(R))^b \left(1 - \frac{\varepsilon}{\Delta + \varepsilon}\right)^{\frac{R}{2}} \ll h_b.$$

(iii) $R \gg \frac{1}{\varepsilon}$.

Then we have

$$\mathbb{E}\left[\left(-\log \mathbb{P}_v\left(X_{\tau_1} = \widetilde{X}_{\tilde{\tau}_1} \,\middle|\, X, T\right)\right)^b \,\middle|\, T, v\right] \asymp h_b. \tag{5}$$

(Here h_b and $b(R)$ can both depend on n.)

Proof. Let X^a be a random walk on T^a. For a vertex u let $Y^{(u)}$ denote a walk that starts from u and has transition probabilities as Y. Let us define $X^{(u)}$ and $X^{a,(u)}$ analogously. First we will compare Y_E to $X^a_{\tau_1^a}$ and then we compare $X^a_{\tau_1^a}$ to X_{τ_1}.

Since the ball of radius $\frac{R}{2}$ around v is contained in the R-ball of ρ in T, we have

$$\left((Y_{k\wedge E})_{k=1}^{R/2}, E\wedge\frac{R}{2}\right) \overset{\mathrm{d}}{=} \left((X^a_{k\wedge(\tau_1^a-1)})_{k=1}^{R/2}, (\tau_1^a-1)\wedge\frac{R}{2}\right).$$

In particular

$$\sum_e \mathbb{P}_v\left(Y_E=e^-, E<\frac{R}{2}\right)\left(-\log\mathbb{P}_v\left(Y_E=e^-, E<\frac{R}{2}\right)\right)^b$$

$$= \sum_e \mathbb{P}_v\left(X^a_{\tau_1^a-1}=e^-, \tau_1^a-1<\frac{R}{2}\right)\left(-\log\mathbb{P}_v\left(X^a_{\tau_1^a-1}=e^-, \tau_1^a-1<\frac{R}{2}\right)\right)^b. \tag{6}$$

We will show that both $H_b(Y_E^{(v)})$ and $H_b(X_{\tau_1}^{a,(v)})$ are at distance $\ll h_b$ from this sum. Once we established that, it is immediate to see that $H_b(X_{\tau_1}^{a,(v)}) \asymp h_b$.

First let us consider $H_b(Y_E^{(v)})$. By properties (i) and (ii) in Lemma A.4 we get that

$$H_b(Y_E^{(v)}) \geq \sum_e \mathbb{P}_v\left(Y_E=e^-, E<\frac{R}{2}\right)\left(-\log\mathbb{P}_v\left(Y_E=e^-, E<\frac{R}{2}\right)\right)^b$$

and

$$\begin{aligned} H_b(Y_E^{(v)}) \leq & \sum_e \mathbb{P}_v\left(Y_E=e^-, E<\frac{R}{2}\right)\left(-\log\mathbb{P}_v\left(Y_E=e^-, E<\frac{R}{2}\right)\right)^b \\ & +\sum_e \mathbb{P}_v\left(Y_E=e^-, E\geq\frac{R}{2}\right)\left(-\log\mathbb{P}_v\left(Y_E=e^-, E\geq\frac{R}{2}\right)\right)^b \end{aligned}$$

hence

$$\begin{aligned} &\left|H_b(Y_E^{(v)}) - \sum_e \mathbb{P}_v\left(Y_E=e^-, E<\frac{R}{2}\right)\left(-\log\mathbb{P}_v\left(Y_E=e^-, E<\frac{R}{2}\right)\right)^b\right| \\ &\leq \sum_e \mathbb{P}_v\left(Y_E=e^-, E\geq\frac{R}{2}\right)\left(-\log\mathbb{P}_v\left(Y_E=e^-, E\geq\frac{R}{2}\right)\right)^b. \end{aligned}$$

Note that $\left(Y_E^{(v)}|E\geq k, Y_k=u\right) \overset{\mathrm{d}}{=} Y_E^{(u)}$, hence

$$\mathbb{P}_v\left(Y_E=e^-, E\geq\frac{R}{2}\right) = \mathbb{P}\left(E\geq\frac{R}{2}\right)\sum_u \mathbb{P}_v\left(Y_{\frac{R}{2}}=u \,\middle|\, E\geq\frac{R}{2}\right)\mathbb{P}_u\big(Y_E=e^-\big),$$

therefore (using properties (iii) and (iv) in Lemma A.4 and all three assumptions of Lemma II.1) we get that

$$\begin{aligned} &\sum_e \mathbb{P}_v\left(Y_E=e^-, E\geq\frac{R}{2}\right)\left(-\log\mathbb{P}_v\left(Y_E=e^-, E\geq\frac{R}{2}\right)\right)^b \\ &\lesssim \mathbb{P}\left(E\geq\frac{R}{2}\right)\left(H_b\left(\left(Y_{\frac{R}{2}}^{(v)}\middle| E\geq\frac{R}{2}\right)\right)+\sum_u \mathbb{P}_v\left(Y_{\frac{R}{2}}=u \,\middle|\, E\geq\frac{R}{2}\right)H_b\left(Y_E^{(u)}\right)\right) \\ &\lesssim \left(1-\frac{\varepsilon}{\Delta+\varepsilon}\right)^{\frac{R}{2}}\left((\log b(R))^b+h_b\right) \ll h_b \asymp H_b\left(Y_E^{(v)}\right). \end{aligned}$$

In particular it shows that the sum in (6) is $\asymp h_b$.

Now let us proceed to approximating $H_b(X^{a,(v)}_{\tau_1^a}) = H_b(X^{a,(v)}_{\tau_1^a-1})$. As before we get that

$$\begin{aligned}&\left| H_b(X^{a,(v)}_{\tau_1^a-1}) - \sum_e \mathbb{P}_v\Big(Y_E = e^-, E < \frac{R}{2}\Big)\Big(-\log \mathbb{P}_v\Big(Y_E = e^-, E < \frac{R}{2}\Big)\Big)^b\right| \\ \leq\ & \sum_e \mathbb{P}_v\Big(X^a_{\tau_1^a-1} = e^-, \tau_1^a - 1 \geq \frac{R}{2}\Big)\Big(-\log \mathbb{P}_v\Big(X^a_{\tau_1^a-1} = e^-, \tau_1^a - 1 \geq \frac{R}{2}\Big)\Big)^b \\ \lesssim\ & \mathbb{P}\Big(\tau_1^a - 1 \geq \frac{R}{2}\Big) H_b\Big(\Big(X^{a,(v)}_{\tau_1^a-1}\Big|\tau_1^a \geq \frac{R}{2}\Big)\Big) \lesssim \Big(1 - \frac{\varepsilon}{\Delta+\varepsilon}\Big)^{\frac{R}{2}} (\log b(R))^b \ll h_b.\end{aligned}$$

This finishes the proof of $H_b(X^{a,(v)}_{\tau_1}) \asymp h_b$.

Let $X^{(v)}$ be the restriction of $X^{a,(v)}$ to T (which is indeed distributed as a random walk on T). Then $X^{(v)}_{\tau_1} \overset{\mathrm{d}}{=} \Big(X^{a,(v)}_{\tau_1^a}\Big| X^{a,(v)}_{\tau_1^a} \neq \rho^a\Big)$. From Lemma 2.4 we know that $\mathbb{P}_v\Big(X^a_{\tau_1^a} \neq \rho^a\Big) = 1 - o(1)$. Together these show that

$$\begin{aligned} H_b(X^{(v)}_{\tau_1}) &= \sum_e \mathbb{P}_v\Big(X^a_{\tau_1^a} = e^+ \;\Big|\; X^a_{\tau_1^a} \neq \rho^a\Big)\Big(-\log \mathbb{P}_v\Big(X^a_{\tau_1^a} = e^+ \;\Big|\; X^a_{\tau_1^a} \neq \rho^a\Big)\Big)^b \\ &= \sum_e \frac{\mathbb{P}_v\Big(X^a_{\tau_1^a} = e^+\Big)}{\mathbb{P}_v\Big(X^a_{\tau_1^a} \neq \rho^a\Big)}\left(-\log \frac{\mathbb{P}_v\Big(X^a_{\tau_1^a} = e^+\Big)}{\mathbb{P}_v\Big(X^a_{\tau_1^a} \neq \rho^a\Big)}\right)^b \\ &\asymp \sum_e \mathbb{P}_v\Big(X^a_{\tau_1^a} = e^+\Big)\Big(-\log \mathbb{P}_v\Big(X^a_{\tau_1^a} = e^+\Big)\Big)^b + 1 \\ &= H_b(X^{a,(v)}_{\tau_1^a}) + 1 - \mathbb{P}_v\Big(X^a_{\tau_1^a} = \rho^a\Big)\Big(-\log \mathbb{P}_v\Big(X^a_{\tau_1^a} = \rho^a\Big)\Big)^b \asymp H_b(X^{a,(v)}_{\tau_1^a}) \asymp h_b \end{aligned}$$

where the sums are taken over all long-range edges e of T from the R-ball of ρ. This finishes the proof. ◻

Lemma II.2. *Let us consider the setup of Lemma II.1 and assume that the assumptions also hold for $R = n$, with the same values of h_b. Let ξ and $\widetilde{\xi}$ be independent loop-erased random walks on T. Then we have*

$$\mathbb{E}\Big[\Big(-\log \mathbb{P}_\rho\Big(\xi_1 = \widetilde{\xi}_1 \;\Big|\; \xi, T\Big)\Big)^b \;\Big|\; T\Big] \asymp \mathbb{E}\Big[\Big(-\log \mathbb{P}_\rho\Big(X_{\tau_1} = \widetilde{X}_{\widetilde{\tau}_1} \;\Big|\; X, T\Big)\Big)^b \;\Big|\; T\Big]. \tag{7}$$

Proof. We first present the proof of the $\gtrsim$ direction, which is very quick. Then we will prove the $\lesssim$ direction in case $R = n$. Finally we use this result to conclude the $\lesssim$ direction for any R satisfying assumptions (ii) and (iii) in Lemma II.1. To simplify formulae we will sometimes drop the conditioning on T from the notation, but it is assumed throughout.

For any long-range edge e from the R-ball of ρ in T we have $\mathbb{P}_\rho(\xi_1 = e \mid T) \geq (1-\delta)\mathbb{P}_\rho(X_{\tau_1} = e \mid T)$, hence

$$\begin{aligned}&\sum_e \mathbb{P}_\rho(\xi_1 = e \mid T)\,(-\log \mathbb{P}_\rho(\xi_1 = e \mid T))^b \\ &\qquad \gtrsim \sum_e \mathbb{P}_\rho(X_{\tau_1} = e \mid T)\,(-\log \mathbb{P}_\rho(X_{\tau_1} = e \mid T))^b \asymp h_b.\end{aligned}$$

This proves the $\gtrsim$ direction.

Now let us assume that $R = n$ and prove the $\lesssim$ direction. In this case the R-ball B of ρ in T is the whole graph G, so (5) holds for all vertices v in B.

For any u in B and any $k \geq 1$ let $A_{k,u}$ be the event that X leaves B and returns to it at least k times and the kth return is at vertex u. For $k = 0$ we take $A_{0,u}$ such that $\mathbb{P}(A_{0,\rho}) = \mathbf{1}_{u=\rho}$.

Then for any long-range edge e from B we have

$$\mathbb{P}_\rho(\xi_1 = e) \quad = \quad \sum_{k\geq 0}\sum_u \mathbb{P}_\rho(A_{k,u})\,\mathbb{P}_u\big(X_{\tau_1} = e^+\big)\,\mathbb{P}_{e^+}(\tau_{e^-} = \infty)\,.$$

Note that $\sum_{k\geq 0}\mathbb{P}_\rho(A_{k,u}) = \mathbb{E}_\rho[a_u]$ where $a_u = \#(\text{returns of } X \text{ to } B \text{ via } u) + \mathbf{1}_{u=\rho}$. We know that

- $\sum_u \mathbb{E}_\rho[a_u] = \mathbb{E}[\#(\text{returns of } X \text{ to } B)] \leq \mathbb{E}[\mathrm{Geom}_{\geq 1}(1-\delta)] \leq \frac{1}{1-\delta}$,
- $\sum_e \mathbb{P}_u(X_{\tau_1} = e^+) = 1$,
- $\sum_e \mathbb{P}_u(X_{\tau_1} = e^+)\,(-\log \mathbb{P}_u(X_{\tau_1} = e^+))^b \asymp h_b$,
- $1 - \delta \leq \mathbb{P}_{e^+}(\tau_{e^-} = \infty) \leq 1$.

This then gives

$$\mathbb{E}\left[\left(-\log \mathbb{P}_\rho\left(\xi_1 = \widetilde{\xi}_1 \,\middle|\, \xi, T\right)\right)^b \,\middle|\, T\right] \quad = \quad \sum_e \mathbb{P}_\rho(\xi_1 = e)\,(-\log \mathbb{P}_\rho(\xi_1 = e))^b$$

$$= \sum_e \left(\sum_u \mathbb{E}_\rho[a_u]\,\mathbb{P}_u\big(X_{\tau_1} = e^+\big)\,\mathbb{P}_{e^+}(\tau_{e^-} = \infty)\right)\left(-\log\left(\sum_u \mathbb{E}_\rho[a_u]\,\mathbb{P}_u\big(X_{\tau_1} = e^+\big)\,\mathbb{P}_{e^+}(\tau_{e^-} = \infty)\right)\right)^b$$
$$\leq \sum_e\sum_u \mathbb{E}_\rho[a_u]\,\mathbb{P}_u\big(X_{\tau_1} = e^+\big)\,\mathbb{P}_{e^+}(\tau_{e^-} = \infty)\,\big(-\log\big(\mathbb{E}_\rho[a_u]\,\mathbb{P}_u\big(X_{\tau_1} = e^+\big)\,\mathbb{P}_{e^+}(\tau_{e^-} = \infty)\big)\big)^b$$

$$\asymp \quad \sum_u \mathbb{E}_\rho[a_u]\sum_e \mathbb{P}_u\big(X_{\tau_1} = e^+\big)\,\mathbb{P}_{e^+}(\tau_{e^-} = \infty)\,(-\log \mathbb{P}_{e^+}(\tau_{e^-} = \infty))^b \tag{8}$$
$$+ \quad \sum_u \mathbb{E}_\rho[a_u]\sum_e \Big(\mathbb{P}_u\big(X_{\tau_1} = e^+\big)\,\big(-\log \mathbb{P}_u\big(X_{\tau_1} = e^+\big)\big)^b\Big)\,\mathbb{P}_{e^+}(\tau_{e^-} = \infty) \tag{9}$$
$$+ \quad \sum_u \Big(\mathbb{E}_\rho[a_u]\,(-\log \mathbb{E}_\rho[a_u])^b\Big)\sum_e \mathbb{P}_u\big(X_{\tau_1} = e^+\big)\,\mathbb{P}_{e^+}(\tau_{e^-} = \infty)\,. \tag{10}$$

The first term (8) on the right-hand side is $\lesssim 1$ and the second one (9) is $\lesssim 1$. We will prove that

$$\sum_u \mathbb{E}_\rho[a_u]\,(-\log \mathbb{E}_\rho[a_u]) \quad \lesssim \quad h_b. \tag{11}$$

Once we establish that, it follows that the third term (10) is $\lesssim h_b$, hence
$\mathbb{E}\left[\left(-\log \mathbb{P}_\rho\left(\xi_1 = \widetilde{\xi}_1 \,\middle|\, \xi\right)\right)^b\right] \lesssim h_b$ as required. Let us proceed to prove (11).
We know that

$$\mathbb{E}_\rho[a_u] - \mathbf{1}_{u=\rho} = \sum_{k\geq 1}\mathbb{P}_\rho(A_{k,u})\,,$$

hence

$$\sum_u \mathbb{E}_\rho[a_u]\,(-\log \mathbb{E}_\rho[a_u])^b \quad \lesssim \quad \sum_{k\geq 1}\sum_u \mathbb{P}_\rho(A_{k,u})\,(-\log \mathbb{P}_\rho(A_{k,u}))^b\,.$$

We will prove by induction on k that for each $k \geq 1$ we have

$$\sum_u \mathbb{P}_\rho(A_{k,u})\,(-\log \mathbb{P}_\rho(A_{k,u}))^b \quad \lesssim \quad k\delta^k h_b$$

For any vertex w of B we have $\mathbb{P}_w(A_{1,u}) = \sum_u \mathbb{P}_w(X_{\tau_1} = u)\,\mathbb{P}_{u^+}(\tau_u < \infty)$, hence

$$\begin{aligned}
&\sum_u \mathbb{P}_w(A_{1,u})\,(-\log \mathbb{P}_w(A_{1,u}))^b \\
&\asymp \quad \sum_u \mathbb{P}_w\big(X_{\tau_1} = u^+\big)\,\big(-\log \mathbb{P}_w\big(X_{\tau_1} = u^+\big)\big)^b\,\mathbb{P}_{u^+}(\tau_u < \infty) \\
&\quad + \quad \sum_u \mathbb{P}_w(X_{\tau_1} = u)\,\mathbb{P}_{u^+}(\tau_u < \infty)\,(-\log \mathbb{P}_{u^+}(\tau_u < \infty))^b \\
&\leq \quad \delta \sum_u \mathbb{P}_w\big(X_{\tau_1} = u^+\big)\,\big(-\log \mathbb{P}_w\big(X_{\tau_1} = u^+\big)\big)^b \,+\, \delta(-\log\delta)\sum_u \mathbb{P}_w\big(X_{\tau_1} = u^+\big) \,\lesssim\, \delta h_b,
\end{aligned}$$

which is the required bound for $k = 1$.
Also for all $k \geq 1$ we have $\sum_u \mathbb{P}_w(A_{k,u}) = \mathbb{P}_w(X \text{ leaves } B \text{ and returns } \geq k \text{ times}) \leq \delta^k$.
Note that $\mathbb{P}_\rho(A_{k,u}) = \sum_w \mathbb{P}_\rho(A_{k-1,w})\,\mathbb{P}_w(A_{1,u})$, hence (assuming the bound for $k-1$) we get

$$\begin{aligned}
&\sum_u \mathbb{P}_\rho(A_{k,u})\,(-\log \mathbb{P}_\rho(A_{k,u}))^b \\
&\leq \quad \sum_w \sum_u \mathbb{P}_\rho(A_{k-1,w})\,\mathbb{P}_w(A_{1,u})\,(-\log(\mathbb{P}_\rho(A_{k-1,w})\,\mathbb{P}_w(A_{1,u})))^b \\
&\asymp \quad \sum_w \mathbb{P}_\rho(A_{k-1,w})\,(-\log \mathbb{P}_\rho(A_{k-1,w}))^b \sum_u \mathbb{P}_w(A_{1,u}) \\
&\qquad + \quad \sum_w \mathbb{P}_\rho(A_{k-1,w}) \sum_u \mathbb{P}_w(A_{1,u})\,(-\log \mathbb{P}_w(A_{1,u}))^b \\
&\lesssim \quad (k-1)\delta^{k-1}\delta h_b \quad + \quad \delta^{k-1}\delta h_b \quad \asymp \quad k\delta^k h_b.
\end{aligned}$$

Therefore

$$\sum_u \mathbb{E}_\rho[a_u]\,(-\log \mathbb{E}_\rho[a_u]) \quad \leq \quad \sum_{k\geq 1}\sum_u \mathbb{P}_\rho(A_{k,u})\,(-\log \mathbb{P}_\rho(A_{k,u})) \quad \lesssim \quad \sum_{k\geq 1} k\delta^k h_b \quad \lesssim \quad h_b.$$

This finishes the proof of the $\lesssim$ direction in (7) in case $R = n$.

Now let us consider any R satisfying assumptions (ii) and (iii) in Lemma II.1. Let B be the R-ball of ρ in T. Let $\widetilde{T}$ be a realisation of the random quasi-tree with root ρ, with radius n, containing T as a subtree. Let $\widetilde{B}$ be the ball of ρ in $\widetilde{T}$. Let $\hat{T}$ be a graph obtained by removing the long-range edges of $\widetilde{T}$ that start from the vertices in $\widetilde{B} \setminus B$ and removing any parts that got disconnected from ρ. Note that T is a subgraph of $\hat{T}$. Let $\widetilde{X}$ be a random walk on $\widetilde{T}$ and let $\widetilde{\xi}$ be its loop-erasure. Note that the restriction of $\widetilde{X}$ to $\hat{T}$ is a random walk on $\hat{T}$, but it is possible that it only runs for a finite number of steps (if $\widetilde{\xi}_1 \in B \setminus B'$). Let $\hat{X}$ be a random walk on $\hat{T}$ from ρ such that in case $\widetilde{\xi}_1 \in B \setminus B'$ it agrees with the restriction of $\widetilde{X}$ to $\hat{T}$ and otherwise it is independent of $\widetilde{X}$. Let $\hat{\xi}$ be the loop-erasure of $\hat{X}$. Note that for any long-range edge e in $\widetilde{T}$ from B' we have

$$\begin{aligned}
\mathbb{P}\Big(\hat{\xi}_1 = e\Big) \quad &= \quad \mathbb{P}\Big(\hat{\xi}_1 = e, \widetilde{\xi}_1 \notin B'\Big) + \mathbb{P}\Big(\hat{\xi}_1 = e, \widetilde{\xi}_1 \in B'\Big) \\
= \mathbb{P}\Big(\widetilde{\xi}_1 \notin B'\Big)\,\mathbb{P}\Big(\hat{\xi}_1 = e \,\Big|\, \widetilde{\xi}_1 \notin B'\Big) + \mathbb{P}\Big(\widetilde{\xi}_1 = e\Big) \;&=\; \mathbb{P}\Big(\widetilde{\xi}_1 \notin B'\Big)\,\mathbb{P}\Big(\hat{\xi}_1 = e\Big) + \mathbb{P}\Big(\widetilde{\xi}_1 = e\Big),
\end{aligned}$$

hence

$$\mathbb{P}\Big(\hat{\xi}_1 = e\Big) \quad = \quad \frac{\mathbb{P}\Big(\widetilde{\xi}_1 = e\Big)}{1 - \mathbb{P}\Big(\widetilde{\xi}_1 \notin B'\Big)} \quad = \quad \mathbb{P}\Big(\widetilde{\xi}_1 = e \,\Big|\, \widetilde{\xi}_1 \in B'\Big).$$

This means that $\hat{\xi}_1 \overset{\mathrm{d}}{=} \Big(\widetilde{\xi}_1 \Big| \widetilde{\xi}_1 \in B'\Big)$.

Let X be the restriction of $\hat{X}$ to T, which is a random walk on T. Let ξ be the loop-erasures of X. Then $\xi = \hat{\xi}$.

We also know that $1 \geq \mathbb{P}\Big(\widetilde{\xi}_1 \in B'\Big) \geq \mathbb{P}(\tau_1 < R)\,(1-\delta) \gtrsim 1$. Overall this shows that

$$\begin{aligned}\sum_{e \in B'} \mathbb{P}_\rho(\xi_1 = e)\,(-\log \mathbb{P}_\rho(\xi_1 = e))^b \quad &\asymp \quad \sum_{e \in B'} \mathbb{P}_\rho\Big(\widetilde{\xi}_1 = e\Big)\Big(-\log \mathbb{P}_\rho\Big(\widetilde{\xi}_1 = e\Big)\Big)^b \\ &\leq \quad \sum_{e \in B} \mathbb{P}_\rho\Big(\widetilde{\xi}_1 = e\Big)\Big(-\log \mathbb{P}_\rho\Big(\widetilde{\xi}_1 = e\Big)\Big)^b \quad \asymp \quad h_b.\end{aligned}$$

This finishes the proof. □

Lemma II.3. *Let G be as before. Let ρ be any vertex of G and let T be any realisation of the random quasi-tree corresponding to G, rooted at ρ. Let ξ and $\widetilde{\xi}$ be independent loop-erased random walks on T. Also let T' be any realisation of the conditioned random quasi-tree corresponding to G as in Definition 2.10, rooted at ρ. Let ξ' and $\widetilde{\xi}'$ be independent loop-erased random walks on T'. Let $b \in \mathbb{Z}_{\geq 1}$. Assume that there exist h_b such that for any choice of ρ and T we have*

$$\mathbb{E}\Big[\Big(-\log \mathbb{P}_\rho\Big(\xi_1 = \widetilde{\xi}_1 \,\Big|\, \xi, T\Big)\Big)^b \,\Big|\, T\Big] \quad \asymp \quad h_b. \tag{12}$$

Then we have

$$\mathbb{E}\Big[\Big(-\log \mathbb{P}_\rho\Big(\xi'_1 = \widetilde{\xi}'_1 \,\Big|\, \xi', T'\Big)\Big)^b \,\Big|\, T'\Big] \quad \asymp \quad h_b. \tag{13}$$

Proof. For any quasi-tree t we have $\Big(\xi', \widetilde{\xi}' \Big| T' = t\Big) \overset{\mathrm{d}}{=} \Big(\xi, \widetilde{\xi} \Big| T = t\Big)$. The result follows from assumption (12). □

Lemma II.4. *Let G be as before. Let ρ be any vertex of G and let T be any realisation of the random quasi-tree corresponding to G, rooted at ρ. Let ξ and $\widetilde{\xi}$ be independent loop-erased random walks on T.*

Also let T', X' and ξ' be as in Definition 2.10, with T' rooted at ρ. Let $\Big(\widetilde{X}', \widetilde{\xi}'\Big)$ be an independent copy of (X', ξ') given T'.

Let $b \in \mathbb{Z}_{\geq 1}$. Assume that there exist h_b such that for any choice of ρ and T we have

$$\mathbb{E}\Big[\Big(-\log \mathbb{P}_\rho\Big(\xi_1 = \widetilde{\xi}_1 \,\Big|\, \xi, T\Big)\Big)^b \,\Big|\, T\Big] \quad \asymp \quad h_b. \tag{14}$$

Then we have

$$\mathbb{E}\Big[\Big(-\log \mathbb{P}_\rho\Big(X'_{\sigma'_1} \in \widetilde{\xi}' \,\Big|\, X', T'\Big)\Big)^b \,\Big|\, T'\Big] \quad \asymp \quad \mathbb{E}\Big[\Big(-\log \mathbb{P}_\rho\Big(\xi'_1 = \widetilde{\xi}'_1 \,\Big|\, \xi', T'\Big)\Big)^b \,\Big|\, T'\Big]. \tag{15}$$

Proof. For $r \geq 1$ let $A_r(T')$ denote the set of long-range edges of T' between levels $r-1$ and r. Note that

$$\mathbb{E}\left[\left(-\log \mathbb{P}_\rho\left(X'_{\sigma'_1} \in \xi' \;\middle|\; X', T'\right)\right)^b\right]$$

$$= \quad \mathbb{E}\left[\sum_{r\geq 1}\sum_{e\in A_r(T')} \left(-\log \mathbb{P}_\rho\left(\xi'_r = e \mid T'\right)\right)^b \mathbb{P}_\rho\left(X'_{\sigma'_1} = e^+ \;\middle|\; T'\right)\right].$$

We will show that the sum of the terms with $r=1$ is of order $\mathbb{E}\left[\left(-\log \mathbb{P}_\rho\left(\xi'_1 = \widetilde{\xi}'_1 \;\middle|\; \xi', T'\right)\right)^b\right]$, while the sum of the terms with $r \geq 2$ is of strictly smaller order.

From Lemma 2.11 we know that for any $e \in A_1(T')$ we have $\mathbb{P}_\rho\left(X'_{\sigma'_1} = e^+ \;\middle|\; T'\right) \asymp \mathbb{P}_\rho(\xi'_1 = e \mid T')$ and for any $e \in A_r$ $(r \geq 1)$ we have $\mathbb{P}_\rho\left(X'_{\sigma'_1} = e^+ \;\middle|\; T'\right) \lesssim (2\delta^2)^{r-1}\mathbb{P}_\rho(\xi'_r = e \mid T')$. This gives that

$$\mathbb{E}\left[\sum_{e\in A_1(T')} \left(-\log \mathbb{P}_\rho\left(\xi'_1 = e \mid T'\right)\right)^b \mathbb{P}_\rho\left(X'_{\sigma'_1} = e^+ \;\middle|\; T'\right)\right]$$

$$\asymp \mathbb{E}\left[\sum_{e\in A_1(T')} \left(-\log \mathbb{P}_\rho\left(\xi'_1 = e \mid T'\right)\right)^b \mathbb{P}_\rho\left(\xi'_1 = e \mid T'\right)\right] = \mathbb{E}\left[\left(-\log \mathbb{P}_\rho\left(\xi'_1 = \widetilde{\xi}'_1 \;\middle|\; \xi', T'\right)\right)^b\right].$$

We also get that

$$\mathbb{E}\left[\sum_{r\geq 2}\sum_{e\in A_r(T')} \left(-\log \mathbb{P}_\rho\left(\xi'_r = e \mid T'\right)\right)^b \mathbb{P}_\rho\left(X'_{\sigma'_1} = e^+ \;\middle|\; T'\right)\right]$$

$$\lesssim \quad \sum_{r\geq 2}(2\delta^2)^{r-1}\mathbb{E}\left[\sum_{e\in A_r(T')} \left(-\log \mathbb{P}_\rho\left(\xi'_r = e \mid T'\right)\right)^b \mathbb{P}_\rho\left(\xi'_r = e \mid T'\right)\right]. \tag{16}$$

For a given T' and given $e \in A_r(T')$ let $(e_i)_{i=1}^r$ be the long-range edges leading from ρ to e and let $e_0^+ = \rho$. Then we have

$$\begin{aligned}
&\left(-\log \mathbb{P}_\rho\left(\xi'_r = e \mid T'\right)\right)^b \mathbb{P}_\rho\left(\xi'_r = e \mid T'\right)\\
&= \quad \left(\sum_{i=1}^r -\log \mathbb{P}_\rho\left(\xi'_i = e_i \mid T', \xi'_{i-1} = e_{i-1}\right)\right)^b \prod_{i=1}^r \mathbb{P}_\rho\left(\xi'_i = e_i \mid T', \xi'_{i-1} = e_{i-1}\right)\\
&\leq \quad r^{b-1}\sum_{i=1}^r \left(-\log \mathbb{P}_\rho\left(\xi'_i = e_i \mid T', \xi'_{i-1} = e_{i-1}\right)\right)^b \mathbb{P}_\rho\left(\xi'_i = e_i \mid T', \xi'_{i-1} = e_{i-1}\right). \;[8]
\end{aligned} \tag{17}$$

Note that given T', the process ξ' has the distribution of a loop-erased random walk ξ on T'. Hence

$$\mathbb{P}_\rho\left(\xi'_i = e_i \mid T', \xi'_{i-1} = e_{i-1}\right) = \mathbb{P}_\rho\left(\xi_i = e_i \mid T', \xi_{i-1} = e_{i-1}\right) = \mathbb{P}_{e_{i-1}^+}\left(\xi_1^i = e_i \mid T'(e_{i-1}^+)\right)$$

[8] We used here that for $r, b \in \mathbb{Z}_{\geq 1}$ and $a_1, ..., a_r > 0$ we have $\left(\sum_{i=1}^r a_i\right)^b \leq r^{b-1}\sum_{i=1}^r a_i^b$. This can be proved by induction on b and repeated use of the rearrangement inequality.

where ξ^i is a loop-erased random walk on tree $T'(e^+_{i-1})$, started from e^+_{i-1}.

By assumption (14) and by using that ξ' is a loop-erased random walk on T' we know that for any realisation of T' and for any fixed $e_{i-1} \in A_{i-1}(T')$ we have

$$\mathbb{E}\left[\sum_{e_i \in A_1(T'(e^+_{i-1}))} \Big(-\log \mathbb{P}_{e^+_{i-1}}\big(\xi^i_1 = e_i \mid T'(e^+_{i-1})\big)\Big)^b \mathbb{P}_{e^+_{i-1}}\big(\xi^i_1 = e_i \mid T'(e^+_{i-1})\big) \,\middle|\, T'\right]$$
$$\asymp \quad \mathbb{E}\left[\sum_{e \in A_1(T')} \big(-\log \mathbb{P}_\rho\big(\xi'_1 = e \mid T'\big)\big)^b \mathbb{P}_\rho\big(\xi'_1 = e \mid T'\big) \,\middle|\, T'\right].$$

Plugging this back into (16) we get that

$$\mathbb{E}\left[\sum_{r\geq 2}\sum_{e \in A_r(T')} \big(-\log \mathbb{P}_\rho\big(\xi'_r = e \mid T'\big)\big)^b \mathbb{P}_\rho\Big(X'_{\sigma'_1} = e^+ \,\Big|\, T'\Big)\right]$$
$$\lesssim \sum_{r\geq 2}(2\delta^2)^{r-1} r^{b-1}\mathbb{E}\left[\sum_{e \in A_r(T')}\sum_{i=1}^{r} \Big(-\log \mathbb{P}_{e^+_{i-1}}\big(\xi^i_1 = e_i \mid T'(e^+_{i-1})\big)\Big)^b \mathbb{P}_{e^+_{i-1}}\big(\xi^i_1 = e_i \mid T'(e^+_{i-1})\big)\right]$$
$$\asymp \quad \sum_{r\geq 2}(2\delta^2)^{r-1} r^{b}\mathbb{E}\left[\sum_{e \in A_1(T')} \big(-\log \mathbb{P}_\rho\big(\xi'_1 = e \mid T'\big)\big)^b \mathbb{P}_\rho\big(\xi'_1 = e \mid T'\big)\right]$$
$$\ll \quad \mathbb{E}\left[\sum_{e \in A_1(T')} \big(-\log \mathbb{P}_\rho\big(\xi'_1 = e \mid T'\big)\big)^b \mathbb{P}_\rho\big(\xi'_1 = e \mid T'\big)\right].$$

This finishes the proof. □

III Some miscellaneous statements

Proof of Prop 6.5. Let X be a random walk on G and Y be a random walk on G^*, starting from the same vertex. Let us couple them such that they move together until τ_{LR}, the first time that Y crosses a long-range edge. Note that for any $t \asymp t^G_{\mathrm{mix}}(\theta) \ll \frac{1}{\varepsilon}$ we have

$$d_{\mathrm{TV}}\left(P^t_X(x,\cdot), P^t_Y(x,\cdot)\right) \quad \leq \quad \mathbb{P}_x(\tau_{\mathrm{LR}} < t) \quad \ll \quad 1,$$

where the $\ll$ follows from a union bound. Also

$$d_{\mathrm{TV}}\left(\pi_X(\cdot), \pi_Y(\cdot)\right) \quad = \quad \frac{1}{2}\sum_y \left|\frac{\deg(y)}{2|E|} - \frac{\deg(y)+\varepsilon}{2|E|+n\varepsilon}\right| \quad = \quad \sum_y \frac{|2\varepsilon|E| - \varepsilon n \deg(y)|}{4|E|(2|E|+n\varepsilon)}$$
$$\lesssim \quad \varepsilon \cdot n \cdot \frac{n}{n^2} \quad \asymp \quad \varepsilon \quad \ll \quad 1.$$

(Here deg is the degree in G, and E is the set of edges of G.) Therefore for any $t \asymp t^G_{\mathrm{mix}}(\theta)$ we have

$$\left|d_{\mathrm{TV}}\left(P^t_Y(x,\cdot), \pi_Y(\cdot)\right) - d_{\mathrm{TV}}\left(P^t_X(x,\cdot), \pi_X(\cdot)\right)\right|$$
$$\leq \quad d_{\mathrm{TV}}\left(P^t_X(x,\cdot), P^t_Y(x,\cdot)\right) + d_{\mathrm{TV}}\left(\pi_X(\cdot), \pi_Y(\cdot)\right) \quad \ll \quad 1.$$

This shows that Y has cutoff if and only if X does. □

Proof of Lemma 6.11. From [9, Theorem 1.4] we know that

$$t_{\text{ave}}\left(\frac{1}{4}\right) := \quad \min\left\{t:\ \max_x d_{\text{TV}}\left(\frac{P^t(x,\cdot)+P^{t+1}(x,\cdot)}{2},\pi(\cdot)\right)\leq\frac{1}{4}\right\} \quad \asymp \quad t_{\text{mix}}^{\text{lazy}}\left(\frac{1}{4}\right).$$

Let $t = t_{\text{ave}}\left(\frac{1}{4}\right)$. Then for any x we have

$$\begin{aligned}\frac{3}{4} \quad &\leq \quad 1-d_{\text{TV}}\left(\frac{P^t(x,\cdot)+P^{t+1}(x,\cdot)}{2},\pi(\cdot)\right)\\ &= \quad \sum_y\left(\frac{P^t(x,y)+P^{t+1}(x,y)}{2}\right)\wedge\pi(y)\\ &\leq^{9} \quad \sum_y P^t(x,y)\wedge\pi(y) \quad + \quad \sum_y P^{t+1}(x,y)\wedge\pi(y).\end{aligned}$$

This shows that for any x we have $\sum_y P^t(x,y)\wedge\pi(y)\geq\frac{3}{8}$ or $\sum_y P^{t+1}(x,y)\wedge\pi(y)\geq\frac{3}{8}$. Using that the degrees are bounded and $\pi(y)\asymp\pi(z)$ for all y, z we get

$$\sum_y P^{t+1}(x,y)\wedge\pi(y) \quad = \quad \sum_y\left(\sum_{z:z\sim y}\frac{1}{\deg(z)}P^t(x,z)\right)\wedge\pi(y) \quad \asymp \quad \sum_z P^t(x,z)\wedge\pi(z),$$

therefore in either case there exists a $\theta\in(0,1)$ such that

$$1-d_{\text{TV}}\left(P^t(x,\cdot),\pi(\cdot)\right) \quad = \quad \sum_y P^t(x,y)\wedge\pi(y) \quad \geq \quad \theta.$$

This shows that $t_{\text{mix}}(1-\theta)\leq t\asymp t_{\text{mix}}^{\text{lazy}}$. □

Proof of Lemma A.1. By [3, Theorem 1.2] we know that there exists a constant c_2 such that for all $a>0$ we have

$$t_{\text{unif}}(a) \quad \leq \quad \frac{c_2}{a}\left((mn)\wedge\frac{m^2}{a}\right) \tag{18}$$

where m is the number of edges of G,

$$t_{\text{unif}}(a) \quad = \quad \min\left\{t\geq 0:\ \max_{u,v}\left|\frac{P^t(u,v)}{\pi(v)}-1\right|\leq a\right\},$$

P is the transition matrix of the walk X and π is the corresponding invariant distribution.

Let us apply (18) with $a=\frac{\sqrt{c_2}\Delta n}{\sqrt{t}}\geq\frac{\sqrt{c_2}\Delta}{\sqrt{A}}$. We know that $m\leq\Delta n$ and $\pi(y)\leq\frac{\Delta}{n}$, hence we get that

$$t_{\text{unif}}(a) \quad \leq \quad \frac{c_2}{a}\left((\Delta n^2)\wedge\frac{\Delta^2 n^2}{a}\right) \quad \leq \quad \frac{c_2\Delta^2 n^2}{a^2} \quad = \quad t,$$

therefore

$$\left|\frac{P^t(x,y)}{\pi(y)}-1\right| \quad \leq \quad a,$$

hence

$$P^t(x,y) \quad \leq \quad (a+1)\pi(y) \quad \leq \quad a\left(1+\frac{\sqrt{A}}{\Delta\sqrt{c_2}}\right)\pi(y) \quad \leq \quad \left(c_2\Delta^2+\Delta\sqrt{A}\right)\frac{1}{\sqrt{t}}.$$

This finishes the proof. □

[9] Here we used that for any $A,B,C\in\mathbb{R}_{\geq 0}$ we have $\left(\frac{A+B}{2}\right)\wedge C\leq(A\wedge C)+(B\wedge C)$.

Proof of Lemma A.2. Let E be a renewal process with $\mathrm{Geom}_{\geq 1}\left(\frac{1}{2}\right)$ renewal times, independently of X. Then $Y_t := X_{E_t}$ is a lazy simple random walk on G from x. Let P be the transition matrix of the chain X and π the corresponding invariant distribution.

Using Lemma A.1 and that for any vertex x the transition probability $\mathbb{P}_x(X_{2t} = x)$ is non-increasing in t (see e.g. [7, Proposition 10.25 (i)]), we get that for any constant A, any $t < An^2$ and for any x we have

$$\begin{aligned}\frac{1}{\sqrt{t}} \quad &\gtrsim \quad \mathbb{P}_x(Y_{6t} = x) \quad = \quad \sum_{k=0}^{6t} \mathbb{P}(E_{6t} = k)\, P^k(x,x) \\ &\geq \quad \sum_{k=t}^{3t} \mathbb{P}(E_{6t} = 2k)\, P^{2k}(x,x) \quad \geq \quad \mathbb{P}(E_{6t} \geq 2t, E_{6t} \text{ even})\, P^{2t}(x,x).\end{aligned}$$

We know that $\mathbb{P}(E_{6t} \geq 2t) = 1 - o(1)$ and $\mathbb{P}(E_{6t} \text{ even}) \asymp 1$, hence $\mathbb{P}(E_{6t} \geq 2t, E_{6t} \text{ even}) \gtrsim 1$, and so

$$\frac{1}{\sqrt{t}} \quad \gtrsim \quad P^{2t}(x,x).$$

Since $P^{2t}(x,x)$ is non-increasing in t, we also get that $P^{2t}(x,x) \lesssim \frac{1}{\sqrt{t} \wedge n}$ for all t.

Then using reversibility, Cauchy-Schwarz and that $\pi(z) \asymp \frac{1}{n}$ for all z, we get that for any vertices x and y and for any t we have

$$P^{2t}(x,y) \quad \asymp \quad \frac{1}{n}\frac{P^{2t}(x,y)}{\pi(y)} \quad \leq \quad \frac{1}{n}\sqrt{\frac{P^{2t}(x,x)}{\pi(x)}\frac{P^{2t}(y,y)}{\pi(y)}} \quad \lesssim \quad \frac{1}{\sqrt{t} \wedge n}.$$

Then we also get that

$$P^{2t+1}(x,y) \quad = \quad \sum_{z:\, z\sim y} P^{2t}(x,z)P(z,y) \quad \lesssim \quad \frac{1}{\sqrt{t} \wedge n}.$$

This finishes the proof. □

Proof of Lemma A.3. For any time t, any state x, and any subset A of the state space we have

$$\begin{aligned}\mathbb{P}_x\left(\tau_A^Y > 2t + t^{\frac{2}{3}}\right) \quad &\leq \quad \mathbb{P}_x\left(\tau_A^X > t\right) + \mathbb{P}\left(\mathrm{Bin}\left(2t + t^{\frac{2}{3}}, \frac{1}{2}\right) \leq t\right), \qquad \text{and} \\ \mathbb{P}_x\left(\tau_A^Y < 2t - t^{\frac{2}{3}}\right) \quad &\geq \quad \mathbb{P}_x\left(\tau_A^X < t\right) - \mathbb{P}\left(\mathrm{Bin}\left(2t - t^{\frac{2}{3}}, \frac{1}{2}\right) \geq t\right).\end{aligned}$$

Using this and that $\mathrm{hit}_\alpha^{X(n)}(\theta) \gg 1$ we get the result. □

IV Estimates for walks on vertex-transitive graphs of polynomial growth

Proof of Lemma 6.28. The proof is similar to the proof of [2, Lemma 6.12].

In what follows let X be a lazy random walk on G; let $P = P_{G,\text{lazy}}$; for a set A let τ_A be the hitting time of A by X; let $B_\ell = B_G(o,\ell)$; and for a set A let

$$\alpha_A(x) := \lim_{s\to\infty} \mathbb{P}_o(X_s = x \mid \tau_A > s)$$

be the quasi-stationary distribution of P corresponding to A.
By Cauchy-Schwarz, reversibility and vertex-transitivity we get that for any time t we have

$$\max_x P^{2t+1}(o,x) \le \max_x P^{2t}(o,x) \le \max_x \sqrt{P^{2t}(o,o)P^{2t}(x,x)} = P^{2t}(o,o) \le 2P^{t+1}(o,o).$$

Hence for any t and ℓ we have

$$P^t(o,o) \quad \gtrsim \quad \frac{\mathbb{P}_o(X_t \in B_\ell)}{V(\ell)} \quad \ge \quad \frac{\mathbb{P}_o\big(\tau_{B_\ell^c} > t\big)}{V(\ell)}.$$

Using that for any $v \in B\left(o, \frac{1}{2}\ell\right)$ we have $B\left(v, \frac{1}{2}\ell\right) \subseteq B\left(o, \ell\right)$, and using transitivity we get that

$$\mathbb{P}_o\big(\tau_{B_\ell^c} > t\big) \quad \ge \quad \max_{v\in B\left(o,\frac{1}{2}\ell\right)} \mathbb{P}_o\Big(\tau_{B\left(v,\frac{1}{2}\ell\right)^c} > t\Big) \quad = \quad \max_{v\in B\left(o,\frac{1}{2}\ell\right)} \mathbb{P}_v\Big(\tau_{B\left(o,\frac{1}{2}\ell\right)^c} > t\Big).$$

This is then

$$\ge \quad \mathbb{P}_{\alpha_{B^c_{\ell/2}}}\Big(\tau_{B^c_{\ell/2}} > t\Big) \quad = \quad \mathbb{P}_{\alpha_{B^c_{\ell/2}}}\Big(\tau_{B^c_{\ell/2}} > 1\Big)^t.$$

It is known (see e.g. [1, Theorem 3.33]) that $p(r) := \mathbb{P}_{\alpha_{B_r^c}}\big(\tau_{B_r^c} > 1\big)$ satisfies

$$\frac{1}{1-p(r)} \quad = \quad \mathbb{E}_{\alpha_{B_r^c}}\big[\tau_{B_r^c}\big] \quad = \quad \sup_{\substack{f\colon f|_{B_r^c}\equiv 0,\\ f\neq\text{const}}} \frac{\langle f,f\rangle_{\pi_{B_r}}}{\langle (I-P_{B_r})f,f\rangle_{\pi_{B_r}}},$$

where π_{B_r} is π conditioned on B_r, i.e. $\pi_{B_r}(x) = \frac{\pi(x)}{\pi(B_r)}$ for $x \in B_r$. (In the current setup it is the uniform distribution on B_r.)

Plugging in $f(x) = d_G\left(x, B_r^c\right)$; noting that f is L^1-Lipschitz, hence $\langle (I-P_{B_r})f,f\rangle_{\pi_{B_r}} \le 1$; and noting that $f(x) \ge \frac{1}{2}r$ for all $x \in B_{r/2}$ we get that

$$\frac{1}{1-p(r)} \quad \ge \quad \pi_{B_r}(B_{r/2})\frac{1}{4}r^2 \quad \asymp \quad \frac{V(r/2)}{V(r)}r^2.$$

By Lemma 6.27 we get that $V(r) \asymp V(r/2)$ for all r, hence $p(r) \ge 1 - \frac{c'}{r^2}$, where c' is a positive constant.

Using this for $\ell = c\sqrt{t}$, $r = \ell/2$ we get that

$$P^t(o,o) \quad \gtrsim \quad \frac{1}{V(c\sqrt{t})}\left(1 - \frac{c'}{(c\sqrt{t}/2)^2}\right)^t \quad \asymp \quad \frac{1}{V(c\sqrt{t})}$$

where c' is some positive constant. This gives the required result. □

For the proof of the upper bound on $P^t_{G,\text{lazy}}(o,o)$ we will also need the following lemma.

Lemma IV.1. *Let G be as in Proposition 6.14 and let $P = P_{G,\mathrm{lazy}}$ be the transition matrix of the lazy simple random walk on G. For $s \in (0,1)$ let*

$$\Lambda(s) := \min_{A \neq \emptyset\colon \pi(A) \leq s} (1 - \lambda_1(P_A))$$

where P_A is the substochastic matrix obtained by restricting P to the set A (i.e. deleting the rows and columns corresponding to A^c), and $\lambda_1(P_A)$ is its largest eigenvalue. Then for any $s \in \left(0, \frac{1}{2}\right]$ we have

$$\Lambda(s) \quad \geq \quad \frac{1}{2\Delta V^{-1}(2ns)^2}$$

where $V^{-1}(k)$ denotes the minimal radius r such that $V(r) \geq k$.

Proof. Let A be any subset of the vertices with $m := |A| \leq \frac{n}{2}$ and let $r = V^{-1}(2m)$. (It exists since $2m \leq n$.) Let

$$K(x,y) := \frac{1}{V(r)} \mathbf{1}_{y \in B(x,r)}$$

Note that K is reversible with invariant distribution π. Let K_A be the substochastic matrix obtained by restricting K to the set A and let $\lambda_1(K_A)$ be the largest eigenvalue of K_A. We will compare this to $\lambda_1(P_A)$.

Note that by [1, Theorem 3.33] we have

$$1 - \lambda_1(K_A) \quad = 1 - \mathbb{P}_{\alpha_{A^c}}(\tau_{A^c} > 1) \quad = \mathbb{P}_{\alpha_{A^c}}(\tau_{A^c} = 1) \quad = \sum_{x \in A} \alpha_{A^c}(x) \frac{|A^c \cap B(x,r)|}{V(r)} \quad \geq \frac{1}{2},$$

where the probabilities concern a random walk with transition matrix K. The last $\geq$ is because $V(r) \geq 2m = 2|A|$ and so $\frac{|A^c \cap B(x,r)|}{V(r)} \geq \frac{1}{2}$ for each $x \in A$.

Also note that

$$1 - \lambda_1(K_A) \quad = \inf_{\substack{f\colon f|_{A^c} \equiv 0,\\ f \neq \mathrm{const}}} \frac{\mathcal{E}_K(f)}{\langle f, f \rangle_\pi}, \qquad \text{and} \qquad 1 - \lambda_1(P_A) \quad = \inf_{\substack{f\colon f|_{A^c} \equiv 0,\\ f \neq \mathrm{const}}} \frac{\mathcal{E}_P(f)}{\langle f, f \rangle_\pi}, \tag{19}$$

where $\mathcal{E}_K$ and $\mathcal{E}_P$ are the Dirichlet forms associated to K and P respectively.

For two vertices x and y of G let $\mathcal{P}_{xy}$ be the set of paths form x to y in G and let ν_{xy} be the probability measure on $\mathcal{P}_{xy}$ selecting a uniformly chosen geodesic from x to y in G.

Then by [7, Theorem 13.20] for any function f we have

$$\mathcal{E}_P(f) \quad \geq \quad \frac{1}{B} \mathcal{E}_K(f)$$

where B is the congestion ratio defined as

$$B := \max_{e\colon Q_P(e) \neq 0} \left(\frac{1}{Q_P(e)} \sum_{x,y} Q_K(x,y) \sum_{\Gamma\colon e \in \Gamma \in \mathcal{P}_{xy}} \nu_{xy}(\Gamma)|\Gamma| \right).$$

Here $Q_P(x,y) = \pi(x)P(x,y)$, $Q_K(x,y) = \pi(x)K(x,y)$.

For a fixed vertex v let

$$B(v) := \sum_{\substack{e\colon e=(v,\cdot),\\ Q_P(e) \neq 0}} \left(\frac{1}{Q_P(e)} \sum_{x,y} Q_K(x,y) \sum_{\Gamma\colon e \in \Gamma \in \mathcal{P}_{xy}} \nu_{xy}(\Gamma)|\Gamma| \right).$$

By vertex-transitivity we have $B(v) \geq B$. For a given v let us consider the contributions to $B(v)$ from different values of x as follows

$$f(u,v) := \sum_{\substack{e\colon e=(v,\cdot),\\ Q_P(e)\neq 0}} \left(\frac{1}{Q_P(e)} \sum_y Q_K(u,y) \sum_{\Gamma\colon e\in\Gamma\in\mathcal{P}_{uy}} \nu_{uy}(\Gamma)|\Gamma| \right).$$

Since $f(\gamma u, \gamma v) = f(u,v)$ for any vertices u, v and any graph automorphism γ, and since G is finite, we can apply the mass transport principle [8, equation (8.4)] to get

$$B(o) = \sum_u f(u,o) = \sum_u f(o,u) = \sum_u \sum_{\substack{e\colon e=(u,\cdot),\\ Q_P(e)\neq 0}} \left(\frac{1}{Q_P(e)} \sum_y Q_K(o,y) \sum_{\Gamma\colon e\in\Gamma\in\mathcal{P}_{oy}} \nu_{oy}(\Gamma)|\Gamma| \right)$$

$$= \sum_{e\colon Q_P(e)\neq 0} \left(\frac{1}{Q_P(e)} \sum_y Q_K(o,y) \sum_{\Gamma\colon e\in\Gamma\in\mathcal{P}_{oy}} \nu_{oy}(\Gamma)|\Gamma| \right).$$

Note that $Q_P(e) = \frac{1}{\Delta n}$ whenever $Q_P(e) \neq 0$; $\sum_y Q_K(o,y) = \pi(o) = \frac{1}{n}$; and any y with $Q_K(o,y) \neq 0$ is within graph distance r from o, hence we get

$$B \leq B(o) = \Delta n \sum_y Q_K(o,y) \sum_{\Gamma\in\mathcal{P}_{oy}} \nu_{oy}(\Gamma)|\Gamma|^2 \leq \Delta r^2.$$

Using this bound, the characterisations in (19) and the comparison $\mathcal{E}_P(f) \geq \frac{1}{B}\mathcal{E}_K(f)$ for all f with $f|_{A^c} \equiv 0$, $f \neq \text{const}$ we get that

$$1 - \lambda_1(P_A) \geq \frac{1-\lambda_1(K_A)}{\Delta r^2} \geq \frac{1}{2\Delta r^2}.$$

Applying this bound for all non-empty sets A with $\pi(A) \leq s$ gives the required result. □

Proof of Lemma 6.29. In what follows let $P = P_{G,\text{lazy}}$. Let $\Lambda(s)$ and $V^{-1}(r)$ be defined as in Lemma IV.1.

From [4, Corollary 2.1] we know that for any M we have

$$\left|\left|P^t(o,\cdot) - \pi(\cdot)\right|\right|_\infty \leq M \quad \text{for} \quad t \geq \int_{\frac{4}{n}}^{\frac{4}{M}} \frac{4}{s\Lambda(s)} ds =: I_M.$$

Let $M = \frac{n}{m}$ where $m \leq \frac{1}{16}n$. Then for $t \geq I_M$ we have

$$\frac{n}{m} \geq \left|\left|P^t(o,\cdot) - \pi(\cdot)\right|\right|_\infty \geq \left(\frac{P^t(o,o)}{\pi(o)} - 1\right) = n\left(P^t(o,o) - \frac{1}{n}\right),$$

hence

$$P^t(o,o) \leq \frac{1}{n} + \frac{1}{m}.$$

Now we proceed to bound I_M. Using Lemma IV.1 we get that

$$I_M \leq \sum_{j=1}^{\lceil \log_2 m\rceil} \int_{\frac{4}{n}2^{j-1}}^{\frac{4}{n}2^j} \frac{4}{s\Lambda(s)} ds \leq \sum_{j=1}^{\lceil \log_2 m\rceil} 8\Delta V^{-1}\left(8\cdot 2^j\right)^2.$$

By Lemma 6.27 we get that the above sum is dominated by the last term and this last term is $\asymp V^{-1}\left(8\cdot 2^{\lceil \log_2 m\rceil}\right)^2 \leq V^{-1}(16m)^2$. [10] Note that we needed the assumption $m \leq \frac{1}{16}n$ to ensure that all terms in the sum are defined.

This shows that

$$I_M \quad \lesssim \quad V^{-1}(16m)^2 .$$

Let C be a constant such that $I_M \leq CV^{-1}(16m)^2$. Let us fix any $t \leq C\mathrm{diam}(G)^2$ and let $m = \left\lfloor \frac{1}{16}V\left(\lfloor\sqrt{t/C}\rfloor\right)\right\rfloor$. Then $I_M \leq CV^{-1}(16m)^2 \leq CV^{-1}\left(V\left(\lfloor\sqrt{t/C}\rfloor\right)\right)^2 \leq C\lfloor\sqrt{t/C}\rfloor^2 \leq t$. [11] Hence

$$P^t(o,o) \quad \lesssim \quad \frac{1}{n}+\frac{1}{m} \quad \asymp \quad \frac{1}{V(c\sqrt{t})}$$

for any constant $c>0$. □

Proof of Lemma 6.33. First we show that $t_{\mathrm{mix}}^{G,\mathrm{lazy}}\left(\frac{1}{4}\right) \lesssim \mathrm{diam}(G)^2$.

Let $t = c\mathrm{diam}(G)^2$ where c is a sufficiently small constant so that $P^t_{G,\mathrm{lazy}}(o,o) \asymp \frac{1}{n}$ (we are using Lemma 6.28 and Lemma 6.29 here). Let $s = C\mathrm{diam}(G)^2$ where C is a sufficiently large constant. From Lemma 6.28 we know that $P^{t+s}_{G,\mathrm{lazy}}(o,o) \gtrsim \frac{1}{n}$ where the constant in $\gtrsim$ does not depend on C.

Let a_1, a_2 and a_3 be constants such that $P^t_{G,\mathrm{lazy}}(o,o) \leq a_1\frac{1}{n}$, $P^{t+s}_{G,\mathrm{lazy}}(o,o) \geq a_2\frac{1}{n}$ for all C, and in Lemma 6.30 we have

$$\left|P^{t+s}_{G,\mathrm{lazy}}(o,x) - P^{t+s}_{G,\mathrm{lazy}}(o,y)\right| \quad \leq \quad a_3 d(x,y)\frac{1}{\sqrt{s}}P^t_{G,\mathrm{lazy}}(o,o). \tag{20}$$

Let $\theta \in (0,1)$ be a sufficiently small constant and let us choose C such that $\frac{a_3}{\sqrt{C}}\frac{a_1}{a_2} \leq \theta$.

Then by (20) for any o and x we have

$$\left|P^{t+s}_{G,\mathrm{lazy}}(o,x) - P^{t+s}_{G,\mathrm{lazy}}(o,o)\right| \quad \leq \quad \frac{a_3}{\sqrt{C}}P^t_{G,\mathrm{lazy}}(o,o) \quad \leq \quad \theta P^{t+s}_{G,\mathrm{lazy}}(o,o),$$

i.e. for all o and x we have

$$(1-\theta)P^{t+s}_{G,\mathrm{lazy}}(o,o) \quad \leq \quad P^{t+s}_{G,\mathrm{lazy}}(o,x) \quad \leq \quad (1+\theta)P^{t+s}_{G,\mathrm{lazy}}(o,o).$$

Choosing θ sufficiently small this shows that $t_{\mathrm{mix}}^{G,\mathrm{lazy}}\left(\frac{1}{4}\right) \leq t+s \asymp \mathrm{diam}(G)^2$.

Then by Lemma 6.11 we also get that $t^G_{\mathrm{mix}}(1-\theta) \lesssim \mathrm{diam}(G)^2$ for some $\theta \in (0,1)$.

Now we show that for any $\theta \in (0,1)$ we have $t_{\mathrm{mix}}^{G,\mathrm{lazy}}(\theta) \gtrsim \mathrm{diam}(G)^2$.

From [10, Corollary 2.8] we know that for any sufficiently large n the graph G has (c,a)-moderate growth for some a and c (meaning that $V(k) \geq \frac{n}{c}\left(\frac{n}{\mathrm{diam}(G)}\right)^a$ for all $k \leq \mathrm{diam}(G)$). Then by [5, Proposition 11.1] we get that $t_{\mathrm{rel}} \asymp \mathrm{diam}(G)^2$. Then using [7, Theorem 12.5] gives $t_{\mathrm{mix}}^{G,\mathrm{lazy}}(\theta) \gtrsim t_{\mathrm{rel}} \asymp \mathrm{diam}(G)^2$ for all $\theta \in (0,1)$.

---

[10] $r_j := V^{-1}\left(8\cdot 2^j\right)$ and let L be as in Lemma 6.27. Then $2^{J-j} = \frac{8\cdot 2^J}{8\cdot 2^j} \leq \frac{V(r_J)}{V(r_j-1)} \lesssim \left(\lceil\frac{r_J}{r_j-1}\rceil\right)^L \asymp \left(\frac{r_J}{r_j}\right)^L$. This shows that $\sum_{j=1}^J r_j^2 \lesssim r_J^2\sum_{j=1}^J 2^{-2(J-j)/L} \asymp r_J^2$.

[11] Here we used that for $r \leq \mathrm{diam}(G)$ we have $V^{-1}(V(r)) = r$.

By [9, Corollary 9.5] we also get a lower bound of order $\operatorname{diam}(G)^2$ for the mixing time if instead of a lazy walk we consider a p-lazy walk on G for some $p \in (0,1)$ bounded away from 0 and 1, i.e. a walk with transition matrix $pI + (1-p)P_G$.

Finally we show that $t_{\text{mix}}^G(\theta) \gtrsim \operatorname{diam}(G)^2$ for all $\theta \in (0,1)$.

Let H be a graph obtained as follows. Let H have the same vertex set as G. For each pair $\{x, y\}$ with $x \neq y$ let H have twice as many edges between x and y as the number of paths of length 2 between x and y in G, and for each x let it have $\deg_G(x)$ loops from x. Note that the simple random walk on this graph H has transition matrix P^2.

Also note that each vertex in H has degree $2\deg_G(o)^2$ and has exactly $\deg_G(o)$ loops. Let $\widetilde{G}$ be a graph obtained by deleting all loops in H.

Then $\widetilde{G}$ is a vertex-transitive graph with bounded degrees and polynomial growth of balls (and having multiple edges). Also either $\widetilde{G}$ is connected (if G is not bipartite) or it has two connected components of size $\frac{n}{2}$ (if G is bipartite). Note that the transition matrix of the SRW on $\widetilde{G}$ satisfies $\frac{1}{\deg_G(o)} I + \left(1 - \frac{1}{\deg_G(o)}\right) P_{\widetilde{G}} = P^2$. Also each connected component of $\widetilde{G}$ has diameter $\asymp \operatorname{diam}(G)$.

Using the above result for the $\frac{1}{\deg_G(o)}$-lazy random walk on one connected component of $\widetilde{G}$ we get that $t_{\text{mix}}^G(\theta) \gtrsim \operatorname{diam}(G)^2$ for all $\theta \in (0,1)$. $\square$

References

[1] David Aldous and James Allen Fill. *Reversible Markov Chains and Random Walks on Graphs.* unfinished monograph, 2002.

[2] Nathanaël Berestycki, Jonathan Hermon, and Lucas Teyssier. On the universality of fluctuations for the cover time. 2022.

[3] Lucas Boczkowski, Yuval Peres, and Perla Sousi. Sensitivity of mixing times in Eulerian digraphs. *SIAM Journal on Discrete Mathematics*, 32, 03 2016.

[4] Sharad Goel, Ravi Montenegro, and Prasad Tetali. Mixing Time Bounds via the Spectral Profile. *Electronic Journal of Probability*, 11:1 – 26, 2006.

[5] Jonathan Hermon and Richard Pymar. The exclusion process mixes (almost) faster than independent particles. *The Annals of Probability*, 48(6):3077 – 3123, 2020.

[6] Jonathan Hermon, Allan Sly, and Perla Sousi. Universality of cutoff for graphs with an added random matching. *The Annals of Probability*, 50(1):203 – 240, 2022.

[7] David A. Levin, Yuval Peres, and Elizabeth L. Wilmer. *Markov chains and mixing times.* American Mathematical Society, 2006.

[8] Russell Lyons and Yuval Peres. *Probability on Trees and Networks.* Cambridge Series in Statistical and Probabilistic Mathematics. Cambridge University Press, 2017.

[9] Yuval Peres and Perla Sousi. Mixing times are hitting times of large sets. *Journal of Theoretical Probability*, 28, 07 2011.

[10] R. Tessera and M.C.H. Tointon. A finitary structure theorem for vertex-transitive graphs of polynomial growth. *Combinatorica*, 41:263–298, 2021.
